# Copper‐Catalyzed 1,2‐Dicarbonylative Cyclization of Alkenes with Alkyl Bromides via Radical Cascade Process

**DOI:** 10.1002/anie.202214812

**Published:** 2022-11-10

**Authors:** Fengqian Zhao, Xing‐Wei Gu, Robert Franke, Xiao‐Feng Wu

**Affiliations:** ^1^ Leibniz-Institut für Katalyse e.V. 18059 Rostock Germany; ^2^ Dalian National Laboratory for Clean Energy Dalian Institute of Chemical Physics Chinese Academy of Sciences 116023 Liaoning Dalian China; ^3^ Evonik Performance Materials GmbH Paul-Baumann-Str. 1 45772 Marl Germany; ^4^ Lehrstuhl für Theoretische Chemie Ruhr-Universität Bochum 44780 Bochum Germany

**Keywords:** 1,2-Dicarbonylation, 1,4-Diketone, Alkyl Bromides, Cyclization, Radical Cascade Reaction

## Abstract

Herein, we developed a new procedure on 1,2‐dicarbonylative cyclization of 4‐aryl‐1‐butenes with alkyl bromides. Using simple copper catalyst, two molecules of carbon monoxide were introduced into the double bond with the formation of four new C−C bonds and a new ring. Various *α*‐tetralones and 2,3‐dihydroquinolin‐4‐ones were formed in moderate to good yields.

## Graphical abstract

Here, we have described a new copper‐catalyzed 1,2‐dicarbonylative cyclization of 4‐aryl‐1‐butenes with unactivated alkyl bromides. In this reaction, two carbonyl groups were introduced into the double bond by the insertion of two molecules of carbon monoxide, and four new C−C bonds could be constructed in one reaction. At the same time, the corresponding skeletons of *α*‐tetralones as well as 2,3‐dihydroquinolin‐4‐one were obtained smoothly, which could also be transformed into three consecutive ring structures via subsequent derivatizations.

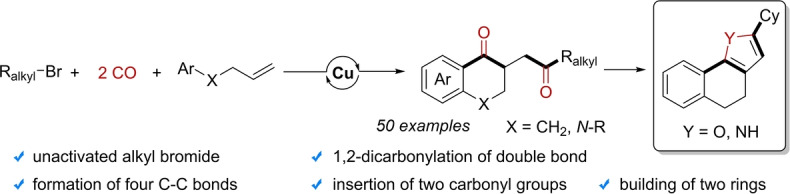

## Introduction

**Scheme 1:**
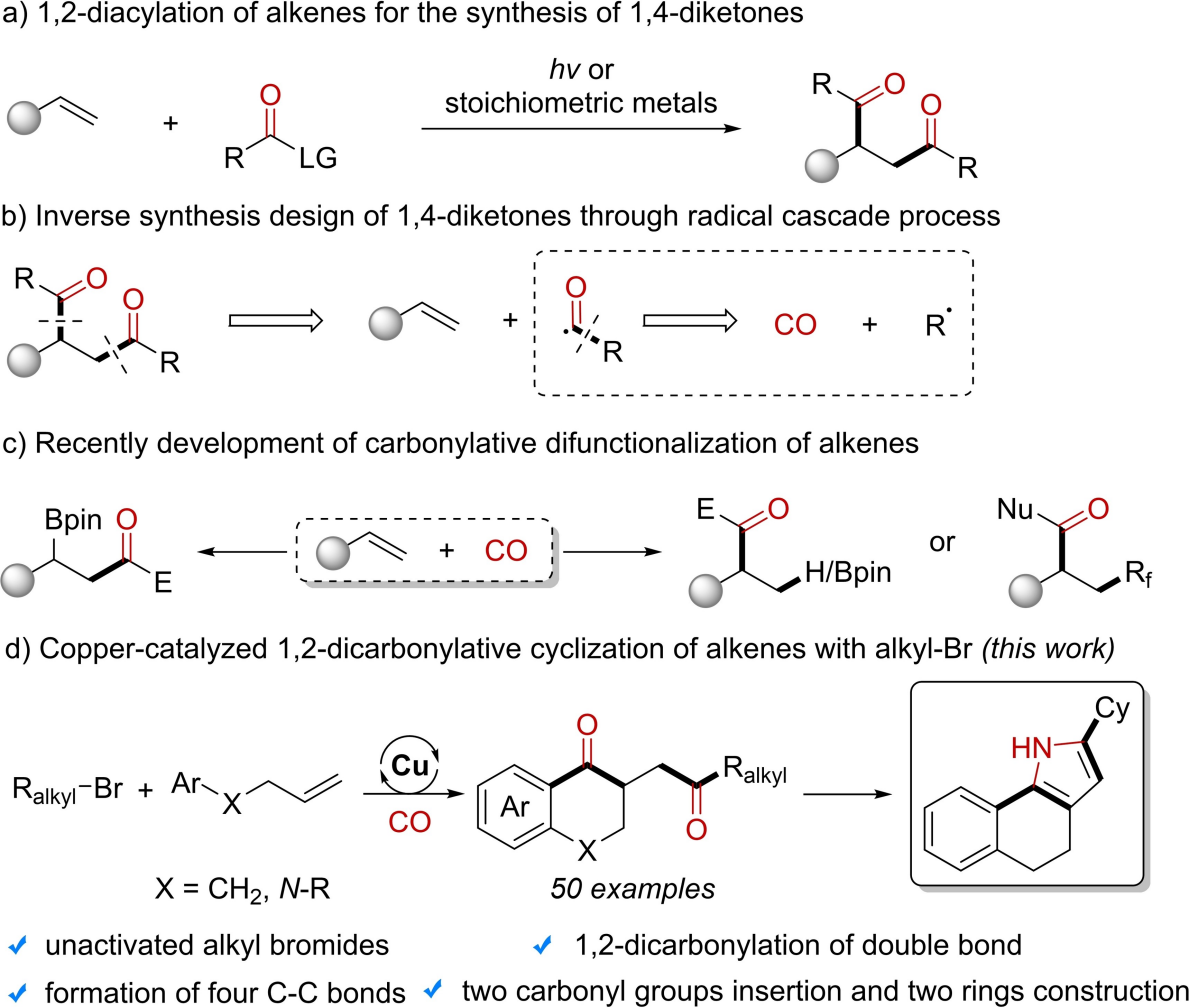
1,2‐dicarbonylation of double bond.

As a common structural backbone, 1,4‐diketones are widely present in pharmaceuticals and bioactive nature molecules.^[1]^ Additionally, they are also important building blocks in organic transformations.^[2]^ Among the various methods for their synthesis,^[3]^ the strategy of introducing two carbonyl groups into one double bond is both an opportunity and a challenge. In the limited reports on 1,2‐diacylation of alkenes by photocatalysis or stoichiometric metal regents,^[4]^ acyl chlorides and *α*‐ketocarboxylic acid are usually used as the source of acyl groups (Scheme 1a). On the other hand, the construction of C−C bonds is one the key transformations in organic synthesis. The final goal is the simultaneous construction of multiple chemical bonds in one single chemical operation, which could rapidly increase the compounds complexity by using simple small molecules. Carbon monoxide, as an abundant and cheap C1 resource, is widely used in the carbonylation of unsaturated bonds through the formation of acyl species, which could produce at least two or three new bonds in one reaction.^[5]^ Hence, the application of CO as the source of carbonyl group can be considered as a solution for the diacylation of alkenes (Scheme 1b). In recent years, various strategies for the carbonylative difunctionalization of alkenes have been reported by our group and other groups (Scheme 1c).[^6^, ^7^] Although these strategies have achieved selective control of the alkene carbonylation sites, only one carbonyl group was introduced into the final product for most of the procedures. The addition of acyl radicals to double bond occurred within the molecule and only applicable to substrates with a specific structure.^[7]^ Therefore, the 1,2‐dicarbonylation of alkenes for the synthesis of 1,4‐diketones using CO as the source of carbonyl groups still remains a huge challenge.

On the other hand, *α*‐tetralones play an indispensable role in the structural motifs of various natural molecules and bioactive compounds, such as anthracyclines, estrones, tetracyclines as well as their derivatives.^[8]^ However, methods for synthesizing these compounds are still very limited compared with their broad utility, which usually rely on the Friedel–Crafts reaction of arylbutyric acids^[9]^ and the cyclization of substrates with special structures.^[10]^ With the development of radical chemistry, the radical cyclization with aromatic rings has been established as a convenient and powerful approach and various benzocyclic compounds have been constructed efficiently.^[11]^ Unfortunately, most of these methods involved the cyclization of alkyl radicals with aromatic rings. To the best of our knowledge, there are almost no strategies for the intramolecular cyclization of acyl groups with aromatic rings to synthesize *α*‐tetralone compounds.

Herein, we developed a procedure on 1,2‐dicarbonyaltive cyclization of 4‐aryl‐1‐butenes with alkyl bromides for the synthesis of 1,4‐diketones (Scheme 1d). In this system, four new C−C bonds were constructed, two carbonyl groups, and one ring have been introduced to construct the backbone of *α*‐tetralones as well as 2,3‐dihydroquinolin‐4‐one through the radical cascade process. In addition, the construction of three consecutive ring structures were also successfully achieved in the subsequent transformations.

## Results and Discussion

At the beginning of this study, bromocyclohexane **1 a** and 4‐phenyl‐1‐butene **2 a** were used as model substrates in anisole at 70 °C under 40 bar of CO. A variety of copper catalysts were firstly screened by using bpy as the ligand, most of which barely allowed the reaction to proceed (Table 1, entries 1–4). The delightful yield of 1,2‐dicarbonylative cyclization product **3 aa** was obtained when CuBr(Me_2_S) was used as the catalyst (Table 1, entry 5). In testing the effects of bases, poor yields were obtained with either other inorganic bases or organic bases (Table 1, entries 6 and 7; see Supporting Information for more details). Increasing the loading of additive had a slight increase in the reaction effect. And a similar result was obtained when TMEDA was used instead of DABCO (Table 1, entries 8 and 9). Subsequently, the effect of ligands was tested (Table 1, entries 10 and 11). Moderate yields were obtained when bipyridine ligands with different substituents or 1,10‐phenanthroline were employed in the system. Triarylphosphines delivered the desired product with similar results. However, a significant decrease in the yield was observed when triarylphosphine with electron‐withdrawing groups was used. Finally, the best result was obtained with phosphite **L6** as the ligand. For other common phosphine ligands, Cy‐Johnphos gave a poor yield, while other bisphosphine ligands, such as BINAP, Xantphos, and DPPE, barely produce the product (see Supporting Information for more ligands screening). When the reaction was carried out under the optimized conditions with a scale of 0.2 mmol, a reduced yield was observed, which may be due to the reduction of relative concentration of CO in the reaction solution (Table 1, entry 12). Finally, 60 % yield was obtained after 24 h of the reaction by increasing the amount of solvent and the pressure of CO (Table 1, entries 13 and 14). Concerning the necessity of the catalyst components, control experiments were performed (See Table S1). Decreased yield was obtained in the absence of acac or the other additive. Here, we believe acac acts as soft coordinating ligand to maintain the reactivity of copper center. It is also worth noting that only trace amounts of monocarbonylation by‐products were detected during the whole optimization process.

**Table 1:** Optimization of the reaction conditions.^[a]^

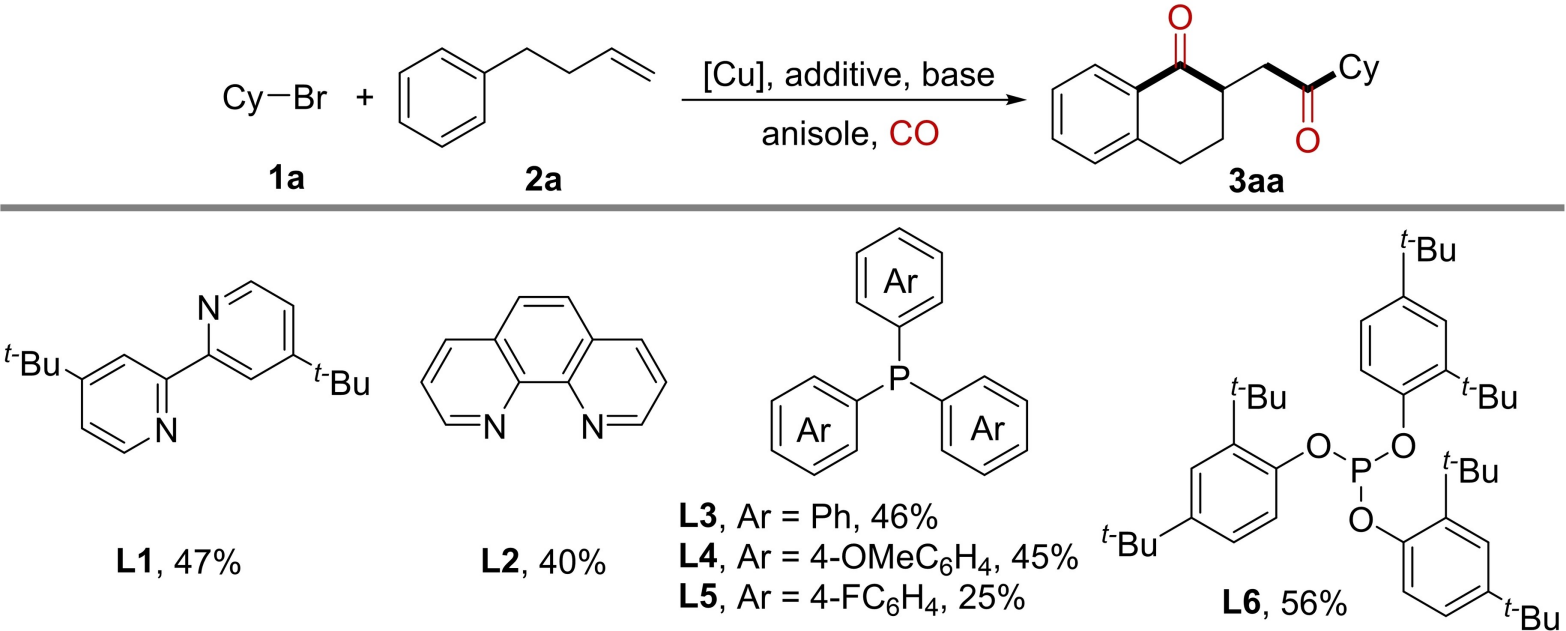					
Entry	[Cu]	Ligand	Add. [mol %]	Base	**3 aa** [%]
1	CuI	bpy	TMEDA (10)	K_3_PO_4_	7
2	CuCl_2_	bpy	TMEDA (10)	K_3_PO_4_	6
3	Cu(OAc)_2_	bpy	TMEDA (10)	K_3_PO_4_	22
4	Cu(acac)_2_	bpy	TMEDA (10)	K_3_PO_4_	9
5	CuBr(Me_2_S)	bpy	TMEDA (10)	K_3_PO_4_	44
6	CuBr(Me_2_S)	bpy	TMEDA (10)	Cs_2_CO_3_	8
7	CuBr(Me_2_S)	bpy	TMEDA (10)	^*t*‐^BuOK	trace
8	CuBr(Me_2_S)	bpy	TMEDA (20)	K_3_PO_4_	51
9	CuBr(Me_2_S)	bpy	DABCO (20)	K_3_PO_4_	53
10	CuBr(Me_2_S)	**L1**–**L5**	DABCO (20)	K_3_PO_4_	25 ‐ 47
11	CuBr(Me_2_S)	**L6**	DABCO (20)	K_3_PO_4_	56
12^[b]^	CuBr(Me_2_S)	**L6**	DABCO (20)	K_3_PO_4_	46
13^[b,c]^	CuBr(Me_2_S)	**L6**	DABCO (20)	K_3_PO_4_	51
14^[b,c,d]^	CuBr(Me_2_S)	**L6**	DABCO (20)	K_3_PO_4_	60 (57)^[e]^

[a] Reaction condition: **1 a** (0.2 mmol, 2.0 eq.), **2 a** (0.1 mmol, 1.0 eq.), [Cu] (10 mol %), ligands (15 mol % for bidentate ligands and 30 mol % for monodentate ligands), acac (20 mol %), base (3.0 eq.), anisole (0.5 mL), CO (40 bar), 70 °C, 15 h. The yield was determined by GC, using hexadecane as the internal standard. [b] 0.2 mmol scale. [c] Anisole (1.5 mL). [d] CO (50 bar), 24 h. [e] Isolated yield. bpy=2, 2′‐bipyridine. acac=acetylacetone. TMEDA=*N*, *N*, *N′*, *N′*‐tetramethylethylenediamine. DABCO=triethylenediamine.

**Scheme 2:**
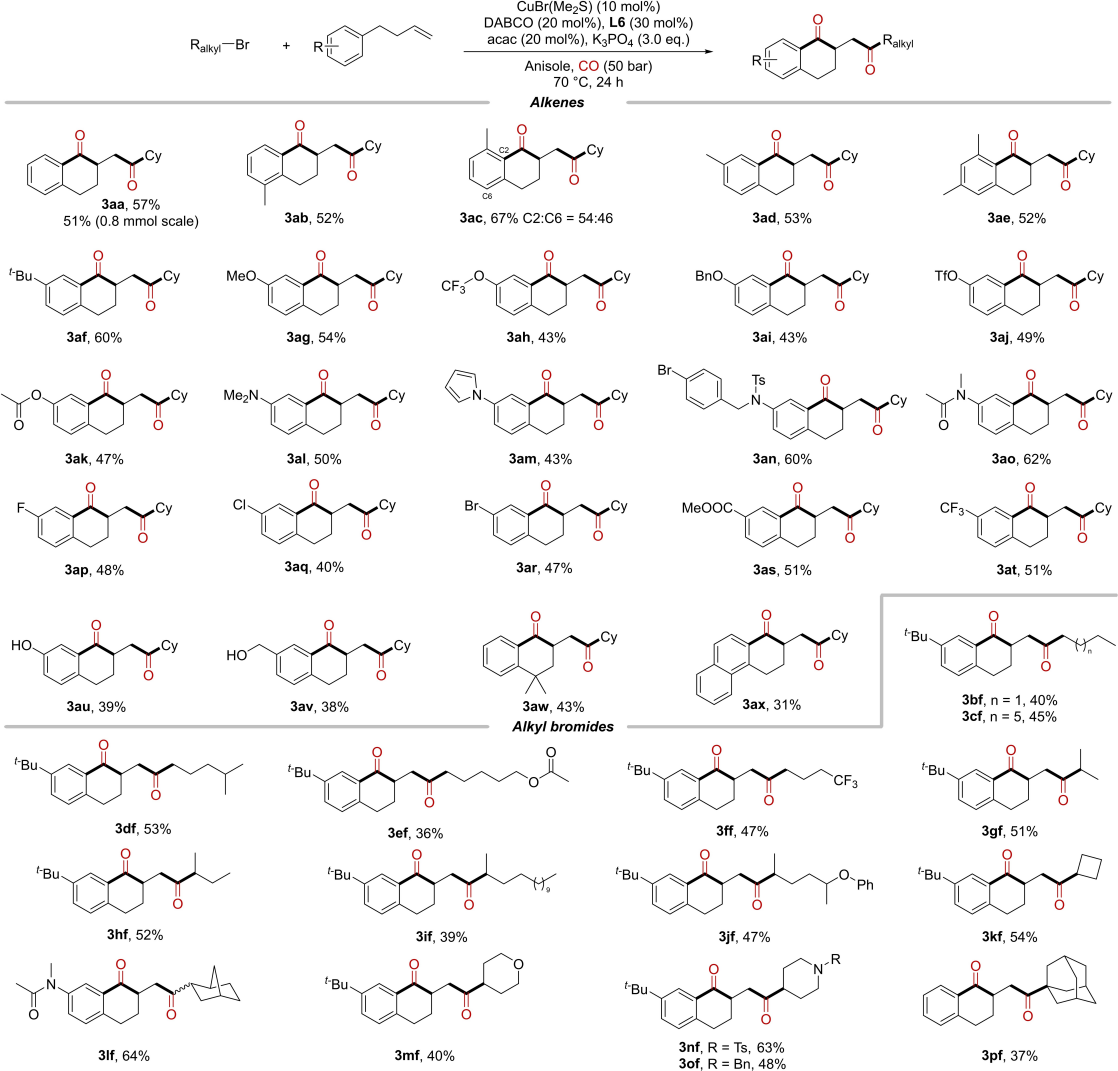
Scope of 1,2‐dicarbonylative cyclization of alkyl bromides with 4‐aryl‐1‐butenes. Reaction conditions: alkyl bromides **1** (0.4 mmol, 2.0 eq.), alkenes **2** (0.2 mmol, 1.0 eq.), CuBr(Me_2_S) (10 mol %), **L6** (30 mol %), DABCO (20 mol %), acac (20 mol %), K_3_PO_4_ (3.0 eq.), Anisole (1.5 mL), CO (50 bar), 70 °C, 24 h.

With the optimized reaction conditions in hand, we examined the scope of substrates in this reaction. As shown in Scheme 2, a variety of alkenes were firstly explored, and the corresponding products were delivered in moderate yields. Both *ortho*‐, *meta*‐, and *para*‐substituted alkenes were converted into the desired products smoothly, for *meta*‐substituted alkene, two cyclization products at different positions were obtained in almost equal amounts (**3 ab**–**3 ad**). Dimethyl‐substituted and *tert*‐butyl substituted alkenes can also be efficiently converted in this system (**3 ae**–**3 af**). In addition to alkyl substituents, other heteroatomic groups, such as methoxy (**3 ag**), trifluoromethoxy (**3 ah**), benzyloxy (**3 ai**), trifluoromethanesulfonate (**3 aj**), acetoxy (**3 ak**), *N*, *N*‐dimethyl (**3 al**), and pyrrolyl (**3 am**) were all tolerated well in the reaction. It is worth noting that when there is a sulfonamide or amide group on the substrate, the reaction was hardly occurred due to the existing of NH group. However, 60–62 % yields could also be obtained when the hydrogen atom was replaced by benzyl or methyl groups (**3 an** and **3 ao**). For substrate with electron‐withdrawing groups, such as fluorine (**3 ap**), chlorine (**3 aq**), bromine (**3 ar**), ester (**3 as**), and trifluoromethyl (**3 at**), the corresponding products were obtained with a slightly decreased. Unlike the inhibition of the reaction by NH groups, phenolic and benzyl hydroxyl groups could be tolerated in the reaction and gave the corresponding products in moderate yields (**3 au** and **3 av**). Besides the groups on the benzene ring, the effect of substituents on the carbon chain was also examined. 4,4‐Dimethyl‐substituted alkenes could be transformed smoothly (**3 aw**). Regrettably, the reaction was hardly occurred when there were other substituents on the double bond (see Supporting for more details). This cyclization can also proceed to naphthalene ring (**3 ax**) but was not applied to the indole or thiophene rings. Subsequently, the compatibility of alkyl bromides in the reaction was also tested using 1‐(but‐3‐en‐1‐yl)‐4‐(*tert*‐butyl)benzene **2 f** as the coupling partner. Primary and secondary alkyl bromides with different lengths of carbon chains were smoothly converted into the desired products in moderate yields (**3 bf**–**3 jf**). Various groups on the carbon chain, such as ester (**3 ef**), trifluoromethyl (**3 ff**), and ether (**3 jf**) were tolerated as well.

For cyclic alkyl bromides, four‐, six‐membered carbocycles and oxygen‐ or nitrogen‐containing heterocycles were all transformed smoothly and delivered the desired products in 40–64 % yields (**3 kf**–**3 of**). In addition, sterically hindered tertiary bromide could also be used in this transformation, despite with low yield (**3 pf**). Finally, our model system was performed on 0.8 mmol scale as well, and 51 % yield of the desired product was obtained. For the related analogue of alkenes, such as allylbenzene and pent‐4‐en‐1‐ylbenzene, they were also tested under our standard conditions with bromocyclohexane as the reaction partner and no desired product could be detected.

**Scheme 3:**
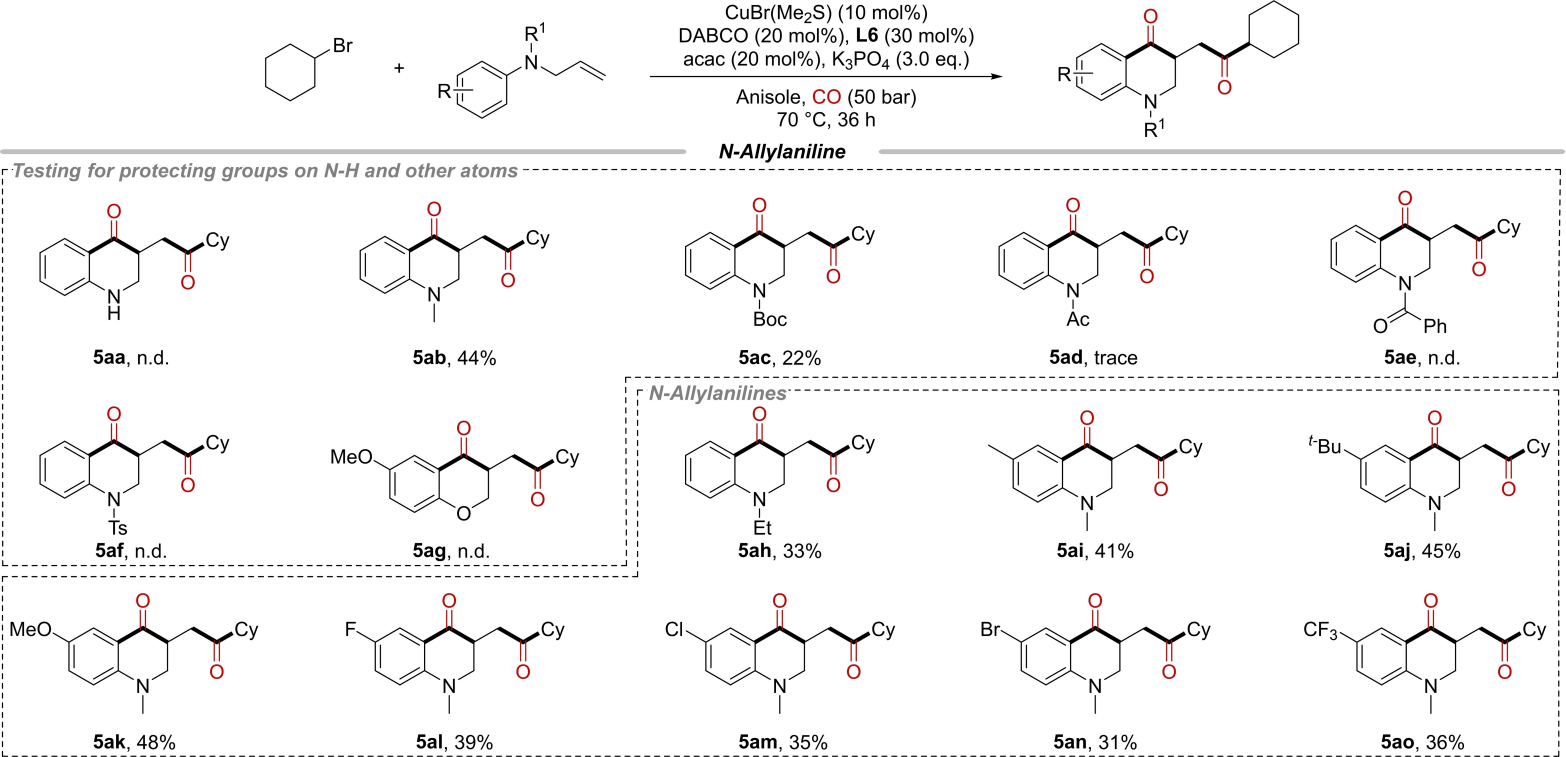
Scope of 1,2‐dicarbonylative cyclization of alkyl bromides with *N*‐allylanilines. Reaction conditions: Bromocyclohexane **1 a** (0.4 mmol, 2.0 eq.), *N*‐allylanilines **4** (0.2 mmol, 1.0 eq.), CuBr(Me_2_S) (10 mol %), **L6** (30 mol %), DABCO (20 mol %), acac (20 mol %), K_3_PO_4_ (3.0 eq.), Anisole (1.5 mL), CO (50 bar), 70 °C, 36 h.

2,3‐Dihydro‐4(1*H*)‐quinolinone and their derivatives are commonly occur and important backbones in the structure of natural products and medicines.^[12]^ Additionally, they are also identified as valuable synthetic precursors in the synthesis of various pharmaceutical and bioactive molecules.^[13]^ In the previous works, 2, 3‐dihydro‐4(1*H*)‐quinolinones were generally prepared from *ortho*‐aminoaryl ketones, such as acid‐ or base‐catalyzed isomerization of *ortho*‐aminochalcones,^[14]^ and the cyclization of *ortho*‐aminoacetophenones with aryl aldehydes.[^15^, ^16^] The synthesis was limited by the abundancy of substrates. On the contrary, aniline is an abundant and inexpensive raw material in industrial production, and *N*‐allylanilines could be easily obtained from anilines and allyl halides. Here, we also explored the possibility of synthesizing 2,3‐dihydro‐4(1*H*)‐quinolinone from *N*‐allylanilines in this radical cascade process. As shown in Scheme 3, *N*‐allylaniline **4 a** was used first in the reaction system, similar to previous work,^[17]^ the double carbonylation occurred on the secondary amine and no desired product was detected (**5 aa**). Then, various protecting groups were used to replace hydrogen atoms. 44 % and 22 % yields were obtained when the NH group was protected by methyl and Boc groups, respectively (**5 ab** and **5 ac**). For substrates with other protecting groups, such as acetyl, benzoyl, and sulfonyl, were hardly delivered the corresponding product (**5 ad**–**5 af**). When oxygen atom was used to replace the NH group, the reaction was unable to occur (**5 ag**). Subsequently, the scope of substrates for the synthesis of 2,3‐dihydro‐4(1*H*)‐quinolinone was examined. Other alkyl substitutes on NH groups were tested firstly. The corresponding products was delivered in 33 % yield when ethyl group was used (**5 ah**) and only 18 % yield was obtained with cyclohexyl as the protecting group. In addition, various *N*‐allylanilines with different electronic effects could be converted smoothly in this reaction and gave the desired 2,3‐dihydro‐4(1*H*)‐quinolinone derivatives in moderate yields (**5 ai**–**5 ao**). It is important to mention that no desired product could be detected when *N*‐methyl‐*N*‐vinylaniline or *N*‐(but‐3‐en‐1‐yl)‐*N*‐methylaniline was tested.

**Scheme 4:**
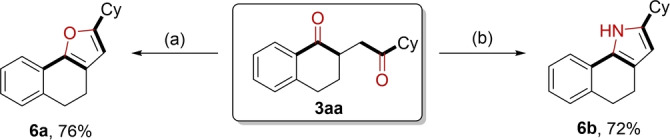
Derivatization of 1,4‐diketone compounds. Reaction conditions: a) TsOH⋅H_2_O (1.5 eq.), Toluene, 75 °C, 12 h; b) NH_4_OAc (8 eq.), DMF, 120 °C, 12 h.

As a valuable synthetic precursor, 1,4‐diketone is widely used in the synthesis of furan and pyrrole rings. Here, we also tested the possibility of our products for their synthesis. As shown in Scheme 4, the corresponding furan and pyrrole rings with three consecutive ring structures could be obtained in good yields (**6 a** and **6 b**).

**Scheme 5:**
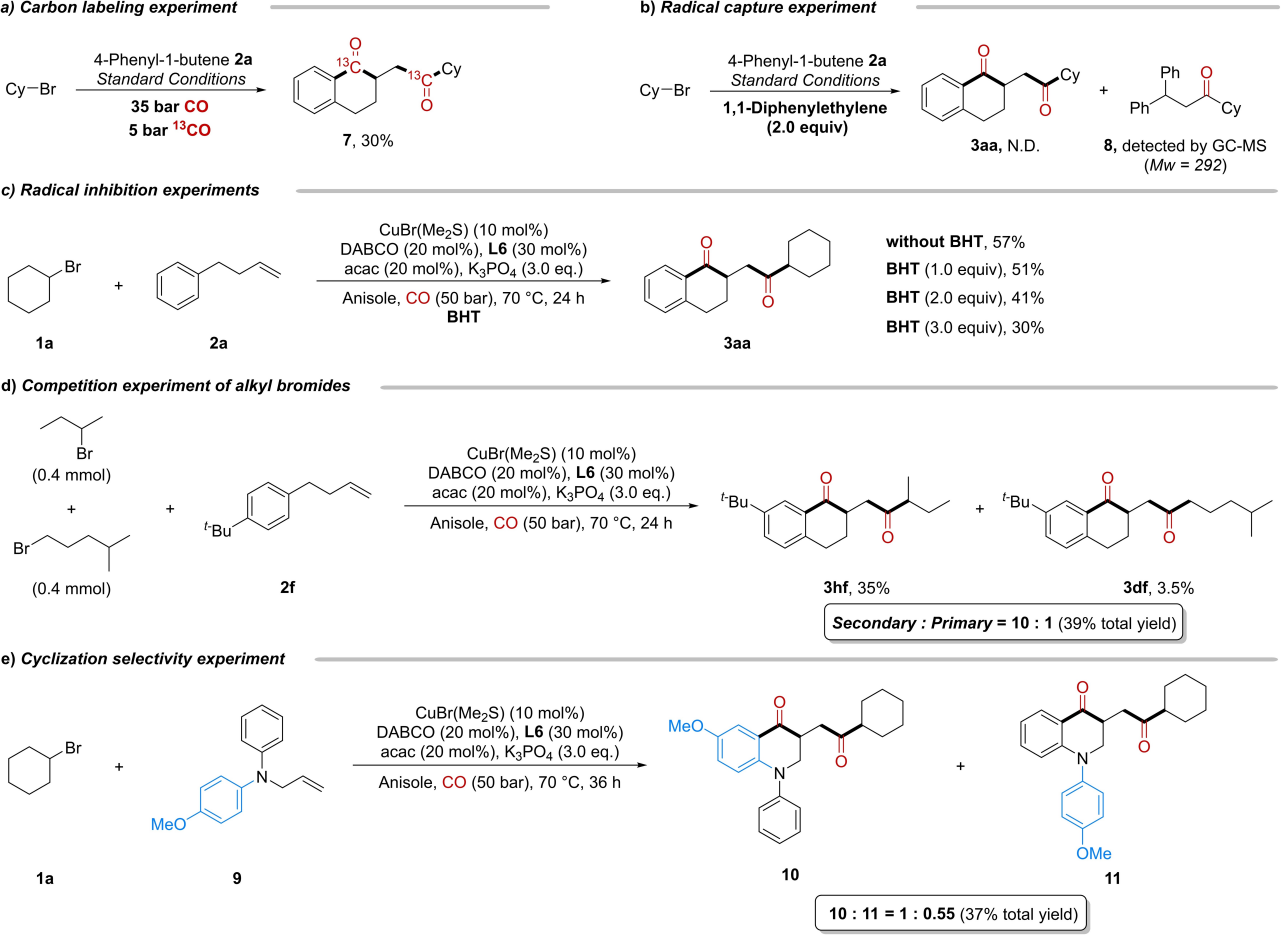
Mechanistic experiments.

For a better mechanistic understanding, several control experiments were performed. Firstly, when the reaction was carried out under a mixture gas of 35 bar of CO and 5 bar of ^13^CO, the carbon labeling product **7** was obtained in 30 % yield (Scheme 5a). This not only indicates the source of the carbonyl group in the product, but also provides a new method to introduce carbon labeling in the molecular backbones, which has important applications in the field of pharmaceutical research. Worthy to mention that yield of the carbon labeling product **7** was calculated based on average molecular weight of four possible compounds. Subsequently, the reaction was completely inhibited by 1,1‐diphenylethylene and the corresponding alkyl and acyl radical were captured by the olefin (Scheme 5b; see Supporting Information for more details). This suggests that these radicals may exist during the reaction process. In the radical inhibition experiments, the yields gradually decreased with the gradual addition of **BHT** (2,6‐di‐*tert*‐butyl‐4‐methylphenol, a radical scavenger, 0–3 equiv) (Scheme 5c). Notably, as radical clock experiments, reactions using (bromomethyl)cyclopropane or 6‐bromohex‐1‐ene as the substrates with 4‐phenyl‐1‐buten as the partner were performed. In these two cases, the reactions were totally inhibited with full consumption of (bromomethyl)cyclopropane when it was applied. However, no wished radical captured compounds could be detected neither. These might due to the truth that this reaction is very sensitive to alkene functional group. The reaction can be fully inhibited in the presence of another alkene which was found in our competition reaction with two alkenes. Differences in reaction rates between secondary and primary alkyl bromides in this system were also tested. The corresponding product delivered from secondary bromide was in higher yields than the one obtained from primary alkyl bromide (Scheme 5d), this indicates that secondary alkyl bromide could be converted easier. We also studied the selectivity of cyclization on benzene rings with different electronic nature. As shown in Scheme 5e, when the allylamine attached with two benzene rings **9** was used in the reaction, the cyclization reaction preferred to occur on the benzene rings with electron‐donating groups.

**Scheme 6:**
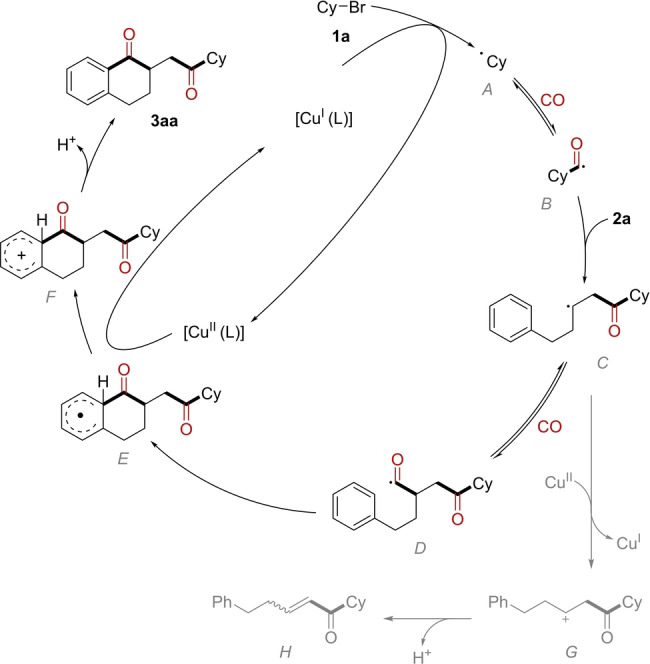
Proposed mechanism.

On the basis of above results and also previous reports,[^7^, ^18^] a possible radical cascade process was proposed (Scheme 6). Initially, alkyl radical **A** was formed from **1 a** through a *SET* process with Cu^I^, then this radical was captured by CO to give an acyl radical **B** intermediate. Then a radical addition reaction between acyl radical and double bond occurred and produce a new radical **C**, the second CO molecule could be captured by **C** to form another acyl radical **D**. Then the acyl radical addition to the benzene ring and followed by oxidation and re‐aromatized to give product **3 aa** through a deprotonation process. The radical **C** may also be oxidized by Cu^II^ to give a cation **G**, then the by‐product **H** was delivered via deprotonation. which will be oxidized by Cu^II^ to give an acyl cation **E**.

## Conclusion

We have developed a new copper‐catalyzed 1,2‐dicarbonylative cyclization of alkyl bromides with alkenes for the synthesis of 1,4‐diketones. The compounding backbones of *α*‐tetralones as well as 2,3‐dihydroquinolin‐4‐one were obtained in mild reaction conditions. In this reaction, four new C−C bonds, two carbonyl groups, and one carbon cyclic or heterocyclic ring were constructed in one reaction. Three consecutive ring structures can also be obtained in the subsequent transformations.

## Conflict of interest

The authors declare no conflict of interest.

1

## Supporting information

As a service to our authors and readers, this journal provides supporting information supplied by the authors. Such materials are peer reviewed and may be re‐organized for online delivery, but are not copy‐edited or typeset. Technical support issues arising from supporting information (other than missing files) should be addressed to the authors.

Supporting Information

## Data Availability

The data that support the findings of this study are available in the supplementary material of this article.
