# Supplementary material for: Copper‐Catalyzed 1,2‐Dicarbonylative Cyclization of Alkenes with Alkyl Bromides via Radical Cascade Process
Source: Angew Chem Int Ed Engl. 2022 Nov 10;61(49):e202214812. doi: 10.1002/anie.202214812 (PMC10100518; doi:10.1002/anie.202214812)
Supplement: Supplementary file 1 — Supporting Information [file ANIE-61-0-s001.pdf]

Angewandte Chemie
Eine Zeitschrift der Gesellschaft Deutscher Chemiker

Supporting Information

Copper-Catalyzed 1,2-Dicarbonylative Cyclization of Alkenes with Alkyl Bromides via Radical Cascade Process

*F. Zhao, X.-W. Gu, R. Franke, X.-F. Wu**

Supporting Information

Table of Contents

1. General information S1

2. Optimization studies..S2

3. General procedure for the synthesis of substrates...S4

4. General procedure for the carbonylation of alkyl bromides with alkenes.........S13

5. Characterization and procedure of the products..S14

6. Derivatization of 1,4-diketones S31

7. Mechanism and competition studies S32

8. Reference...S39

9. NMR spectra of products: ^{1}H, ^{13}C and ^{19}F NMR..S40

1. General information

Unless otherwise noted, materials were purchased from commercial suppliers and used without further purification. Flash column chromatography was performed using 200-300 mesh silica gel. ^{1}H NMR spectra were recorded on 300 or 400 MHz spectrophotometers. Chemical shifts are reported in ppm relative to tetramethylsilane (TMS) with the solvent resonance employed as the internal standard ($CDCl_3$: δ = 7.26 ppm). ^{13}C NMR was recorded at 75 MHz or 101 MHz: chemical shifts are reported in ppm from tetramethylsilane (TMS) with the solvent resonance as the internal standard ($CDCl_3$: δ = 77.00 ppm). Electron impact (EI) mass spectra were recorded on AMD 402 mass spectrometer (70 eV). High resolution mass spectra (HR-MS) were recorded on Agilent 6210. The data were given as mass units per charge (m/z). Gas chromatography analysis was performed on an Agilent HP-5890 instrument with a FID detector and HP-5 capillary column (polydimethylsiloxane with 5% phenyl groups, 30 m, 0.32 mm i.d., 0.25 μm film thickness) using argon as carrier gas.

4-Bromo-1-tosylpiperidine (**1n**), [1a] methyl 4-(but-3-en-1-yl)benzoate (**2s**), [1b] (4-(but-3-en-1-yl)phenyl)methanol (**2v**), [1b] (2-methylpent-4-en-2-yl)benzene (**2w**), [1c] *N*-allyl-*N*-ethylaniline (**4h**) [1d] were synthesized according to the corresponding literature.

Because of the high toxicity of carbon monoxide, all of the reactions should be performed in an autoclave. The laboratory should be well-equipped with a CO detector and alarm system.

2. Optimization studies

Table S1 Optimization of the reaction conditions

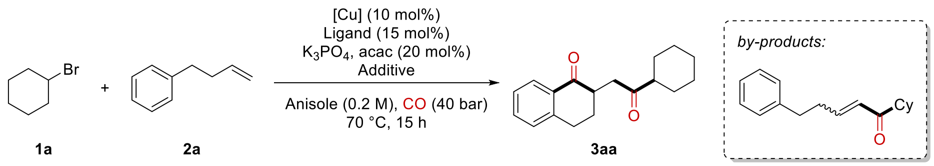

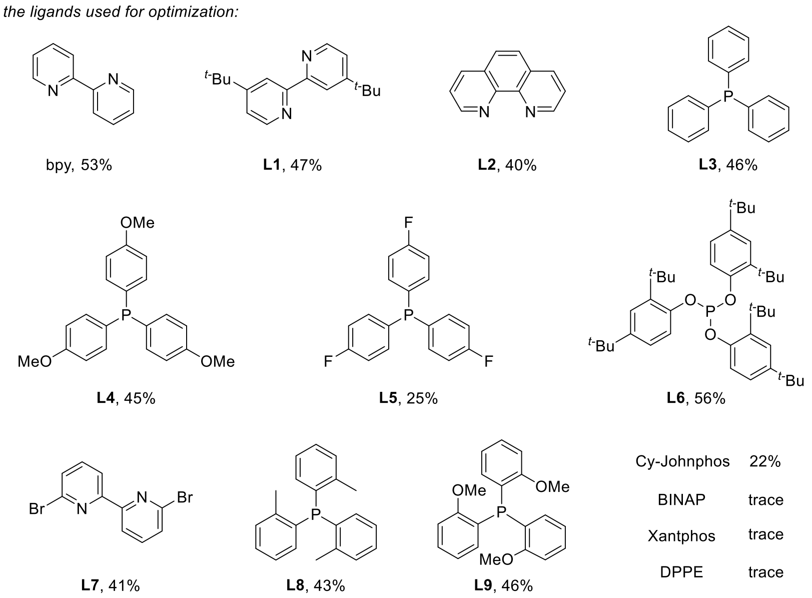

Entry	1a (mmol)	2a (mmol)	[Cu] (10 mol%)	L (mol%)	Additive (mol%)	Base (equiv)	Solvent (mL)	CO (bar)	3aa (%)
1	0.2	0.1	CuBr(Me$_2$S)	bpy	TMEDA (10)	K_3PO_4 (2.0)	Anisole	40	44
2	0.1	0.2	CuBr(Me$_2$S)	bpy	TMEDA (10)	K_3PO_4 (2.0)	Anisole	40	18
3[a]	0.2	0.1	CuBr(Me$_2$S)	bpy	TMEDA (10)	K_3PO_4 (2.0)	Anisole	40	29
4[b]	0.2	0.1	CuBr(Me$_2$S)	bpy	TMEDA (10)	K_3PO_4 (2.0)	Anisole	40	47
5	0.2	0.1	CuBr(Me$_2$S)	bpy	TMEDA (10)	K_3PO_4 (3.0)	Anisole	40	46
6	0.2	0.1	CuBr(Me$_2$S)	bpy	TMEDA (10)	K_3PO_4 (4.0)	Anisole	40	48
7	0.2	0.1	CuBr(Me$_2$S)	bpy	TMEDA (10)	Cs_2CO_3 (3.0)	Anisole	40	8
8	0.2	0.1	CuBr(Me$_2$S)	bpy	TMEDA (10)	K_2CO_3 (3.0)	Anisole	40	trace
9	0.2	0.1	CuBr(Me$_2$S)	bpy	TMEDA (10)	^{t}BuOK (3.0)	Anisole	40	trace
10	0.2	0.1	Cu(acac)$_2$	bpy	TMEDA (10)	K_3PO_4 (3.0)	Anisole	40	9
11	0.2	0.1	$CuCl_2$	bpy	TMEDA (10)	K_3PO_4 (3.0)	Anisole	40	6
12	0.2	0.1	CuI	bpy	TMEDA (10)	K_3PO_4 (3.0)	Anisole	40	7
13	0.2	0.1	Cu(OAc)$_2$	bpy	TMEDA (10)	K_3PO_4 (3.0)	Anisole	40	22
14	0.2	0.1	CuBr(Me$_2$S)	bpy	\	K_3PO_4 (3.0)	Anisole	40	9
15	0.2	0.1	CuBr(Me$_2$S)	bpy	TMEDA (20)	K_3PO_4 (3.0)	Anisole	40	51
16	0.2	0.1	CuBr(Me$_2$S)	bpy	DABCO (20)	K_3PO_4 (3.0)	Anisole	40	53
17	0.2	0.1	CuBr(Me$_2$S)	**L1**	DABCO (20)	K_3PO_4 (3.0)	Anisole	40	47
18	0.2	0.1	CuBr(Me$_2$S)	**L2**	DABCO (20)	K_3PO_4 (3.0)	Anisole	40	40
19	0.2	0.1	CuBr(Me$_2$S)	**L3**	DABCO (20)	K_3PO_4 (3.0)	Anisole	40	446
20	0.2	0.1	CuBr(Me$_2$S)	**L4**	DABCO (20)	K_3PO_4 (3.0)	Anisole	40	45

21	0.2	0.1	CuBr(Me_2S)	**L5**	DABCO (20)	K_3PO_4 (3.0)	Anisole	40	25
22	0.2	0.1	CuBr(Me_2S)	**L6**	DABCO (20)	K_3PO_4 (3.0)	Anisole	40	56(55)[c]
23	0.2	0.1	CuBr(Me_2S)	**L7**	DABCO (20)	K_3PO_4 (3.0)	Anisole	40	41
24	0.2	0.1	CuBr(Me_2S)	**L8**	DABCO (20)	K_3PO_4 (3.0)	Anisole	40	43
25	0.2	0.1	CuBr(Me_2S)	**L9**	DABCO (20)	K_3PO_4 (3.0)	Anisole	40	46
26	0.2	0.1	CuBr(Me_2S)	Cy-Johnphos	DABCO (20)	K_3PO_4 (3.0)	Anisole	40	22
27	0.2	0.1	CuBr(Me_2S)	BINAP	DABCO (20)	K_3PO_4 (3.0)	Anisole	40	trace
28	0.2	0.1	CuBr(Me_2S)	Xantphos	DABCO (20)	K_3PO_4 (3.0)	Anisole	40	trace
29	0.2	0.1	CuBr(Me_2S)	DPPE	DABCO (20)	K_3PO_4 (3.0)	Anisole	40	trace
30	0.2	0.1	CuBr(Me_2S)	**L6**	DABCO (20)	K_3PO_4 (3.0)	1,4-Dioxane	40	39
31	0.2	0.1	CuBr(Me_2S)	**L6**	DABCO (20)	K_3PO_4 (3.0)	PhMe	40	17
32	0.2	0.1	CuBr(Me_2S)	**L6**	DABCO (20)	K_3PO_4 (3.0)	THF	40	25
33	0.2	0.1	CuBr(Me_2S)	**L6**	DABCO (20)	K_3PO_4 (3.0)	MTBE	40	20
34	0.2	0.1	CuBr(Me_2S)	**L6**	DABCO (20)	K_3PO_4 (3.0)	DMAc	40	trace
35[d]	0.2	0.1	CuBr(Me_2S)	**L6**	DABCO (20)	K_3PO_4 (3.0)	Anisole	40	36
36[e]	0.2	0.1	CuBr(Me_2S)	**L6**	DABCO (20)	K_3PO_4 (3.0)	Anisole	40	32
37	0.4	0.2	CuBr(Me_2S)	**L6**	DABCO (20)	K_3PO_4 (3.0)	Anisole (1.0)	40	46
38	0.4	0.2	CuBr(Me_2S)	**L6**	DABCO (20)	K_3PO_4 (3.0)	Anisole (1.5)	40	51
39[f]	0.4	0.2	CuBr(Me_2S)	**L6**	DABCO (20)	K_3PO_4 (3.0)	Anisole (1.5)	40	55
40	0.5	0.2	CuBr(Me_2S)	**L6**	DABCO (20)	K_3PO_4 (3.0)	Anisole (1.5)	40	54
41	0.4	0.2	CuBr(Me_2S)	**L6**	DABCO (20)	K_3PO_4 (3.0)	Anisole (1.5)	50	60(57)[c]
42	0.4	0.2	\	**L6**	DABCO (20)	K_3PO_4 (3.0)	Anisole (1.5)	50	n.d.
42	0.4	0.2	CuBr(Me_2S)	\	DABCO (20)	K_3PO_4 (3.0)	Anisole (1.5)	50	53
43	0.4	0.2	CuBr(Me_2S)	**L6**	\	K_3PO_4 (3.0)	Anisole (1.5)	50	40
44[a,f]	0.4	0.2	CuBr(Me_2S)	**L6**	DABCO (20)	K_3PO_4 (3.0)	Anisole (1.5)	50	55
44[a,f]	0.4	0.2	CuBr(Me_2S)	**L6**	\	K_3PO_4 (3.0)	Anisole (1.5)	50	7
44[a,f]	0.4	0.2	CuBr(Me_2S)	\	DABCO (20)	K_3PO_4 (3.0)	Anisole (1.5)	50	50
44[f]	0.4	0.2	CuBr(Me_2S)	\	\	K_3PO_4 (3.0)	Anisole (1.5)	50	20

Reaction conditions: **1a** (0.2 mmol, 2.0 equiv), **2a** (0.1 mmol), [Cu] (10 mol%), additive (10 or 20 mol%), **L** (15 mol% for bidentate ligands and 30 mol% for monodentate ligands), acac (20 mol%), Base, Solvent, CO, 70 °C, 15 h. Determined by GC using hexadecane as the internal standard. [a] No acac was used; [b] 30 mol% acac was used; [c] Isolated yields; [d] 80 °C; [e] 60 °C; [f] 24 h. acac = Acetylacetone. TMEDA = *N*, *N*, *N'*, *N'*-Tetramethylethylenediamine. DABCO = Triethylenediamine.

bad examples:

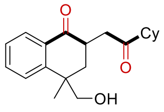

41%
(cannot be purified)

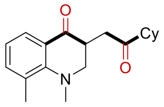

29%

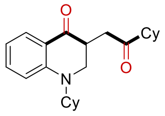

18%

failed examples:

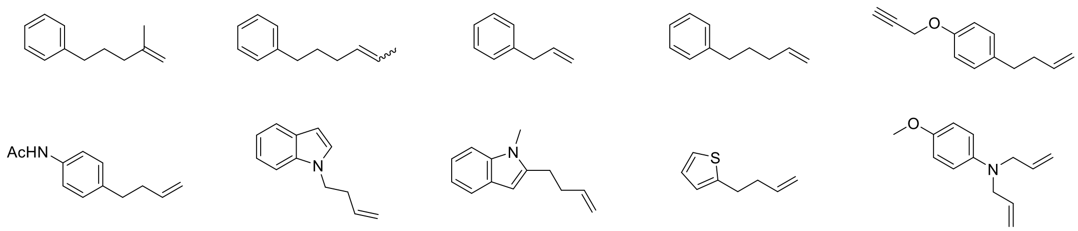

3. General procedure for the synthesis of substrates

3.1 General procedure for the synthesis of alkyl bromides

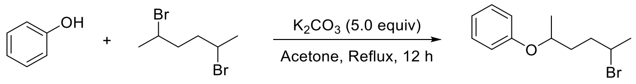

To a solution of phenol (3 mmol) in acetone (15 mL) was added K_2CO_3 (2.07 g, 5.0 equiv) and 2,5-dibromohexane (2.3 mL, 5.0 equiv) at room temperature. The reaction mixture was refluxed for 12 h. After the reaction was completed, the mixture was quenched with H_2O and extracted with CH_2Cl_2. The combined organic layer was dried over Na_2SO_4, filtered and concentrated under reduced pressure. The crude product was further purified by flash column chromatograph (pentane/EA = 50:1, R_f = 0.6) to give ((5-bromohexan-2-yl)oxy)benzene **1j** as a colorless oil (462 mg, 60%).

((5-Bromohexan-2-yl)oxy)benzene (1j)

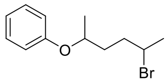

^{1}H NMR (300 MHz, $CDCl_3$) δ 7.35 – 7.26 (m, 2H), 6.99 – 6.90 (m, 3H), 4.49 – 4.35 (m, 1H), 4.25 – 4.12 (m, 1H), 2.11 – 1.79 (m, 4H), 1.75 (d, J = 6.7 Hz, 3H), 1.35 (d, J = 6.1 Hz, 3H). **^{13}C NMR** (75 MHz, $CDCl_3$) δ 157.9, 129.4, 120.6, 115.8, 73.3, 51.7, 37.3, 35.0, 26.6, 19.7.

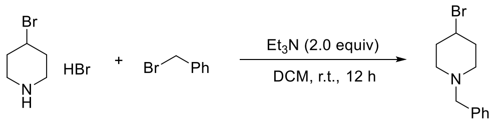

To a solution of 4-bromopiperidine hydrobromide (1.2 g, 5 mmol) in DCM (20 mL) was added Et_3N (1.4 mL, 2.0 equiv) and benzyl bromide (890 µL, 1.5 equiv). The reaction mixture was stirred for 12 h at room temperature. After the reaction was completed, the mixture was quenched with H_2O and extracted with CH_2Cl_2. The combined organic layer was dried over Na_2SO_4, filtered and concentrated under reduced pressure. The crude product was further purified by flash column chromatograph (pentane/EA = 10:1, R_f = 0.4) to give 1-benzyl-4-bromopiperidine **1o** as a colorless oil (1.0 g, 80%). Spectroscopic data in agreement with that reported previously.[2]

3.2 General procedure for the synthesis of alkenes

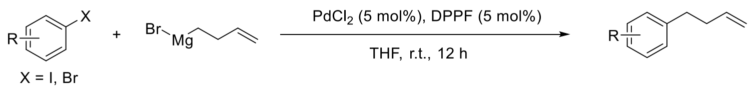

The material was prepared according to the reported literature.[3, 4] To an oven dried 100 mL schlenk tube equipped with a magnetic stir bar, activated magnesium powder (1.3 g, 54 mmol, 1.2 equiv) was added. The equipment was

sealed with rubber septum, evacuated, and backfilled with nitrogen and this operation was repeated three times. Then I_2 and dry THF (20 mL) were added to the tube. The corresponding 4-bromo-1-butene (4.6 mL, 45 mmol) in dry THF (25 mL) was added dropwise by syringe at room temperature. The reaction mixture was stirred at 45 °C for 2 - 4 h. Then, the reaction mixture was cooled to room temperature and used directly in the next step.

The solvent of but-3-en-1-yl magnesium bromide in THF (6.0 mL, 6.0 mmol, 1M) was added dropwise to a solvent of aryl iodide or bromide (2 mmol), $PdCl_2$ (17.7 mg, 5 mol%) and DPPF (55.4 mg, 5 mol%) in dry THF (10 mL) under nitrogen. Then the reaction mixture was stirred at room temperature for 12 h and quenched by a cold solution of NH_4Cl (sat. aq.). The mixture was extracted with CH_2Cl_2. The combined organic layer was dried over Na_2SO_4, filtered and concentrated under reduced pressure. The crude residue was purified by flash chromatography using pentane to afford the corresponding products.

1-(But-3-en-1-yl)-2-methylbenzene (2b)

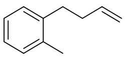

The title compound was prepared from 2-iodotoluene (254 µL, 2 mmol) and but-3-en-1-yl magnesium bromide (6.0 mL, 6.0 mmol, 1M in THF) as a colorless oil (207 mg, 71%, pentane, R_f = 0.8). **^{1}H NMR** (300 MHz, $CDCl_3$) δ 7.20 – 7.11 (m, 4H), 6.03 – 5.84 (m, 1H), 5.15 – 4.99 (m, 2H), 2.77 – 2.69 (m, 2H), 2.41 – 2.32 (m, 2H), 2.34 (s, 3H). **^{13}C NMR** (75 MHz, $CDCl_3$) δ 140.0, 138.3, 135.9, 130.1, 128.7, 125.9, 125.9, 114.8, 34.3, 32.7, 19.3.

1-(But-3-en-1-yl)-3-methylbenzene (2c)

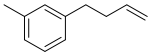

The title compound was prepared from 3-iodotoluene (257 µL, 2 mmol) and but-3-en-1-yl magnesium bromide (6.0 mL, 6.0 mmol, 1M in THF) as a colorless oil (234 mg, 80%, pentane, R_f = 0.8). **^{1}H NMR** (300 MHz, $CDCl_3$) δ 7.22 – 7.15 (m, 1H), 7.05 – 6.97 (m, 3H), 5.96 – 5.81 (m, 1H), 5.11 – 4.97 (m, 2H), 2.69 (dd, *J* = 9.2, 6.6 Hz, 2H), 2.43 – 2.36 (m, 2H), 2.35 (s, 3H). **^{13}C NMR** (75 MHz, $CDCl_3$) δ 141.8, 138.2, 137.8, 129.2, 128.2, 126.5, 125.4, 114.8, 35.5, 35.3, 21.4.

1-(But-3-en-1-yl)-4-methylbenzene (2d)

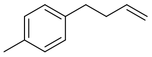

The title compound was prepared from 4-iodotoluene (259 µL, 2 mmol) and but-3-en-1-yl magnesium bromide (6.0 mL, 6.0 mmol, 1M in THF) as a colorless oil (220 mg, 75%, pentane, R_f = 0.8).
Spectroscopic data in agreement with that reported previously.[5]

1-(But-3-en-1-yl)-3,5-dimethylbenzene (2e)

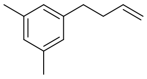

The title compound was prepared from 1-iodo-3,5-dimethylbenzene (288 µL, 2 mmol) and but-3-en-1-yl magnesium bromide (6.0 mL, 6.0 mmol, 1M in THF) as a colorless oil (262 mg, 82%, pentane, R_f = 0.8). **^{1}H NMR** (300 MHz, $CDCl_3$) δ 6.89 – 6.82 (m, 3H), 5.98 – 5.82 (m, 1H), 5.14 – 4.97 (m, 2H), 2.71 – 2.63 (m, 2H), 2.43 – 2.35 (m, 2H), 2.33 (s, 6H). **^{13}C NMR** (75 MHz, $CDCl_3$) δ 141.8, 138.3, 137.7, 127.4, 126.2, 114.7, 35.6, 35.3, 21.3.

1-(But-3-en-1-yl)-4-(*tert*-butyl)benzene (2f)

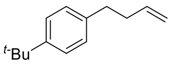

The title compound was prepared from 1-iodo-4-*tert*-butylbenzene (520 mg, 2 mmol) and but-3-en-1-yl magnesium bromide (6.0 mL, 6.0 mmol, 1M in THF) as a colorless oil (320 mg, 85%, pentane, R_f = 0.8). **^{1}H NMR** (300 MHz, $CDCl_3$) δ 7.38 – 7.31 (m, 2H), 7.20 – 7.14 (m, 2H), 6.03 – 5.83 (m, 1H), 5.16 – 4.97 (m, 2H), 2.79 – 2.68 (m, 2H), 2.49 – 2.34 (m, 2H), 1.35 (s, 9H). **^{13}C NMR** (75 MHz, $CDCl_3$) δ 148.6, 138.8, 138.3, 128.0, 125.2, 114.7, 35.5, 34.8, 34.3, 31.4.

1-(But-3-en-1-yl)-4-methoxybenzene (2g)

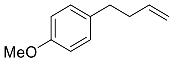

The title compound was prepared from 4-iodoanisole (5.6 g, 25 mmol) and but-3-en-1-yl magnesium bromide (50 mL, 50 mmol, 1M in THF) as a colorless oil (3.4 g, 84%, pentane, R_f = 0.5). **^{1}H NMR** (300 MHz, $CDCl_3$) δ 7.15 – 7.08 (m, 2H), 6.88 – 6.81 (m, 2H), 5.95 – 5.79 (m, 1H), 5.11 – 4.95 (m, 2H), 3.80 (s, 3H), 2.71 – 2.63 (m, 2H), 2.41 – 2.31 (m, 2H). **^{13}C NMR** (75 MHz, $CDCl_3$) δ 157.7, 138.2, 133.9, 129.3, 114.8, 113.7, 55.2, 35.8, 34.5.

1-(But-3-en-1-yl)-4-(trifluoromethoxy)benzene (2h)

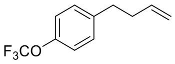

The title compound was prepared from 1-iodo-4-(trifluoromethoxy)benzene (313 µL, 2 mmol) and but-3-en-1-yl magnesium bromide (6.0 mL, 6.0 mmol, 1M in THF) as a colorless oil (220 mg, 51%, pentane, R_f = 0.8).

Spectroscopic data in agreement with that reported previously.[6]

1-(4-(But-3-en-1-yl)phenyl)-1*H*-pyrrole (2m)

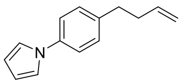

The title compound was prepared from 1-(4-iodophenyl)pyrrole (538 mg, 2 mmol) and but-3-en-1-yl magnesium bromide (6.0 mL, 6.0 mmol, 1M in THF) as a yellow oil (244 mg, 62%, pentane, R_f = 0.7). **^{1}H NMR** (300 MHz, $CDCl_3$) δ 7.20 – 7.06 (m, 4H), 6.93 (t, *J* = 2.2 Hz, 2H), 6.20 (t, *J* = 2.2 Hz, 2H), 5.82 – 5.65 (m, 1H), 4.98 – 4.81 (m, 2H), 2.68 – 2.50 (m, 2H), 2.34 – 2.18 (m, 2H). **^{13}C NMR** (75 MHz, $CDCl_3$) δ 139.3, 138.8, 137.8, 129.4, 120.5, 119.3, 115.2, 110.1, 35.4, 34.7.

1-(But-3-en-1-yl)-4-fluorobenzene (2p)

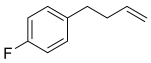

The title compound was prepared from 1-fluoro-4-iodobenzene (230 µL, 2 mmol) and but-3-en-1-yl magnesium bromide (6.0 mL, 6.0 mmol, 1M in THF) as a colorless oil (226 mg, 75%, pentane, R_f = 0.8).

Spectroscopic data in agreement with that reported previously. [5]

1-(But-3-en-1-yl)-4-chlorobenzene (2q)

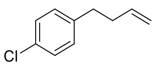

The title compound was prepared from 4-bromochlorobenzene (382 mg, 2 mmol) and but-3-en-1-yl magnesium bromide (6.0 mL, 6.0 mmol, 1M in THF) as a colorless oil (200 mg, 60%, pentane, R_f = 0.8). Spectroscopic data in agreement with that reported previously. [5]

1-(But-3-en-1-yl)-4-(trifluoromethyl)benzene (2t)

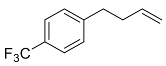

The title compound was prepared from 4-bromobenzotrifluoride (280 µL, 2 mmol) and but-3-en-1-yl magnesium bromide (6.0 mL, 6.0 mmol, 1M in THF) as a colorless oil (189 mg, 47%, pentane, R_f = 0.8). Spectroscopic data in agreement with that reported previously.[6]

1-(But-3-en-1-yl)naphthalene (2x)

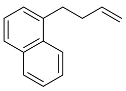

The title compound was prepared from 1-iodonaphthalene (292 µL, 2 mmol) and but-3-en-1-yl magnesium bromide (6.0 mL, 6.0 mmol, 1M in THF) as a colorless oil (333 mg, 91%, pentane, R_f = 0.8). **^{1}H NMR** (300 MHz, $CDCl_3$) δ 8.11 – 8.02 (m, 1H), 7.92 – 7.84 (m, 1H), 7.73 (d, *J* = 8.0 Hz, 1H), 7.56 – 7.33 (m, 4H), 6.06 – 5.89 (m, 1H), 5.18 – 4.99 (m, 2H), 3.26 – 3.13 (m, 2H), 2.60 – 2.47 (m, 2H). **^{13}C NMR** (75 MHz, $CDCl_3$) δ 138.2, 137.9, 133.9, 131.8, 128.8, 126.6, 125.9, 125.7, 125.5, 125.4, 123.7, 114.9, 34.8, 32.5.
Spectroscopic data in agreement with that reported previously.[7]

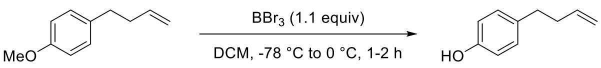

The material was prepared according to the reported literature.[6] The solvent of 1-(but-3-en-1-yl)-4-methoxybenzene (2.4 g, 15 mmol) in CH_2Cl_2 (30 mL) was cooled to -78 °C, then BBr_3 (16.5 mL, 1M in CH_2Cl_2, 16.5 mmol, 1.1 equiv) was added dropwise to the solvent. The reaction mixture was stirred at -78 °C for 1 h and then stirred at 0 °C for another 1 h. The reaction was detected by TLC, if it was incomplete, additional BBr_3 (4.5 mL, 1M in CH_2Cl_2, 4.5 mmol, 0.3 equiv) was added dropwise to the solvent at 0 °C and stirred for 1 h. The mixture was quenched with H_2O and extracted with CH_2Cl_2. The combined organic layer was dried over Na_2SO_4, filtered and concentrated under reduced pressure. The crude product was further purified by flash column chromatograph (pentane/EA = 5:1, R_f = 0.4) to give 4-(but-3-en-1-yl)phenol **2u** as a colorless oil (1.7 g, 77%). Spectroscopic data in agreement with that reported previously. [8]

Note: In this reaction, the products will react with excess bromine to form brominated by-products that were difficult to separate. To avoid this, the temperature and the amount of BBr_3 need to be controlled and, if necessary, the by-product formation can be reduced by decreasing the conversion of the reaction.

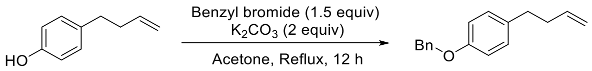

To a solution of the 4-(but-3-en-1-yl)phenol (148 mg, 1 mmol) in acetone (5 mL) was added K_2CO_3 (276 mg, 2 equiv) and benzyl bromide (178 µL, 1.5 equiv). The reaction mixture was refluxed for 12 h. After the reaction was completed, the mixture was quenched with H_2O and extracted with CH_2Cl_2. The combined organic layer was dried over Na_2SO_4, filtered and concentrated under reduced pressure. The crude product was further purified by flash column chromatograph (pentane/EA = 20:1, R_f = 0.3) to give 1-(benzyloxy)-4-(but-3-en-1-yl)benzene **2i** as a colorless oil (128 mg, 54%). Spectroscopic data in agreement with that reported previously.[9]

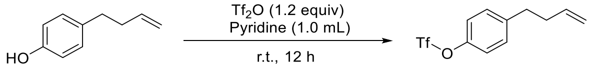

To a solution of 4-(but-3-en-1-yl)phenol (148 mg, 1 mmol) in pyridine (1 mL) was added Tf_2O (201 µL, 1.2 equiv) at 0 °C. The reaction mixture was stirred for 12 h at room temperature. After the reaction was completed, Et_2O was added to the reaction and the mixture was washed with HCl aqueous solution. The organic layer was dried over Na_2SO_4, filtered and concentrated under reduced pressure. The crude product was further purified by flash column chromatograph (pentane/EA = 10:1, R_f = 0.4) to give 4-(but-3-en-1-yl)phenyl trifluoromethanesulfonate **2j** as a colorless oil (156 mg, 56%).

4-(But-3-en-1-yl)phenyl trifluoromethanesulfonate (2j)

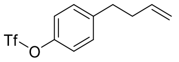

^{1}H NMR (400 MHz, $CDCl_3$) δ 7.27 – 7.22 (m, 2H), 7.19 – 7.15 (m, 2H), 5.82 (ddt, J = 16.9, 10.2, 6.6 Hz, 1H), 5.07 – 4.96 (m, 2H), 2.76 – 2.69 (m, 2H), 2.41 – 2.32 (m, 2H). **^{13}C NMR** (100 MHz, $CDCl_3$) 147.8, 142.4, 137.2, 130.1, 121.1, 118.74 (d, J = 320.7 Hz), 115.5, 35.2, 34.6.

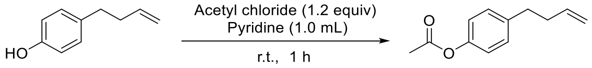

To a solution of the 4-(but-3-en-1-yl)phenol (148 mg, 1 mmol) in pyridine (1 mL) was added AcCl (85.3 µL, 1.2 equiv) at 0 °C. The reaction mixture was stirred for 1 h at room temperature. After the reaction was completed, Et_2O was added to the reaction and the mixture was washed with HCl aqueous solution. The organic layer was dried over Na_2SO_4, filtered and concentrated under reduced pressure. The crude product was further purified by flash column chromatograph (pentane/EA = 2:1, R_f = 0.3) to give 4-(but-3-en-1-yl)phenyl acetate **2k** as a colorless oil (185 mg, 97%).

4-(But-3-en-1-yl)phenyl acetate (2k)

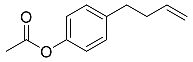

^{1}H NMR (300 MHz, $CDCl_3$) δ 7.21 – 7.16 (m, 2H), 7.02 – 6.97 (m, 2H), 5.86 (ddt, *J* = 16.9, 10.2, 6.6 Hz, 1H), 5.09 – 4.95 (m, 2H), 2.70 (dd, *J* = 9.0, 6.6 Hz, 2H), 2.41 – 2.32 (m, 2H), 2.29 (s, 3H). **^{13}C NMR** (75 MHz, $CDCl_3$) 169.6, 148.7, 139.4, 137.8, 129.3, 121.2, 115.0, 35.4, 34.7, 21.1.

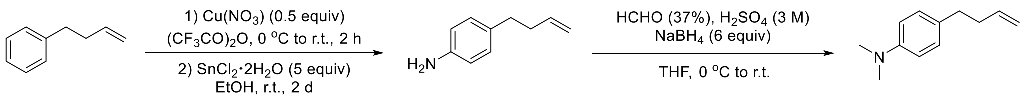

4-(but-3-en-1-yl)aniline was prepared according to the reported literature.[10]

A mixture of 3 M sulfuric acid (2 mL, 6 mmol) and aqueous formaldehyde solution (37%, 2 mL) was cooled to 0 ºC. A slurry of 4-(but-3-en-1-yl)aniline (294 mg, 2 mmol) and finely grinded sodium borohydride (456 mg, 12 mmol) in THF (10 mL) was added dropwise to keep the reaction temperature below 20 ºC. After complete addition, the reaction mixture was adjusted to neutral (pH ≈ 7) with K_2CO_3 and extracted with EtOAc (3 × 50 mL). The combined organic layers were washed with brine (50 mL), dried over Na_2SO_4, filtered and concentrated under reduced pressure. The crude product was further purified by flash column chromatograph (pentane/EA = 20:1, Rf = 0.4) to give 4-(but-3-en-1-yl)-*N*, *N*-dimethylaniline **2l** as a colorless oil (280 mg, 80%). Spectroscopic data in agreement with that reported previously.[11]

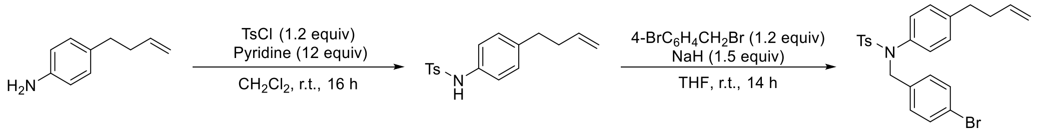

To a solution of the 4-(but-3-en-1-yl)aniline (294 mg, 2 mmol) in CH_2Cl_2 (10 mL) was added pyridine (1.9 mL, 12 equiv) and TsCl (457 mg, 1.2 equiv). The reaction mixture was stirred for 16 h at room temperature. After the reaction was completed, the mixture was washed with HCl. The organic layer was dried over Na_2SO_4, filtered and concentrated under reduced pressure. *N*-(4-(but-3-en-1-yl)phenyl)-4-methylbenzenesulfonamide was further purified by flash column chromatograph (pentane/EA = 10:1, R_f = 0.3) to give the product as a colorless oil (568mg, 94%).

To a solution of *N*-(4-(but-3-en-1-yl)phenyl)-4-methylbenzenesulfonamide (301 mg, 1 mmol) in THF (10 mL) was added NaH (60 mg, 1.5 mmol, 1.5 equiv) at room temperature. After stirring for 10 min, 1-bromo-4-(bromomethyl)benzene (298.8 mg, 1.2 mmol, 1.2 equiv) was added and the reaction mixture was stirred for 14 h at room temperature. After the reaction was completed, the mixture was quenched with H_2O and extracted with CH_2Cl_2. The combined organic layer was dried over Na_2SO_4, filtered and concentrated under reduced pressure. The crude

product was further purified by flash column chromatograph (pentane/EA = 10:1, R_f = 0.5) to give *N*-(4-bromobenzyl)-*N*-(4-(but-3-en-1-yl)phenyl)-4-methylbenzenesulfonamide **2n** as a white solid (235 mg, 50%).

***N*-(4-Bromobenzyl)-*N*-(4-(but-3-en-1-yl)phenyl)-4-methylbenzenesulfonamide (2n)**

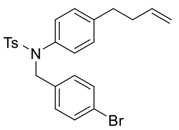

^{1}H NMR (300 MHz, $CDCl_3$) δ 7.45 (d, *J* = 8.1 Hz, 2H), 7.27 (d, *J* = 8.4 Hz, 2H), 7.20 (d, *J* = 8.1 Hz, 2H), 7.03 (d, *J* = 8.4 Hz, 2H), 6.95 (d, *J* = 8.4 Hz, 2H), 6.79 (d, *J* = 8.4 Hz, 2H), 5.80 – 5.64 (m, 1H), 4.98 – 4.83 (m, 2H), 4.57 (s, 2H), 2.62 – 2.50 (m, 2H), 2.37 (s, 3H), 2.30 – 2.17 (m, 2H). **^{13}C NMR** (75 MHz, $CDCl_3$) δ 143.5, 141.7, 137.6, 136.4, 135.5, 135.2, 131.4, 130.2, 129.5, 128.9, 128.6, 127.7, 121.5, 115.1, 54.1, 35.1, 34.8, 21.5.

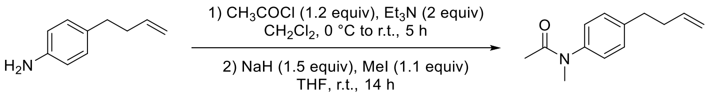

To a solution of the 4-(but-3-en-1-yl)aniline (147 mg, 1 mmol) in CH_2Cl_2 (5 mL) was added Et_3N (278 μL, 2.0 equiv) and AcCl (85.3 μL, 1.2 equiv) at 0 °C. The reaction mixture was stirred for 5 h at room temperature. After the reaction was completed, the mixture was quenched with H_2O and extracted with CH_2Cl_2. The combined organic layer was dried over Na_2SO_4, filtered and concentrated under reduced pressure. *N*-(4-(but-3-en-1-yl)phenyl)acetamide was further purified by flash column chromatograph (pentane/EA = 2:1, R_f = 0.2) to give the product as a white solid (180 mg, 95%).

To a solution of *N*-(4-(but-3-en-1-yl)phenyl)acetamide (180 mg, 0.95 mmol) in THF (10 mL) was added NaH (60 mg, 1.5 mmol, 1.5 equiv) at room temperature. After stirring for 10 min, MeI (69 μL, 1.1 mmol, 1.1 equiv) was added and the reaction mixture was stirred for 14 h at room temperature. After the reaction was completed, the mixture was quenched with H_2O and extracted with CH_2Cl_2. The combined organic layer was dried over Na_2SO_4, filtered and concentrated under reduced pressure. The crude product was further purified by flash column chromatograph (pentane/EA = 5:1, R_f = 0.2) to give *N*-(4-(But-3-en-1-yl)phenyl)-*N*-methylacetamide **2o** as a colorless oil (174 mg, 90%).

***N*-(4-(But-3-en-1-yl)phenyl)-*N*-methylacetamide (2o)**

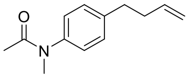

^{1}H NMR (300 MHz, $CDCl_3$) δ 7.25 – 7.18 (m, 2H), 7.08 (d, *J* = 8.3 Hz, 2H), 5.93 – 5.75 (m, 1H), 5.09 – 4.96 (m, 2H), 3.24 (s, 3H), 2.72 (dd, *J* = 8.9, 6.6 Hz, 2H), 2.44 – 2.33 (m, 2H), 1.85 (s, 3H). **^{13}C NMR** (75 MHz, $CDCl_3$) δ 170.7, 142.3, 141.5, 137.6, 129.6, 126.8, 115.2, 37.1, 35.2, 34.8, 22.3.

3.3 General procedure for the synthesis of *N*-alkyl-*N*-allylanilines

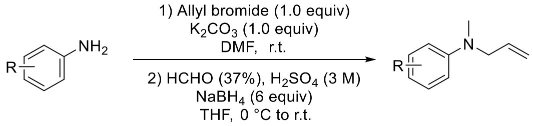

To a solution of aniline (5 mmol) in DMF (15 mL) was added K_2CO_3 (690 mg, 1.0 equiv) and allyl bromide (432 µL, 1.0 equiv) at room temperature. The reaction mixture was stirred for 12 h at room temperature. After the reaction was completed, the mixture was quenched with H_2O and extracted with CH_2Cl_2. The combined organic layer was washed with H_2O and dried over Na_2SO_4, filtered and concentrated under reduced pressure. The crude product was further purified by flash column chromatograph to give the corresponding *N*-allylaniline and used in the next step.

A mixture of 3 M sulfuric acid (2 mL, 6 mmol) and aqueous formaldehyde solution (37%, 2 mL) was cooled to 0 ºC. A slurry of *N*-allylaniline (2 mmol) and finely grinded sodium borohydride (456 mg, 12 mmol) in THF (10 mL) was added dropwise to keep the reaction temperature below 20 ºC. After complete addition, the reaction mixture was adjusted to neutral (pH ≈ 7) with K_2CO_3 and extracted with EtOAc (3 × 50 mL). The combined organic layers were washed with brine (50 mL), dried over Na_2SO_4, filtered and concentrated under reduced pressure. The crude product was further purified by flash column chromatograph to give the corresponding product.

Note: A certain amount of diallyl substitution was formed in the first step and almost completely converted in the second step.

N-Allyl-N,4-dimethylaniline (4i)

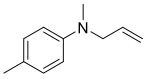

322 mg, 40% yield, colorless oil, pentane/EA = 50:1, R_f = 0.7. Spectroscopic data in agreement with that reported previously.[12]

***N*-Allyl-4-(*tert*-butyl)-*N*-methylaniline (4j)**

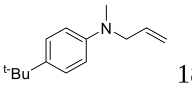

183 mg, 18% yield, light yellow oil, pentane/EA = 50:1, R_f = 0.8. **^{1}H NMR** (300 MHz, $CDCl_3$) δ 7.36 – 7.27 (m, 2H), 6.80 – 6.70 (m, 2H), 5.99 – 5.83 (m, 1H), 5.29 – 5.15 (m, 2H), 3.96 – 3.91 (m, 2H), 2.96 (s, 3H), 1.34 (s, 9H). **^{13}C NMR** (75 MHz, $CDCl_3$) δ 147.3, 139.1, 134.2, 125.8, 116.1, 112.3, 55.6, 38.0, 33.7, 31.5.

***N*-Allyl-4-methoxy-*N*-methylaniline (4k)**

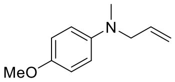

277 mg, 31% yield, colorless oil, pentane/EA = 50:1, R_f = 0.5. **^{1}H NMR** (300 MHz, $CDCl_3$) δ 6.90 – 6.83 (m, 2H), 6.79 – 6.73 (m, 2H), 5.88 (ddt, *J* = 17.2, 10.2, 5.4 Hz, 1H), 5.25 – 5.15 (m, 2H), 3.86 (dt, *J* = 5.4, 1.6 Hz, 2H), 3.78 (s, 3H), 2.89 (s, 3H). **^{13}C NMR** (75 MHz, $CDCl_3$) δ 151.7, 144.4, 134.2, 116.4, 114.7, 114.6, 56.5, 55.6, 38.6.

N-Allyl-4-fluoro-N-methylaniline (4l)

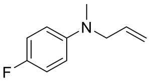
313 mg, 38% yield, light yellow oil, pentane/EA = 50:1, R_f = 0.7. **^{1}H NMR** (300 MHz, $CDCl_3$) δ 6.98 – 6.90 (m, 2H), 6.71 – 6.64 (m, 2H), 5.91 – 5.78 (m, 1H), 5.22 – 5.18 (m, 1H), 5.15 (t, *J* = 1.6 Hz, 1H), 3.88 (dt, *J* = 5.2, 1.7 Hz, 2H), 2.91 (s, 3H). **^{13}C NMR** (75 MHz, $CDCl_3$) δ 155.4 (d, *J* = 235.2 Hz), 146.2, 133.7, 116.4, 115.4 (d, *J* = 22.0 Hz), 113.8 (d, *J* = 7.4 Hz), 56.1, 38.5. **^{19}F NMR** (282 MHz, $CDCl_3$) δ -129.2.

N-Allyl-4-chloro-N-methylaniline (4m)

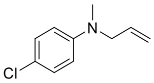
334 mg, 37% yield, light yellow oil, pentane/EA = 50:1, R_f = 0.7. **^{1}H NMR** (300 MHz, $CDCl_3$) δ 7.21 – 7.13 (m, 2H), 6.69 – 6.61 (m, 2H), 5.84 (ddt, *J* = 16.7, 10.7, 5.0 Hz, 1H), 5.19 (td, *J* = 1.7, 1.1 Hz, 1H), 5.15 (dq, *J* = 8.9, 1.7 Hz, 1H), 3.91 (dt, *J* = 5.0, 1.7 Hz, 2H), 2.94 (s, 3H). **^{13}C NMR** (75 MHz, $CDCl_3$) δ 147.9, 133.2, 128.8, 121.1, 116.3, 113.5, 55.2, 38.2.

N-Allyl-4-bromo-N-methylaniline (4n)

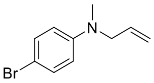
565 mg, 50% yield, light yellow oil, pentane/EA = 50:1, R_f = 0.7. Spectroscopic data in agreement with that reported previously.[13]

N-Allyl-N-methyl-4-(trifluoromethyl)aniline (4o)

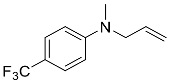
430 mg, 40% yield, light yellow oil, pentane/EA = 50:1, R_f = 0.7. **^{1}H NMR** (300 MHz, $CDCl_3$) δ 7.47 – 7.41 (m, 2H), 6.74 – 6.67 (m, 2H), 5.83 (ddt, *J* = 17.1, 10.4, 4.8 Hz, 1H), 5.22 – 5.09 (m, 2H), 3.97 (dt, *J* = 4.8, 1.8 Hz, 2H), 3.01 (s, 3H). **^{13}C NMR** (75 MHz, $CDCl_3$) δ 151.3, 132.6, 126.4 (q, *J* = 3.8 Hz), 125.1 (d, *J* = 270.0 Hz), 116.4, 111.2, 54.8, 38.1. **^{19}F NMR** (282 MHz, $CDCl_3$) δ -60.9.

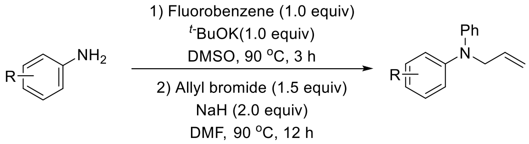

The *N*-arylaniline was prepared according to the reported literature.[14]

To a solution of *N*-arylaniline (3 mmol) in DMF (10 mL) was added NaH (240 mg, 2.0 equiv) at room temperature. After stirring for 30 min, allyl bromide (389 µL, 1.5 equiv) was added at room temperature and the reaction mixture was stirred at 90 °C for 12 h. Then the mixture was quenched with H_2O and extracted with CH_2Cl_2. The combined organic layer was washed with H_2O and dried over Na_2SO_4, filtered and concentrated under reduced pressure. The

crude product was further purified by flash column chromatograph (pentane/EA = 50:1, R_f = 0.5) to give *N*-Allyl-4-methoxy-*N*-phenylaniline **9** as colorless oil (294 mg, 42%).

***N*-Allyl-4-methoxy-*N*-phenylaniline (9)**

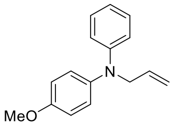

^{1}H NMR (300 MHz, $CDCl_3$) δ 7.22 – 7.11 (m, 4H), 6.93 – 6.88 (m, 2H), 6.82 – 6.75 (m, 3H), 6.04 – 5.89 (m, 1H), 5.32 – 5.16 (m, 2H), 4.31 (dt, *J* = 5.0, 1.7 Hz, 2H), 3.83 (s, 3H). ^{13}C NMR (75 MHz, $CDCl_3$) δ 156.3, 148.7, 140.7, 134.4, 128.9, 126.9, 118.1, 116.3, 115.7, 114.8, 55.4, 55.1.

4. General procedure for the carbonylation of alkyl bromides with alkenes

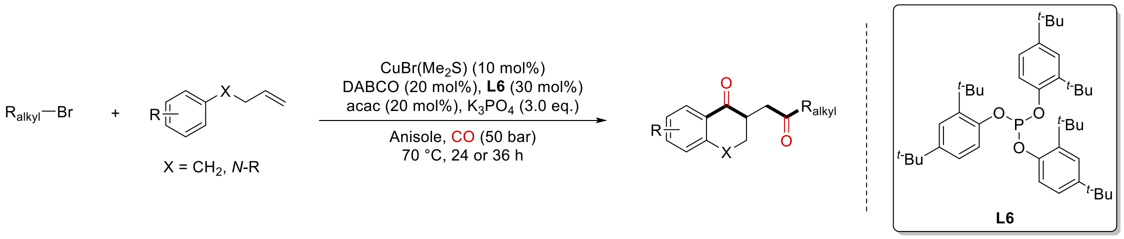

General procedure: A 4 mL screw-cap vial was charged with $CuBr(Me_2S)$ (4.1 mg, 10 mol%), tris(2,4-di-*tert*-butylphenyl) phosphite **L6** (38.8 mg, 30 mol%), DABCO (4.4 mg, 20 mol%), K_3PO_4 (127.4 mg, 0.6 mmol, 3.0 equiv) and an oven-dried stirring bar. The vial was closed by Teflon septum and phenolic cap and connected to atmosphere with a needle. After flashed the vials three times with argon and vacuum, dry anisole (1.5 mL), alkyl bromides (0.4 mmol, 2.0 equiv), alkenes (0.2 mmol, 1.0 equiv) and acac (4.1 µL, 20 mol%) were injected by syringe. The vial was fixed in an alloy plate and put into Parr 4560 series autoclave (500 mL). At room temperature, the autoclave was flushed three times with carbon monoxide and 50 bar of carbon monoxide was charged. The autoclave was reacted at 70 °C for 24 h (for *N*-allylanilines, 36 h was used). Afterwards, the autoclave was cooled to room temperature and the pressure was carefully released. The crude product was purified by silica gel chromatography (pentane/EA) to afford the corresponding products.

5. Characterization and procedure of the products

2-(2-Cyclohexyl-2-oxoethyl)-3,4-dihydronaphthalen-1(2*H*)-one (3aa):

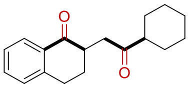

The title compound was prepared from bromocyclohexane (49.2 µL, 0.4 mmol) and but-3-en-1-ylbenzene (30 µL, 0.2 mmol) according to the general procedure, and the crude residue was purified by flash chromatography (pentane/EA = 20:1, R_f = 0.3) to give the product as a colorless oil (31.0 mg, 57%).

^{1}H NMR (300 MHz, $CDCl_3$) δ 7.99 (dd, *J* = 7.9, 1.5 Hz, 1H), 7.45 (td, *J* = 7.5, 1.5 Hz, 1H), 7.31 – 7.21 (m, 2H), 3.29 – 3.02 (m, 3H), 2.99 – 2.89 (m, 1H), 2.52 – 2.41 (m, 2H), 2.21 – 2.10 (m, 1H), 1.99 – 1.86 (m, 3H), 1.83 – 1.75 (m, 2H), 1.71 – 1.64 (m, 1H), 1.41 – 1.22 (m, 5H). **^{13}C NMR** (75 MHz, $CDCl_3$) δ 212.2, 199.0, 144.0, 133.2, 132.2, 128.6, 127.2, 126.4, 51.0, 43.9, 40.9, 29.4, 29.3, 28.5, 28.3, 25.8, 25.7, 25.5. **HRMS** (ESI) calcd for $C_{18}H_{23}O_2^+$ [M+H$^+$]: 271.1693, Found: 271.1695.

2-(2-Cyclohexyl-2-oxoethyl)-5-methyl-3,4-dihydronaphthalen-1(2*H*)-one (3ab):

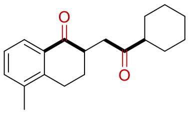

The title compound was prepared from bromocyclohexane (49.2 µL, 0.4 mmol) and 1-(but-3-en-1-yl)-2-methylbenzene (29.2 mg, 0.2 mmol) according to the general procedure, and the crude residue was purified by flash chromatography (pentane/EA = 20:1, R_f = 0.3) to give the product as a colorless oil (29.4 mg, 52%).

^{1}H NMR (300 MHz, $CDCl_3$) δ 7.87 (d, *J* = 7.8 Hz, 1H), 7.34 (d, *J* = 7.4 Hz, 1H), 7.20 (t, *J* = 7.6 Hz, 1H), 3.30 – 3.07 (m, 2H), 3.01 – 2.79 (m, 2H), 2.53 – 2.37 (m, 2H), 2.30 (s, 3H), 2.24 – 2.15 (m, 1H), 1.98 – 1.88 (m, 2H), 1.87 – 1.74 (m, 3H), 1.70 – 1.63 (m, 1H), 1.42 – 1.20 (m, 5H). **^{13}C NMR** (75 MHz, $CDCl_3$) δ 212.5, 199.5, 142.3, 136.3, 134.6, 132.5, 126.1, 125.2, 51.2, 43.2, 40.9, 28.8, 28.6, 28.4, 26.4, 25.9, 25.8, 25.6, 19.3. **HRMS** (ESI) calcd for $C_{19}H_{25}O_2^+$ [M+H$^+$]: 285.1849, Found: 285.1857.

2-(2-Cyclohexyl-2-oxoethyl)-8-methyl-3,4-dihydronaphthalen-1(2*H*)-one and 2-(2-Cyclohexyl-2-oxoethyl)-6-methyl-3,4-dihydronaphthalen-1(2*H*)-one (3ac):

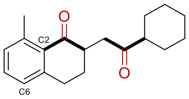

The title compound was prepared from bromocyclohexane (49.2 µL, 0.4 mmol) and 1-(but-3-en-1-yl)-3-methylbenzene (29.2 mg, 0.2 mmol) according to the general procedure, and the crude residue was purified by flash chromatography to give the product as a colorless oil (37.9 mg, 67%).

2-(2-Cyclohexyl-2-oxoethyl)-8-methyl-3,4-dihydronaphthalen-1(2*H*)-one (3ac-I)

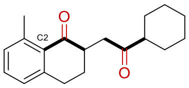

3ac-I: 20.6 mg, 36%, colorless oil, pentane/EA = 20:1, R_f = 0.5. **^{1}H NMR** (300 MHz, $CDCl_3$) δ 7.29 (t, *J* = 7.6 Hz, 1H), 7.07 (d, *J* = 7.6 Hz, 2H), 3.26 – 3.01 (m, 3H), 3.00 – 2.89 (m, 1H), 2.59 (s, 3H), 2.51 – 2.40 (m, 2H), 2.15 – 2.04 (m, 1H), 1.96 – 1.75 (m, 5H), 1.70 – 1.62 (m, 1H), 1.39 – 1.22 (m, 5H). **^{13}C NMR** (75 MHz, $CDCl_3$) δ 212.6, 201.0, 145.0, 141.3, 132.0, 131.1, 130.3, 126.7, 51.1, 45.2, 41.4, 30.3, 29.2, 28.6, 28.4, 25.9, 25.8, 25.6, 23.2. **HRMS** (ESI) calcd for $C_{19}H_{25}O_2^+$ [M+H$^+$]: 285.1849, Found: 285.1859.

2-(2-Cyclohexyl-2-oxoethyl)-6-methyl-3,4-dihydronaphthalen-1(2*H*)-one (3ac-II)

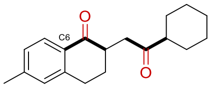

3ac-II: 17.3 mg, 31%, colorless oil, pentane/EA = 20:1, R_f = 0.4. **^{1}H NMR** (300 MHz, $CDCl_3$) δ 7.89 (d, *J* = 8.0 Hz, 1H), 7.13 – 7.06 (m, 1H), 7.03 (s, 1H), 3.28 – 2.99 (m, 3H), 2.94 – 2.83 (m, 1H), 2.53 – 2.39 (m, 2H), 2.36 (s, 3H), 2.19 – 2.08 (m, 1H), 1.98 – 1.76 (m, 5H), 1.70 – 1.62 (m, 1H), 1.40 – 1.22 (m, 5H). **^{13}C NMR** (75 MHz, $CDCl_3$) δ 212.5, 198.9, 144.2, 144.1, 129.9, 129.1, 127.6, 127.5, 51.2, 44.0, 41.1, 29.6, 29.3, 28.6, 28.4, 25.9, 25.8, 25.6, 21.7. **HRMS** (ESI) calcd for $C_{19}H_{25}O_2^+$ [M+H$^+$]: 285.1849, Found: 285.1857.

2-(2-Cyclohexyl-2-oxoethyl)-7-methyl-3,4-dihydronaphthalen-1(2*H*)-one (3ad):

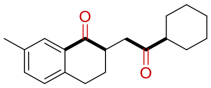

The title compound was prepared from bromocyclohexane (49.2 μL, 0.4 mmol) and 1-(but-3-en-1-yl)-4-methylbenzene (29.2 mg, 0.2 mmol) according to the general procedure, and the crude residue was purified by flash chromatography (pentane/EA = 20:1, R_f = 0.4) to give the product as a white solid (29.9 mg, 53%).
^{1}H NMR (300 MHz, $CDCl_3$) δ 7.80 (s, 1H), 7.30 – 7.24 (m, 1H), 7.12 (d, *J* = 7.8 Hz, 1H), 3.25 – 2.99 (m, 3H), 2.95 – 2.84 (m, 1H), 2.52 – 2.39 (m, 2H), 2.34 (s, 3H), 2.18 – 2.08 (m, 1H), 1.97 – 1.76 (m, 5H), 1.68 (d, *J* = 11.2 Hz, 1H), 1.44 – 1.20 (m, 5H). **^{13}C NMR** (75 MHz, $CDCl_3$) δ 212.4, 199.4, 141.2, 136.2, 134.2, 132.0, 128.6, 127.4, 51.2, 44.0, 41.1, 29.7, 29.0, 28.6, 28.4, 25.9, 25.8, 25.6, 20.9. **HRMS** (ESI) calcd for $C_{19}H_{25}O_2^+$ [M+H$^+$]: 285.1849, Found: 285.1854.

2-(2-Cyclohexyl-2-oxoethyl)-6,8-dimethyl-3,4-dihydronaphthalen-1(2*H*)-one (3ae):

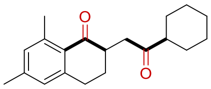

The title compound was prepared from bromocyclohexane (49.2 μL, 0.4 mmol) and 1-(but-3-en-1-yl)-3,5-dimethylbenzene (32.0 mg, 0.2 mmol) according to the general procedure, and the crude residue was purified by flash chromatography (pentane/EA = 20:1, R_f = 0.1) to give the product as a colorless oil (31.1 mg, 52%).
^{1}H NMR (300 MHz, $CDCl_3$) δ 6.88 (s, 2H), 3.21 – 3.01 (m, 3H), 2.93 – 2.84 (m, 1H), 2.56 (s, 3H), 2.51 – 2.39 (m, 2H), 2.30 (s, 3H), 2.13 – 2.04 (m, 1H), 1.97 – 1.87 (m, 2H), 1.85 – 1.74 (m, 3H), 1.71 – 1.63 (m, 1H), 1.45 – 1.18 (m,

5H). **^{13}C NMR** (75 MHz, $CDCl_3$) δ 212.6, 200.5, 145.1, 142.6, 141.4, 131.2, 128.6, 127.2, 51.1, 45.2, 41.4, 30.3, 29.3, 28.6, 28.3, 25.9, 25.7, 25.6, 23.1, 21.3. **HRMS** (ESI) calcd for $C_{20}H_{27}O_2^+$ [M+H$^+$]: 299.2006, Found: 299.2014.

7-(*tert*-Butyl)-2-(2-cyclohexyl-2-oxoethyl)-3,4-dihydronaphthalen-1(2*H*)-one (3af):

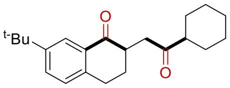

The title compound was prepared from bromocyclohexane (49.2 μL, 0.4 mmol) and 1-(but-3-en-1-yl)-4-(*tert*-butyl)benzene (37.6 mg, 0.2 mmol) according to the general procedure, and the crude residue was purified by flash chromatography (pentane/EA = 20:1, R_f = 0.4) to give the product as a white solid (39.1 mg, 60%).

^{1}H NMR (300 MHz, $CDCl_3$) δ 8.03 (d, *J* = 2.2 Hz, 1H), 7.51 (dd, *J* = 8.0, 2.2 Hz, 1H), 7.19 – 7.15 (m, 1H), 3.25 – 3.01 (m, 3H), 2.96 – 2.86 (m, 1H), 2.53 – 2.37 (m, 2H), 2.18 – 2.08 (m, 1H), 2.01 – 1.84 (m, 3H), 1.83 – 1.74 (m, 2H), 1.72 – 1.64 (m, 1H), 1.46 – 1.20 (m, 5H), 1.30 (s, 9H). **^{13}C NMR** (75 MHz, $CDCl_3$) δ 212.4, 199.5, 149.6, 141.3, 131.8, 130.7, 128.5, 123.8, 51.1, 44.1, 41.1, 34.6, 31.2, 29.6, 28.9, 28.6, 28.4, 25.9, 25.7, 25.6. **HRMS** (ESI) calcd for $C_{22}H_{31}O_2^+$ [M+H$^+$]: 327.2319, Found: 327.2324.

2-(2-Cyclohexyl-2-oxoethyl)-6-methoxy-3,4-dihydronaphthalen-1(2*H*)-one (3ag):

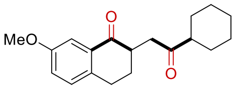

The title compound was prepared from bromocyclohexane (49.2 μL, 0.4 mmol) and 1-(but-3-en-1-yl)-4-methoxybenzene (32.4 mg, 0.2 mmol) according to the general procedure, and the crude residue was purified by flash chromatography (pentane/EA = 20:1, R_f = 0.1) to give the product as a colorless oil (32.5 mg, 54%).

^{1}H NMR (300 MHz, $CDCl_3$) δ 7.47 (d, *J* = 2.8 Hz, 1H), 7.18 – 7.09 (m, 1H), 7.03 (dd, *J* = 8.4, 2.8 Hz, 1H), 3.80 (s, 3H), 3.24 – 3.08 (m, 2H), 3.08 – 2.96 (m, 1H), 2.93 – 2.81 (m, 1H), 2.53 – 2.39 (m, 2H), 2.17 – 2.07 (m, 1H), 1.99 – 1.90 (m, 2H), 1.88 – 1.73 (m, 3H), 1.72 – 1.63 (m, 1H), 1.43 – 1.21 (m, 5H). **^{13}C NMR** (75 MHz, $CDCl_3$) δ 212.3, 199.1, 158.2, 136.7, 133.0, 129.9, 121.7, 109.2, 55.4, 51.1, 43.9, 41.1, 29.7, 28.6, 28.6, 28.4, 25.9, 25.7, 25.6. **HRMS** (ESI) calcd for $C_{19}H_{25}O_3^+$ [M+H$^+$]: 301.1798, Found: 301.1805.

2-(2-Cyclohexyl-2-oxoethyl)-7-(trifluoromethoxy)-3,4-dihydronaphthalen-1(2*H*)-one (3ah):

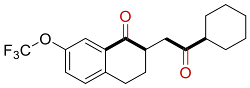

The title compound was prepared from bromocyclohexane (49.2 μL, 0.4 mmol) and 1-(but-3-en-1-yl)-4-(trifluoromethoxy)benzene (43.2 mg, 0.2 mmol) according to the general procedure, and the crude residue was purified by flash chromatography (pentane/EA = 20:1, R_f = 0.2) to give the product as a white solid (30.5 mg, 43%).

^{1}H NMR (300 MHz, $CDCl_3$) δ 7.88 – 7.79 (m, 1H), 7.33 – 7.25 (m, 2H), 3.23 – 3.03 (m, 3H), 3.02 – 2.90 (m, 1H), 2.55 – 2.40 (m, 2H), 2.20 – 2.12 (m, 1H), 1.99 – 1.84 (m, 3H), 1.84 – 1.75 (m, 2H), 1.71 – 1.63 (m, 1H), 1.44 – 1.19 (m, 5H). **^{13}C NMR** (75 MHz, $CDCl_3$) δ 212.1, 197.9, 147.9 (q, *J* = 2.0 Hz), 142.5, 133.6, 130.5, 125.9, 120.4 (q, *J* =

257.5 Hz), 119.3, 51.1, 43.7, 40.9, 29.2, 28.8, 28.5, 28.4, 25.8, 25.7, 25.6. **^{19}F NMR** (282 MHz, $CDCl_3$) δ -58.0. **HRMS** (ESI) calcd for $C_{19}H_{22}F_3O_3^+$ [M+H$^+$]: 355.1516, Found: 355.1516.

7-(Benzyloxy)-2-(2-cyclohexyl-2-oxoethyl)-3,4-dihydronaphthalen-1(2*H*)-one (3ai):

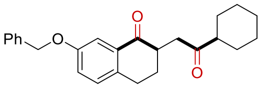

The title compound was prepared from bromocyclohexane (49.2 μL, 0.4 mmol) and 1-(benzyloxy)-4-(but-3-en-1-yl)benzene (47.7 mg, 0.2 mmol) according to the general procedure, and the crude residue was purified by flash chromatography (pentane/EA = 20:1, R_f = 0.2) to give the product as a colorless oil (32.2 mg, 43%).

^{1}H NMR (300 MHz, $CDCl_3$) δ 7.58 (d, *J* = 2.2 Hz, 1H), 7.47 – 7.28 (m, 5H), 7.19 – 7.08 (m, 2H), 5.07 (s, 2H), 3.24 – 2.98 (m, 3H), 2.93 – 2.83 (m, 1H), 2.55 – 2.40 (m, 2H), 2.19 – 2.07 (m, 1H), 2.00 – 1.89 (m, 2H), 1.89 – 1.73 (m, 3H), 1.72 – 1.65 (m, 1H), 1.46 – 1.19 (m, 5H). **^{13}C NMR** (75 MHz, $CDCl_3$) δ 212.3, 199.1, 157.4, 137.0, 136.6, 133.0, 130.0, 128.5, 128.0, 127.5, 122.3, 110.4, 70.1, 51.1, 43.8, 41.1, 29.7, 28.6, 28.4, 25.9, 25.7, 25.6. **HRMS** (ESI) calcd for $C_{25}H_{29}O_3^+$ [M+H$^+$]: 377.2111, Found: 377.2122.

7-(2-Cyclohexyl-2-oxoethyl)-8-oxo-5,6,7,8-tetrahydronaphthalen-2-yl trifluoromethanesulfonate (3aj):

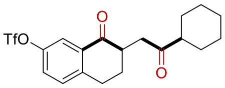

The title compound was prepared from bromocyclohexane (49.2 μL, 0.4 mmol) and 4-(but-3-en-1-yl)phenyl trifluoromethanesulfonate (56.0 mg, 0.2 mmol) according to the general procedure, and the crude residue was purified by flash chromatography (pentane/EA = 10:1, R_f = 0.3) to give the product as a white solid (40.8 mg, 49%).

^{1}H NMR (300 MHz, $CDCl_3$) δ 7.94 – 7.79 (m, 1H), 7.42 – 7.29 (m, 2H), 3.21 – 3.07 (m, 3H), 3.02 – 2.95 (m, 1H), 2.57 – 2.41 (m, 2H), 2.21 – 2.14 (m, 1H), 1.99 – 1.87 (m, 3H), 1.85 – 1.76 (m, 2H), 1.72 – 1.65 (m, 1H), 1.42 – 1.20 (m, 5H). **^{13}C NMR** (75 MHz, $CDCl_3$) δ 211.9, 197.3, 148.3, 144.2, 134.0, 131.0, 125.9, 119.8, 118.7 (d, *J* = 320.8 Hz), 51.1, 43.6, 40.8, 29.0, 28.9, 28.5, 28.4, 25.8, 25.7, 25.6. **^{19}F NMR** (282 MHz, $CDCl_3$) δ -72.8. **HRMS** (ESI) calcd for $C_{19}H_{22}F_3O_5S^+$ [M+H$^+$]: 419.1135, Found: 419.1141.

7-(2-Cyclohexyl-2-oxoethyl)-8-oxo-5,6,7,8-tetrahydronaphthalen-2-yl acetate (3ak):

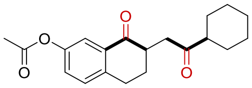

The title compound was prepared from bromocyclohexane (49.2 μL, 0.4 mmol) and methyl 4-(but-3-en-1-yl)phenyl acetate (38.0 mg, 0.2 mmol) according to the general procedure, and the crude residue was purified by flash chromatography (pentane/EA = 10:1, R_f = 0.2) to give the product as a colorless oil (30.9 mg, 47%).

^{1}H NMR (300 MHz, $CDCl_3$) δ 7.67 (d, *J* = 2.5 Hz, 1H), 7.28 – 7.23 (m, 1H), 7.18 (dd, *J* = 8.3, 2.5 Hz, 1H), 3.26 – 3.00 (m, 3H), 2.99 – 2.88 (m, 1H), 2.54 – 2.39 (m, 2H), 2.29 (s, 3H), 2.20 – 2.10 (m, 1H), 1.98 – 1.84 (m, 3H), 1.83 – 1.74 (m, 2H), 1.72 – 1.63 (m, 1H), 1.43 – 1.18 (m, 5H). **^{13}C NMR** (75 MHz, $CDCl_3$) δ 212.2, 198.2, 169.4, 149.2,

141.6, 133.3, 129.9, 126.8, 119.9, 51.1, 43.7, 40.9, 29.3, 28.8, 28.5, 28.4, 25.8, 25.7, 25.6, 21.0. **HRMS** (ESI) calcd for $C_{20}H_{25}O_4^+$ [M+H$^+$]: 329.1747, Found: 329.1755.

2-(2-Cyclohexyl-2-oxoethyl)-7-(dimethylamino)-3,4-dihydronaphthalen-1(2*H*)-one (3al):

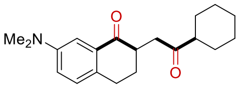

The title compound was prepared from bromocyclohexane (49.2 µL, 0.4 mmol) and 4-(but-3-en-1-yl)-*N*, *N*-dimethylaniline (35.0 mg, 0.2 mmol) according to the general procedure, and the crude residue was purified by flash chromatography (pentane/EA = 10:1, R_f = 0.4) to give the product as a yellow oil (31.0 mg, 50%).

^{1}H NMR (300 MHz, $CDCl_3$) δ 7.32 (d, *J* = 2.9 Hz, 1H), 7.10 (d, *J* = 8.4 Hz, 1H), 6.91 (dd, *J* = 8.5, 2.9 Hz, 1H), 3.22 – 3.06 (m, 2H), 3.05 – 2.95 (m, 1H), 2.94 (s, 6H), 2.89 – 2.77 (m, 1H), 2.52 – 2.36 (m, 2H), 2.16 – 2.06 (m, 1H), 2.00 – 1.75 (m, 5H), 1.73 – 1.63 (m, 1H), 1.47 – 1.18 (m, 5H). **^{13}C NMR** (75 MHz, $CDCl_3$) δ 212.5, 199.9, 149.3, 132.5, 129.4, 118.7, 109.9, 51.2, 44.2, 41.3, 40.7, 30.0, 28.6, 28.4, 25.9, 25.8, 25.6. **HRMS** (ESI) calcd for $C_{20}H_{28}NO_2^+$ [M+H$^+$]: 314.2115, Found: 314.2116.

2-(2-Cyclohexyl-2-oxoethyl)-7-(1*H*-pyrrol-1-yl)-3,4-dihydronaphthalen-1(2*H*)-one (3am):

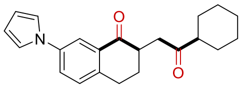

The title compound was prepared from bromocyclohexane (49.2 µL, 0.4 mmol) and 1-(4-(but-3-en-1-yl)phenyl)-1*H*-pyrrole (39.4 mg, 0.2 mmol) according to the general procedure, and the crude residue was purified by flash chromatography (pentane/EA = 20:1, R_f = 0.2) to give the product as a white solid (28.6 mg, 43%).

^{1}H NMR (300 MHz, $CDCl_3$) δ 8.01 (d, *J* = 2.5 Hz, 1H), 7.50 (dd, *J* = 8.2, 2.6 Hz, 1H), 7.29 (d, *J* = 8.2 Hz, 1H), 7.15 – 7.06 (m, 2H), 6.39 – 6.30 (m, 2H), 3.24 – 3.06 (m, 3H), 3.00 – 2.91 (m, 1H), 2.57 – 2.41 (m, 2H), 2.22 – 2.12 (m, 1H), 2.01 – 1.87 (m, 3H), 1.86 – 1.77 (m, 2H), 1.73 – 1.64 (m, 1H), 1.47 – 1.19 (m, 5H). **^{13}C NMR** (75 MHz, $CDCl_3$) δ 212.1, 198.6, 141.1, 139.3, 133.2, 130.1, 124.9, 119.1, 118.2, 110.6, 51.1, 43.9, 41.0, 29.4, 28.8, 28.6, 28.4, 25.8, 25.7, 25.6. **HRMS** (ESI) calcd for $C_{22}H_{26}NO_2^+$ [M+H$^+$]: 336.1958, Found: 336.1961.

***N*-(4-Bromobenzyl)-*N*-(7-(2-cyclohexyl-2-oxoethyl)-8-oxo-5,6,7,8-tetrahydronaphthalen-2-yl)-4-methylbenzenesulfonamide (3an):**

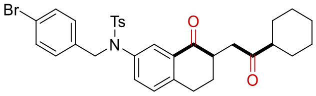

The title compound was prepared from bromocyclohexane (49.2 µL, 0.4 mmol) and *N*-(4-bromobenzyl)-*N*-(4-(but-3-en-1-yl)phenyl)-4-methylbenzenesulfonamide (94.1 mg, 0.2 mmol) according to the general procedure, and the crude residue was purified by flash chromatography (pentane/EA = 5:1, R_f = 0.2) to give the product as a colorless oil (73.5 mg, 60%).

^{1}H NMR (300 MHz, $CDCl_3$) δ ^{1}H NMR (300 MHz, $CDCl_3$) δ 7.53 – 7.46 (m, 3H), 7.35 – 7.23 (m, 5H), 7.13 – 7.07 (m, 3H), 4.70 – 4.54 (m, 2H), 3.16 – 2.98 (m, 3H), 2.92 – 2.82 (m, 1H), 2.48 – 2.36 (m, 2H), 2.43 (s, 3H), 2.14 –

2.05 (m, 1H), 1.95 – 1.86 (m, 2H), 1.85 – 1.73 (m, 3H), 1.72 – 1.64 (m, 1H), 1.43 – 1.20 (m, 5H). **^{13}C NMR** (75 MHz, $CDCl_3$) δ 212.1, 198.1, 143.9, 143.7, 137.5, 135.0, 134.7, 132.7, 131.5, 130.1, 129.7, 129.7, 127.6, 125.2, 121.6, 53.5, 51.1, 43.6, 40.9, 29.1, 29.0, 28.5, 28.3, 25.8, 25.7, 25.5, 21.5. **HRMS** (ESI) calcd for $C_{32}H_{35}BrNO_4S^+$ [M+H$^+$]: 608.1465, Found: 608.1475.

***N*-(7-(2-Cyclohexyl-2-oxoethyl)-8-oxo-5,6,7,8-tetrahydronaphthalen-2-yl)-*N*-methylacetamide (3ao):**

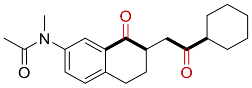

The title compound was prepared from bromocyclohexane (49.2 µL, 0.4 mmol) and *N*-(4-(but-3-en-1-yl)phenyl)-*N*-methylacetamide (40.6 mg, 0.2 mmol) according to the general procedure, and the crude residue was purified by flash chromatography (pentane/EA = 2:1, R_f = 0.3) to give the product as a colorless oil (42.2 mg, 62%).

^{1}H NMR (300 MHz, $CDCl_3$) δ 7.84 – 7.71 (m, 1H), 7.36 – 7.16 (m, 2H), 3.31 – 3.02 (m, 3H), 3.20 (s, 3H), 3.01 – 2.90 (m, 1H), 2.56 – 2.36 (m, 2H), 2.19 – 2.09 (m, 1H), 1.99 – 1.72 (m, 5H), 1.82 (s, 3H), 1.68 – 1.60 (m, 1H), 1.43 – 1.14 (m, 5H). **^{13}C NMR** (75 MHz, $CDCl_3$) δ 212.0, 198.3, 170.3, 143.4, 143.0, 133.4, 131.7, 130.3, 125.4, 51.0, 43.7, 40.8, 36.9, 29.1, 28.9, 28.5, 28.3, 25.7, 25.6, 25.5, 22.3. **HRMS** (ESI) calcd for $C_{21}H_{28}NO_3^+$ [M+H$^+$]: 342.2064, Found: 342.2069.

2-(2-Cyclohexyl-2-oxoethyl)-7-fluoro-3,4-dihydronaphthalen-1(2*H*)-one (3ap):

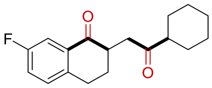

The title compound was prepared from bromocyclohexane (49.2 µL, 0.4 mmol) and 1-(but-3-en-1-yl)-4-fluorobenzene (30.0 mg, 0.2 mmol) according to the general procedure, and the crude residue was purified by flash chromatography (pentane/EA = 20:1, R_f = 0.3) to give the product as a colorless oil (27.8 mg, 48%).

^{1}H NMR (300 MHz, $CDCl_3$) δ 7.64 (dd, *J* = 9.2, 2.7 Hz, 1H), 7.25 – 7.09 (m, 2H), 3.23 – 2.99 (m, 3H), 2.97 – 2.86 (m, 1H), 2.54 – 2.40 (m, 2H), 2.18 – 2.09 (m, 1H), 1.98 – 1.84 (m, 3H), 1.84 – 1.75 (m, 2H), 1.71 – 1.64 (m, 1H), 1.42 – 1.19 (m, 5H). **^{13}C NMR** (75 MHz, $CDCl_3$) δ 212.1, 198.1, 161.4 (d, *J* = 246.0 Hz), 139.7 (d, *J* = 3.0 Hz), 133.8 (d, *J* = 5.9 Hz), 130.5 (d, *J* = 7.1 Hz), 120.6 (d, *J* = 22.2 Hz), 113.2 (d, *J* = 21.8 Hz), 51.1, 43.6, 40.9, 29.4, 28.7, 28.6, 28.4, 25.8, 25.7, 25.6. **^{19}F NMR** (282 MHz, $CDCl_3$) δ -115.3. **HRMS** (ESI) calcd for $C_{18}H_{22}FO_2^+$ [M+H$^+$]: 289.1598, Found: 289.1606.

7-Chloro-2-(2-cyclohexyl-2-oxoethyl)-3,4-dihydronaphthalen-1(2*H*)-one (3aq):

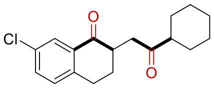

The title compound was prepared from bromocyclohexane (49.2 µL, 0.4 mmol) and 1-(but-3-en-1-yl)-4-chlorobenzene (33.3 mg, 0.2 mmol) according to the general procedure, and the crude residue was purified by flash chromatography (pentane/EA = 20:1, R_f = 0.1) to give the product as a colorless oil (24.1 mg, 40%).

^{1}**H NMR** (300 MHz, $CDCl_3$) δ 7.94 (d, *J* = 2.3 Hz, 1H), 7.39 (dd, *J* = 8.2, 2.3 Hz, 1H), 7.17 (d, *J* = 8.2 Hz, 1H), 3.24 – 2.97 (m, 3H), 2.96 – 2.84 (m, 1H), 2.55 – 2.39 (m, 2H), 2.19 – 2.08 (m, 1H), 1.97 – 1.83 (m, 3H), 1.82 – 1.73 (m, 2H), 1.71 – 1.64 (m, 1H), 1.43 – 1.19 (m, 5H). ^{13}**C NMR** (75 MHz, $CDCl_3$) δ 212.0, 198.0, 142.2, 133.4, 133.1, 132.7, 130.3, 127.0, 51.1, 43.7, 40.9, 29.2, 28.8, 28.5, 28.4, 25.8, 25.7, 25.5. **HRMS** (ESI) calcd for $C_{18}H_{22}ClO_2^+$ [M+H$^+$]: 305.1303, Found: 305.1308.

7-Bromo-2-(2-cyclohexyl-2-oxoethyl)-3,4-dihydronaphthalen-1(2*H*)-one (3ar):

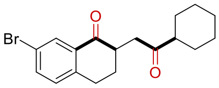

The title compound was prepared from bromocyclohexane (49.2 μL, 0.4 mmol) and 1-bromo-4-(but-3-en-1-yl)benzene (42.2 mg, 0.2 mmol) according to the general procedure, and the crude residue was purified by flash chromatography (pentane/EA = 20:1, R_f = 0.3) to give the product as a colorless oil (32.6 mg, 47%).
^{1}**H NMR** (400 MHz, $CDCl_3$) δ 8.09 (d, *J* = 2.2 Hz, 1H), 7.54 (dd, *J* = 8.1, 2.2 Hz, 1H), 7.11 (d, *J* = 8.2 Hz, 1H), 3.19 – 2.98 (m, 3H), 2.93 – 2.84 (m, 1H), 2.53 – 2.40 (m, 2H), 2.17 – 2.09 (m, 1H), 1.95 – 1.83 (m, 3H), 1.83 – 1.75 (m, 2H), 1.70 – 1.63 (m, 1H), 1.42 – 1.17 (m, 5H). ^{13}**C NMR** (101 MHz, $CDCl_3$) δ 212.0, 197.8, 142.7, 135.9, 133.7, 130.5, 130.1, 120.5, 51.1, 43.7, 40.9, 29.1, 28.8, 28.5, 28.4, 25.8, 25.7, 25.5. **HRMS** (ESI) calcd for $C_{18}H_{22}BrO_2^+$ [M+H$^+$]: 349.0798, Found: 349.0804.

Methyl 7-(2-cyclohexyl-2-oxoethyl)-8-oxo-5,6,7,8-tetrahydronaphthalene-2-carboxylate (3as):

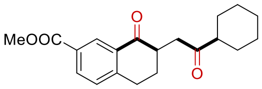

The title compound was prepared from bromocyclohexane (49.2 μL, 0.4 mmol) and methyl 4-(but-3-en-1-yl)benzoate (38.0 mg, 0.2 mmol) according to the general procedure, and the crude residue was purified by flash chromatography (pentane/EA = 10:1, R_f = 0.1) to give the product as a colorless oil (33.5 mg, 51%).
^{1}**H NMR** (300 MHz, $CDCl_3$) δ 8.64 (s, 1H), 8.10 (d, *J* = 7.7 Hz, 1H), 7.31 (d, *J* = 7.9 Hz, 1H), 3.91 (s, 3H), 3.33 – 3.05 (m, 3H), 3.05 – 2.88 (m, 1H), 2.61 – 2.39 (m, 2H), 2.24 – 2.11 (m, 1H), 1.99 – 1.85 (m, 3H), 1.84 – 1.75 (m, 2H), 1.72 – 1.64 (m, 1H), 1.38 – 1.23 (m, 5H). ^{13}**C NMR** (75 MHz, $CDCl_3$) δ 212.1, 198.2, 166.3, 148.7, 133.7, 132.3, 129.1, 128.9, 128.9, 52.2, 51.1, 43.9, 40.9, 29.6, 29.0, 28.6, 28.4, 25.8, 25.7, 25.6. **HRMS** (ESI) calcd for $C_{20}H_{25}O_4^+$ [M+H$^+$]: 329.1747, Found: 329.1758.

2-(2-Cyclohexyl-2-oxoethyl)-7-(trifluoromethyl)-3,4-dihydronaphthalen-1(2*H*)-one (3at):

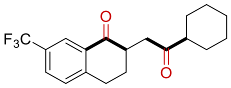

The title compound was prepared from bromocyclohexane (49.2 μL, 0.4 mmol) and 1-(but-3-en-1-yl)-4-(trifluoromethyl)benzene (40.0 mg, 0.2 mmol) according to the general procedure, and the crude residue was purified by flash chromatography (pentane/EA = 20:1, R_f = 0.3) to give the product as a white solid (34.2 mg, 51%).

^{1}H NMR (300 MHz, $CDCl_3$) δ 8.26 (d, *J* = 2.0 Hz, 1H), 7.67 (dd, *J* = 8.1, 2.1 Hz, 1H), 7.36 (d, *J* = 8.0 Hz, 1H), 3.24 – 3.08 (m, 3H), 3.05 – 2.96 (m, 1H), 2.59 – 2.39 (m, 2H), 2.22 – 2.13 (m, 1H), 2.01 – 1.86 (m, 3H), 1.84 – 1.75 (m, 2H), 1.71 – 1.64 (m, 1H), 1.44 – 1.19 (m, 5H). **^{13}C NMR** (75 MHz, $CDCl_3$) δ 212.0, 197.9, 147.5, 132.5, 129.5, 129.31 (q, *J* = 3.4 Hz), 129.2 (q, *J* = 33.0 Hz), 124.6 (q, *J* = 3.9 Hz), 123.7 (q, *J* = 272.4 Hz), 51.1, 43.8, 40.8, 29.3, 28.9, 28.5, 28.4, 25.8, 25.7, 25.5. **^{19}F NMR** (282 MHz, $CDCl_3$) δ -62.8. **HRMS** (ESI) calcd for $C_{19}H_{22}F_3O_2^+$ [M+H$^+$]: 339.1566, Found: 339.1580.

2-(2-Cyclohexyl-2-oxoethyl)-7-hydroxy-3,4-dihydronaphthalen-1(2*H*)-one (3au):

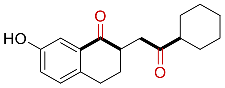

The title compound was prepared from bromocyclohexane (49.2 µL, 0.4 mmol) and 4-(but-3-en-1-yl)phenol (29.6 mg, 0.2 mmol) according to the general procedure, and the crude residue was purified by flash chromatography (pentane/EA = 5:1, R$_f$ = 0.3) to give the product as a white solid (22.5 mg, 39%).

^{1}H NMR (300 MHz, $CDCl_3$) δ 7.49 (d, *J* = 2.7 Hz, 1H), 7.10 (d, *J* = 8.3 Hz, 1H), 7.00 (dd, *J* = 8.3, 2.8 Hz, 1H), 6.42 (br s, 1H), 3.23 – 2.93 (m, 3H), 2.92 – 2.79 (m, 1H), 2.55 – 2.40 (m, 2H), 2.16 – 2.05 (m, 1H), 1.96 – 1.87 (m, 2H), 1.87 – 1.73 (m, 3H), 1.71 – 1.63 (m, 1H), 1.43 – 1.19 (m, 5H). **^{13}C NMR** (75 MHz, $CDCl_3$) δ 212.9, 199.8, 154.7, 136.4, 133.0, 130.1, 121.6, 112.7, 51.2, 43.9, 41.1, 29.7, 28.6, 28.5, 28.3, 25.8, 25.7, 25.6. **HRMS** (ESI) calcd for $C_{18}H_{23}O_3^+$ [M+H$^+$]: 287.1642, Found: 287.1651.

2-(2-Cyclohexyl-2-oxoethyl)-7-(hydroxymethyl)-3,4-dihydronaphthalen-1(2*H*)-one (3av):

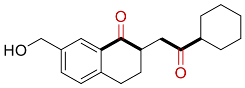

The title compound was prepared from bromocyclohexane (49.2 µL, 0.4 mmol) and (4-(but-3-en-1-yl)phenyl)methanol (32.4 mg, 0.2 mmol) according to the general procedure, and the crude residue was purified by flash chromatography (pentane/EA = 2:1, R$_f$ = 0.2) to give the product as a colorless oil (23.0 mg, 38%).

^{1}H NMR (300 MHz, $CDCl_3$) δ 7.92 (d, *J* = 1.9 Hz, 1H), 7.48 (dd, *J* = 7.9, 2.0 Hz, 1H), 7.21 (d, *J* = 7.9 Hz, 1H), 4.66 (s, 2H), 3.25 – 2.97 (m, 3H), 2.96 – 2.86 (m, 1H), 2.53 – 2.38 (m, 2H), 2.12 (m, 2H), 1.96 – 1.75 (m, 5H), 1.71 – 1.63 (m, 1H), 1.41 – 1.20 (m, 5H). **^{13}C NMR** (75 MHz, $CDCl_3$) δ 212.5, 199.2, 143.4, 139.5, 132.1, 132.0, 129.0, 125.5, 64.6, 51.1, 44.0, 41.0, 29.4, 29.1, 28.5, 28.4, 25.8, 25.7, 25.5. **HRMS** (ESI) calcd for $C_{19}H_{25}O_3^+$ [M+H$^+$]: 301.1798, Found: 301.1805.

2-(2-Cyclohexyl-2-oxoethyl)-4,4-dimethyl-3,4-dihydronaphthalen-1(2*H*)-one (3aw):

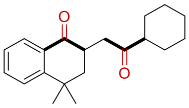

The title compound was prepared from bromocyclohexane (49.2 µL, 0.4 mmol) and (2-methylpent-4-en-2-yl)benzene (32.0 mg, 0.2 mmol) according to the general procedure, and the crude residue was purified by flash chromatography (pentane/EA = 20:1, R$_f$ = 0.4) to give the product as a colorless oil (25.5 mg, 43%).

^{1}**H NMR** (300 MHz, $CDCl_3$) δ 7.96 (d, *J* = 7.9 Hz, 1H), 7.57 – 7.46 (m, 1H), 7.40 (d, *J* = 7.9 Hz, 1H), 7.30 – 7.23 (m, 1H), 3.49 – 3.32 (m, 1H), 3.21 (dd, *J* = 17.6, 5.5 Hz, 1H), 2.51 – 2.36 (m, 2H), 1.99 – 1.85 (m, 4H), 1.84 – 1.76 (m, 2H), 1.68 (d, *J* = 10.6 Hz, 1H), 1.43 (s, 3H), 1.40 (s, 3H), 1.35 – 1.24 (m, 5H). ^{13}**C NMR** (75 MHz, $CDCl_3$) δ 212.4, 199.3, 151.9, 133.7, 130.9, 127.4, 126.3, 126.0, 51.1, 43.8, 41.0, 39.8, 34.4, 30.6, 29.6, 28.6, 28.4, 25.9, 25.8, 25.6. **HRMS** (ESI) calcd for $C_{20}H_{27}O_2^+$ [M+H$^+$]: 299.2006, Found: 299.2013.

2-(2-Cyclohexyl-2-oxoethyl)-3,4-dihydrophenanthren-1(2*H*)-one (3ax):

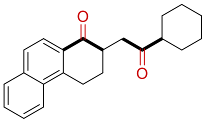

The title compound was prepared from bromocyclohexane (49.2 µL, 0.4 mmol) and 1-(but-3-en-1-yl)naphthalene (36.4 mg, 0.2 mmol) according to the general procedure, and the crude residue was purified by flash chromatography (pentane/EA = 20:1, R_f = 0.3) to give the product as a white solid (19.9 mg, 31%).
^{1}**H NMR** (300 MHz, $CDCl_3$) δ 8.16 – 8.09 (m, 1H), 8.06 (d, *J* = 8.7 Hz, 1H), 7.90 – 7.81 (m, 1H), 7.74 (d, *J* = 8.7 Hz, 1H), 7.67 – 7.51 (m, 2H), 3.65 – 3.49 (m, 1H), 3.41 – 3.19 (m, 3H), 2.56 – 2.43 (m, 2H), 2.42 – 2.32 (m, 1H), 2.06 – 1.91 (m, 3H), 1.86 – 1.77 (m, 2H), 1.72 – 1.64 (m, 1H), 1.45 – 1.21 (m, 5H). ^{13}**C NMR** (75 MHz, $CDCl_3$) δ 212.5, 199.3, 142.4, 135.6, 131.3, 129.6, 128.7, 128.2, 127.0, 126.7, 124.7, 122.9, 51.2, 43.0, 40.9, 28.9, 28.6, 28.4, 25.9, 25.8, 25.6, 25.6. **HRMS** (ESI) calcd for $C_{22}H_{25}O_2^+$ [M+H$^+$]: 321.1849, Found: 321.1848.

7-(*tert*-Butyl)-2-(2-oxohexyl)-3,4-dihydronaphthalen-1(2*H*)-one (3bf):

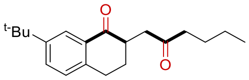

The title compound was prepared from 1-bromobutane (43.0 µL, 0.4 mmol) and and 1-(but-3-en-1-yl)-4-(*tert*-butyl)benzene (37.6 mg, 0.2 mmol) according to the general procedure, and the crude residue was purified by flash chromatography (pentane/EA = 20:1, R_f = 0.3) to give the product as a colorless oil (23.9 mg, 40%).
^{1}**H NMR** (300 MHz, $CDCl_3$) δ 8.04 (d, *J* = 2.2 Hz, 1H), 7.52 (dd, *J* = 8.1, 2.2 Hz, 1H), 7.17 (d, *J* = 8.1 Hz, 1H), 3.22 – 3.02 (m, 3H), 2.96 – 2.87 (m, 1H), 2.66 – 2.49 (m, 2H), 2.47 – 2.36 (m, 1H), 2.22 – 2.11 (m, 1H), 1.98 – 1.82 (m, 1H), 1.67 – 1.58 (m, 2H), 1.39 – 1.29 (m, 2H), 1.32, (s, 9H), 0.93 (t, *J* = 7.3 Hz, 3H). ^{13}**C NMR** (75 MHz, $CDCl_3$) δ 209.6, 199.5, 149.7, 141.3, 131.8, 130.8, 128.5, 123.8, 44.3, 43.1, 43.1, 34.6, 31.2, 29.6, 28.9, 25.9, 22.4, 13.9. **HRMS** (ESI) calcd for $C_{20}H_{29}O_2^+$ [M+H$^+$]: 301.2162, Found: 301.2173.

7-(*tert*-Butyl)-2-(2-oxodecyl)-3,4-dihydronaphthalen-1(2*H*)-one (3cf):

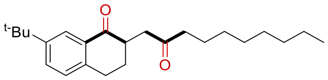

The title compound was prepared from 1-bromooctane (69.1 µL, 0.4 mmol) and 1-(but-3-en-1-yl)-4-(*tert*-butyl)benzene (37.6 mg, 0.2 mmol) according to the general procedure, and the crude residue was purified by flash chromatography (pentane/EA = 20:1, R_f = 0.4) to give the product as a colorless oil (32.1 mg, 45%).

^{1}H NMR (300 MHz, $CDCl_3$) δ 8.03 (d, *J* = 2.2 Hz, 1H), 7.52 (dd, *J* = 8.0, 2.3 Hz, 1H), 7.18 (d, *J* = 8.1 Hz, 1H), 3.25 – 2.99 (m, 3H), 2.97 – 2.86 (m, 1H), 2.65 – 2.36 (m, 3H), 2.20 – 2.11 (m, 1H), 1.97 – 1.83 (m, 1H), 1.67 – 1.58 (m, 2H), 1.32 – 1.25 (m, 10H), 1.31 (s, 9H), 0.87 (t, *J* = 7.2 Hz, 3H). **^{13}C NMR** (75 MHz, $CDCl_3$) δ 209.7, 199.5, 149.6, 141.3, 131.7, 130.8, 128.5, 123.8, 44.3, 43.4, 43.1, 34.6, 31.8, 31.2, 29.6, 29.4, 29.2, 29.1, 28.9, 23.8, 22.6, 14.1. **HRMS** (ESI) calcd for $C_{24}H_{37}O_2^+$ [M+H$^+$]: 357.2788, Found: 357.2801.

7-(*tert*-Butyl)-2-(6-methyl-2-oxoheptyl)-3,4-dihydronaphthalen-1(2*H*)-one (3df):

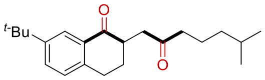

The title compound was prepared from 1-bromo-4-methylpentane (58.2 µL, 0.4 mmol) and 1-(but-3-en-1-yl)-4-(*tert*-butyl)benzene (37.6 mg, 0.2 mmol) according to the general procedure, and the crude residue was purified by flash chromatography (pentane/EA = 20:1, R_f = 0.4) to give the product as a colorless oil (35.0 mg, 53%).

^{1}H NMR (300 MHz, $CDCl_3$) δ 8.03 (d, *J* = 2.3 Hz, 1H), 7.52 (dd, *J* = 8.0, 2.3 Hz, 1H), 7.18 (d, *J* = 8.1 Hz, 1H), 3.24 – 3.00 (m, 3H), 2.98 – 2.87 (m, 1H), 2.64 – 2.46 (m, 2H), 2.45 – 2.35 (m, 1H), 2.23 – 2.09 (m, 1H), 1.98 – 1.82 (m, 1H), 1.67 – 1.60 (m, 2H), 1.59 – 1.52 (m, 1H), 1.32 (s, 9H), 1.24 – 1.16 (m, 2H), 0.89 (d, *J* = 6.4 Hz, 6H). **^{13}C NMR** (75 MHz, $CDCl_3$) δ 209.7, 199.5, 149.6, 141.3, 131.7, 130.8, 128.5, 123.8, 44.3, 43.6, 43.1, 38.5, 34.6, 31.2, 29.6, 28.9, 27.9, 22.5, 22.5, 21.6. **HRMS** (ESI) calcd for $C_{22}H_{33}O_2^+$ [M+H$^+$]: 329.2475, Found: 329.2488.

7-(7-(*tert*-Butyl)-1-oxo-1,2,3,4-tetrahydronaphthalen-2-yl)-6-oxoheptyl acetate (3ef):

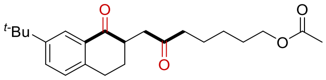

The title compound was prepared from 5-bromopentyl acetate (66.6 µL, 0.4 mmol) and 1-(but-3-en-1-yl)-4-(*tert*-butyl)benzene (37.6 mg, 0.2 mmol) according to the general procedure, and the crude residue was purified by flash chromatography (pentane/EA = 10:1, R_f = 0.1) to give the product as a colorless oil (27.1 mg, 36%).

^{1}H NMR (300 MHz, $CDCl_3$) δ 8.02 (d, *J* = 2.2 Hz, 1H), 7.51 (dd, *J* = 8.1, 2.3 Hz, 1H), 7.17 (d, *J* = 8.0 Hz, 1H), 4.06 (t, *J* = 6.6 Hz, 2H), 3.23 – 2.99 (m, 3H), 2.97 – 2.86 (m, 1H), 2.69 – 2.45 (m, 2H), 2.43 – 2.33 (m, 1H), 2.18 – 2.11 (m, 1H), 2.04 (s, 3H), 1.97 – 1.83 (m, 1H), 1.71 – 1.61 (m, 4H), 1.44 – 1.36 (m, 2H), 1.31 (s, 9H). **^{13}C NMR** (75 MHz, $CDCl_3$) δ 209.1, 199.5, 171.2, 149.7, 141.3, 131.7, 130.8, 128.5, 123.8, 64.3, 44.4, 43.2, 43.1, 34.6, 31.2, 29.6, 28.9, 28.5, 25.6, 23.3, 21.0. **HRMS** (ESI) calcd for $C_{23}H_{33}O_4^+$ [M+H$^+$]: 373.2373, Found: 373.2388.

7-(*tert*-Butyl)-2-(6,6,6-trifluoro-2-oxohexyl)-3,4-dihydronaphthalen-1(2*H*)-one (3ff):

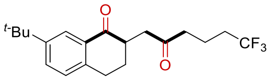

The title compound was prepared from 4-bromo-1,1,1-trifluorobutane (76.0 mg, 0.4 mmol) and 1-(but-3-en-1-yl)-4-(*tert*-butyl)benzene (37.6 mg, 0.2 mmol) according to the general procedure, and the crude residue was purified by flash chromatography (pentane/EA = 10:1, R_f = 0.3) to give the product as a colorless oil (33.2 mg, 47%).

^{1}H NMR (300 MHz, $CDCl_3$) δ 8.02 (d, *J* = 2.2 Hz, 1H), 7.52 (dd, *J* = 8.1, 2.2 Hz, 1H), 7.18 (d, *J* = 8.1 Hz, 1H), 3.25 – 2.99 (m, 3H), 2.98 – 2.88 (m, 1H), 2.78 (dt, *J* = 17.9, 7.1 Hz, 1H), 2.61 (dt, *J* = 17.9, 7.0 Hz, 1H), 2.37 (dd, *J* = 16.7, 5.2 Hz, 1H), 2.25 – 2.09 (m, 3H), 2.01 – 1.85 (m, 3H), 1.32 (s, 9H). **^{13}C NMR** (75 MHz, $CDCl_3$) δ 207.8, 199.4, 149.7, 141.3, 131.6, 130.9, 128.6, 127.0 (q, *J* = 277.5 Hz), 123.7, 44.7, 43.2, 41.4, 34.6, 32.8 (q, *J* = 28.5 Hz), 31.1, 29.7, 28.9, 16.0 (q, *J* = 3.2 Hz). **^{19}F NMR** (282 MHz, $CDCl_3$) δ -66.2. **HRMS** (ESI) calcd for $C_{20}H_{26}F_3O_2^+$ [M+H$^+$]: 355.1879, Found: 355.1893.

7-(*tert*-Butyl)-2-(3-methyl-2-oxobutyl)-3,4-dihydronaphthalen-1(2*H*)-one (3gf):

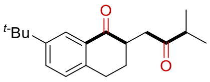

The title compound was prepared from 2-bromopropane (37.6 µL, 0.4 mmol) and 1-(but-3-en-1-yl)-4-(*tert*-butyl)benzene (37.6 mg, 0.2 mmol) according to the general procedure, and the crude residue was purified by flash chromatography (pentane/EA = 20:1, R_f = 0.4) to give the product as a white solid (29.1 mg, 51%). **^{1}H NMR** (300 MHz, CD_3OD) δ 7.98 (d, *J* = 2.2 Hz, 1H), 7.60 (dd, *J* = 8.1, 2.3 Hz, 1H), 7.27 – 7.23 (m, 1H), 3.17 – 3.03 (m, 3H), 3.00 – 2.91 (m, 1H), 2.85 – 2.75 (m, 1H), 2.70 – 2.60 (m, 1H), 2.21 – 2.07 (m, 1H), 2.04 – 1.85 (m, 1H), 1.34 (s, 9H), 1.17 (d, *J* = 5.1 Hz, 3H), 1.15 (d, *J* = 5.1 Hz, 3H). **^{13}C NMR** (75 MHz, $CDCl_3$) δ 213.1, 199.5, 149.6, 141.3, 131.8, 130.7, 128.5, 123.8, 44.2, 41.2, 40.9, 34.6, 31.2, 29.6, 28.9, 18.3, 18.2. **HRMS** (ESI) calcd for $C_{19}H_{27}O_2^+$ [M+H$^+$]: 287.2006, Found: 287.2016.

7-(*tert*-Butyl)-2-(3-methyl-2-oxopentyl)-3,4-dihydronaphthalen-1(2H)-one (3hf):

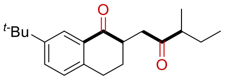

The title compound was prepared from 2-bromobutane (43.7 µL, 0.4 mmol) and 1-(but-3-en-1-yl)-4-(*tert*-butyl)benzene (37.6 mg, 0.2 mmol) according to the general procedure, and the crude residue was purified by flash chromatography (pentane/EA = 20:1, R_f = 0.4) to give the product as a colorless oil (31.4 mg, 52%). **^{1}H NMR** (300 MHz, $CDCl_3$) δ 8.04 (d, *J* = 1.7 Hz, 1H), 7.52 (dd, *J* = 8.1, 2.3 Hz, 1H), 7.18 (d, *J* = 8.1 Hz, 1H), 3.26 – 3.02 (m, 3H), 2.97 – 2.86 (m, 1H), 2.64 – 2.40 (m, 2H), 2.22 – 2.09 (m, 1H), 1.98 – 1.85 (m, 1H), 1.84 – 1.69 (m, 1H), 1.50 – 1.38 (m, 1H), 1.32 (s, 9H), 1.14 (t, *J* = 7.2 Hz, 3H), 0.93 (td, *J* = 7.4, 5.3 Hz, 3H). **^{13}C NMR** (75 MHz, $CDCl_3$) δ 213.0, 199.5, 149.6, 141.3, 131.8, 130.7, 128.5, 123.8, 48.2, 44.0, 41.8, 34.6, 31.2, 29.6, 28.9, 26.0, 15.9, 11.7. **HRMS** (ESI) calcd for $C_{20}H_{29}O_2^+$ [M+H$^+$]: 301.2162, Found: 301.2176.

7-(*tert*-Butyl)-2-(3-methyl-2-oxopentadecyl)-3,4-dihydronaphthalen-1(2*H*)-one (3if):

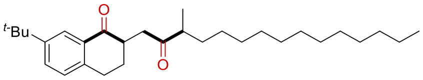

The title compound was prepared from 2-bromotetradecane (107.1 µL, 0.4 mmol) and 1-(but-3-en-1-yl)-4-(*tert*-butyl)benzene (37.6 mg, 0.2 mmol) according to the general procedure, and the crude residue was purified by flash chromatography (pentane/EA = 20:1, R_f = 0.6) to give the product as a colorless oil (34.2 mg, 39%).

^{1}H NMR (300 MHz, $CDCl_3$) δ 8.08 – 8.01 (m, 1H), 7.51 (dd, *J* = 8.0, 2.3 Hz, 1H), 7.18 (dt, *J* = 8.1, 0.6 Hz, 1H), 3.25 – 3.02 (m, 3H), 2.97 – 2.86 (m, 1H), 2.69 – 2.55 (m, 1H), 2.55 – 2.39 (m, 1H), 2.22 – 2.09 (m, 1H), 1.97 – 1.81 (m, 1H), 1.79 – 1.66 (m, 1H), 1.33 – 1.25 (m, 30H), 1.14 (t, *J* = 7.0 Hz, 3H), 0.91 – 0.85 (m, 3H). **^{13}C NMR** (75 MHz, $CDCl_3$) δ 213.0, 199.4, 149.6, 141.3, 131.8, 130.7, 128.5, 123.8, 46.7, 43.8, 41.4, 34.6, 32.8, 31.9, 31.2, 29.7, 29.7, 29.7, 29.6, 29.6, 29.6, 29.5, 29.3, 28.9, 27.3, 22.7, 16.4, 14.1. **HRMS** (ESI) calcd for $C_{30}H_{49}O_2^+$ [M+H$^+$]: 441.3727, Found: 441.3730.

7-(*tert*-Butyl)-2-(3-methyl-2-oxo-6-phenoxyheptyl)-3,4-dihydronaphthalen-1(2*H*)-one (3jf):

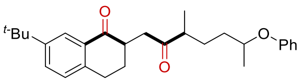

The title compound was prepared from ((5-bromohexan-2-yl)oxy)benzene (102.9 mg, 0.4 mmol) and 1-(but-3-en-1-yl)-4-(*tert*-butyl)benzene (37.6 mg, 0.2 mmol) according to the general procedure, and the crude residue was purified by flash chromatography (pentane/EA = 20:1, R_f = 0.2) to give the product as a white solid (39.2 mg, 47%, d.r. = 1:1).

^{1}H NMR (300 MHz, $CDCl_3$) 7.98 – 7.92 (m, 1H), 7.47 – 7.40 (m, 1H), 7.21 – 7.15 (m, 2H), 7.10 (d, *J* = 8.1 Hz, 1H), 6.86 – 6.79 (m, 3H), 4.37 – 4.23 (m, 1H), 3.19 – 2.92 (m, 3H), 2.89 – 2.77 (m, 1H), 2.67 – 2.54 (m, 1H), 2.46 – 2.29 (m, 1H), 2.10 – 1.97 (m, 1H), 1.89 – 1.69 (m, 2H), 1.67 – 1.48 (m, 3H), 1.31 (s, 9H), 1.25 – 1.21 (m, 3H), 1.11 (d, *J* = 7.2 Hz, 1.5H), 1.08 (d, *J* = 7.0 Hz, 1.5H). **^{13}C NMR** (75 MHz, $CDCl_3$) δ 212.6, 199.4, 158.0, 149.6, 141.3, 131.8, 130.7, 129.4, 128.5, 123.8, 120.6, 115.9, 73.7, 46.5, 44.0, 41.9, 34.6, 34.1, 31.2, 29.6, 28.9, 28.5, 19.8, 16.7. **HRMS** (ESI) calcd for $C_{28}H_{37}O_3^+$ [M+H$^+$]: 421.2737, Found: 421.2750.

7-(*tert*-Butyl)-2-(2-cyclobutyl-2-oxoethyl)-3,4-dihydronaphthalen-1(2*H*)-one (3kf):

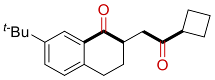

The title compound was prepared from cyclobutyl bromide (37.6 µL, 0.4 mmol) and 1-(but-3-en-1-yl)-4-(*tert*-butyl)benzene (37.6 mg, 0.2 mmol) according to the general procedure, and the crude residue was purified by flash chromatography (pentane/EA = 20:1, R_f = 0.3) to give the product as a white solid (32.0 mg, 54%).

^{1}H NMR (300 MHz, $CDCl_3$) δ 8.03 (d, *J* = 2.3 Hz, 1H), 7.51 (dd, *J* = 8.1, 2.3 Hz, 1H), 7.17 (d, *J* = 8.1 Hz, 1H), 3.38 (p, *J* = 8.5 Hz, 1H), 3.20 – 2.99 (m, 3H), 2.91 (dt, *J* = 16.8, 3.8 Hz, 1H), 2.45 – 2.31 (m, 2H), 2.29 – 2.11 (m, 4H), 2.03 – 1.80 (m, 3H), 1.31 (s, 9H). **^{13}C NMR** (75 MHz, $CDCl_3$) δ 210.1, 199.4, 149.6, 141.3, 131.8, 130.7, 128.5, 123.8, 45.8, 44.0, 40.3, 34.6, 31.2, 29.6, 28.9, 24.4, 24.3, 17.8. **HRMS** (ESI) calcd for $C_{20}H_{27}O_2^+$ [M+H$^+$]: 299.2006, Found: 299.2012.

***N*-(7-(2-((1*R*,4*S*)-Bicyclo[2.2.1]heptan-2-yl)-2-oxoethyl)-8-oxo-5,6,7,8-tetrahydronaphthalen-2-yl)-*N*-methylacetamide (3lf):**

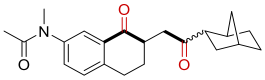

The title compound was prepared from *exo*-2-bromonorbornane (51.4 µL, 0.4 mmol) and *N*-(4-(but-3-en-1-yl)phenyl)-*N*-methylacetamide (40.6 mg, 0.2 mmol) according to the general procedure, and the

crude residue was purified by flash chromatography (pentane/EA = 2:1, R_f = 0.2) to give the product as a colorless oil (44.9 mg, 64%).

^{1}H NMR (300 MHz, $CDCl_3$) δ 7.83 – 7.75 (m, 1H), 7.32 – 7.22 (m, 2H), 3.20 (s, 3H), 3.17 – 3.03 (m, 3H), 3.00 – 2.91 (m, 1H), 2.62 – 2.41 (m, 3H), 2.30 – 2.24 (m, 1H), 2.19 – 2.10 (m, 1H), 2.01 – 1.77 (m, 2H), 1.83 (s, 3H), 1.59 – 1.43 (m, 2H), 1.38 – 1.18 (m, 4H), 1.14 – 1.05 (m, 1H). **^{13}C NMR** (75 MHz, $CDCl_3$) δ 210.0, 198.3, 170.3, 143.4, 143.1, 133.4, 131.7, 130.3, 125.5, 54.4, 44.0, 41.9, 40.0, 37.0, 36.1, 36.0, 35.9, 32.6, 29.7, 29.1, 28.8, 22.3. **HRMS** (ESI) calcd for $C_{22}H_{28}NO_3^+$ [M+H$^+$]: 354.2064, Found: 354.2072.

7-(*tert*-Butyl)-2-(2-oxo-2-(tetrahydrofuran-3-yl)ethyl)-3,4-dihydronaphthalen-1(2*H*)-one (3mf):

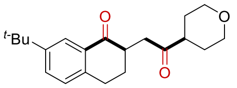

The title compound was prepared from 4-bromotetrahyropyran (45.0 µL, 0.4 mmol) and 1-(but-3-en-1-yl)-4-(*tert*-butyl)benzene (37.6 mg, 0.2 mmol) according to the general procedure, and the crude residue was purified by flash chromatography (pentane/EA = 5:1, R_f = 0.2) to give the product as a colorless oil (26.5 mg, 40%).

^{1}H NMR (300 MHz, $CDCl_3$) δ 8.02 (d, *J* = 2.3 Hz, 1H), 7.52 (dd, *J* = 8.1, 2.2 Hz, 1H), 7.18 (d, *J* = 8.1 Hz, 1H), 4.07 – 3.99 (m, 2H), 3.52 – 3.42 (m, 2H), 3.26 – 3.04 (m, 3H), 2.98 – 2.88 (m, 1H), 2.80 – 2.67 (m, 1H), 2.49 – 2.34 (m, 1H), 2.20 – 2.09 (m, 1H), 1.91– 1.78 (m, 3H), 1.80 – 1.69 (m, 2H), 1.31 (s, 9H). **^{13}C NMR** (75 MHz, $CDCl_3$) δ 210.3, 199.4, 149.7, 141.3, 131.7, 130.8, 128.6, 123.8, 67.3, 47.9, 44.4, 40.9, 34.6, 31.2, 29.7, 29.0, 28.3, 28.1. **HRMS** (ESI) calcd for $C_{21}H_{29}O_3^+$ [M+H$^+$]: 329.2111, Found: 329.2120.

7-(*tert*-Butyl)-2-(2-oxo-2-(1-tosylpiperidin-4-yl)ethyl)-3,4-dihydronaphthalen-1(2*H*)-one (3nf):

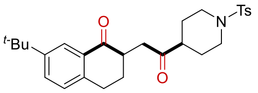

The title compound was prepared from 4-bromo-1-tosylpiperidine (127.3 mg, 0.4 mmol) and 1-(but-3-en-1-yl)-4-(*tert*-butyl)benzene (37.6 mg, 0.2 mmol) according to the general procedure, and the crude residue was purified by flash chromatography (pentane/EA = 5:1, R_f = 0.1) to give the product as a colorless oil (60.4 mg, 63%).

^{1}H NMR (300 MHz, $CDCl_3$) δ 7.97 (d, *J* = 2.2 Hz, 1H), 7.65 (d, *J* = 8.1 Hz, 2H), 7.51 (dd, *J* = 8.0, 2.2 Hz, 1H), 7.32 (d, *J* = 8.0 Hz, 2H), 7.17 (d, *J* = 8.1 Hz, 1H), 3.80 – 3.70 (m, 2H), 3.23 – 2.98 (m, 3H), 2.97 – 2.85 (m, 1H), 2.49 – 2.40 (m, 3H), 2.43 (s, 3H), 2.37 – 2.29 (m, 1H), 2.14 – 1.95 (m, 3H), 1.92 – 1.84 (m, 1H), 1.83 – 1.71 (m, 2H), 1.30 (s, 9H). **^{13}C NMR** (75 MHz, $CDCl_3$) δ 209.9, 199.3, 149.7, 143.5, 141.2, 133.1, 131.5, 130.9, 129.6, 128.6, 127.6, 123.7, 47.8, 45.6, 45.5, 44.6, 41.1, 34.6, 31.1, 29.7, 28.9, 27.1, 26.9, 21.5. **HRMS** (ESI) calcd for $C_{28}H_{36}NO_4S^+$ [M+H$^+$]: 482.2360, Found: 482.2370.

2-(2-(1-Benzylpiperidin-4-yl)-2-oxoethyl)-7-(*tert*-butyl)-3,4-dihydronaphthalen-1(2*H*)-one (3of):

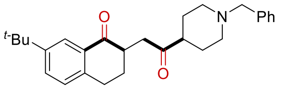

The title compound was prepared from 1-benzyl-4-bromopiperidine (101.7 mg, 0.4 mmol) and 1-(but-3-en-1-yl)-4-(*tert*-butyl)benzene (37.6 mg, 0.2 mmol) according to the general procedure, and the crude residue was purified by flash chromatography (pentane/EA = 3:1, R_f = 0.2) to give the product as a colorless oil (39.8 mg, 48%, d.r. = 87 : 13).

^{1}H NMR (300 MHz, $CDCl_3$) δ 8.02 (d, *J* = 2.2 Hz, 1H), 7.52 (dd, *J* = 8.0, 2.2 Hz, 1H), 7.34 – 7.24 (m, 5H), 7.18 (d, *J* = 8.1 Hz, 1H), 3.54 (s, 1.74H), 3.51 (s, 0.26H), 3.23 – 3.03 (m, 3H), 3.00 – 2.87 (m, 3H), 2.54 – 2.37 (m, 2H), 2.17 – 2.04 (m, 3H), 1.97 – 1.86 (m, 3H), 1.84 – 1.67 (m, 2H), 1.32 (s, 9H). **^{13}C NMR** (75 MHz, $CDCl_3$) δ 211.3, 199.5, 149.6, 141.3, 131.7, 130.8, 129.1, 129.1, 128.5, 128.2, 127.0, 123.8, 63.1, 53.0, 52.4, 48.9, 44.2, 41.0, 34.6, 31.2, 29.7, 28.9, 27.5, 25.4. **HRMS** (ESI) calcd for $C_{28}H_{36}NO_2^+$ [M+H$^+$]: 418.2741, Found: 418.2742.

2-(2-((3*R*,5*R*,7*R*)-Adamantan-1-yl)-2-oxoethyl)-3,4-dihydronaphthalen-1(2*H*)-one (3pf):

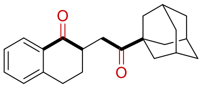

The title compound was prepared from 1-bromoadamantane (86.0 mg, 0.4 mmol) and but-3-en-1-ylbenzene (30 µL, 0.2 mmol) according to the general procedure, and the crude residue was purified by flash chromatography (pentane/EA = 20:1, R_f = 0.2) to give the product as a white solid (24.1 mg, 37%).

^{1}H NMR (300 MHz, $CDCl_3$) δ 8.00 (dd, *J* = 7.8, 1.5 Hz, 1H), 7.45 (td, *J* = 7.5, 1.5 Hz, 1H), 7.31 – 7.21 (m, 2H), 3.28 – 3.05 (m, 3H), 2.97 – 2.88 (m, 1H), 2.55 – 2.46 (m, 1H), 2.16 – 2.03 (m, 4H), 1.94 – 1.85 (m, 6H), 1.81 – 1.66 (m, 7H). **^{13}C NMR** (75 MHz, $CDCl_3$) δ 213.8, 199.4, 144.1, 133.2, 132.4, 128.7, 127.3, 126.5, 46.4, 43.6, 38.3, 36.8, 36.6, 29.5, 29.4, 28.0. **HRMS** (ESI) calcd for $C_{22}H_{27}O_2^+$ [M+H$^+$]: 323.2006, Found: 323.2011.

3-(2-Cyclohexyl-2-oxoethyl)-1-methyl-2,3-dihydroquinolin-4(1*H*)-one (5ab):

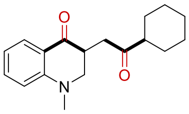

The title compound was prepared from bromocyclohexane (49.2 µL, 0.4 mmol) and *N*-allyl-*N*-methylaniline (29.4 mg, 0.2 mmol) according to the general procedure, and the crude residue was purified by flash chromatography (pentane/EA = 10:1, R_f = 0.3) to give the product as a yellow oil (25.1 mg, 44%).

^{1}H NMR (300 MHz, $CDCl_3$) δ 7.87 (dd, *J* = 7.8, 1.7 Hz, 1H), 7.43 – 7.33 (m, 1H), 6.77 – 6.65 (m, 2H), 3.48 – 3.35 (m, 1H), 3.34 – 3.09 (m, 3H), 2.97 (s, 3H), 2.49 – 2.33 (m, 2H), 1.96 – 1.71 (m, 4H), 1.71 – 1.61 (m, 1H), 1.41 – 1.17 (m, 5H). **^{13}C NMR** (75 MHz, $CDCl_3$) δ 211.9, 194.8, 152.4, 135.3, 128.2, 119.1, 117.0, 113.0, 55.7, 51.0, 42.1, 39.1, 37.6, 28.5, 28.3, 25.8, 25.6, 25.5. **HRMS** (ESI) calcd for $C_{18}H_{24}NO_2^+$ [M+H$^+$]: 286.1802, Found: 286.1809.

tert-Butyl 3-(2-cyclohexyl-2-oxoethyl)-4-oxo-3,4-dihydroquinoline-1(2*H*)-carboxylate (5ac):

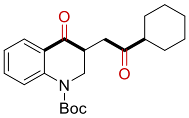

The title compound was prepared from bromocyclohexane (49.2 μL, 0.4 mmol) and *tert*-butyl allyl(phenyl)carbamate (46.6 mg, 0.2 mmol) according to the general procedure, and the crude residue was purified by flash chromatography (pentane/EA = 20:1, R_f = 0.2) to give the product as a colorless oil (16.1 mg, 22%).

^{1}H NMR (300 MHz, $CDCl_3$) δ 7.96 (ddd, *J* = 7.9, 1.8, 0.5 Hz, 1H), 7.81 – 7.73 (m, 1H), 7.48 (ddd, *J* = 8.5, 7.2, 1.7 Hz, 1H), 7.13 (ddd, *J* = 7.9, 7.2, 1.1 Hz, 1H), 4.38 (dd, *J* = 13.2, 4.7 Hz, 1H), 3.70 (dd, *J* = 13.1, 11.0 Hz, 1H), 3.29 – 3.18 (m, 1H), 2.95 (dd, *J* = 18.0, 4.4 Hz, 1H), 2.65 (dd, *J* = 17.9, 7.4 Hz, 1H), 2.46 – 2.35 (m, 1H), 1.95 – 1.85 (m, 2H), 1.82 – 1.75 (m, 2H), 1.69 – 1.63 (m, 1H), 1.55 (s, 9H), 1.39 – 1.19 (m, 5H). **^{13}C NMR** (75 MHz, $CDCl_3$) δ 210.8, 195.9, 152.8, 144.1, 133.9, 127.5, 124.3, 123.8, 123.4, 82.2, 50.9, 48.3, 43.5, 38.0, 28.5, 28.4, 28.2, 25.8, 25.6, 25.6. **HRMS** (ESI) calcd for $C_{22}H_{29}NNaO_4^+$ [M+H$^+$]: 394.1989, Found: 394.1995.

3-(2-Cyclohexyl-2-oxoethyl)-1-ethyl-2,3-dihydroquinolin-4(1*H*)-one (5ah):

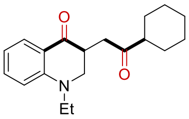

The title compound was prepared from bromocyclohexane (49.2 μL, 0.4 mmol) and *N*-allyl-*N*-ethylaniline (32.2 mg, 0.2 mmol) according to the general procedure, and the crude residue was purified by flash chromatography (pentane/EA = 10:1, R_f = 0.3) to give the product as a yellow oil (19.6 mg, 33%).

^{1}H NMR (300 MHz, $CDCl_3$) δ 7.87 (ddd, *J* = 7.9, 1.8, 0.5 Hz, 1H), 7.36 (ddd, *J* = 8.7, 7.0, 1.8 Hz, 1H), 6.76 – 6.70 (m, 1H), 6.67 (ddd, *J* = 8.0, 7.0, 1.0 Hz, 1H), 3.55 – 3.09 (m, 6H), 2.49 – 2.35 (m, 2H), 1.93 – 1.75 (m, 4H), 1.71 – 1.63 (m, 1H), 1.42 – 1.22 (m, 5H), 1.16 (t, *J* = 7.1 Hz, 3H). **^{13}C NMR** (75 MHz, $CDCl_3$) δ 212.0, 194.8, 150.9, 135.3, 128.6, 119.0, 116.3, 112.8, 52.7, 51.1, 45.5, 41.7, 37.6, 28.6, 28.4, 25.8, 25.7, 25.5, 10.9. **HRMS** (ESI) calcd for $C_{19}H_{26}NO_2^+$ [M+H$^+$]: 300.1958, Found: 300.1962.

3-(2-Cyclohexyl-2-oxoethyl)-1,6-dimethyl-2,3-dihydroquinolin-4(1*H*)-one (5ai):

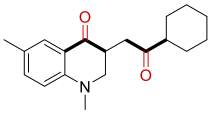

The title compound was prepared from bromocyclohexane (49.2 μL, 0.4 mmol) and *N*-allyl-*N*,4-dimethylaniline (32.2 mg, 0.2 mmol) according to the general procedure, and the crude residue was purified by flash chromatography (pentane/EA = 20:1, R_f = 0.1) to give the product as a yellow oil (24.8 mg, 41%).

^{1}H NMR (300 MHz, $CDCl_3$) δ 7.69 (dd, *J* = 1.6, 0.7 Hz, 1H), 7.22 (ddd, *J* = 8.6, 2.3, 0.7 Hz, 1H), 6.63 (d, *J* = 8.6 Hz, 1H), 3.43 – 3.34 (m, 1H), 3.31 – 3.21 (m, 1H), 3.21 – 3.08 (m, 2H), 2.94 (s, 3H), 2.48 – 2.38 (m, 2H), 2.24 (s, 3H), 1.93 – 1.85 (m, 2H), 1.82 – 1.75 (m, 2H), 1.70 – 1.64 (m, 1H), 1.42 – 1.22 (m, 5H). **^{13}C NMR** (75 MHz, $CDCl_3$) δ 212.0, 195.1, 150.8, 136.5, 127.9, 126.3, 119.0, 113.2, 56.0, 51.1, 42.3, 39.3, 37.7, 28.6, 28.4, 25.8, 25.7, 25.5, 20.0. **HRMS** (ESI) calcd for $C_{19}H_{26}NO_2^+$ [M+H$^+$]: 300.1958, Found: 300.1966.

6-(*tert*-Butyl)-3-(2-cyclohexyl-2-oxoethyl)-1-methyl-2,3-dihydroquinolin-4(1*H*)-one (5aj):

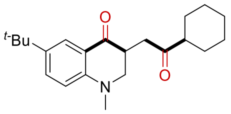

The title compound was prepared from bromocyclohexane (49.2 µL, 0.4 mmol) and *N*-allyl-4-(*tert*-butyl)-*N*-methylaniline (40.6 mg, 0.2 mmol) according to the general procedure, and the crude residue was purified by flash chromatography (pentane/EA = 20:1, R_f = 0.3) to give the product as a yellow oil (30.7 mg, 45%).

^{1}H NMR (300 MHz, $CDCl_3$) δ 7.90 (dd, *J* = 2.6, 0.4 Hz, 1H), 7.48 (dd, *J* = 8.8, 2.6 Hz, 1H), 6.69 (d, *J* = 8.8 Hz, 1H), 3.44 – 3.36 (m, 1H), 3.33 – 3.16 (m, 2H), 3.11 (dd, *J* = 17.7, 4.2 Hz, 1H), 2.96 (s, 3H), 2.50 – 2.37 (m, 2H), 1.96 – 1.86 (m, 2H), 1.83 – 1.76 (m, 2H), 1.71 – 1.64 (m, 1H), 1.44 – 1.29 (m, 5H), 1.28 (s, 9H). **^{13}C NMR** (75 MHz, $CDCl_3$) δ 212.0, 195.2, 150.6, 139.8, 133.1, 124.2, 118.6, 113.1, 55.9, 51.1, 42.3, 39.2, 37.8, 33.9, 31.2, 28.6, 28.4, 25.8, 25.7, 25.5. **HRMS** (ESI) calcd for $C_{22}H_{32}NO_2^+$ [M+H$^+$]: 342.2428, Found: 342.2435.

3-(2-Cyclohexyl-2-oxoethyl)-6-methoxy-1-methyl-2,3-dihydroquinolin-4(1*H*)-one (5ak):

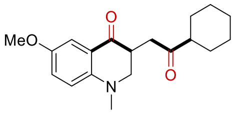

The title compound was prepared from bromocyclohexane (49.2 µL, 0.4 mmol) and N-allyl-4-methoxy-N-methylaniline (35.4 mg, 0.2 mmol) according to the general procedure, and the crude residue was purified by flash chromatography (pentane/EA = 10:1, R_f = 0.1) to give the product as a yellow oil (30.1 mg, 48%).

^{1}H NMR (300 MHz, $CDCl_3$) δ 7.38 (d, *J* = 3.1 Hz, 1H), 7.07 (dd, *J* = 9.1, 3.2 Hz, 1H), 6.70 (d, *J* = 9.2 Hz, 1H), 3.77 (s, 3H), 3.37 (dd, *J* = 10.2, 4.6 Hz, 1H), 3.32 – 3.22 (m, 1H), 3.20 – 3.06 (m, 2H), 2.94 (s, 3H), 2.51 – 2.38 (m, 2H), 1.95 – 1.86 (m, 2H), 1.84 – 1.76 (m, 2H), 1.72 – 1.64 (m, 1H), 1.42 – 1.21 (m, 5H). **^{13}C NMR** (75 MHz, $CDCl_3$) 211.9, 194.9, 151.6, 148.0, 125.0, 119.3, 115.0, 109.1, 56.3, 55.7, 51.1, 42.4, 39.6, 37.8, 28.6, 28.4, 25.8, 25.7, 25.5. **HRMS** (ESI) calcd for $C_{19}H_{26}NO_3^+$ [M+H$^+$]: 316.1907, Found: 316.1912.

3-(2-Cyclohexyl-2-oxoethyl)-6-fluoro-1-methyl-2,3-dihydroquinolin-4(1*H*)-one (5al):

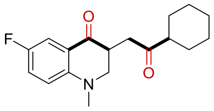

The title compound was prepared from bromocyclohexane (49.2 µL, 0.4 mmol) and *N*-allyl-4-fluoro-*N*-methylaniline (33.0 mg, 0.2 mmol) according to the general procedure, and the crude residue was purified by flash chromatography (pentane/EA = 20:1, R_f = 0.1) to give the product as a yellow oil (23.9 mg, 39%).

^{1}H NMR (300 MHz, $CDCl_3$) δ 7.48 (dd, *J* = 8.9, 3.1 Hz, 1H), 7.07 (ddd, *J* = 9.2, 7.8, 3.2 Hz, 1H), 6.60 (dd, *J* = 9.2, 4.1 Hz, 1H), 3.39 – 3.29 (m, 1H), 3.26 – 3.11 (m, 2H), 3.10 – 3.02 (m, 1H), 2.89 (s, 3H), 2.43 – 2.30 (m, 2H), 1.86 – 1.77 (m, 2H), 1.76 – 1.69 (m, 2H), 1.65 – 1.58 (m, 1H), 1.31 – 1.16 (m, 5H). **^{13}C NMR** (75 MHz, $CDCl_3$) δ 211.7, 194.3 (d, *J* = 2.0 Hz), 155.2 (d, *J* = 237.7 Hz), 149.4, 123.0 (d, *J* = 23.8 Hz), 119.5 (d, *J* = 5.7 Hz), 114.6 (d, *J* = 6.7 Hz), 113.1 (d, *J* = 22.2 Hz), 56.0, 51.1, 42.3 (d, *J* = 1.1 Hz), 39.5, 37.5, 28.6, 28.4, 25.8, 25.7, 25.5. **HRMS** (ESI) calcd for $C_{18}H_{23}FNO_2^+$ [M+H$^+$]: 304.1707, Found: 304.1713.

6-Chloro-3-(2-cyclohexyl-2-oxoethyl)-1-methyl-2,3-dihydroquinolin-4(1*H*)-one (5am):

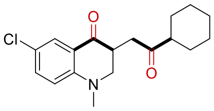

The title compound was prepared from bromocyclohexane (49.2 µL, 0.4 mmol) and *N*-allyl-4-chloro-*N*-methylaniline (36.3 mg, 0.2 mmol) according to the general procedure, and the crude residue was purified by flash chromatography (pentane/EA = 20:1, R_f = 0.1) to give the product as a yellow oil (22.5 mg, 35%).

^{1}H NMR (300 MHz, $CDCl_3$) δ 7.82 (d, *J* = 2.6 Hz, 1H), 7.32 (dd, *J* = 9.0, 2.7 Hz, 1H), 6.65 (d, *J* = 9.0 Hz, 1H), 3.46 – 3.36 (m, 1H), 3.32 – 3.20 (m, 2H), 3.19 – 3.10 (m, 1H), 2.97 (s, 3H), 2.48 – 2.37 (m, 2H), 1.95 – 1.84 (m, 2H), 1.84 – 1.75 (m, 2H), 1.71 – 1.65 (m, 1H), 1.41 – 1.26 (m, 5H). **^{13}C NMR** (75 MHz, $CDCl_3$) δ 211.7, 193.8, 150.8, 135.1, 127.5, 122.6, 119.9, 114.7, 55.6, 51.1, 42.0, 39.3, 37.5, 28.6, 28.4, 25.8, 25.7, 25.5. **HRMS** (ESI) calcd for $C_{18}H_{23}ClNO_2^+$ [M+H$^+$]: 320.1412, Found: 320.1413.

6-Bromo-3-(2-cyclohexyl-2-oxoethyl)-1-methyl-2,3-dihydroquinolin-4(1*H*)-one (5an):

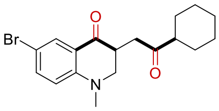

The title compound was prepared from bromocyclohexane (49.2 µL, 0.4 mmol) and *N*-allyl-4-bromo-*N*-methylaniline (45.2 mg, 0.2 mmol) according to the general procedure, and the crude residue was purified by flash chromatography (pentane/EA = 10:1, R_f = 0.2) to give the product as a yellow oil (22.7 mg, 31%).

^{1}H NMR (300 MHz, $CDCl_3$) δ 7.95 (d, *J* = 2.5 Hz, 1H), 7.43 (dd, *J* = 9.0, 2.5 Hz, 1H), 6.58 (d, *J* = 9.0 Hz, 1H), 3.46 – 3.38 (m, 1H), 3.27 – 3.19 (m, 2H), 3.17 – 3.08 (m, 1H), 2.96 (s, 3H), 2.47 – 2.36 (m, 2H), 1.93 – 1.84 (m, 2H), 1.82 – 1.75 (m, 2H), 1.71 – 1.63 (m, 1H), 1.41 – 1.22 (m, 5H). **^{13}C NMR** (75 MHz, $CDCl_3$) δ 211.7, 193.6, 151.1, 137.8, 130.5, 120.3, 115.0, 109.5, 55.5, 51.0, 41.9, 39.2, 37.5, 28.5, 28.4, 25.8, 25.6, 25.5. **HRMS** (ESI) calcd for $C_{18}H_{23}BrNO_2^+$ [M+H$^+$]: 364.0907, Found: 364.0912.

3-(2-Cyclohexyl-2-oxoethyl)-1-methyl-6-(trifluoromethyl)-2,3-dihydroquinolin-4(1*H*)-one (5ao):

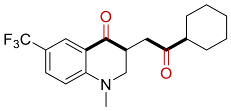

The title compound was prepared from bromocyclohexane (49.2 µL, 0.4 mmol) and *N*-allyl-*N*-methyl-4-(trifluoromethyl)aniline (43.0 mg, 0.2 mmol) according to the general procedure, and the crude residue was purified by flash chromatography (pentane/EA = 20:1, R_f = 0.1) to give the product as a yellow oil (25.3 mg, 36%).

^{1}H NMR (300 MHz, $CDCl_3$) δ 8.13 (dd, *J* = 2.3, 1.0 Hz, 1H), 7.60 – 7.53 (m, 1H), 6.75 (d, *J* = 8.9 Hz, 1H), 3.54 – 3.44 (m, 1H), 3.39 – 3.24 (m, 2H), 3.21 – 3.12 (m, 1H), 3.05 (s, 3H), 2.49 – 2.38 (m, 2H), 1.94 – 1.76 (m, 4H), 1.72 – 1.65 (m, 1H), 1.37 – 1.24 (m, 5H). **^{13}C NMR** (101 MHz, $CDCl_3$) 211.6, 153.6, 131.5, 131.5, 125.95 (q, *J* = 4.0 Hz), 124.36 (d, *J* = 270.9 Hz), 118.81 (q, *J* = 33.4 Hz), 118.1, 113.2, 55.2, 51.1, 41.7, 39.1, 37.3, 28.5, 28.4, 25.8, 25.6, 25.5. **^{19}F NMR** (282 MHz, $CDCl_3$) δ -61.7. **HRMS** (ESI) calcd for $C_{19}H_{23}F_3NO_2^+$ [M+H$^+$]: 354.1675, Found: 354.1681.

6. Derivatization of 1,4-diketones

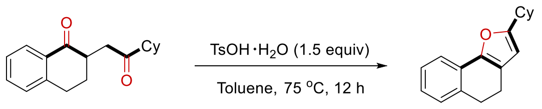

3aa **6a**, 76%

To a solution of 3aa (52.1 mg, 0.19 mmol) in toluene (2 mL) was added TsOH•H_2O (55.0 mg, 1.5 equiv). The reaction mixture was stirred at 75 °C for 12 h. After the reaction was completed, the mixture was concentrated under reduced pressure. The crude product was further purified by flash column chromatograph (pentane/EA = 20:1, R_f = 0.7) to give 2-cyclohexyl-4,5-dihydronaphtho[1,2-*b*]furan **6a** as a colorless oil (37.1 mg, 76%).

2-Cyclohexyl-4,5-dihydronaphtho[1,2-*b*]furan (6a):

^{1}H NMR (300 MHz, $CDCl_3$) δ 7.43 (dd, *J* = 7.6, 1.4 Hz, 1H), 7.25 – 7.14 (m, 2H), 7.07 (td, *J* = 7.4, 1.4 Hz, 1H), 5.94 (d, *J* = 0.9 Hz, 1H), 2.95 (t, *J* = 7.9 Hz, 2H), 2.74 – 2.64 (m, 3H), 2.14 – 2.03 (m, 2H), 1.89 – 1.79 (m, 2H), 1.78 – 1.70 (m, 1H), 1.47 – 1.37 (m, 3H), 1.35 – 1.27 (m, 2H). **^{13}C NMR** (75 MHz, $CDCl_3$) δ 160.9, 147.9, 134.1, 128.5, 127.7, 126.5, 125.6, 119.9, 118.5, 104.3, 37.5, 31.7, 29.2, 26.1, 26.0, 21.1. **HRMS** (ESI) calcd for $C_{18}H_{21}O^+$ [M+H$^+$]: 253.1587, Found: 253.1591.

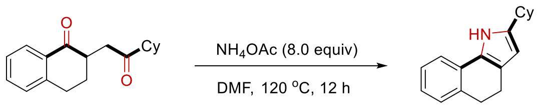

3aa **6b**, 72%

To a solution of 3aa (81.0 mg, 0.3 mmol) in DMF (2 mL) was added NH_4OAc (185 mg, 8.0 equiv). The reaction mixture was stirred at 120 °C for 12 h. After the reaction was completed, the mixture was quenched with H_2O and extracted with EA. The combined organic layer was dried over Na_2SO_4, filtered and concentrated under reduced pressure. The crude product was further purified by flash column chromatograph (pentane/EA = 50:1, R_f = 0.2) to give 2-cyclohexyl-4,5-dihydro-1*H*-benzo[*g*]indole **6b** as a yellow oil (54.7 mg, 72%).

2-Cyclohexyl-4,5-dihydro-1*H*-benzo[*g*]indole (6b):

^{1}H NMR (300 MHz, $CDCl_3$) δ 8.14 – 7.90 (br s, 1H), 7.23 – 7.16 (m, 2H), 7.16 – 7.10 (m, 1H), 7.07 – 7.00 (m, 1H), 5.87 (d, *J* = 2.4 Hz, 1H), 2.95 (dd, *J* = 8.6, 6.6 Hz, 2H), 2.80 – 2.71 (m, 2H), 2.68 – 2.58 (m, 1H), 2.12 – 2.01 (m, 2H), 1.92 – 1.83 (m, 2H), 1.82 – 1.74 (m, 1H), 1.50 – 1.39 (m, 4H), 1.37 – 1.31 (m, 1H). **^{13}C NMR** (75 MHz, $CDCl_3$) δ 139.2, 134.3, 129.5, 128.2, 126.3, 125.9, 124.3, 120.3, 117.5, 103.3, 37.1, 33.3, 30.0, 26.3, 26.1, 21.9. **HRMS** (ESI) calcd for $C_{18}H_{22}N^+$ [M+H$^+$]: 252.1747, Found: 252.1744.

7. Mechanism and competition studies

7.1 Carbon labeling experiment

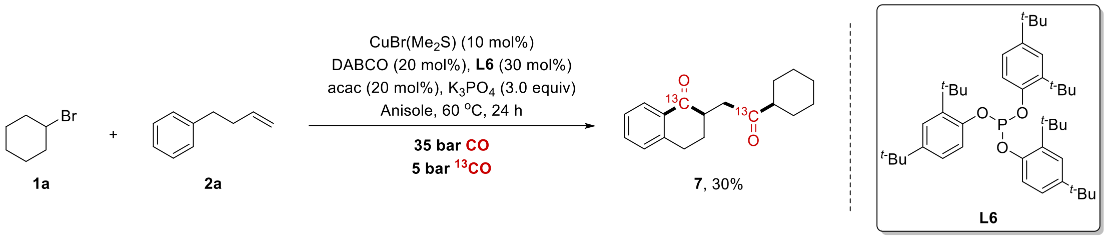

Scheme S1 Carbon labeling experiment

A 4 mL screw-cap vial was charged with CuBr(Me$_2$S) (4.1 mg, 10 mol%), DABCO (4.4 mg, 20 mol%), tris(2,4-di-*tert*-butylphenyl) phosphite (38.8 mg, 30 mol%), K$_3$PO$_4$ (127.4 mg, 0.6 mmol, 3.0 equiv) and an oven-dried stirring bar. The vial was closed by Teflon septum and phenolic cap and connected to atmosphere with a needle. After flashed the vials three times with argon and vacuum, dry anisole (1.5 mL), bromocyclohexane (49.2 µL, 0.4 mmol), but-3-en-1-ylbenzene (30 µL, 0.2 mmol) and acac (4.1 µL, 20 mol%) were injected by syringe. The vial was fixed in an alloy plate and put into Parr 4560 series autoclave (500 mL). At room temperature, the autoclave was flushed three times with CO, then 5 bar ^{13}CO and 35 bar of CO was charged. The autoclave was reacted at 60 °C for 24 h. Afterwards, the autoclave was cooled to room temperature and the pressure was carefully released. The crude product was purified by silica gel chromatography (pentane/EA) to afford the corresponding products **7**.

2-(2-Cyclohexyl-2-oxoethyl-2-^{13}C)-3,4-dihydronaphthalen-1(2*H*)-one-1-^{13}C (7):

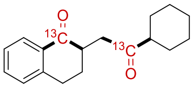

The title compound was prepared from bromocyclohexane (49.2 µL, 0.4 mmol) and but-3-en-1-ylbenzene (30 µL, 0.2 mmol) according to the general procedure, and the crude residue was purified by flash chromatography (pentane/EA = 20:1, R$_f$ = 0.3) to give the product as a colorless oil (16.1 mg, 30%).

^{1}H NMR (300 MHz, CDCl$_3$) δ 8.03 – 7.94 (m, 1H), 7.45 (td, *J* = 7.4, 1.5 Hz, 1H), 7.31 – 7.20 (m, 2H), 3.28 – 3.04 (m, 3H), 2.98 – 2.86 (m, 1H), 2.53 – 2.37 (m, 2H), 2.22 – 2.09 (m, 1H), 2.01 – 1.84 (m, 3H), 1.83 – 1.74 (m, 2H), 1.72 – 1.63 (m, 1H), 1.43 – 1.21 (m, 5H). **^{13}C NMR** (75 MHz, CDCl$_3$) δ 212.3 (^{13}C=O), 199.1 (^{13}C=O), 144.0, 133.2, 132.2, 128.7, 127.3, 126.5, 51.1, 44.0, 41.0, 29.5, 29.3, 28.6, 28.4, 25.8, 25.7, 25.6. **HRMS** (ESI) calcd for $C_{16}{}^{13}C_2H_{23}O_2^+$ [M+H$^+$]: 273.1760, Found: 273.1764.

7.2 Radical capture experiment

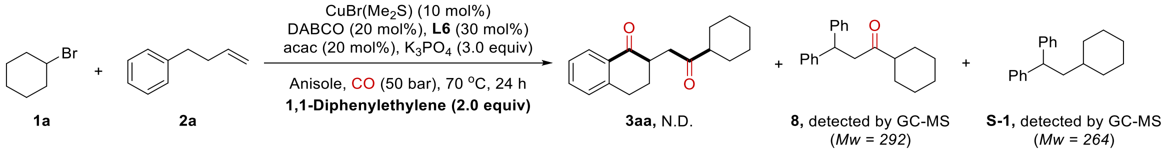

Scheme S2 Radical capture experiments

A 4 mL screw-cap vial was charged with CuBr(Me_2S) (4.1 mg, 10 mol%), DABCO (4.4 mg, 20 mol%), tris(2,4-di-*tert*-butylphenyl) phosphite (38.8 mg, 30 mol%), K_3PO_4 (127.4 mg, 0.6 mmol, 3.0 equiv) and an oven-dried stirring bar. The vial was closed by Teflon septum and phenolic cap and connected to atmosphere with a needle. After flashed the vials three times with argon and vacuum, dry anisole (1.5 mL), bromocyclohexane (49.2 µL, 0.4 mmol), but-3-en-1-ylbenzene (30 µL, 0.2 mmol), acac (4.1 µL, 20 mol%) and 1,1-diphenylethylene (70.6 µL, 0.4 mmol, 2.0 equiv) were injected by syringe. The vial was fixed in an alloy plate and put into Parr 4560 series autoclave (500 mL). At room temperature, the autoclave was flushed three times with CO and 50 bar of CO was charged. The autoclave was reacted at 70 °C for 24 h. Afterwards, the autoclave was cooled to room temperature and the pressure was carefully released. Then, a proper amount of solvent was taken for GC-MS analysis. The result is shown in **Figure S1**.

When 1,1-diphenylethylene were added to the reaction, no products were detected, and alkyl radical and acyl radicals were trapped by 1,1-diphenylethylene.

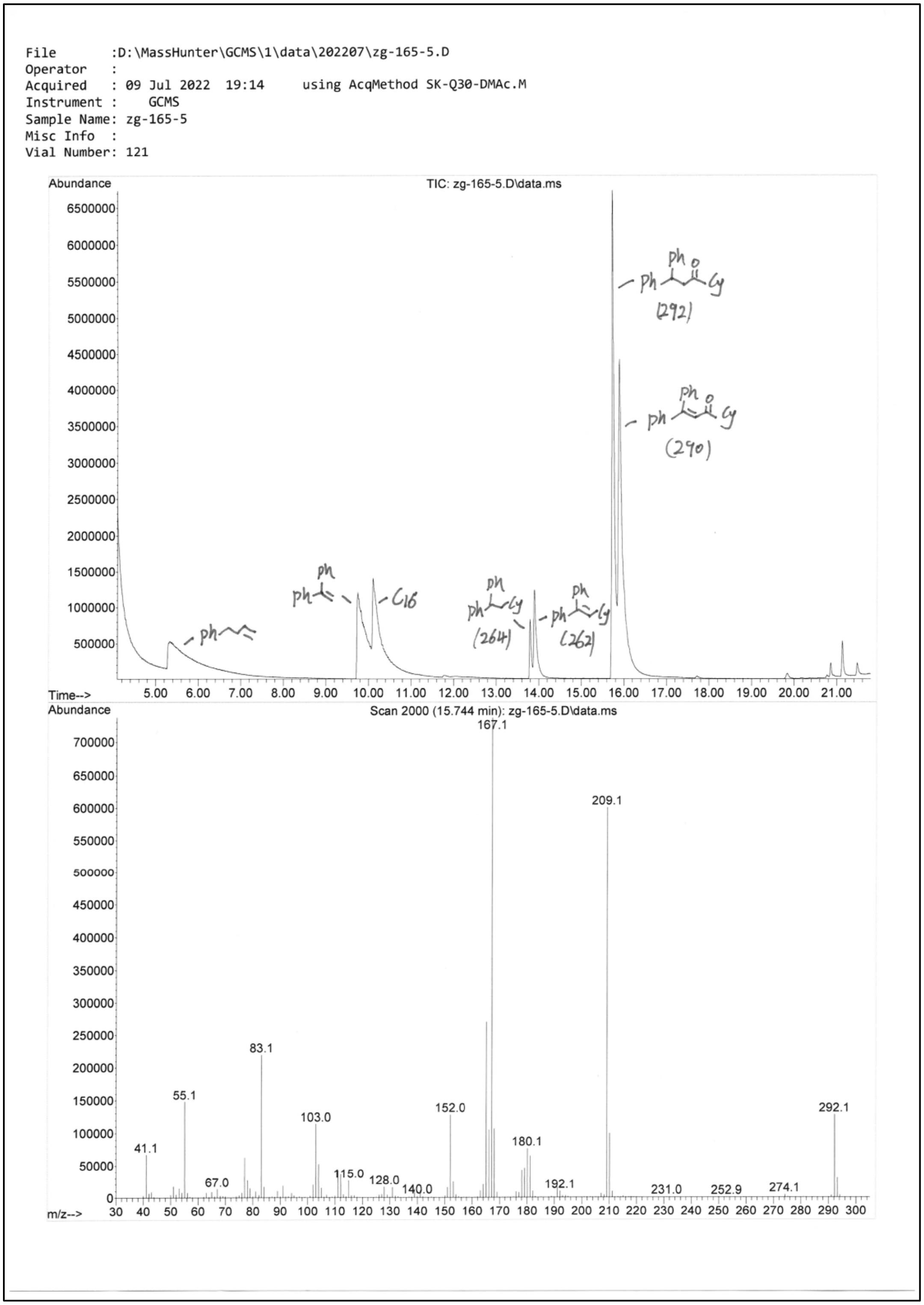

Figure S1 GC-MC of the reaction solvent

7.3 Radical inhibition experiments

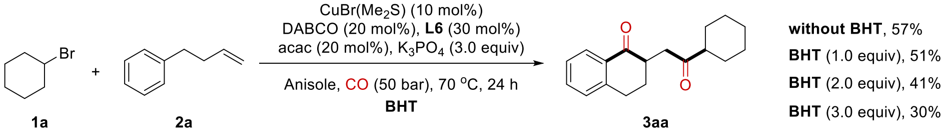

Scheme S3 Radical inhibition experiments

A 4 mL screw-cap vial was charged with CuBr(Me_2S) (4.1 mg, 10 mol%), DABCO (4.4 mg, 20 mol%), tris(2,4-di-*tert*-butylphenyl) phosphite (38.8 mg, 30 mol%), K_3PO_4 (127.4 mg, 0.6 mmol, 3.0 equiv) and an oven-dried stirring bar. Different amounts of 2,6-di-*tert*-butyl-4-methylphenol (BHT) (1 – 3 equiv) were added sequentially to the corresponding vials. The vial was closed by Teflon septum and phenolic cap and connected to atmosphere with a needle. After flashed the vials three times with argon and vacuum, dry anisole (1.5 mL), bromocyclohexane (49.2 μL, 0.4 mmol), but-3-en-1-ylbenzene (30 μL, 0.2 mmol) and acac (4.1 μL, 20 mol%) were injected by syringe. The vial was fixed in an alloy plate and put into Parr 4560 series autoclave (500 mL). At room temperature, the autoclave was flushed three times with CO and 50 bar of CO was charged. The autoclave was reacted at 70 °C for 24 h. Afterwards, the autoclave was cooled to room temperature and the pressure was carefully released. Then, 20 μL of hexadecane was added to the vials. And a proper amount of solvent was taken for GC analysis. The result is shown above.

As BHT was added to the reaction system, the reaction was gradually inhibited.

7.4 Competition experiments

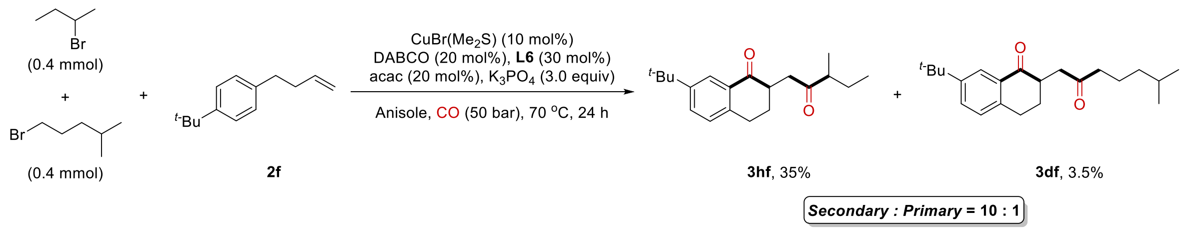

Scheme S4 Competition between secondary alkyl bromides and primary alkyl bromides

A 4 mL screw-cap vial was charged with $CuBr(Me_2S)$ (4.1 mg, 10 mol%), DABCO (4.4 mg, 20 mol%), tris(2,4-di-*tert*-butylphenyl) phosphite (38.8 mg, 30 mol%), K_3PO_4 (127.4 mg, 0.6 mmol, 3.0 equiv) and an oven-dried stirring bar. The vial was closed by Teflon septum and phenolic cap and connected to atmosphere with a needle. After flashed the vials three times with argon and vacuum, dry anisole (1.5 mL), 2-bromobutane (43.7 µL, 0.4 mmol), 1-bromo-4-methylpentane (58.2 µL, 0.4 mmol), 1-(but-3-en-1-yl)-4-(*tert*-butyl)benzene (37.6 mg, 0.2 mmol) and acac (4.1 µL, 20 mol%) were injected by syringe. The vial was fixed in an alloy plate and put into Parr 4560 series autoclave (500 mL). At room temperature, the autoclave was flushed three times with CO and 50 bar of CO was charged. The autoclave was reacted at 70 °C for 24 h. Afterwards, the autoclave was cooled to room temperature and the pressure was carefully released. The crude residue was purified by silica gel chromatography (pentane/EA = 20:1, R_f = 0.4) to give a mixture of **3hf** and **3df** (23.5 mg). The ratio was confirmed by NMR (**3hf** : **3df** = 10 : 1).

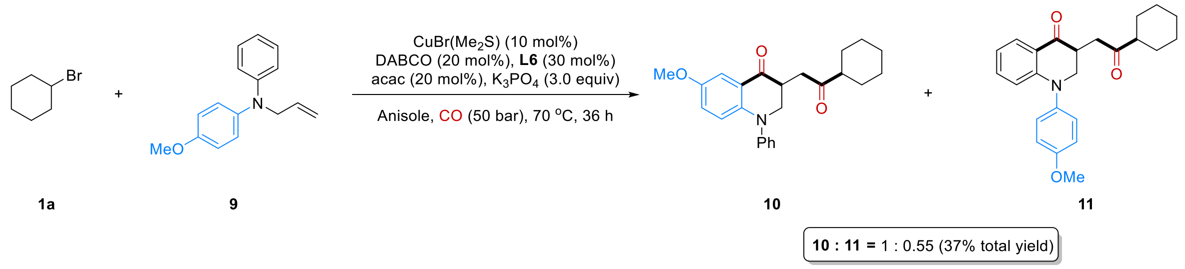

Scheme S5 Cyclization selectivity between different aromatic rings

A 4 mL screw-cap vial was charged with $CuBr(Me_2S)$ (4.1 mg, 10 mol%), DABCO (4.4 mg, 20 mol%), tris(2,4-di-*tert*-butylphenyl) phosphite (38.8 mg, 30 mol%), K_3PO_4 (127.4 mg, 0.6 mmol, 3.0 equiv) and an oven-dried stirring bar. The vial was closed by Teflon septum and phenolic cap and connected to atmosphere with a needle. After flashed the vials three times with argon and vacuum, dry anisole (1.5 mL), bromocyclohexane (49.2 µL, 0.4 mmol), *N*-allyl-4-methoxy-*N*-phenylaniline (47.8 mg, 0.2 mmol) and acac (4.1 µL, 20 mol%) were injected by syringe. The vial was fixed in an alloy plate and put into Parr 4560 series autoclave (500 mL). At room temperature, the autoclave was flushed three times with CO and 50 bar of CO was charged. The autoclave was reacted at 70 °C for 36 h. Afterwards, the autoclave was cooled to room temperature and the pressure was carefully released. The crude residue was purified by silica gel chromatography (pentane/EA = 10:1, R_f = 0.4) to give a mixture of **10** and **11** (27.6 mg, 37%). The ratio was confirmed by NMR (**10** : **11** = 1 : 0.55).

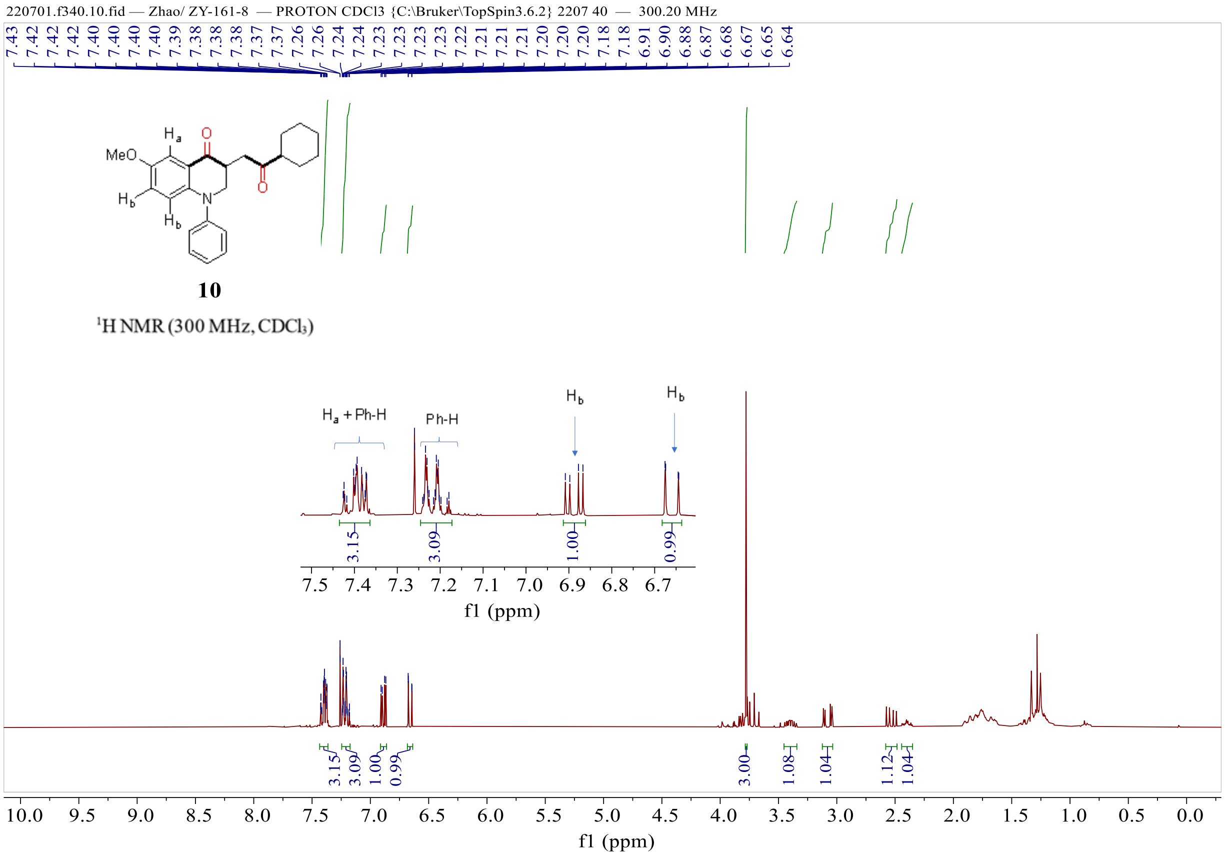

Figure S2 NMR Spectra of **10**

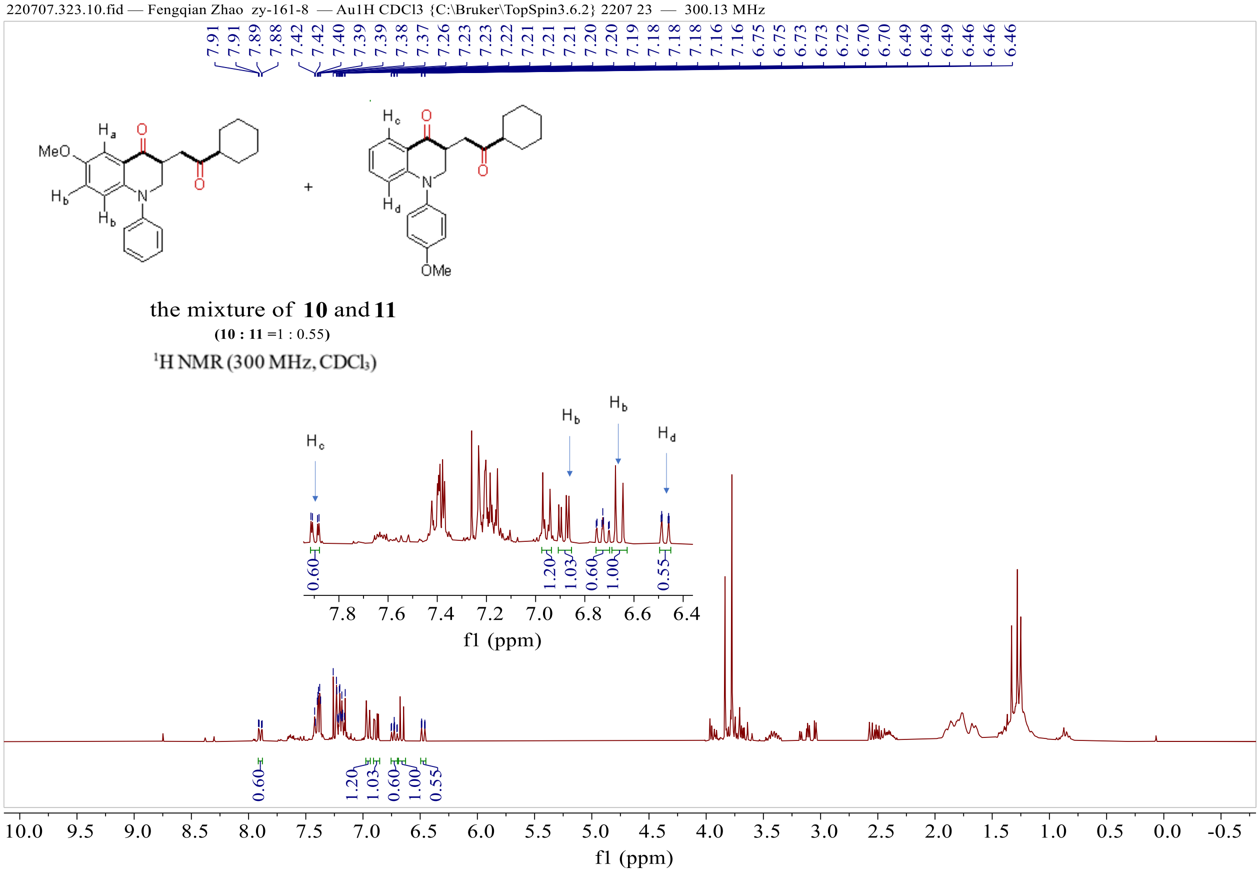

Figure S3 NMR Spectra of a mixture of **10** and **11**

8. Reference:

1. a) H. Zhao, A. J. McMillan, T. Constantin, R. C. Mykura, F. Juliá, D. Leonori, *J. Am. Chem. Soc.* **2021**, *143*, 14806-14813; b) M. D. Greenhalgh, S. P. Thomas, *Chem. Commun.* **2013**, *49*, 11230-11232; c) S. K. De, R. A. Gibbs, *Tetrahedron Lett.* **2005**, *46*, 8345-8350; d) A. Theodorou, D. Limnios, C. G. Kokotos, *Chem. Eur. J.* **2015**, *21*, 5238-5241.
2. J. L. Nallasivam, R. A. Fernandes, *Eur. J. Org. Chem.* **2015**, 2012-2022.
3. J. Richers, A. Pöthig, E. Herdtweck, C. Sippel, F. Hausch, K. Tiefenbacher, *Chem. Eur. J.* **2017**, *23*, 3178-3183.
4. M. Kanehara, Y. Oumi, T. Sano, T. Teranishi, *Bull. Chem. Soc. Jpn.* **2004**, *77*, 1589-1597.
5. Q. Meng, T. E. Schirmer, K. Katou, B. König, *Angew. Chem. Int. Ed.* **2019**, *58*, 5723-5728.
6. A. Kapat, T. Sperger, S. Guven, F. Schoenebeck, *Science*, **2019**, *363*, 391-396.
7. S. Datta, C.-L. Chang, K.-L. Yeh, R.-S. Liu, *J. Am. Chem. Soc.* **2003**, *125*, 9294-9295.
8. Y, Yoshizawa, S, Kawaii, M, Kanauchi, M, Chida, J. Mizutani, *Biosci Biotechnol Biochem.* **2008**, *57*, 1572-1574.
9. I. Solic, P. Seankongsuk, J. K. Loh, T. Vilaivan, R. W. Bates, *Org. Biomol. Chem.* **2018**, *16*, 119-123.
10. L. J. Brown, R. C. D. Brown, R. Raja, *RSC Adv.* **2013**, *3*, 843-850.
11. S. B. Lang, K. M. O’Nele, J. A. Tunge, *J. Am. Chem. Soc.* **2014**, *136*, 13606-13609.
12. J. C. Antilla, S. L. Buchwald, *Org. Lett.* **2001**, *3*, 2077-2079.
13. S. S. Smile, M. Novanna, S. Kannadasan, P. Shanmugam, *RSC Advances* **2022**, *12*, 1834-1839.
14. X. Kong, H. Zhang, Y. Xiao, C. Cao, Y. Shi, G. Pang, *RSC Adv.* **2015**, *5*, 7035-7048.

9. NMR Spectra of products: ^{1}H, ^{13}C and ^{19}F NMR

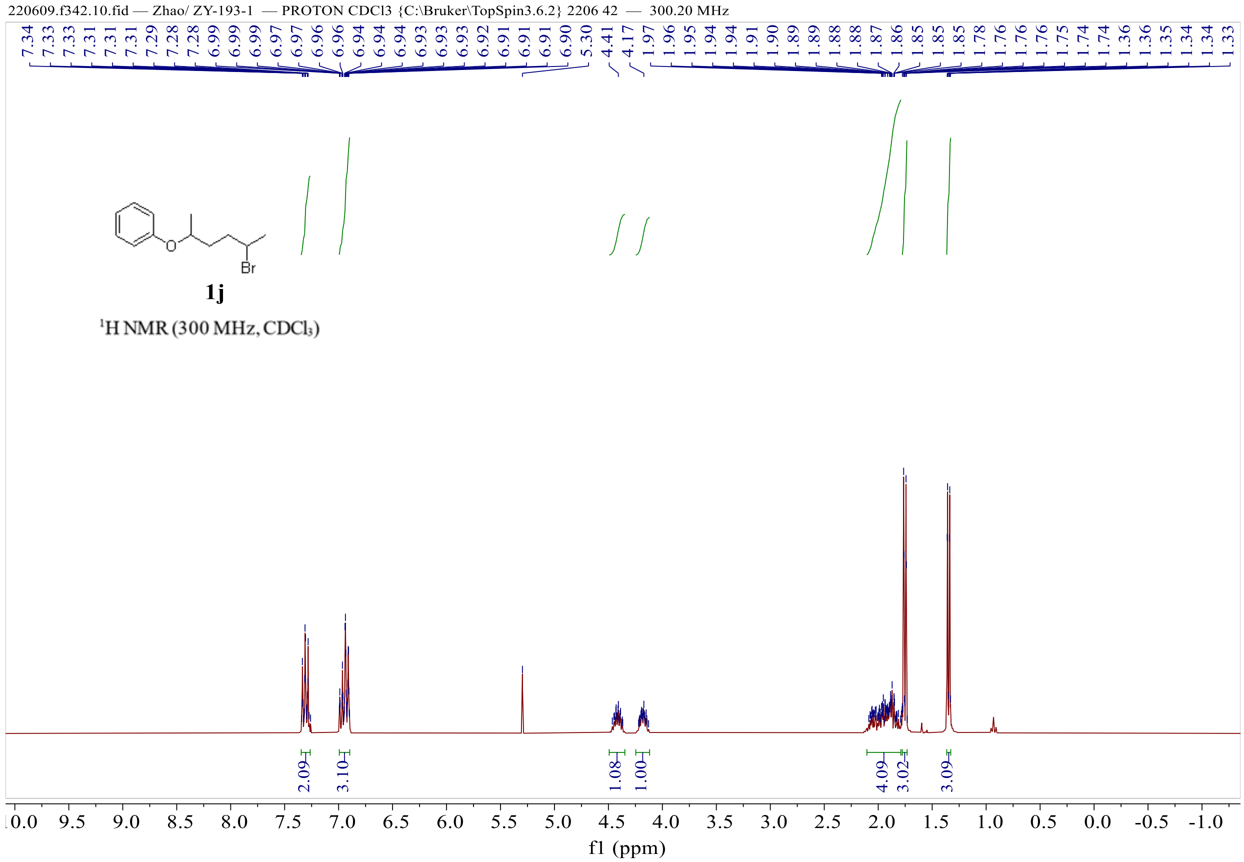

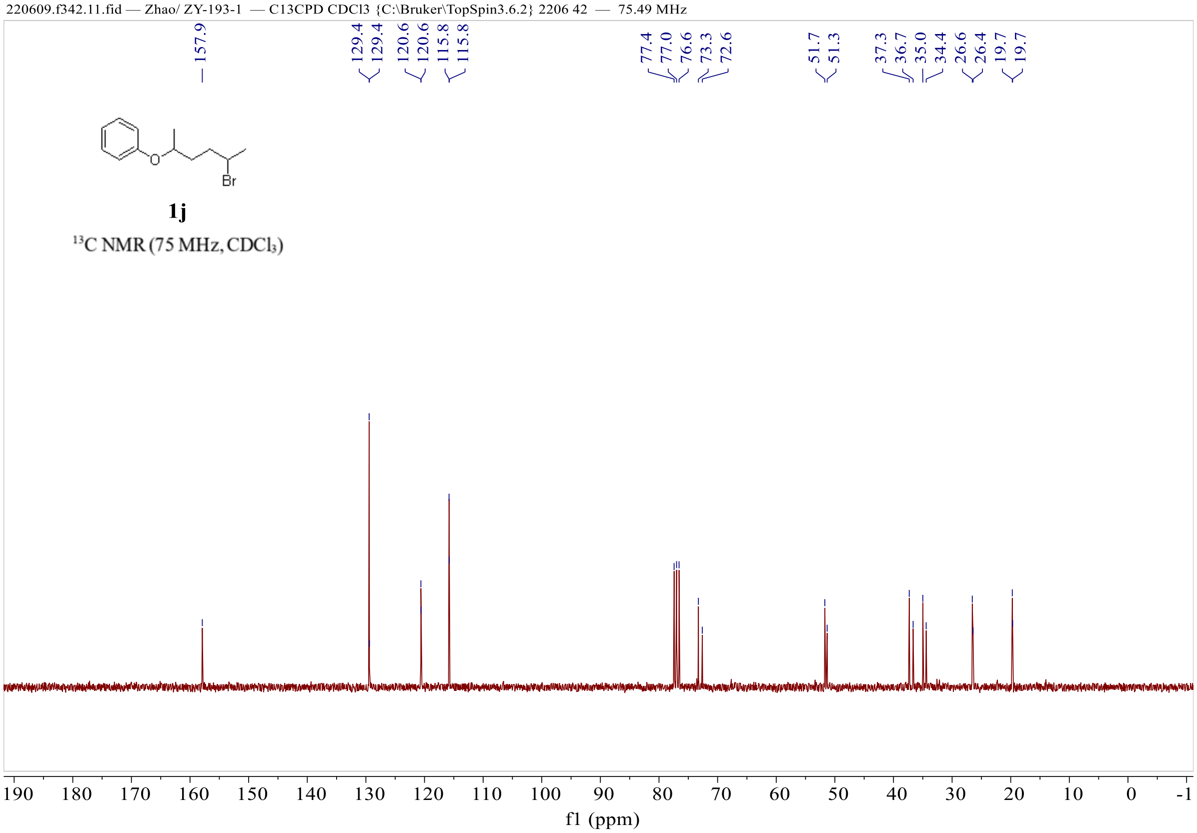

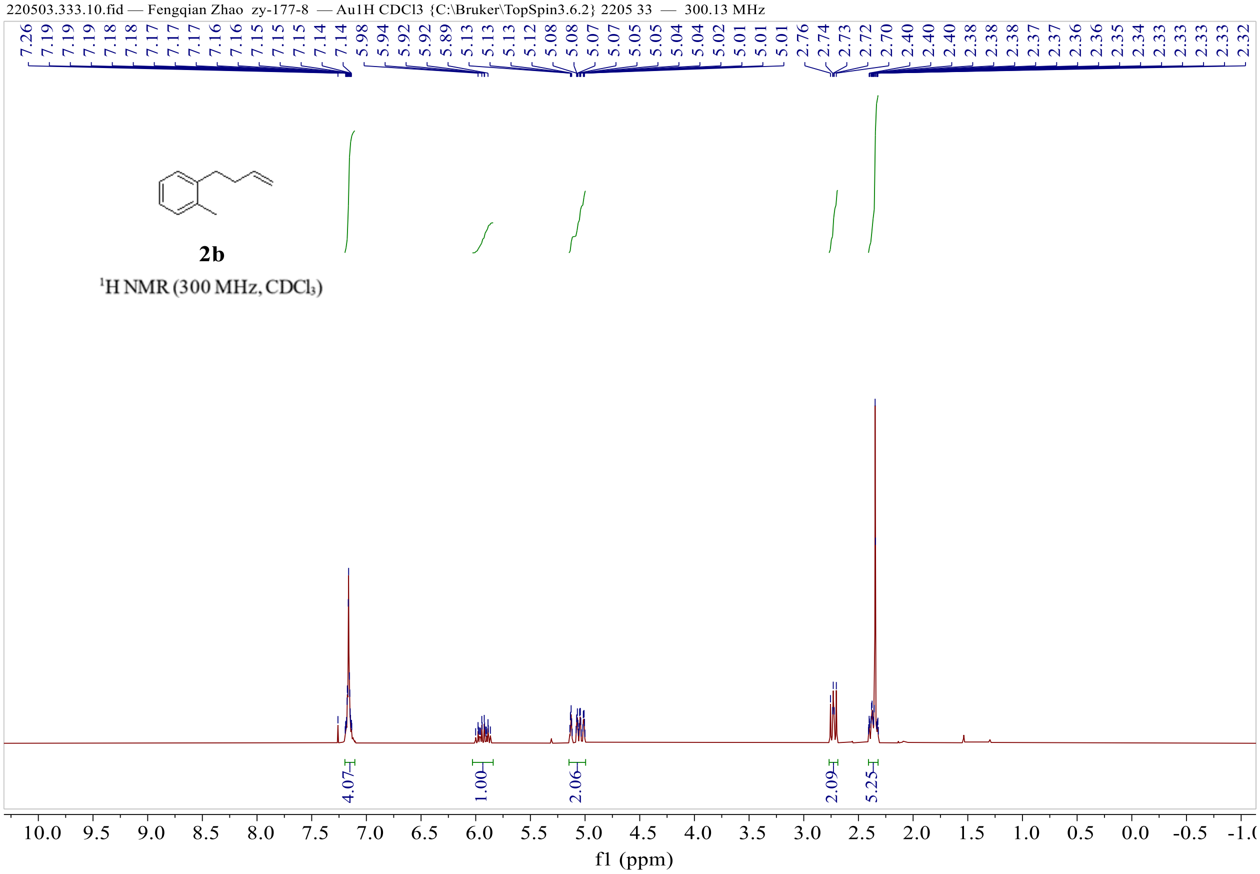
220503.333.10.fid — Fengqian Zhao zy-177-8 — Au1H CDCl3 {C:\Bruker\TopSpin3.6.2} 2205 33 — 300.13 MHz
7.26 7.19 7.19 7.19 7.18 7.18 7.17 7.17 7.17 7.16 7.16 7.15 7.15 7.15 7.14 7.14 5.98 5.94 5.92 5.92 5.89 5.13 5.13 5.13 5.12 5.08 5.08 5.07 5.07 5.05 5.05 5.04 5.04 5.02 5.01 5.01 5.01 2.76 2.74 2.73 2.72 2.70 2.40 2.40 2.40 2.38 2.38 2.38 2.37 2.37 2.36 2.35 2.34 2.33 2.33 2.33 2.33 2.32
2b
¹H NMR (300 MHz, CDCl₃)
4.07
1.00
2.06
2.09
5.25
10.0 9.5 9.0 8.5 8.0 7.5 7.0 6.5 6.0 5.5 5.0 4.5 4.0 3.5 3.0 2.5 2.0 1.5 1.0 0.5 0.0 -0.5 -1.0
f1 (ppm)

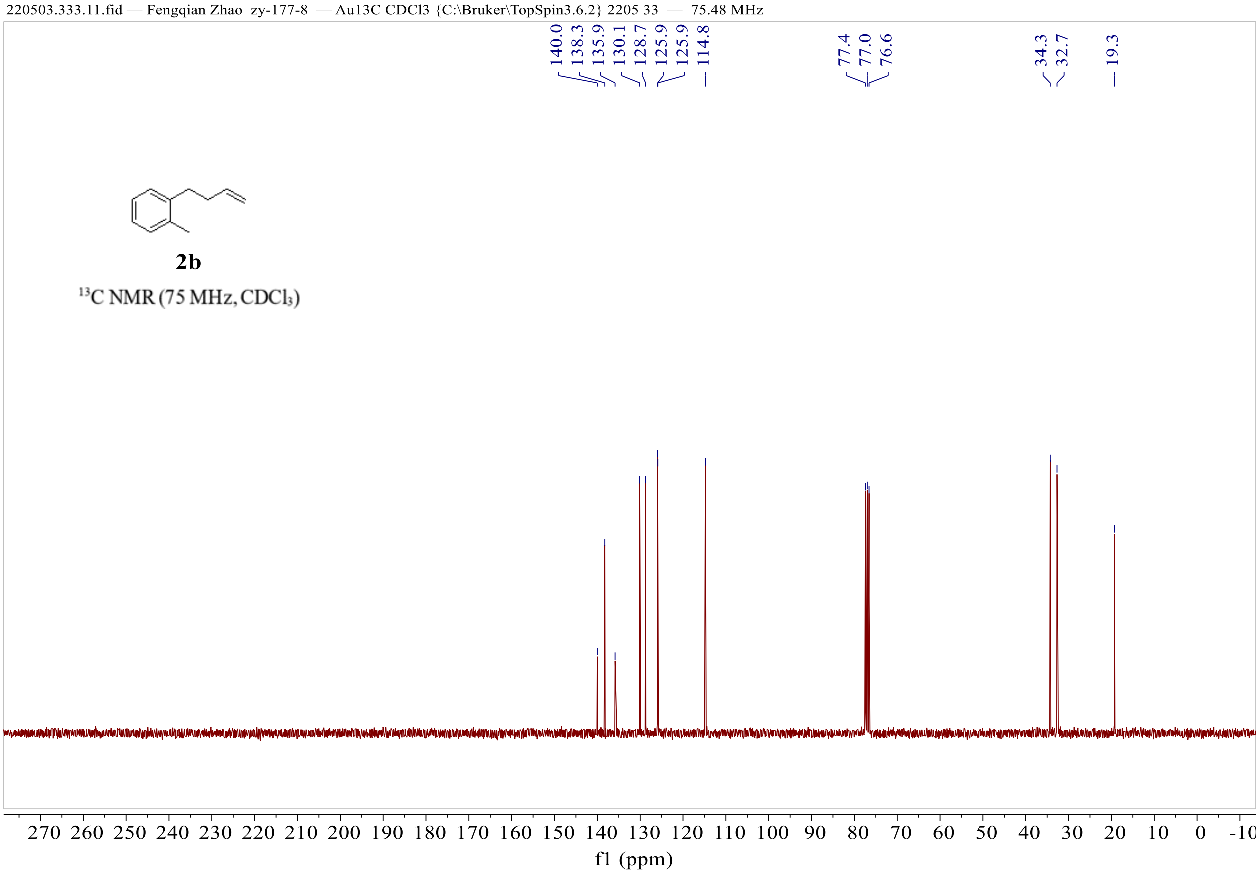
220503.333.11.fid — Fengqian Zhao zy-177-8 — Au13C CDCl3 {C:\Bruker\TopSpin3.6.2} 2205 33 — 75.48 MHz
140.0 138.3 135.9 130.1 128.7 125.9 125.9 114.8
77.4 77.0 76.6
34.3 32.7
19.3
2b
¹³C NMR (75 MHz, CDCl₃)
270 260 250 240 230 220 210 200 190 180 170 160 150 140 130 120 110 100 90 80 70 60 50 40 30 20 10 0 -10
f1 (ppm)

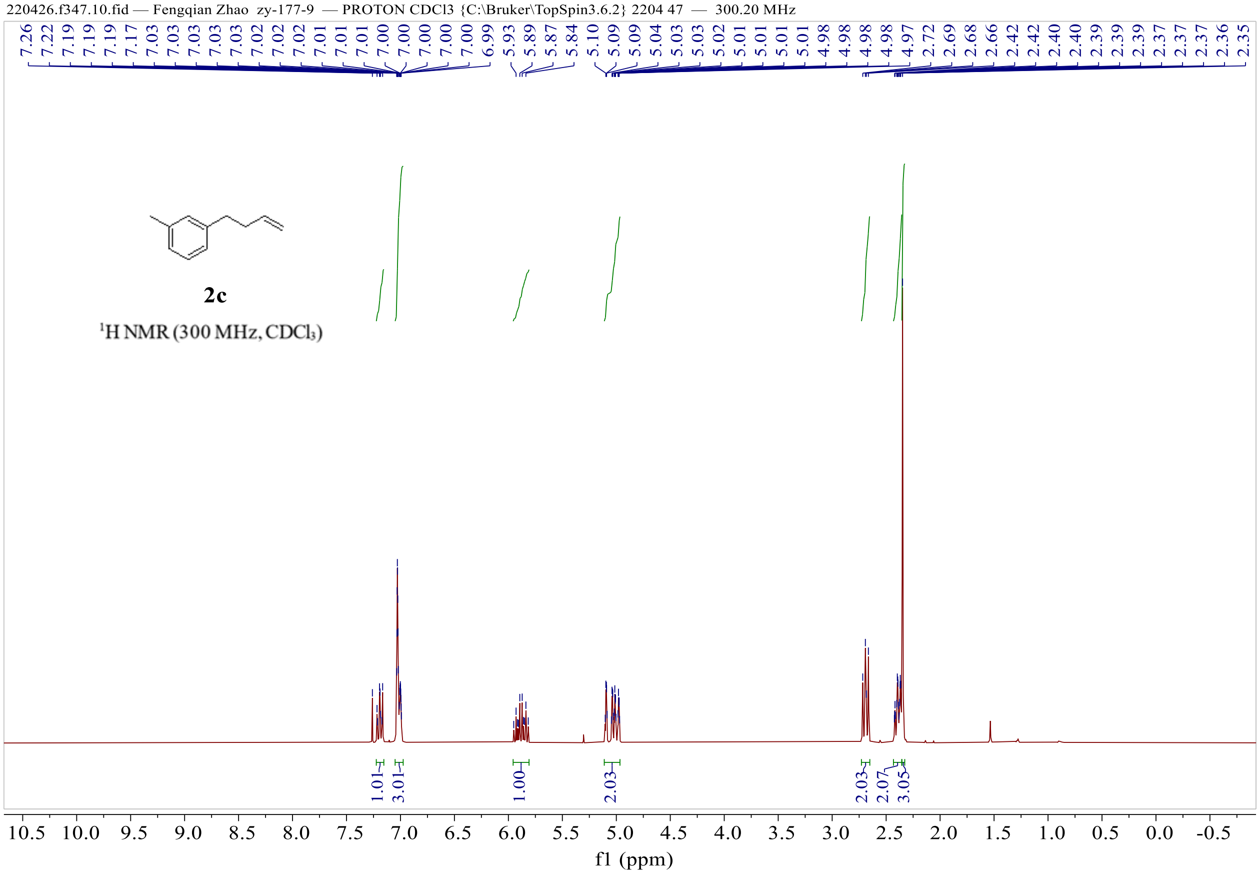
220426.f347.10.fid — Fengqian Zhao zy-177-9 — PROTON CDCl3 {C:\Bruker\TopSpin3.6.2} 2204 47 — 300.20 MHz
7.26 7.22 7.19 7.19 7.17 7.03 7.03 7.03 7.03 7.03 7.02 7.02 7.02 7.01 7.01 7.01 7.00 7.00 7.00 7.00 7.00 6.99 5.93 5.89 5.87 5.84 5.10 5.09 5.09 5.04 5.03 5.03 5.02 5.01 5.01 5.01 5.01 4.98 4.98 4.98 4.97 2.72 2.69 2.68 2.66 2.42 2.42 2.40 2.40 2.39 2.39 2.39 2.37 2.37 2.37 2.36 2.35
2c
1H NMR (300 MHz, CDCl3)
1.01 3.01 1.00 2.03 2.03 2.07 3.05
f1 (ppm)

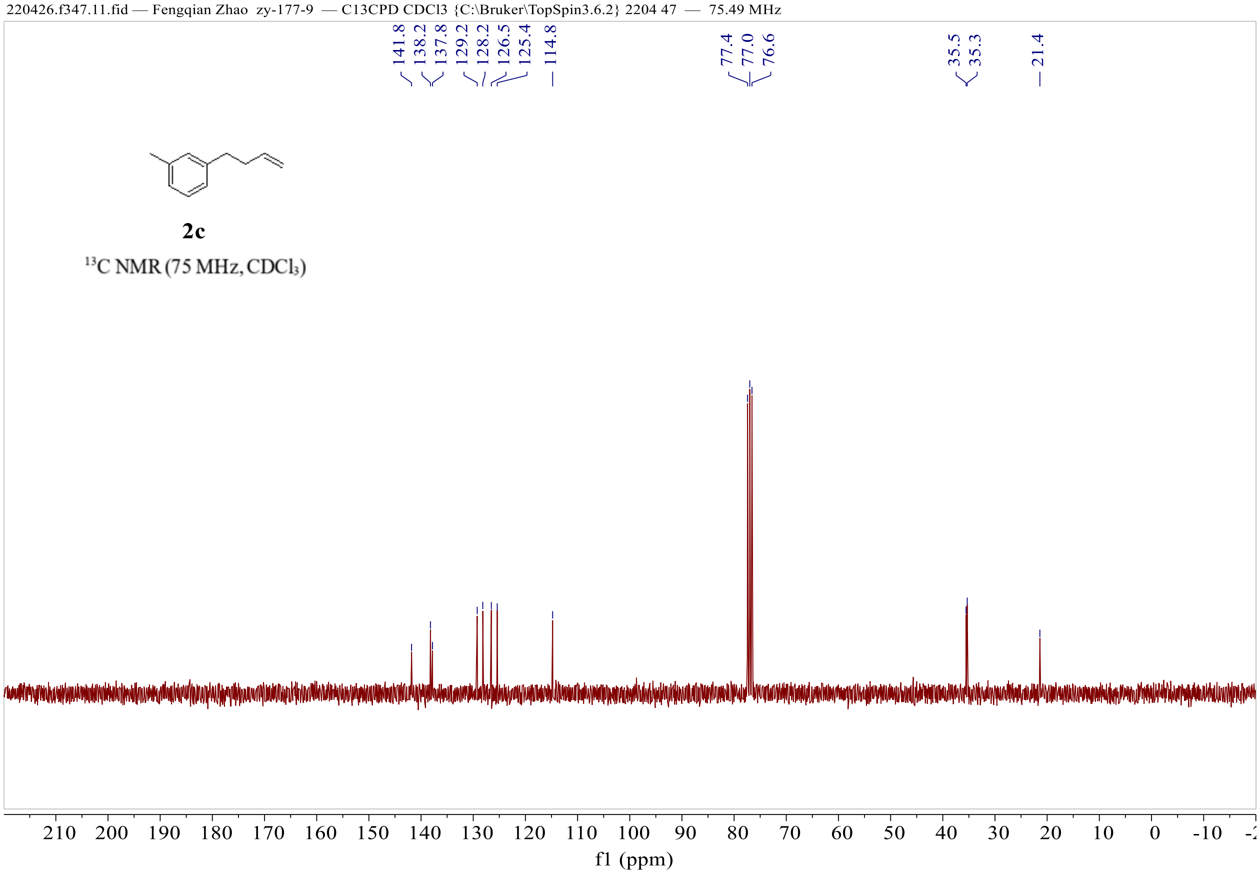
220426.f347.11.fid — Fengqian Zhao zy-177-9 — C13CPD CDCl3 {C:\Bruker\TopSpin3.6.2} 2204 47 — 75.49 MHz
141.8 138.2 137.8 129.2 128.2 126.5 125.4 114.8 77.4 77.0 76.6 35.5 35.3 21.4
2c
13C NMR (75 MHz, CDCl3)
f1 (ppm)

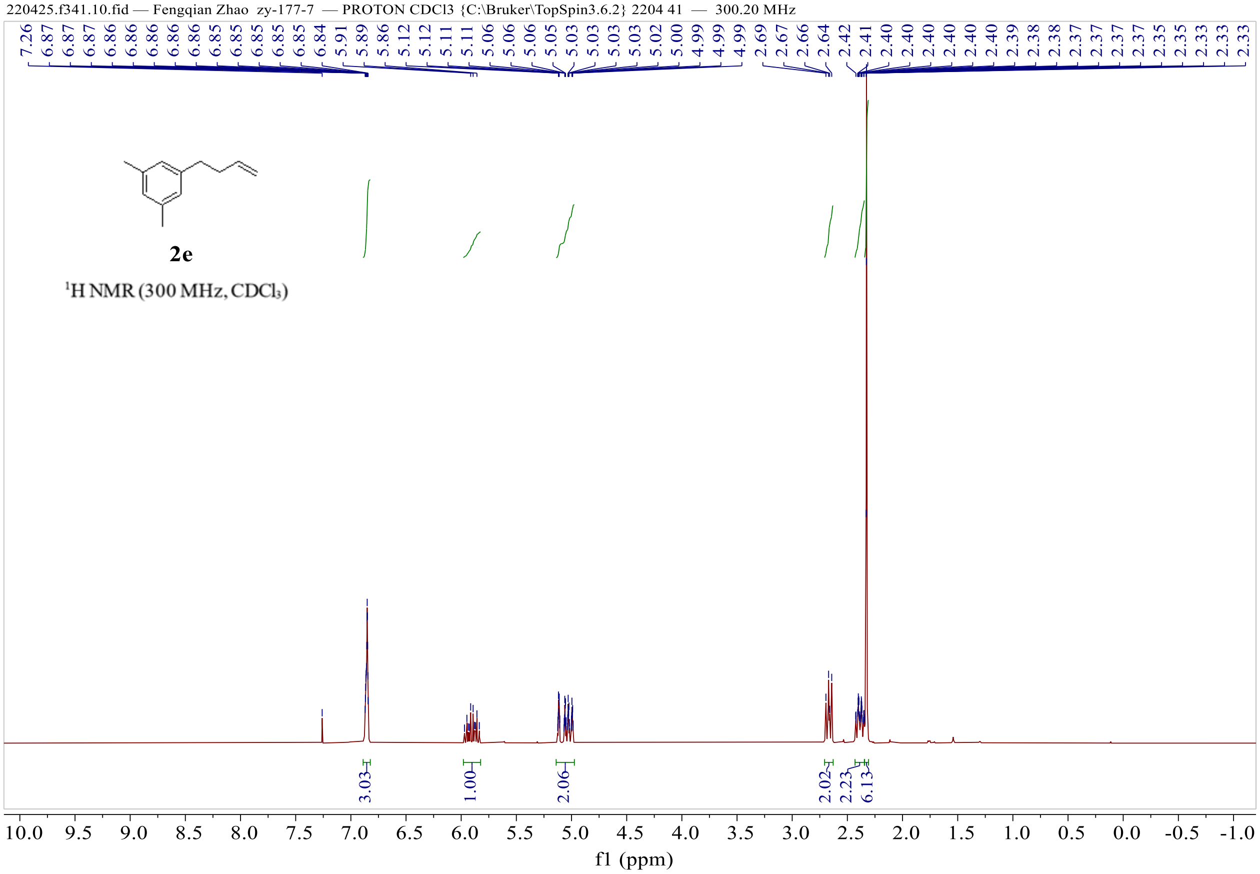

220425.f341.10.fid — Fengqian Zhao zy-177-7 — PROTON CDCl3 {C:\Bruker\TopSpin3.6.2} 2204 41 — 300.20 MHz
2e
1H NMR (300 MHz, CDCl3)
f1 (ppm)

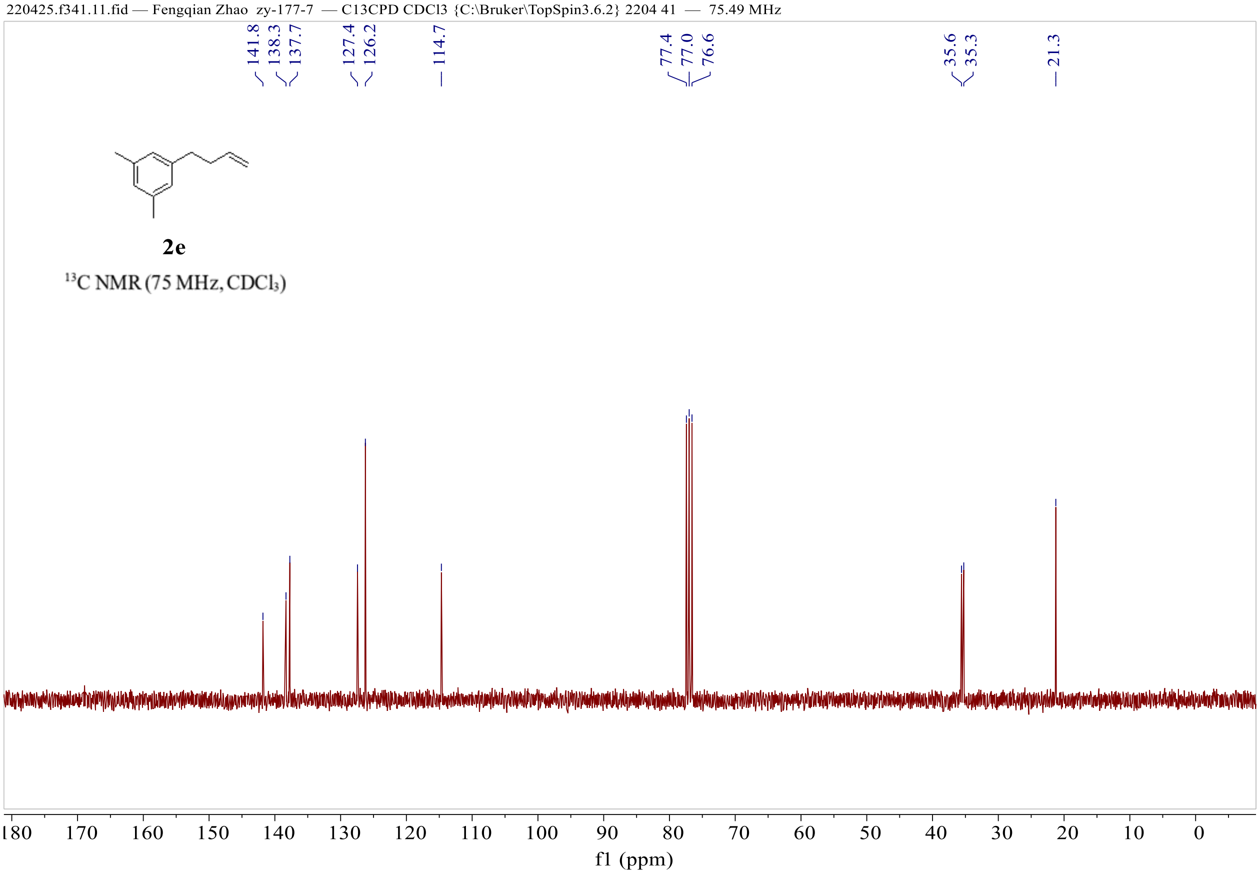

220425.f341.11.fid — Fengqian Zhao zy-177-7 — C13CPD CDCl3 {C:\Bruker\TopSpin3.6.2} 2204 41 — 75.49 MHz
141.8
138.3
137.7
127.4
126.2
114.7
77.4
77.0
76.6
35.6
35.3
21.3
2e
13C NMR (75 MHz, CDCl3)
f1 (ppm)

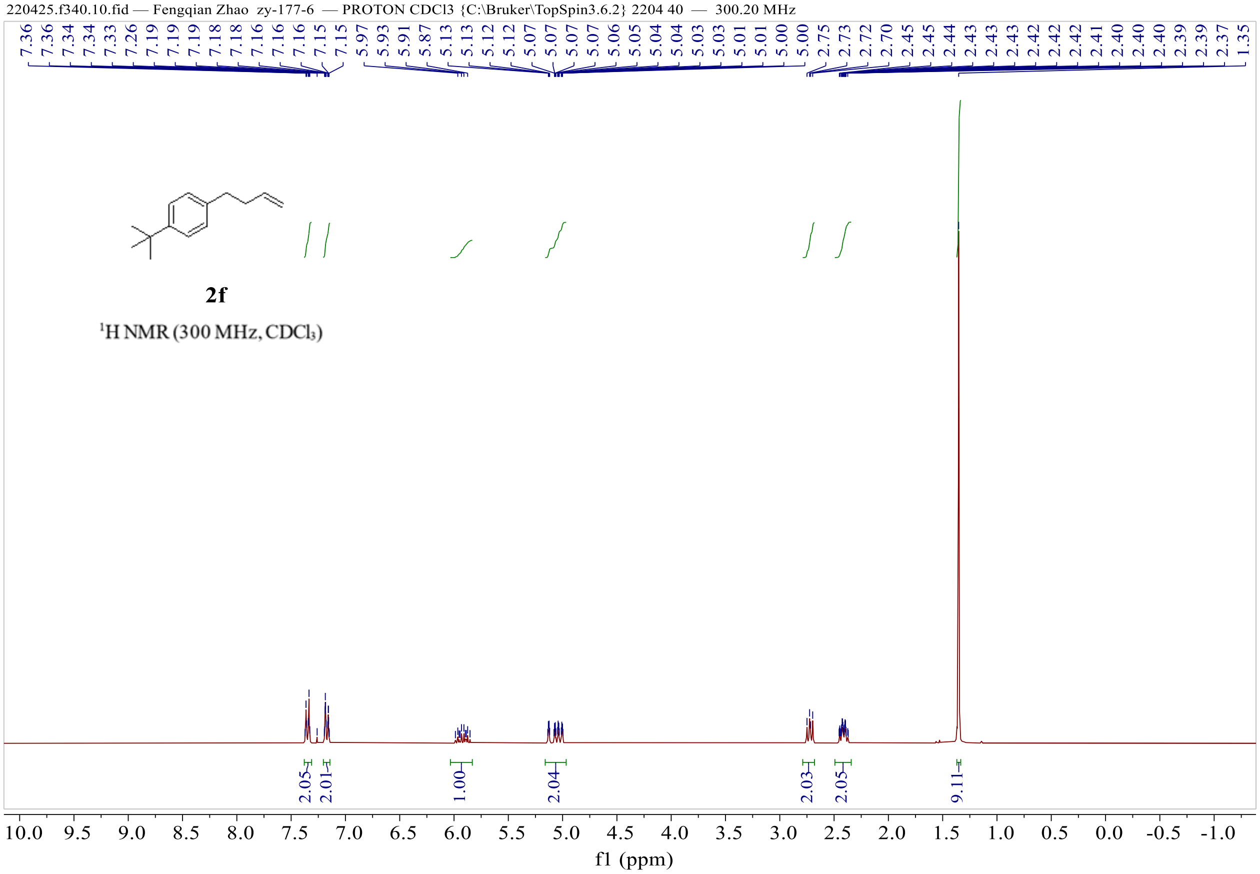
220425.f340.10.fid — Fengqian Zhao zy-177-6 — PROTON CDCl3 {C:\Bruker\TopSpin3.6.2} 2204 40 — 300.20 MHz
7.36 7.36 7.34 7.34 7.33 7.26 7.19 7.19 7.19 7.18 7.18 7.16 7.16 7.16 7.15 7.15 5.97 5.93 5.91 5.87 5.13 5.13 5.12 5.12 5.07 5.07 5.07 5.07 5.06 5.05 5.04 5.04 5.03 5.03 5.01 5.01 5.00 5.00 2.75 2.73 2.72 2.70 2.45 2.45 2.44 2.43 2.43 2.43 2.42 2.42 2.42 2.41 2.40 2.40 2.40 2.39 2.39 2.37 1.35
2f
1H NMR (300 MHz, CDCl3)
2.05 2.01 1.00 2.04 2.03 2.05 9.11
f1 (ppm)

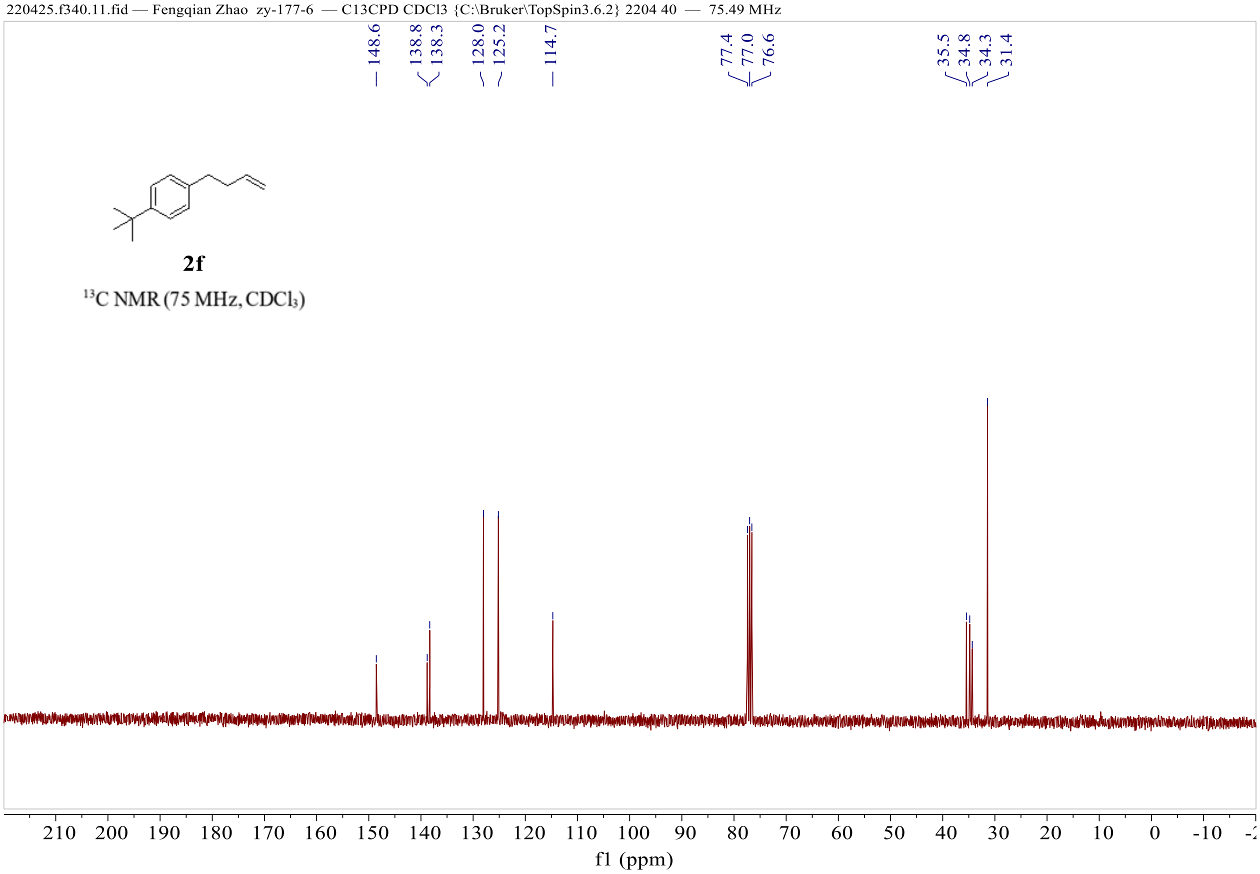
220425.f340.11.fid — Fengqian Zhao zy-177-6 — C13CPD CDCl3 {C:\Bruker\TopSpin3.6.2} 2204 40 — 75.49 MHz
148.6 138.8 138.3 128.0 125.2 114.7 77.4 77.0 76.6 35.5 34.8 34.3 31.4
2f
13C NMR (75 MHz, CDCl3)
f1 (ppm)

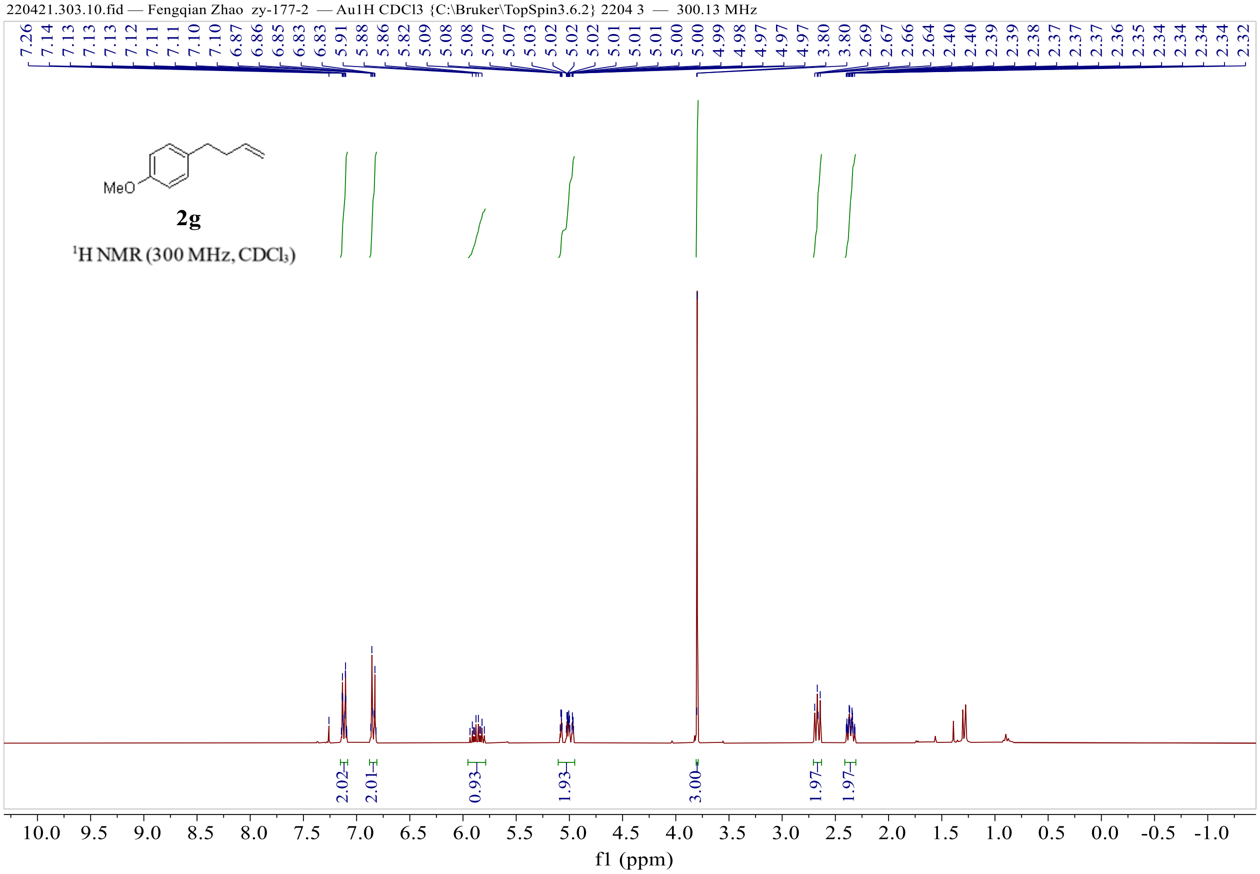
220421.303.10.fid — Fengqian Zhao zy-177-2 — Au1H CDCl3 {C:\Bruker\TopSpin3.6.2} 2204 3 — 300.13 MHz
7.26 7.14 7.13 7.13 7.12 7.11 7.11 7.10 7.10 6.87 6.86 6.85 6.83 6.83 5.91 5.88 5.86 5.82 5.09 5.08 5.08 5.07 5.07 5.03 5.02 5.02 5.01 5.01 5.01 5.00 5.00 4.99 4.98 4.97 4.97 4.97 3.80 3.80 2.69 2.67 2.66 2.64 2.40 2.40 2.39 2.39 2.38 2.37 2.37 2.37 2.36 2.35 2.34 2.34 2.34 2.34 2.32
MeO
2g
1H NMR (300 MHz, CDCl3)
2.02 2.01 0.93 1.93 3.00 1.97 1.97
10.0 9.5 9.0 8.5 8.0 7.5 7.0 6.5 6.0 5.5 5.0 4.5 4.0 3.5 3.0 2.5 2.0 1.5 1.0 0.5 0.0 -0.5 -1.0
f1 (ppm)

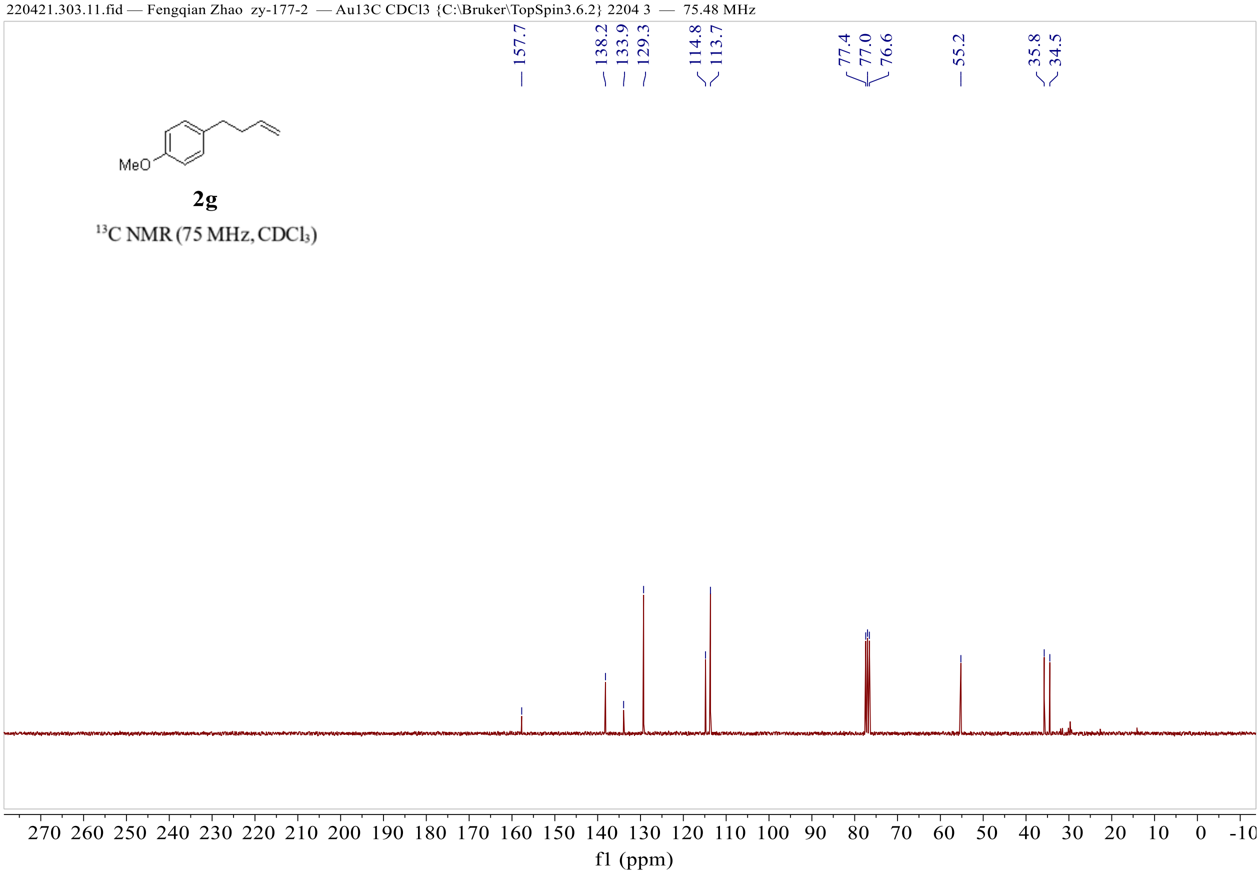
220421.303.11.fid — Fengqian Zhao zy-177-2 — Au13C CDCl3 {C:\Bruker\TopSpin3.6.2} 2204 3 — 75.48 MHz
157.7 138.2 133.9 129.3 114.8 113.7 77.4 77.0 76.6 55.2 35.8 34.5
MeO
2g
13C NMR (75 MHz, CDCl3)
270 260 250 240 230 220 210 200 190 180 170 160 150 140 130 120 110 100 90 80 70 60 50 40 30 20 10 0 -10
f1 (ppm)

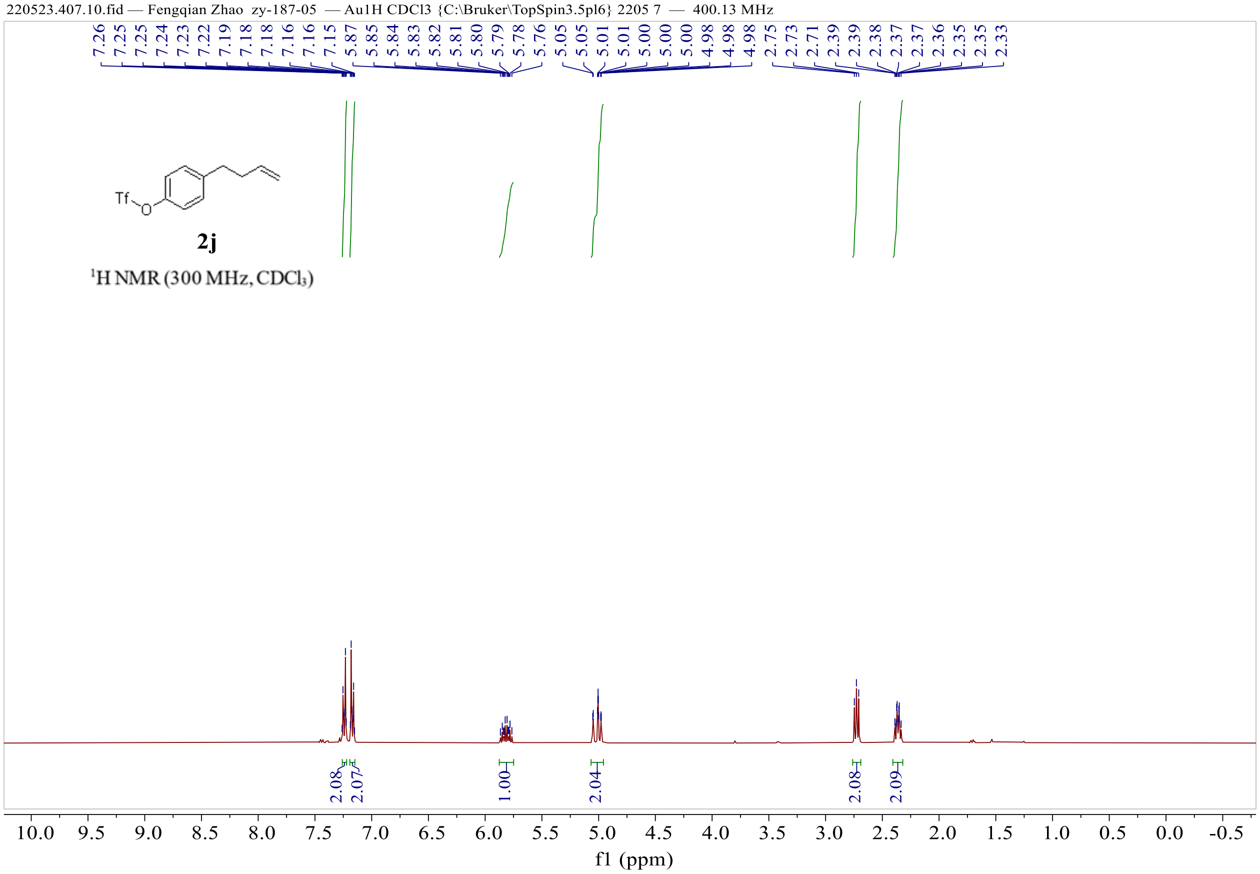
220523.407.10.fid — Fengqian Zhao zy-187-05 — Au1H CDCl3 {C:\Bruker\TopSpin3.5pl6} 2205 7 — 400.13 MHz
7.26 7.25 7.25 7.24 7.23 7.22 7.19 7.18 7.18 7.16 7.16 7.15 5.87 5.85 5.84 5.83 5.82 5.81 5.80 5.79 5.78 5.76 5.05 5.05 5.01 5.01 5.00 5.00 5.00 4.98 4.98 4.98 2.75 2.73 2.71 2.39 2.39 2.38 2.37 2.37 2.36 2.35 2.35 2.33
Tf
O
2j
1H NMR (300 MHz, CDCl3)
2.08 2.07 1.00 2.04 2.08 2.09
10.0 9.5 9.0 8.5 8.0 7.5 7.0 6.5 6.0 5.5 5.0 4.5 4.0 3.5 3.0 2.5 2.0 1.5 1.0 0.5 0.0 -0.5
f1 (ppm)

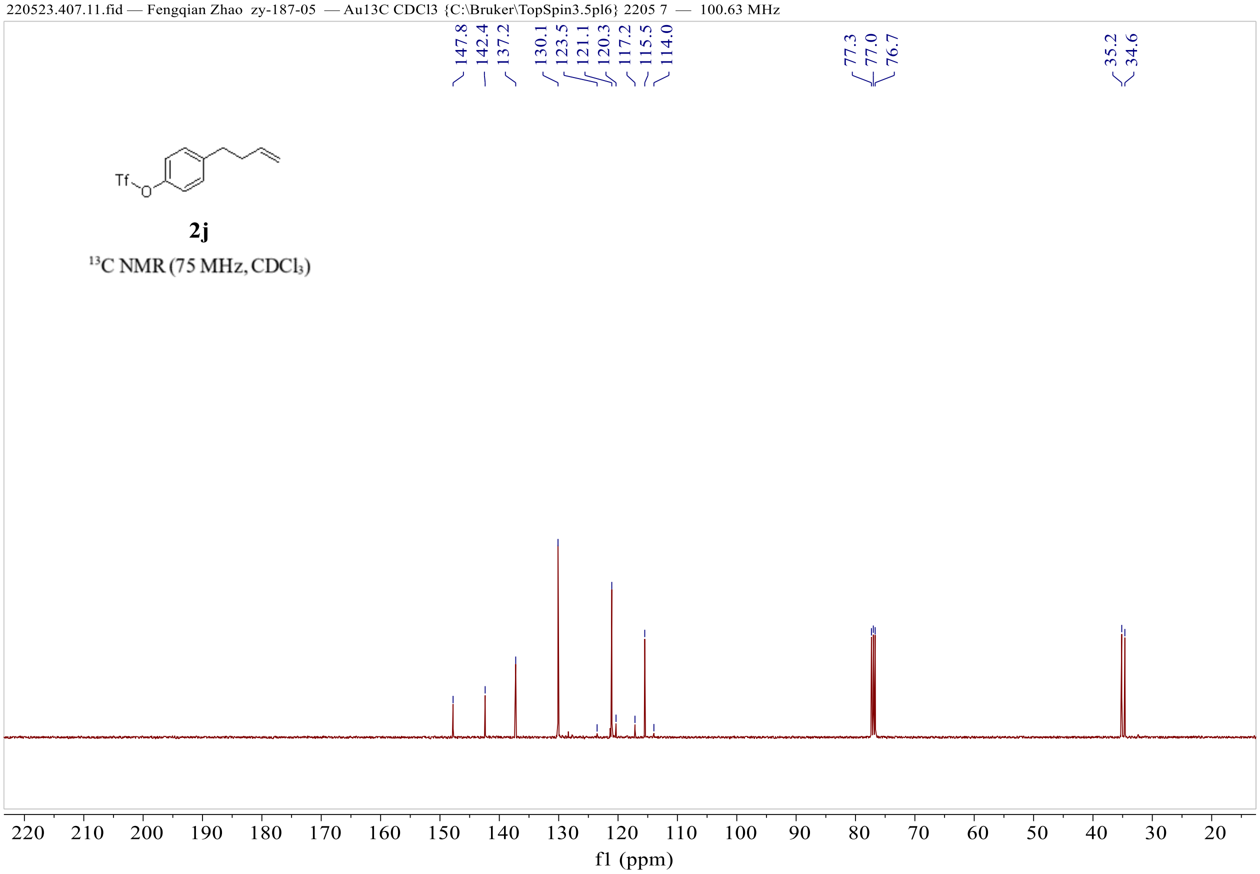
220523.407.11.fid — Fengqian Zhao zy-187-05 — Au13C CDCl3 {C:\Bruker\TopSpin3.5pl6} 2205 7 — 100.63 MHz
147.8 142.4 137.2 130.1 123.5 121.1 120.3 117.2 115.5 114.0 77.3 77.0 76.7 35.2 34.6
Tf
O
2j
13C NMR (75 MHz, CDCl3)
220 210 200 190 180 170 160 150 140 130 120 110 100 90 80 70 60 50 40 30 20
f1 (ppm)

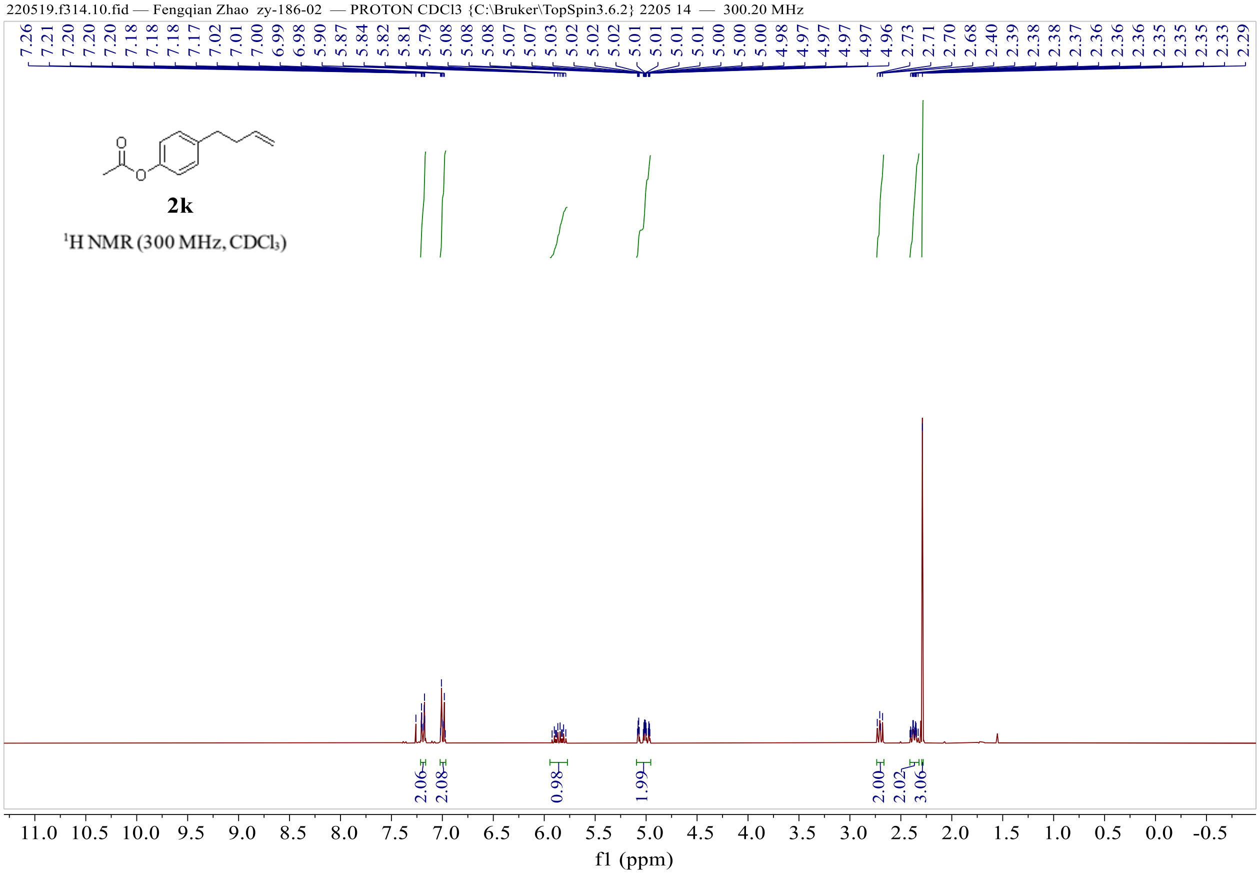
220519.f314.10.fid — Fengqian Zhao zy-186-02 — PROTON CDCl3 {C:\Bruker\TopSpin3.6.2} 2205 14 — 300.20 MHz
7.26 7.21 7.20 7.20 7.20 7.18 7.18 7.18 7.17 7.02 7.01 7.00 6.99 6.98 5.90 5.87 5.84 5.82 5.81 5.79 5.08 5.08 5.08 5.07 5.07 5.03 5.02 5.02 5.02 5.01 5.01 5.01 5.01 5.00 5.00 5.00 4.98 4.97 4.97 4.97 4.96 2.73 2.71 2.70 2.68 2.40 2.39 2.38 2.38 2.37 2.36 2.36 2.36 2.35 2.35 2.35 2.33 2.29
2k
^{1}H NMR (300 MHz, CDCl$_3$)
2.06 2.08 0.98 1.99 2.00 2.02 3.06
11.0 10.5 10.0 9.5 9.0 8.5 8.0 7.5 7.0 6.5 6.0 5.5 5.0 4.5 4.0 3.5 3.0 2.5 2.0 1.5 1.0 0.5 0.0 -0.5
f1 (ppm)

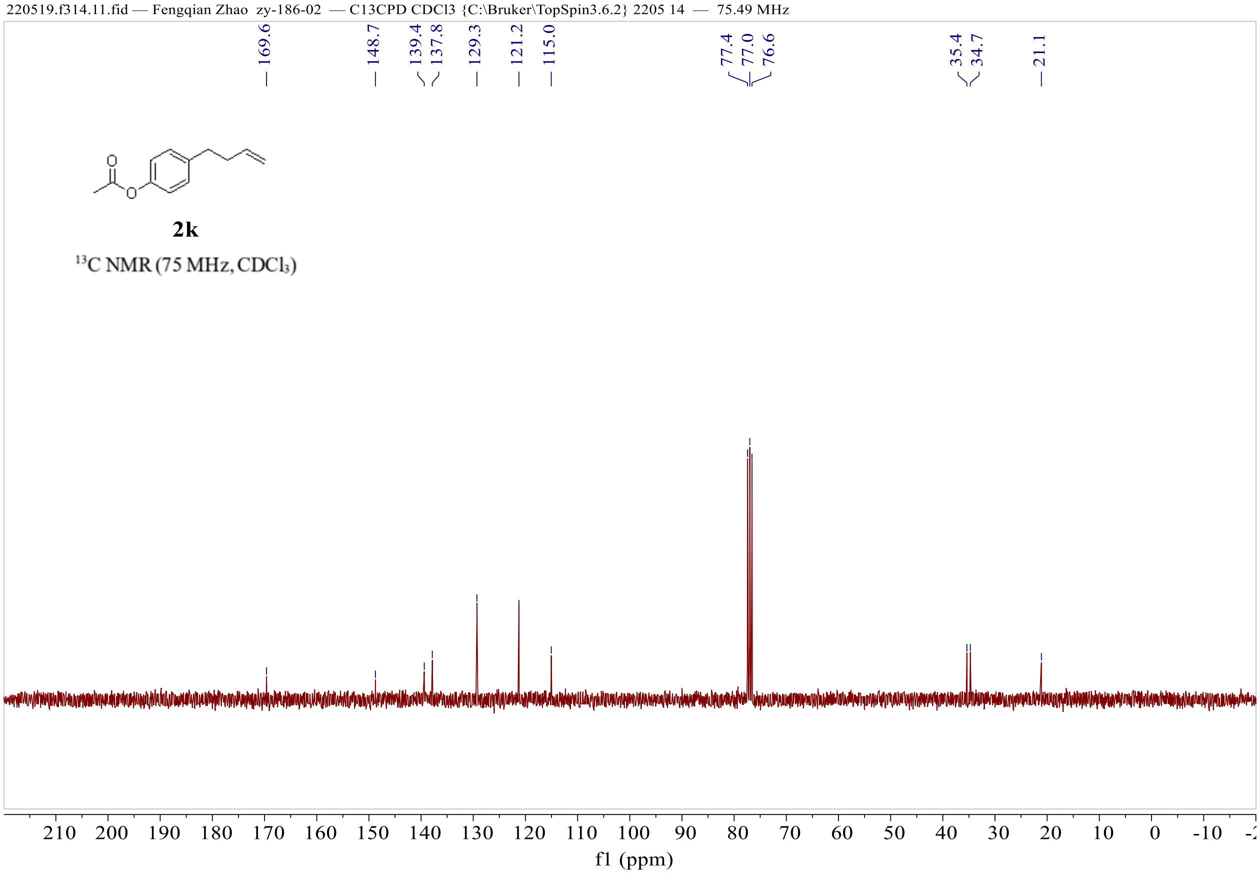
220519.f314.11.fid — Fengqian Zhao zy-186-02 — C13CPD CDCl3 {C:\Bruker\TopSpin3.6.2} 2205 14 — 75.49 MHz
169.6 148.7 139.4 137.8 129.3 121.2 115.0 77.4 77.0 76.6 35.4 34.7 21.1
2k
^{13}C NMR (75 MHz, CDCl$_3$)
210 200 190 180 170 160 150 140 130 120 110 100 90 80 70 60 50 40 30 20 10 0 -10
f1 (ppm)

220426.f348.10.fid — Fengqian Zhao zy-177-10 — PROTON CDCl3 {C:\Bruker\TopSpin3.6.2} 2204 48 — 300.20 MHz

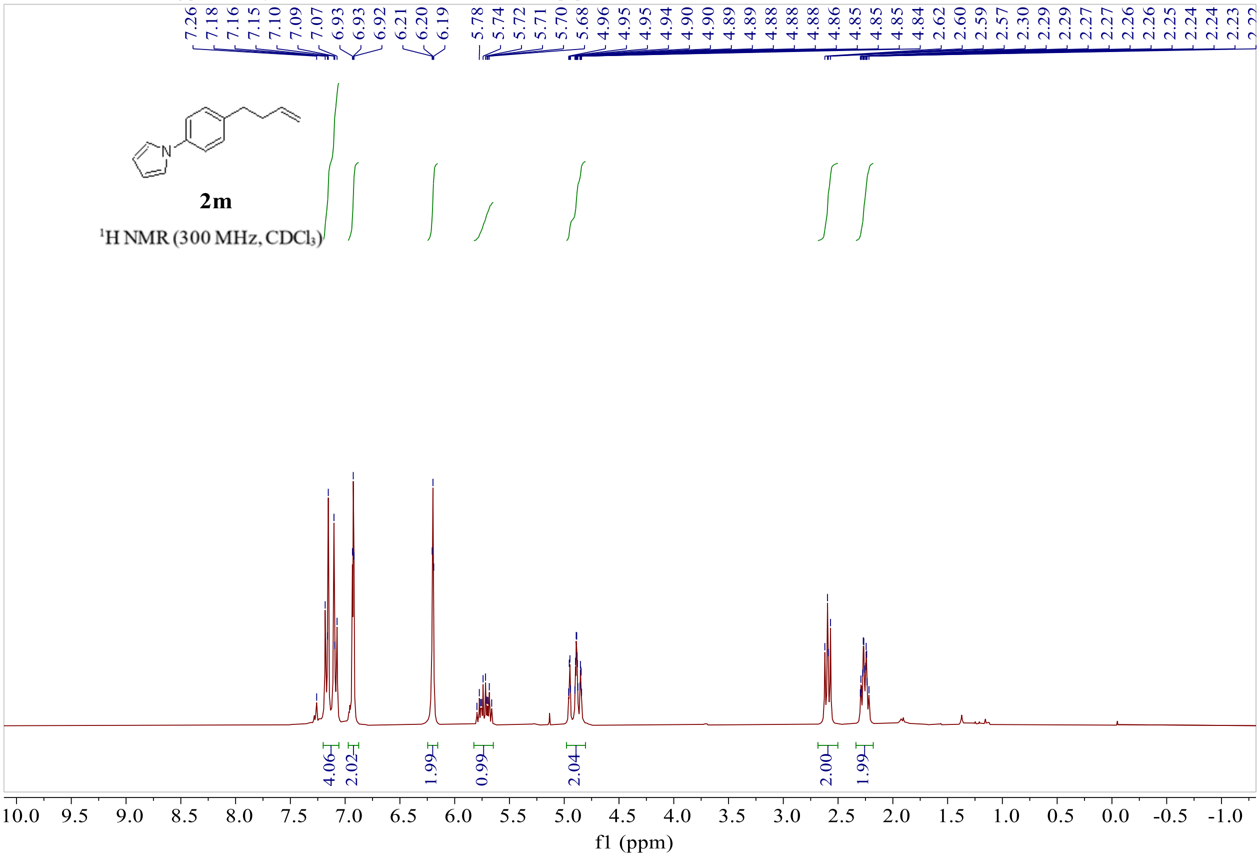

220426.f348.11.fid — Fengqian Zhao zy-177-10 — C13CPD CDCl3 {C:\Bruker\TopSpin3.6.2} 2204 48 — 75.49 MHz

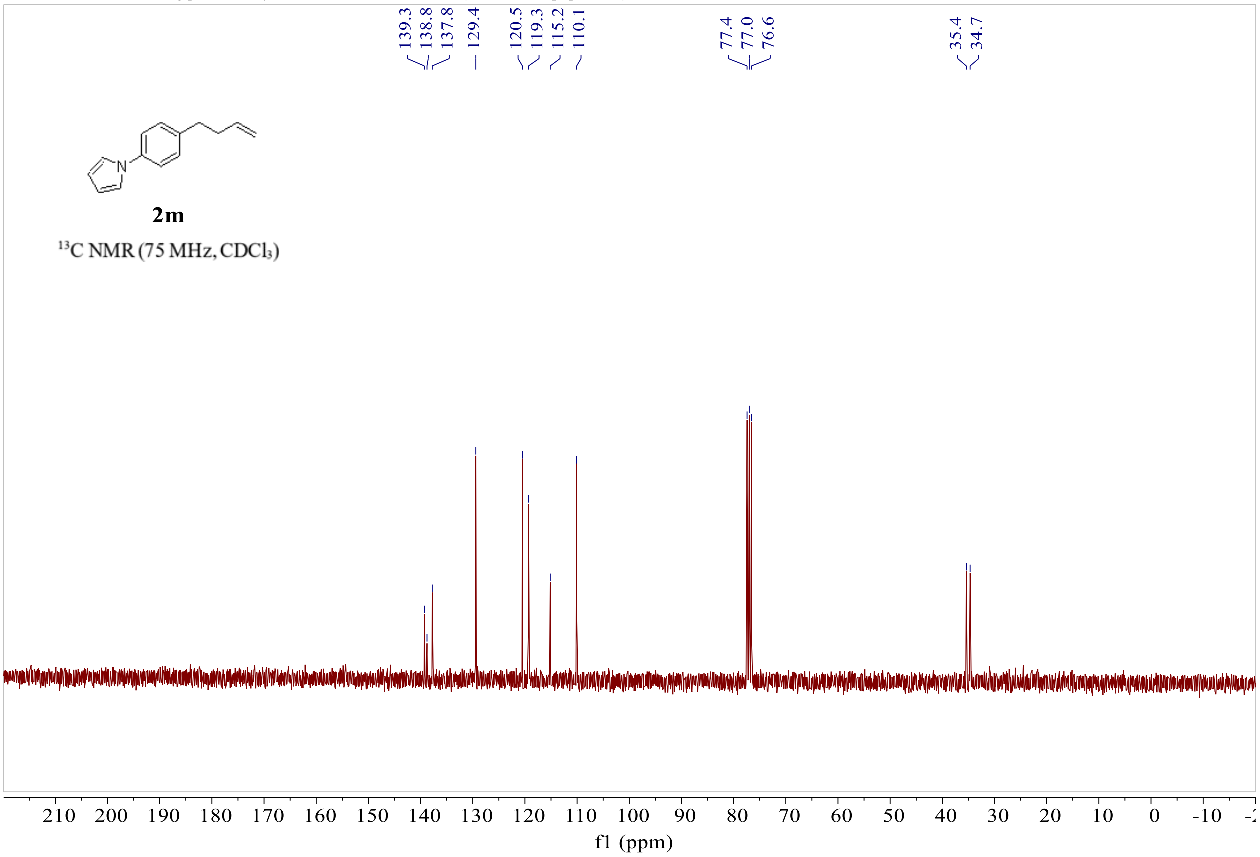

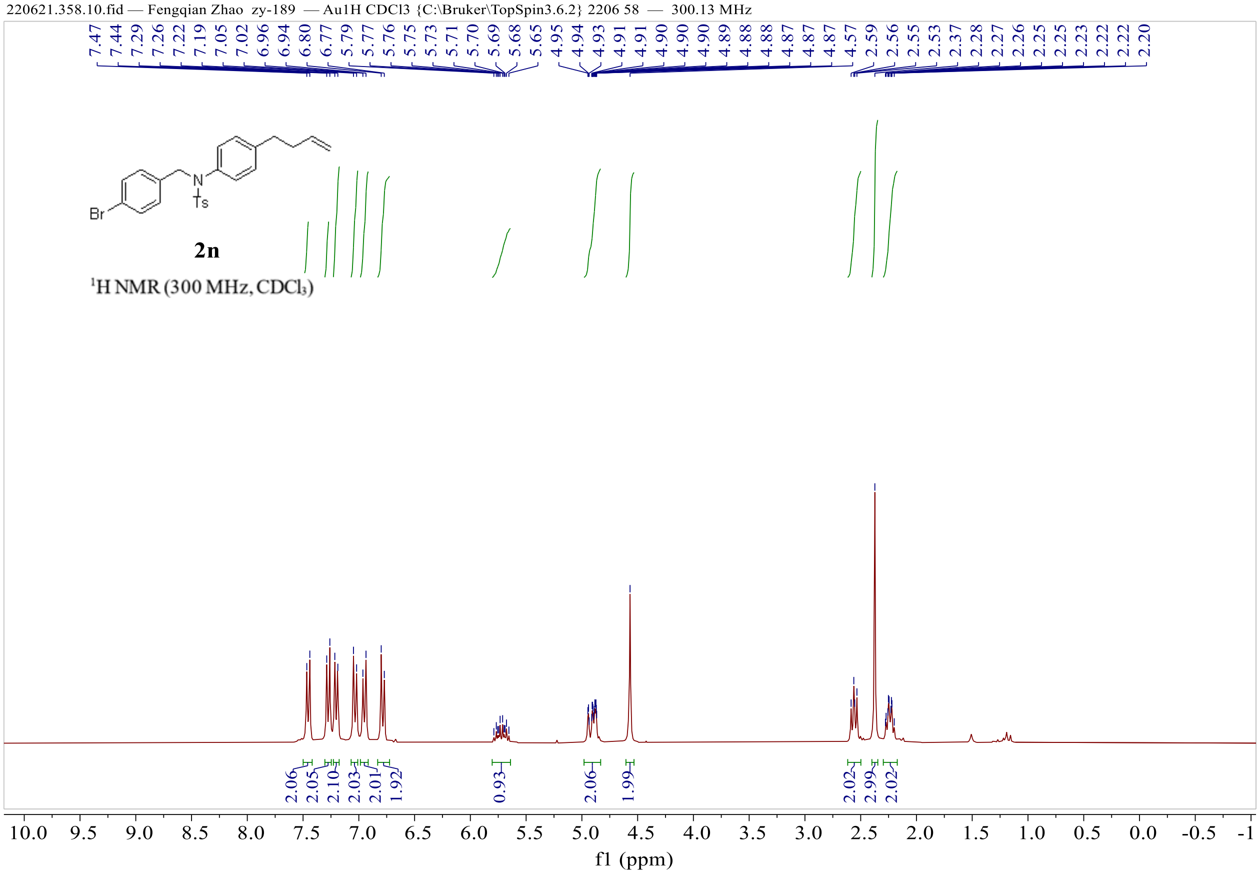
220621.358.10.fid — Fengqian Zhao zy-189 — Au1H CDCl3 {C:\Bruker\TopSpin3.6.2} 2206 58 — 300.13 MHz
2n
1H NMR (300 MHz, CDCl3)
f1 (ppm)

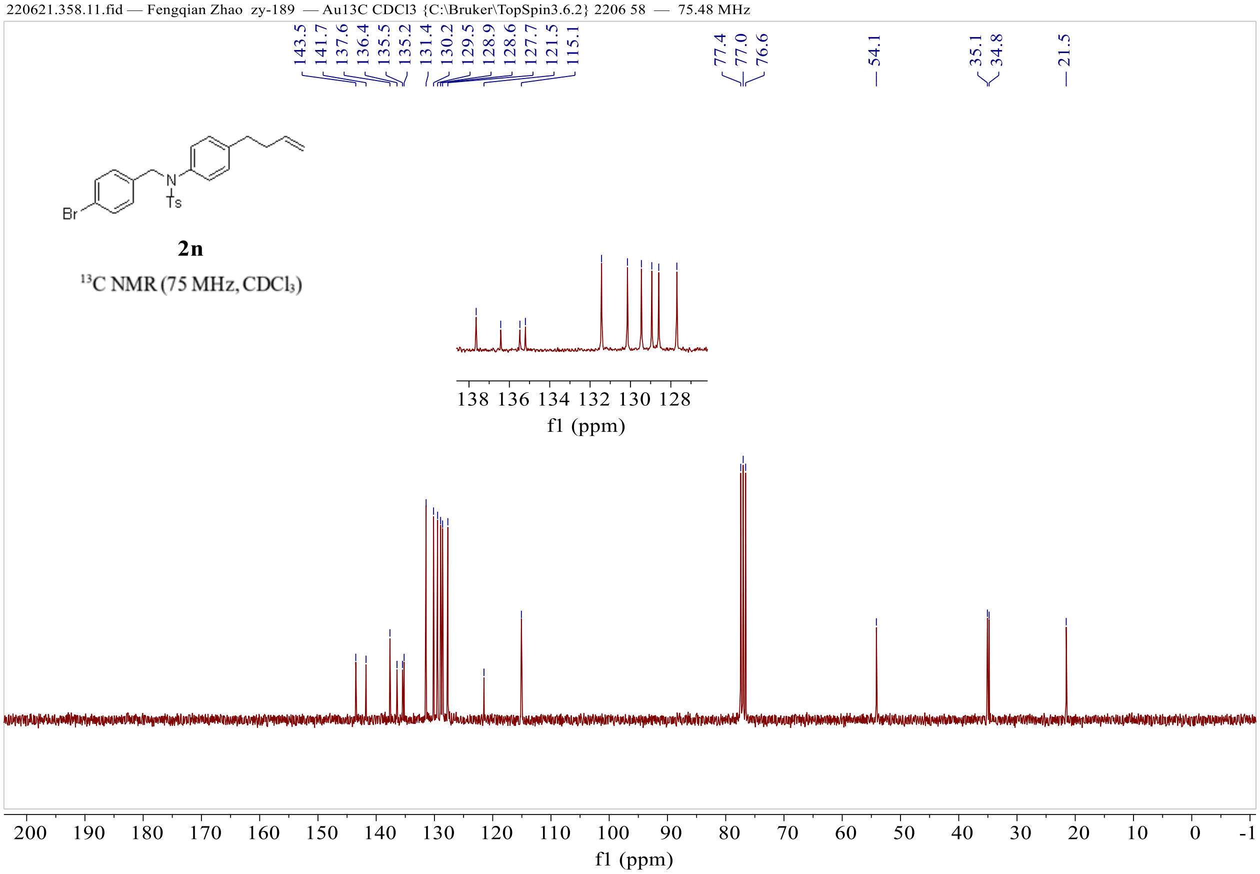
220621.358.11.fid — Fengqian Zhao zy-189 — Au13C CDCl3 {C:\Bruker\TopSpin3.6.2} 2206 58 — 75.48 MHz
2n
13C NMR (75 MHz, CDCl3)
f1 (ppm)

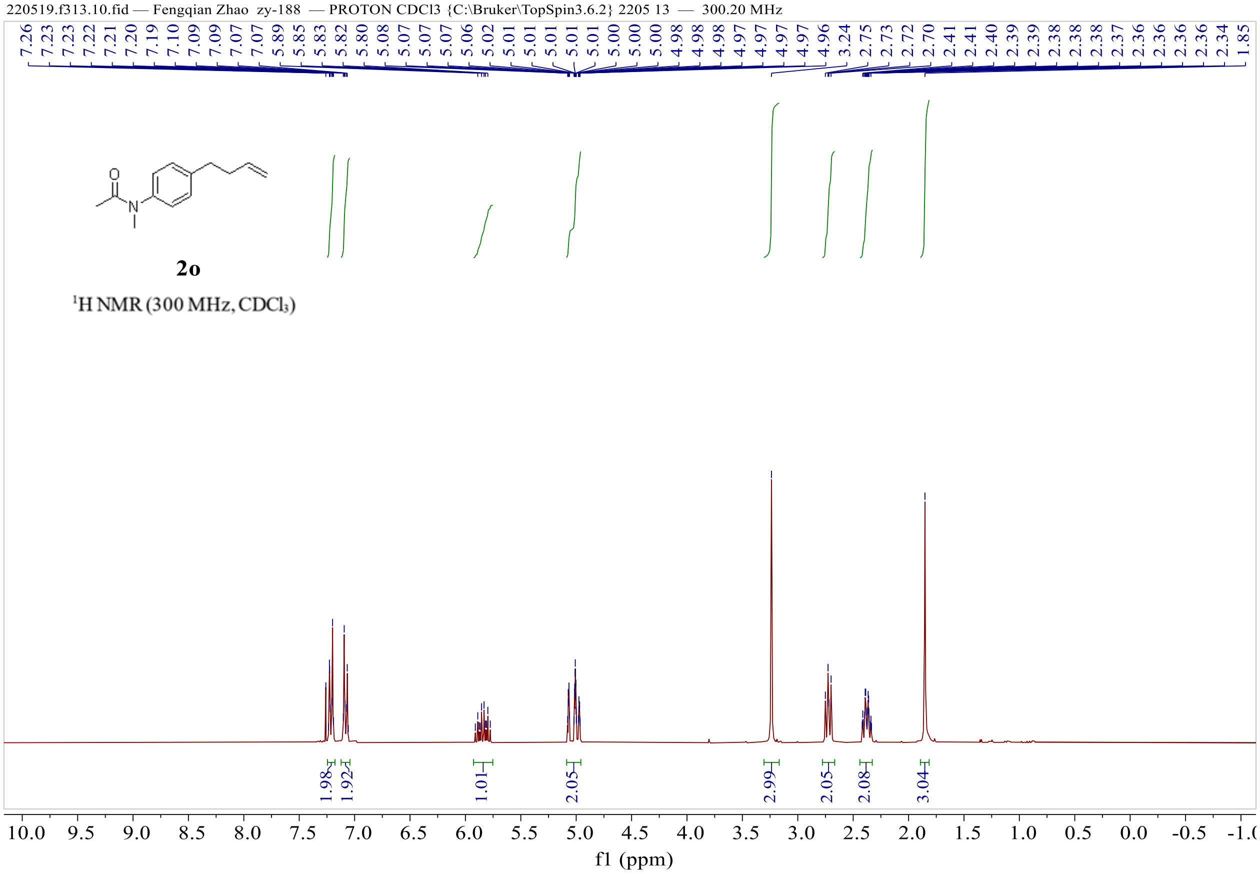
220519.f313.10.fid — Fengqian Zhao zy-188 — PROTON CDCl3 {C:\Bruker\TopSpin3.6.2} 2205 13 — 300.20 MHz
7.26 7.23 7.23 7.22 7.21 7.20 7.19 7.10 7.09 7.09 7.07 7.07 5.89 5.85 5.83 5.82 5.80 5.08 5.07 5.07 5.07 5.06 5.02 5.01 5.01 5.01 5.01 5.01 5.00 5.00 5.00 4.98 4.98 4.98 4.97 4.97 4.97 4.97 4.96 3.24 2.75 2.73 2.72 2.70 2.41 2.41 2.40 2.39 2.39 2.38 2.38 2.38 2.37 2.36 2.36 2.36 2.36 2.34 1.85
2o
1H NMR (300 MHz, CDCl3)
1.98 1.92 1.01 2.05 2.99 2.05 2.08 3.04
10.0 9.5 9.0 8.5 8.0 7.5 7.0 6.5 6.0 5.5 5.0 4.5 4.0 3.5 3.0 2.5 2.0 1.5 1.0 0.5 0.0 -0.5
f1 (ppm)

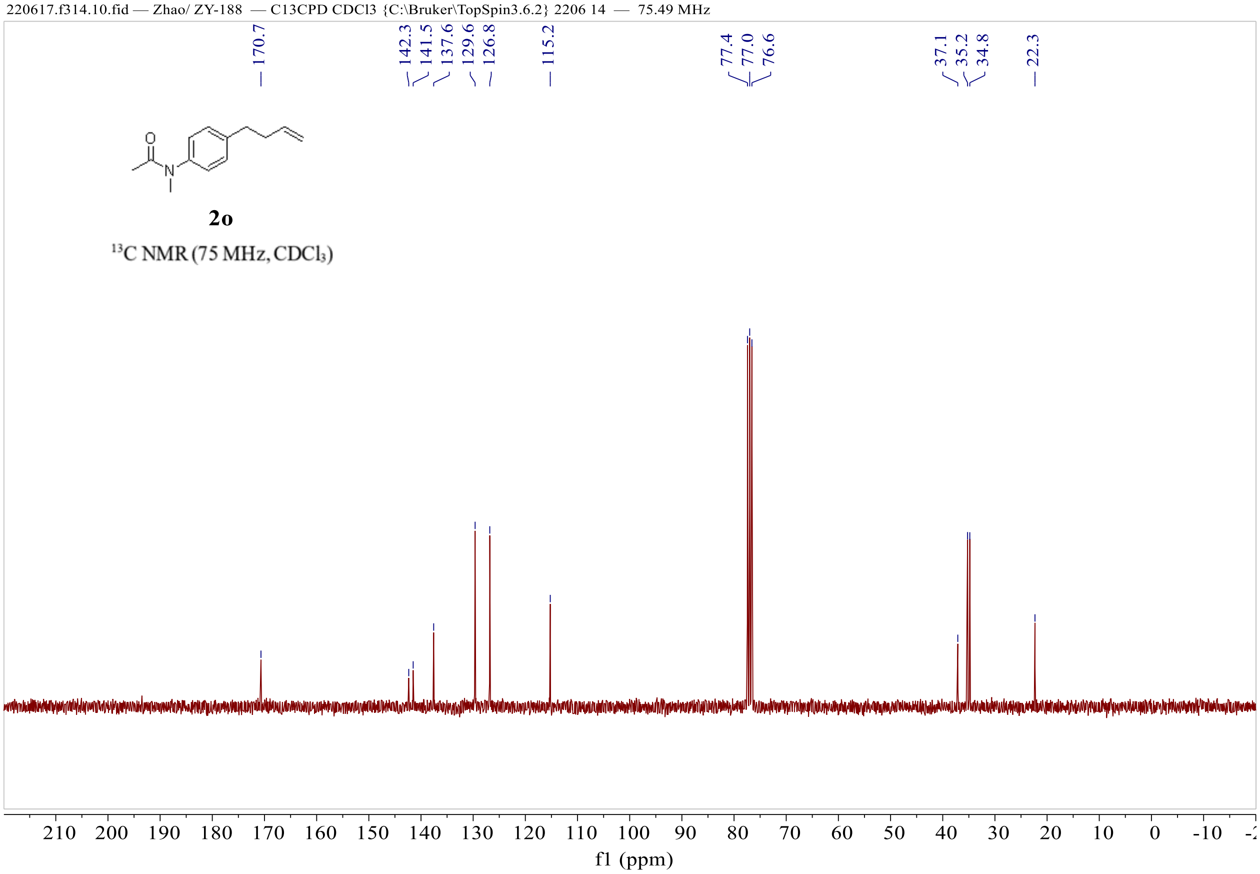
220617.f314.10.fid — Zhao/ ZY-188 — C13CPD CDCl3 {C:\Bruker\TopSpin3.6.2} 2206 14 — 75.49 MHz
170.7 142.3 141.5 137.6 129.6 126.8 115.2 77.4 77.0 76.6 37.1 35.2 34.8 22.3
2o
13C NMR (75 MHz, CDCl3)
210 200 190 180 170 160 150 140 130 120 110 100 90 80 70 60 50 40 30 20 10 0 -10
f1 (ppm)

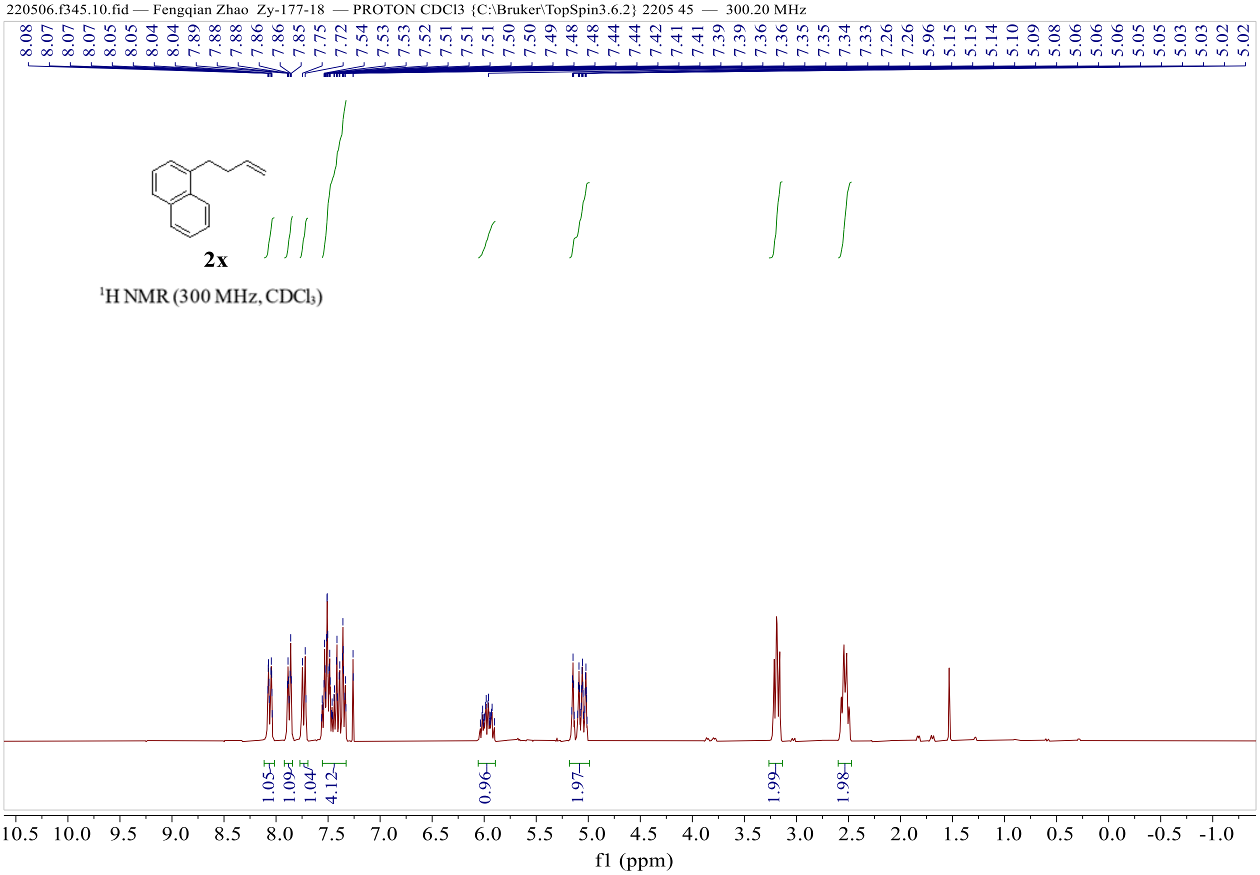
220506.f345.10.fid — Fengqian Zhao Zy-177-18 — PROTON CDCl3 {C:\Bruker\TopSpin3.6.2} 2205 45 — 300.20 MHz
8.08 8.07 8.07 8.07 8.05 8.05 8.04 8.04 7.89 7.88 7.88 7.86 7.86 7.85 7.75 7.72 7.54 7.53 7.53 7.52 7.51 7.51 7.51 7.50 7.50 7.49 7.48 7.48 7.44 7.44 7.42 7.41 7.41 7.39 7.39 7.36 7.36 7.35 7.35 7.34 7.33 7.26 7.26 5.96 5.15 5.15 5.14 5.10 5.09 5.08 5.06 5.06 5.06 5.05 5.05 5.03 5.03 5.02 5.02
2x
^{1}H NMR (300 MHz, CDCl$_3$)
1.05 1.09 1.04 4.12 0.96 1.97 1.99 1.98
10.5 10.0 9.5 9.0 8.5 8.0 7.5 7.0 6.5 6.0 5.5 5.0 4.5 4.0 3.5 3.0 2.5 2.0 1.5 1.0 0.5 0.0 -0.5 -1.0
f1 (ppm)

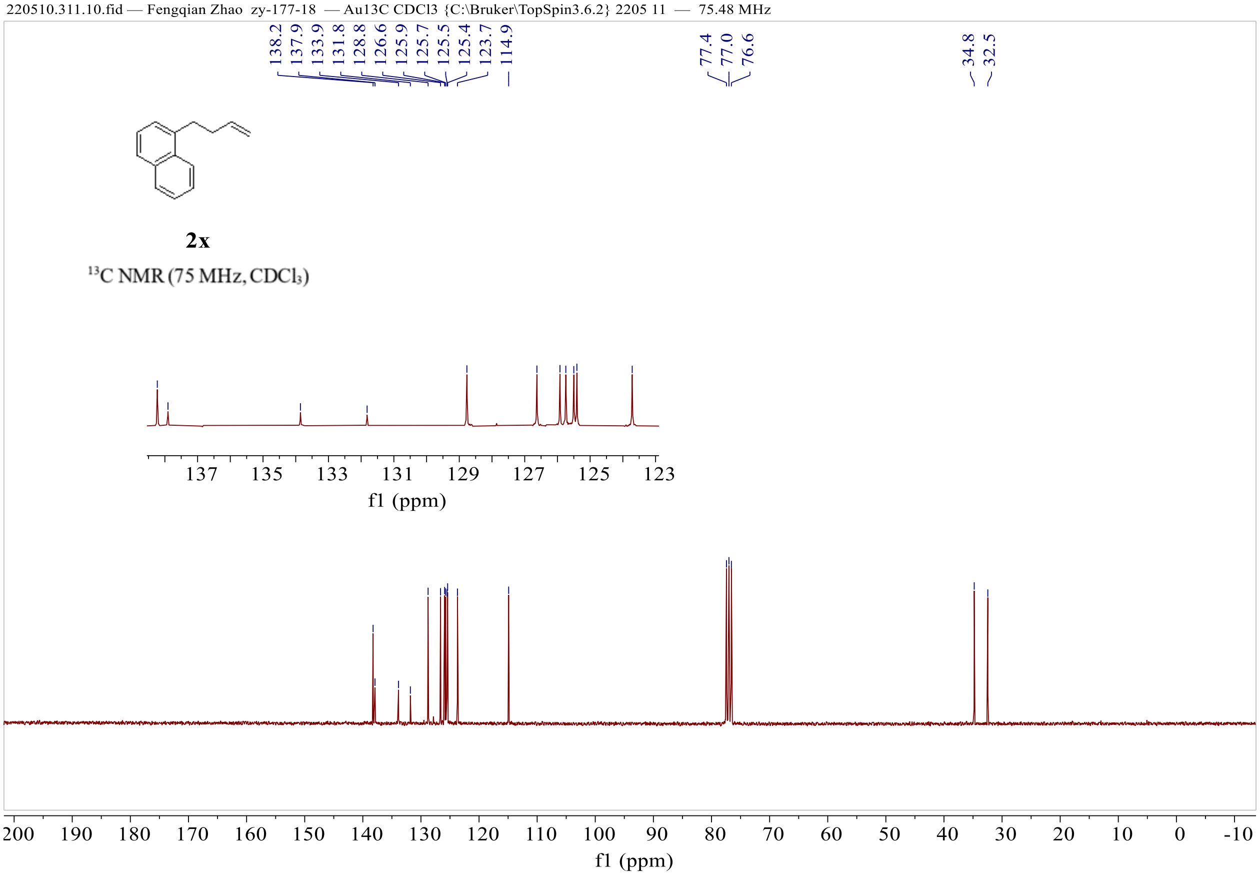
220510.311.10.fid — Fengqian Zhao zy-177-18 — Au13C CDCl3 {C:\Bruker\TopSpin3.6.2} 2205 11 — 75.48 MHz
138.2 137.9 133.9 131.8 128.8 126.6 125.9 125.7 125.5 125.4 123.7 114.9
77.4 77.0 76.6
34.8 32.5
2x
^{13}C NMR (75 MHz, CDCl$_3$)
137 135 133 131 129 127 125 123
f1 (ppm)
200 190 180 170 160 150 140 130 120 110 100 90 80 70 60 50 40 30 20 10 0 -10
f1 (ppm)

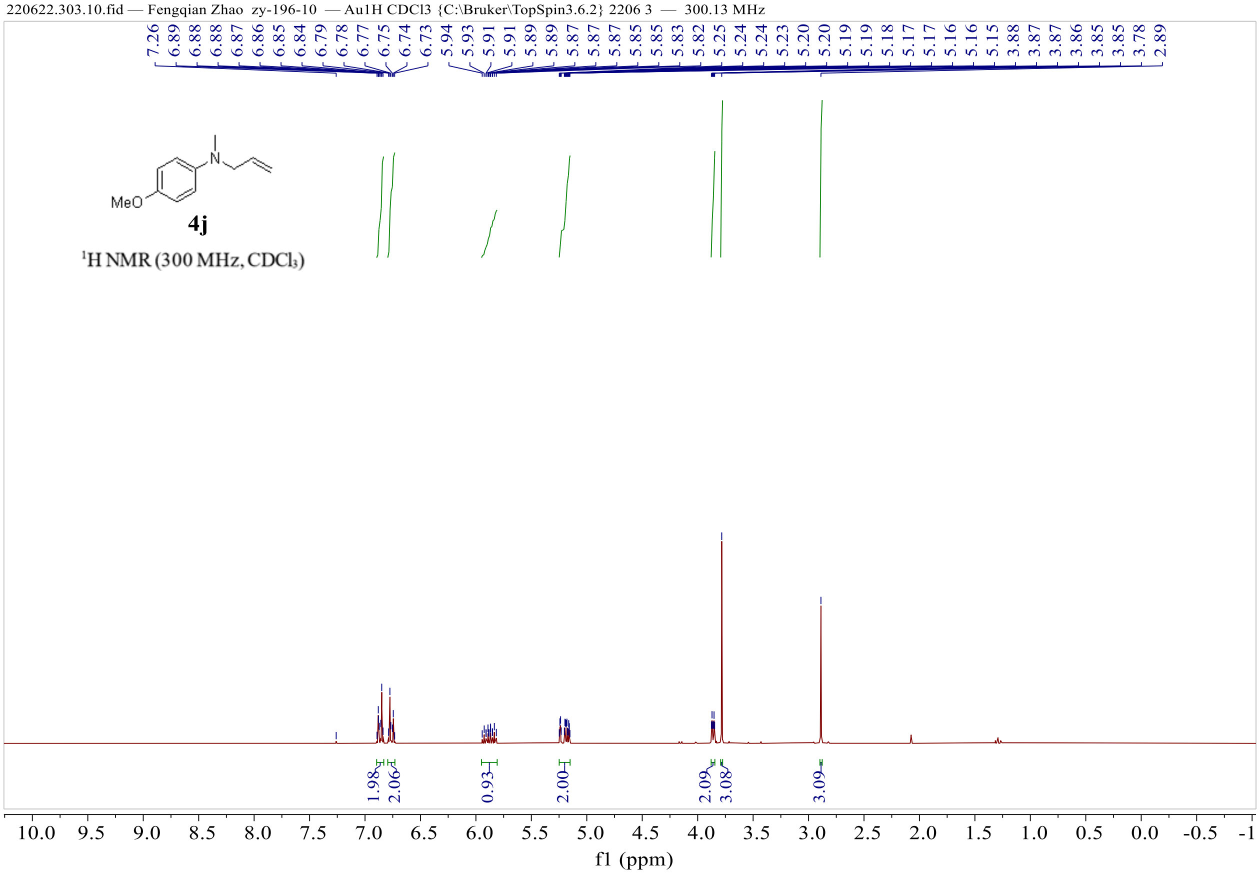
220622.303.10.fid — Fengqian Zhao zy-196-10 — Au1H CDCl3 {C:\Bruker\TopSpin3.6.2} 2206 3 — 300.13 MHz
7.26 6.89 6.88 6.88 6.87 6.86 6.85 6.84 6.79 6.78 6.77 6.75 6.74 6.73 5.94 5.93 5.91 5.91 5.89 5.89 5.87 5.87 5.87 5.85 5.85 5.83 5.82 5.25 5.24 5.24 5.23 5.20 5.20 5.19 5.19 5.18 5.17 5.17 5.16 5.15 3.88 3.87 3.87 3.86 3.85 3.85 3.78 2.89
MeO
N
4j
^{1}H NMR (300 MHz, CDCl$_3$)
1.98 2.06 0.93 2.00 2.09 3.08 3.09
f1 (ppm)

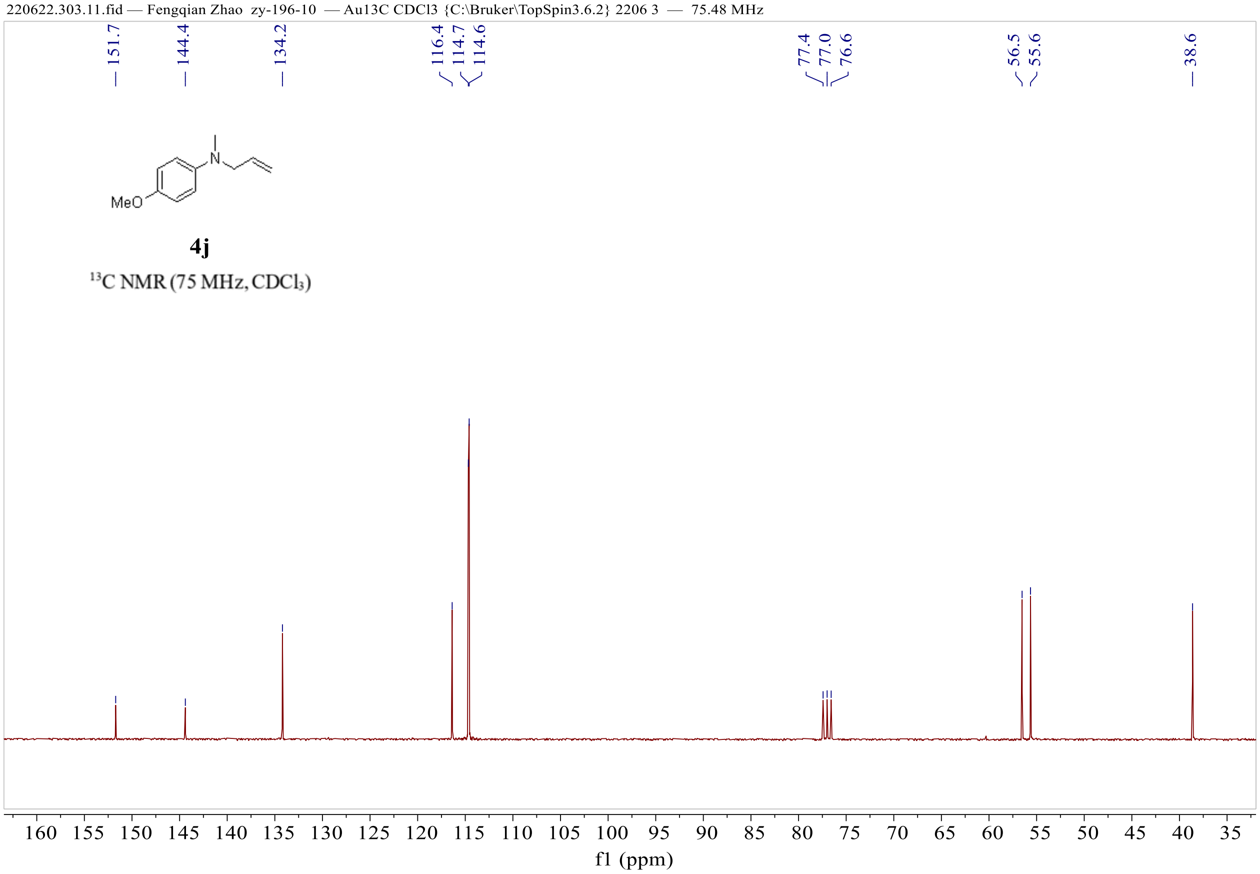
220622.303.11.fid — Fengqian Zhao zy-196-10 — Au13C CDCl3 {C:\Bruker\TopSpin3.6.2} 2206 3 — 75.48 MHz
151.7 144.4 134.2 116.4 114.7 114.6 77.4 77.0 76.6 56.5 55.6 38.6
MeO
N
4j
^{13}C NMR (75 MHz, CDCl$_3$)
f1 (ppm)

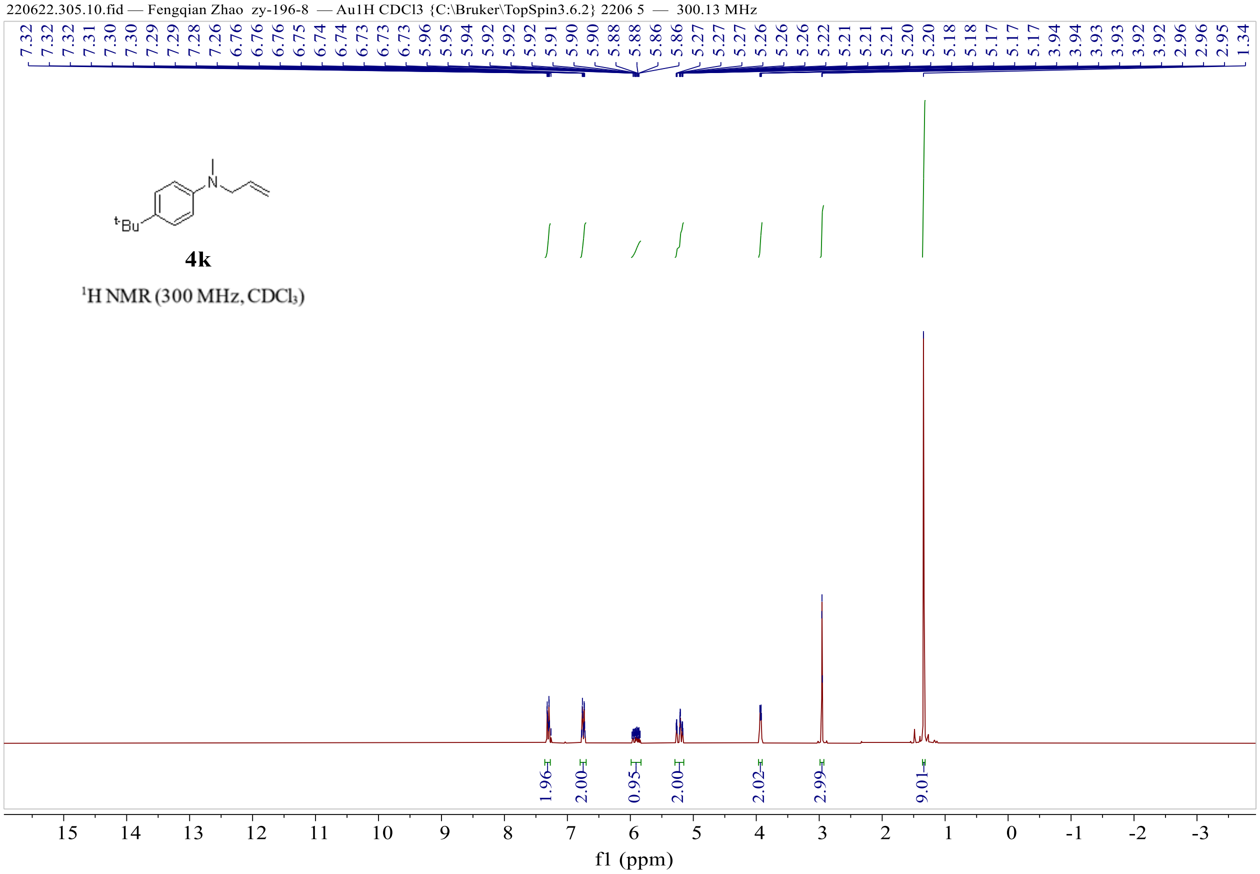
220622.305.10.fid — Fengqian Zhao zy-196-8 — Au1H CDCl3 {C:\Bruker\TopSpin3.6.2} 2206 5 — 300.13 MHz
7.32 7.32 7.32 7.31 7.30 7.30 7.29 7.29 7.28 7.26 6.76 6.76 6.76 6.75 6.75 6.74 6.74 6.73 6.73 6.73 5.96 5.95 5.94 5.94 5.92 5.92 5.92 5.91 5.90 5.90 5.88 5.88 5.86 5.86 5.27 5.27 5.27 5.26 5.26 5.22 5.21 5.21 5.21 5.20 5.20 5.18 5.17 5.17 5.17 3.94 3.94 3.93 3.93 3.92 3.92 2.96 2.96 2.95 1.34
N
tBu
4k
1H NMR (300 MHz, CDCl3)
1.96 2.00 0.95 2.00 2.02 2.99 9.01
15 14 13 12 11 10 9 8 7 6 5 4 3 2 1 0 -1 -2 -3
f1 (ppm)

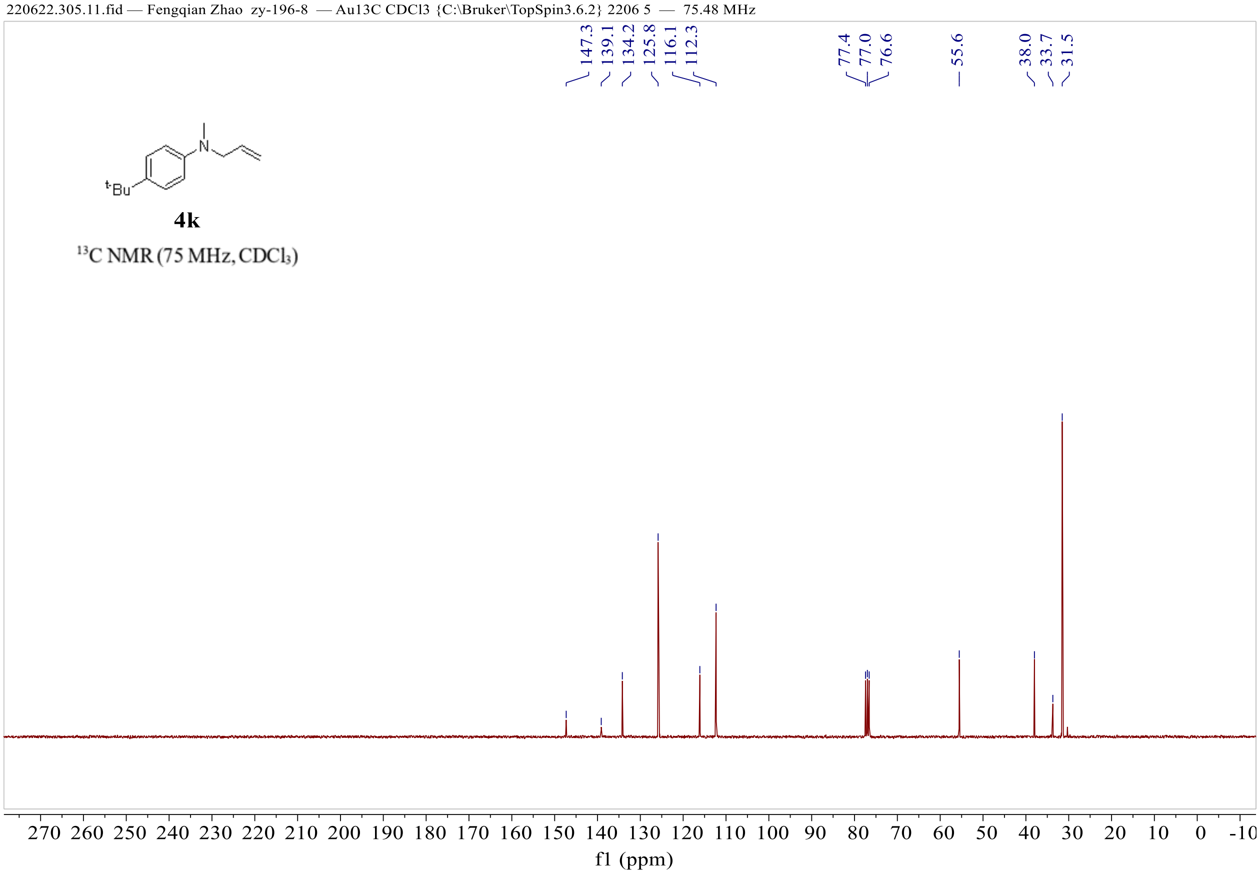
220622.305.11.fid — Fengqian Zhao zy-196-8 — Au13C CDCl3 {C:\Bruker\TopSpin3.6.2} 2206 5 — 75.48 MHz
147.3 139.1 134.2 125.8 116.1 112.3
77.4 77.0 76.6
55.6
38.0 33.7 31.5
N
tBu
4k
13C NMR (75 MHz, CDCl3)
270 260 250 240 230 220 210 200 190 180 170 160 150 140 130 120 110 100 90 80 70 60 50 40 30 20 10 0 -10
f1 (ppm)

220627.f346.10.fid — Zhao/ ZY-202-6 — PROTON CDCl3 {C:\Bruker\TopSpin3.6.2} 2206 46 — 300.20 MHz

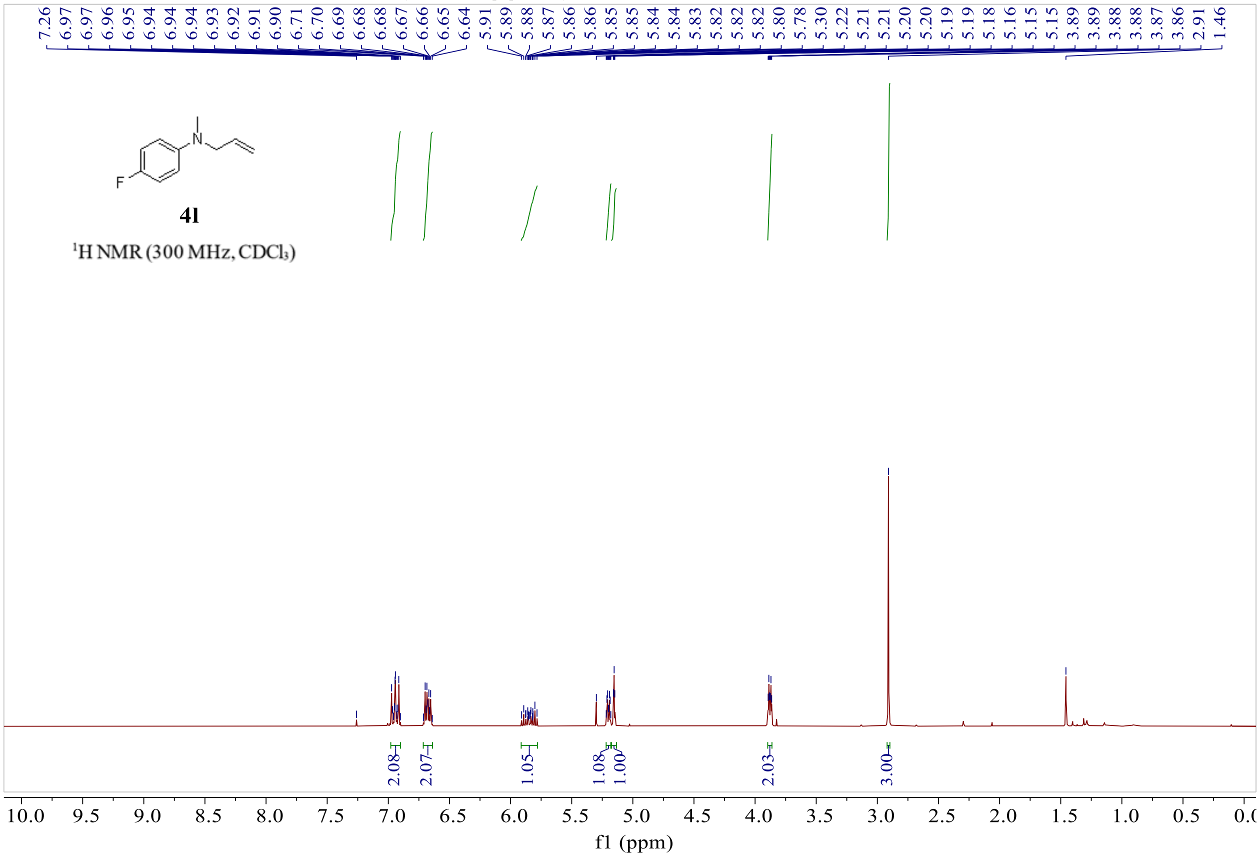

220627.f346.12.fid — Zhao/ ZY-202-6 — C13CPD CDCl3 {C:\Bruker\TopSpin3.6.2} 2206 46 — 75.49 MHz

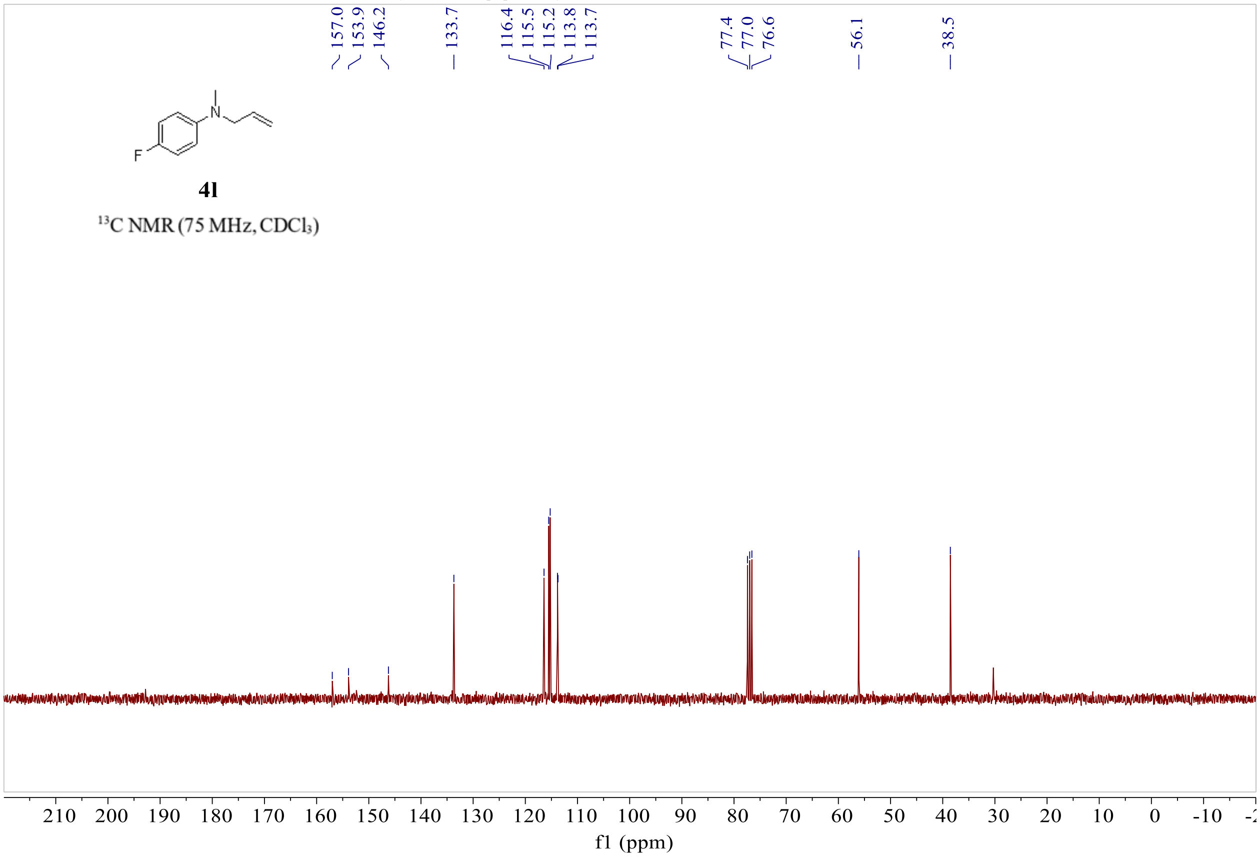

220627.f346.11.fid — Zhao/ ZY-202-6 — F19 CDCl3 {C:\Bruker\TopSpin3.6.2} 2206 46 — 282.44 MHz

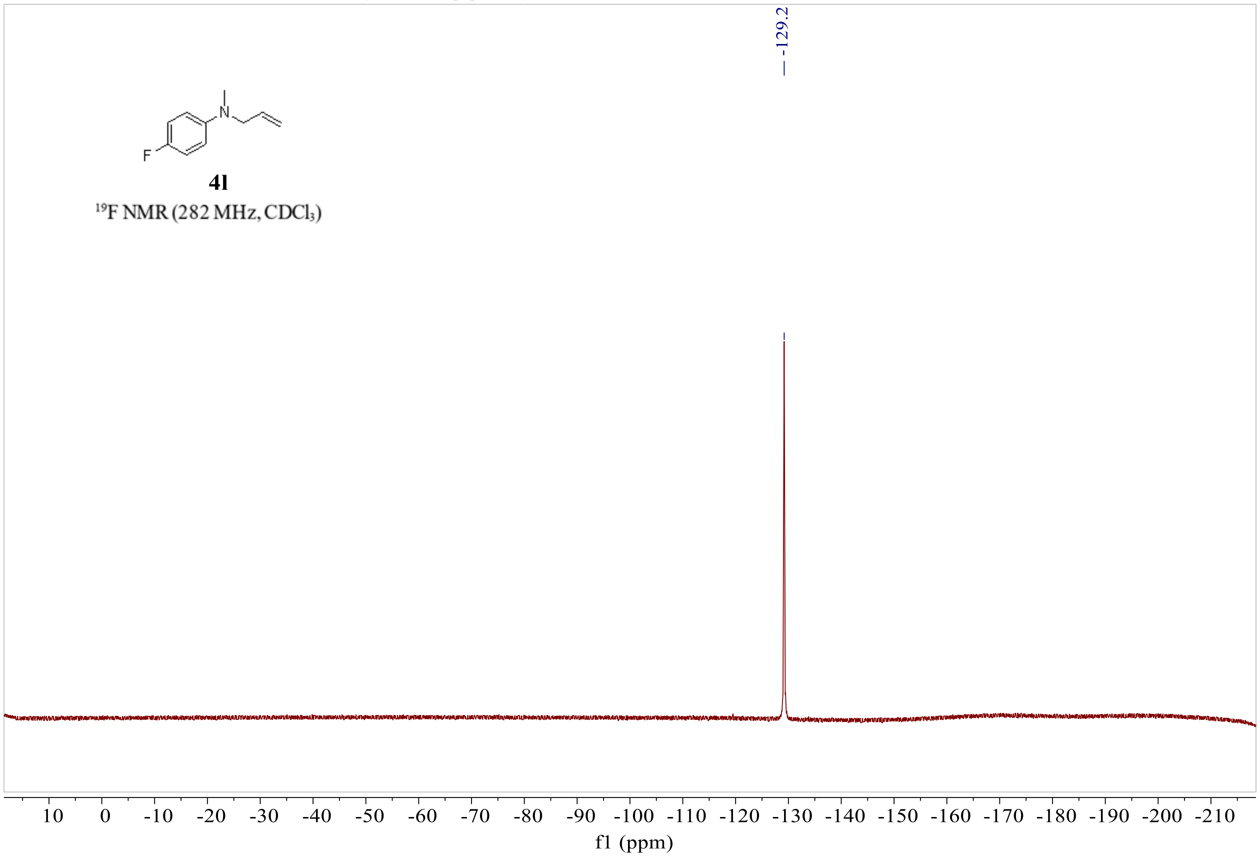

220622.306.10.fid — Fengqian Zhao zy-196-11 — Au1H CDCl3 {C:\Bruker\TopSpin3.6.2} 2206 6 — 300.13 MHz

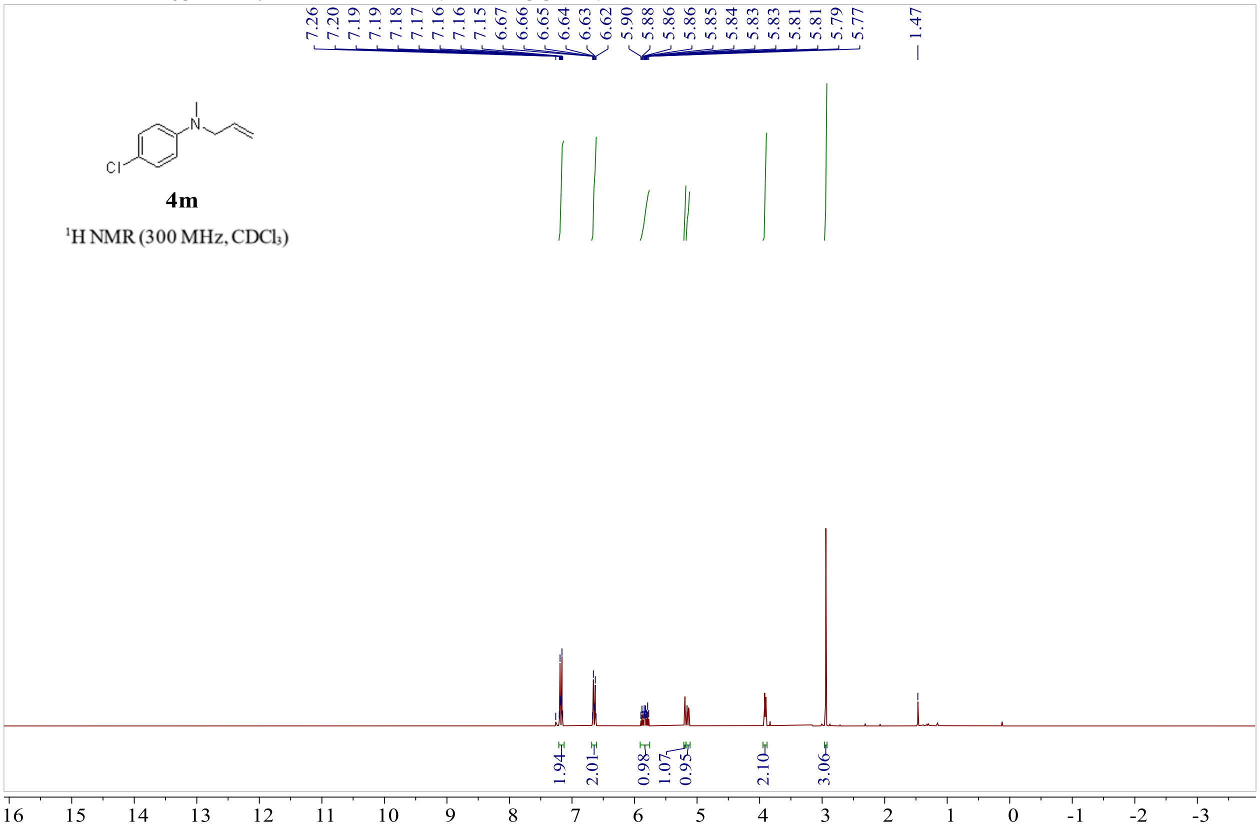

220622.306.11.fid — Fengqian Zhao zy-196-11 — Au13C CDCl3 {C:\Bruker\TopSpin3.6.2} 2206 6 — 75.48 MHz

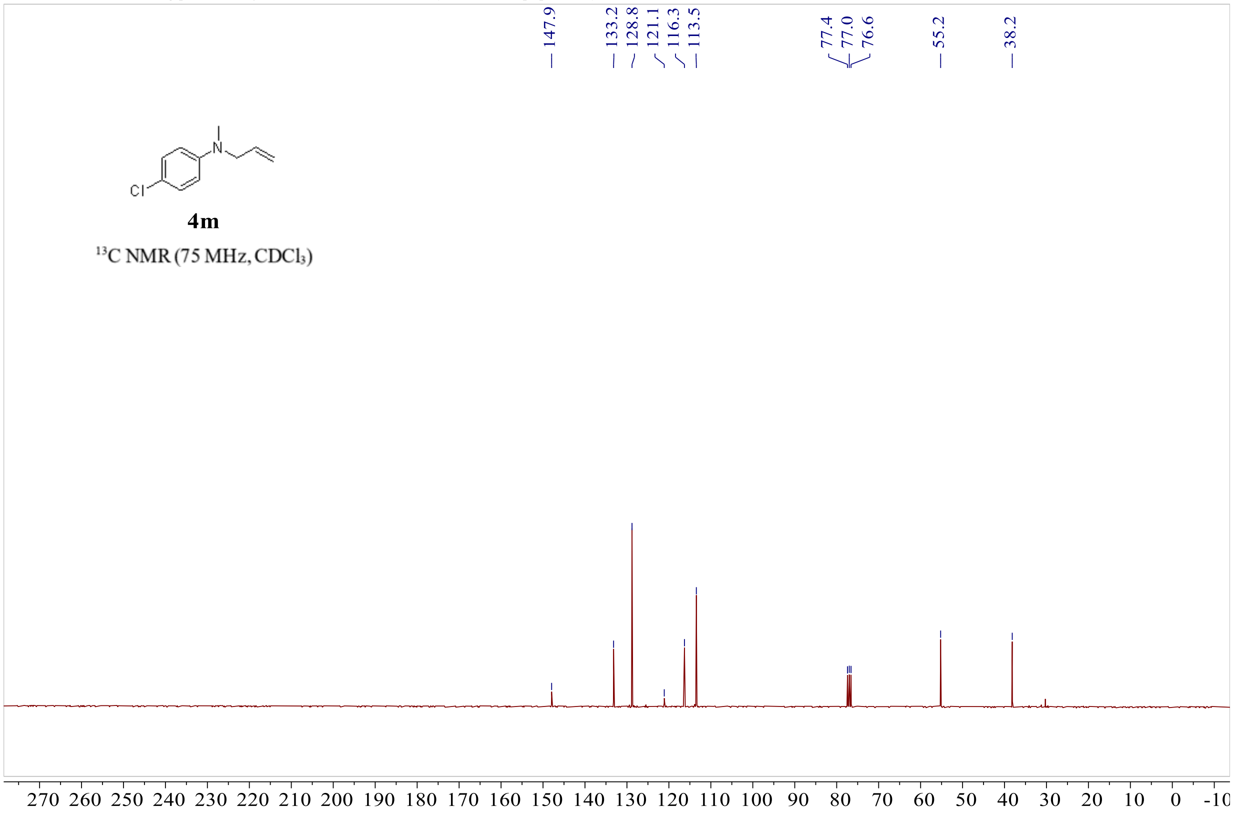

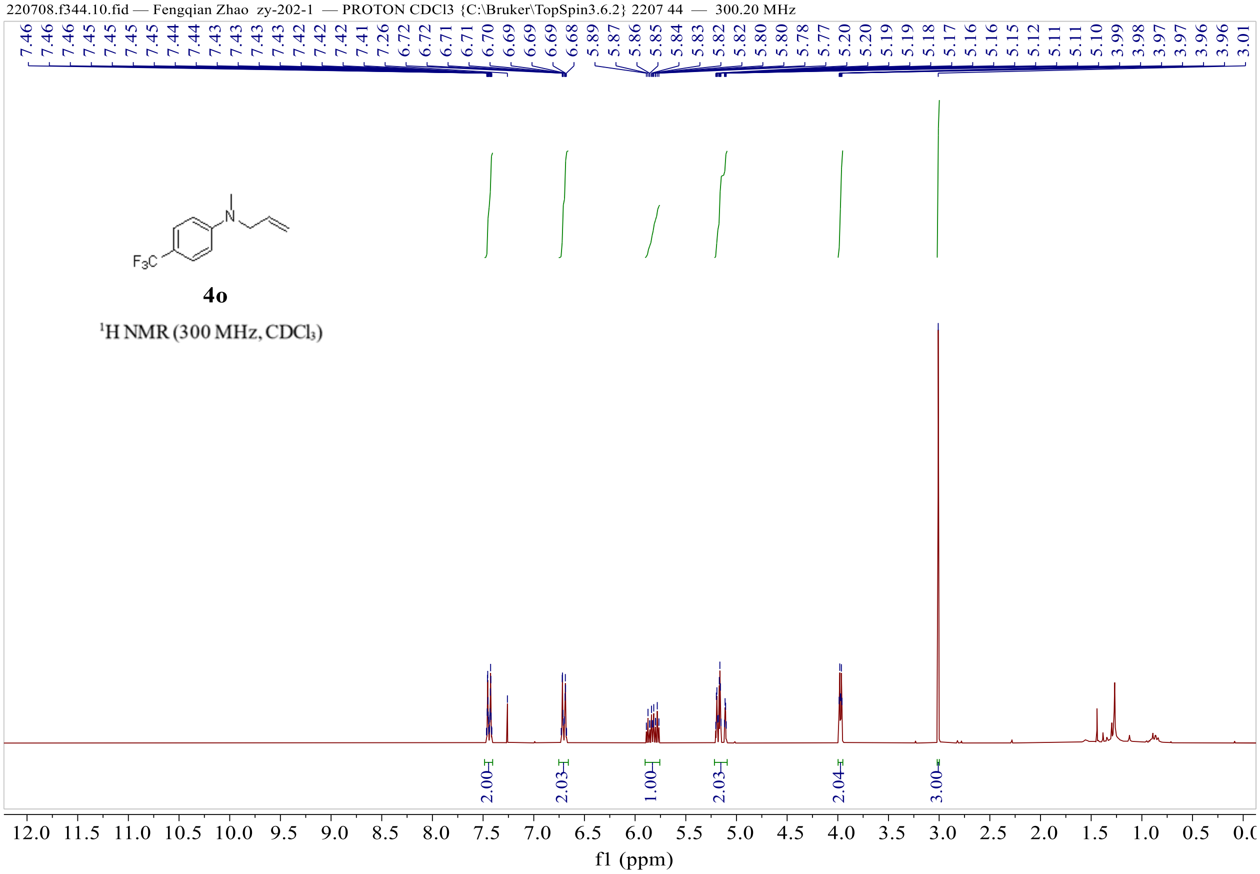

220708.f344.10.fid — Fengqian Zhao zy-202-1 — PROTON CDCl3 {C:\Bruker\TopSpin3.6.2} 2207 44 — 300.20 MHz
7.46 7.46 7.46 7.45 7.45 7.45 7.45 7.44 7.44 7.43 7.43 7.43 7.43 7.42 7.42 7.42 7.41 7.26 6.72 6.72 6.71 6.71 6.70 6.69 6.69 6.69 6.68 5.89 5.87 5.86 5.85 5.84 5.83 5.82 5.82 5.80 5.80 5.78 5.77 5.20 5.20 5.19 5.19 5.18 5.17 5.16 5.15 5.12 5.11 5.11 5.10 3.99 3.98 3.97 3.97 3.96 3.96 3.01
F3C
N
4o
1H NMR (300 MHz, CDCl3)
2.00
2.03
1.00
2.03
2.04
3.00
12.0 11.5 11.0 10.5 10.0 9.5 9.0 8.5 8.0 7.5 7.0 6.5 6.0 5.5 5.0 4.5 4.0 3.5 3.0 2.5 2.0 1.5 1.0 0.5 0.0
f1 (ppm)

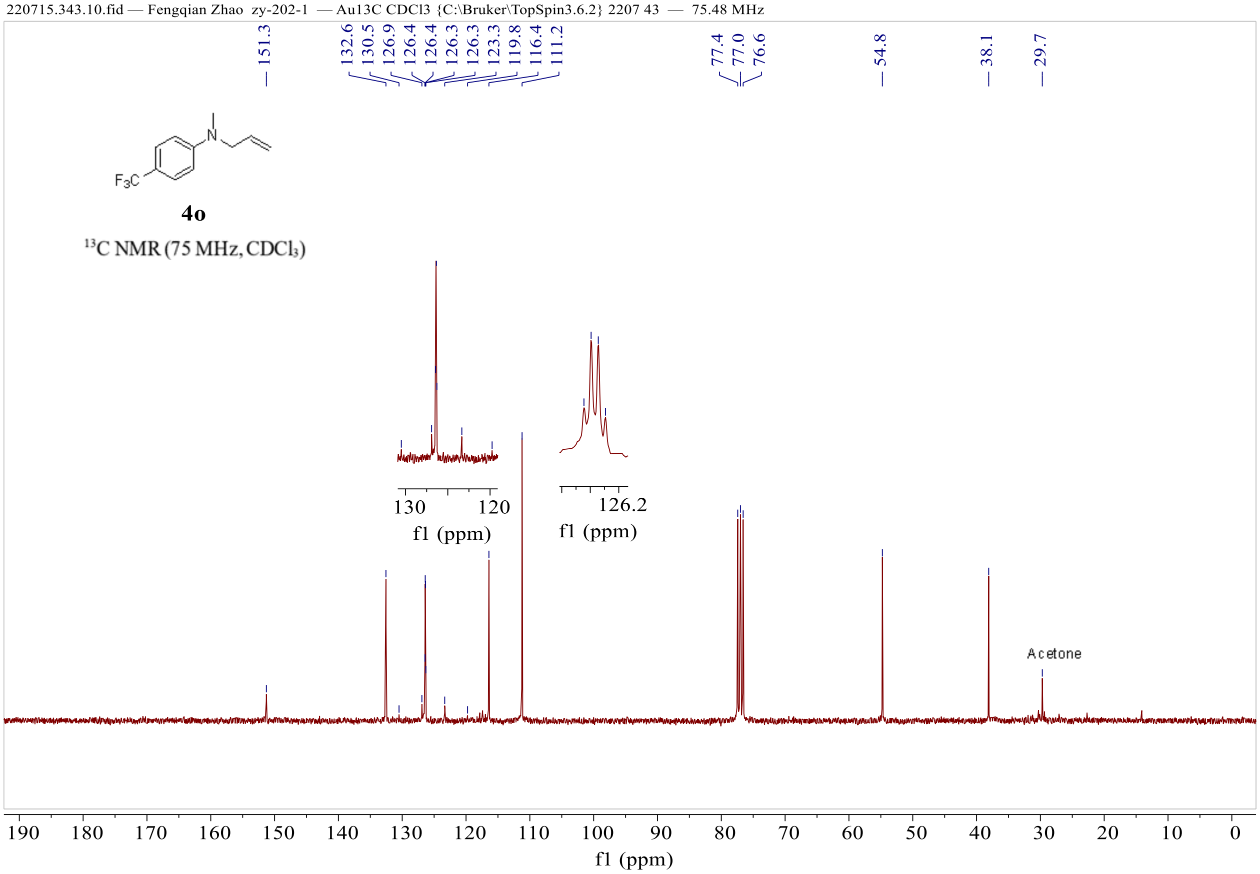

220715.343.10.fid — Fengqian Zhao zy-202-1 — Au13C CDCl3 {C:\Bruker\TopSpin3.6.2} 2207 43 — 75.48 MHz
151.3
132.6 130.5 126.9 126.4 126.4 126.3 126.3 123.3 119.8 116.4 111.2
77.4 77.0 76.6
54.8
38.1
29.7
F3C
N
4o
13C NMR (75 MHz, CDCl3)
130
120
f1 (ppm)
126.2
f1 (ppm)
Acetone
190 180 170 160 150 140 130 120 110 100 90 80 70 60 50 40 30 20 10 0
f1 (ppm)

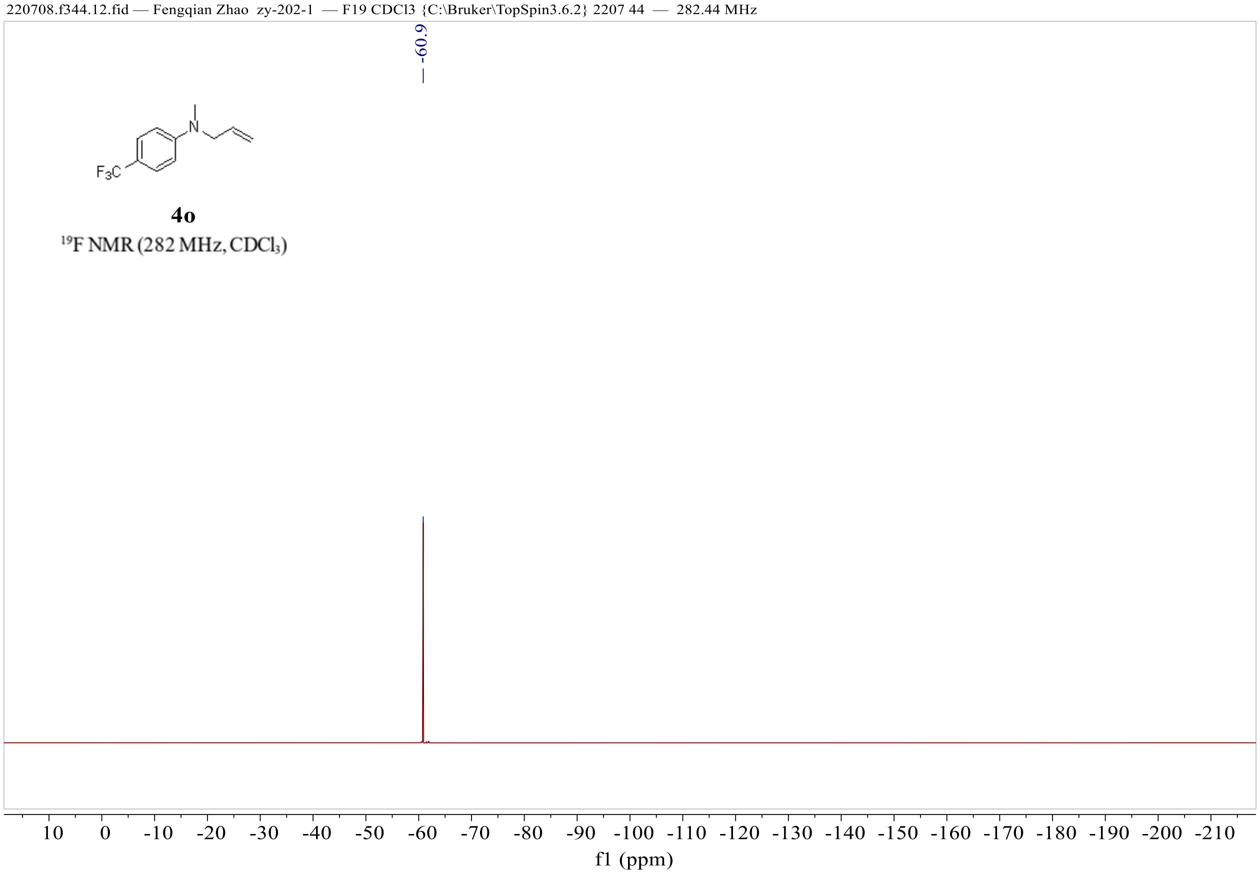
220708.f344.12.fid — Fengqian Zhao zy-202-1 — F19 CDCl3 {C:\Bruker\TopSpin3.6.2} 2207 44 — 282.44 MHz
-60.9
F_3C
N
4o
^{19}F NMR (282 MHz, $CDCl_3$)
10 0 -10 -20 -30 -40 -50 -60 -70 -80 -90 -100 -110 -120 -130 -140 -150 -160 -170 -180 -190 -200 -210
f1 (ppm)

220628.f344.10.fid — Zhao/ ZY-200-3 — PROTON CDCl3 {C:\Bruker\TopSpin3.6.2} 2206 44 — 300.20 MHz

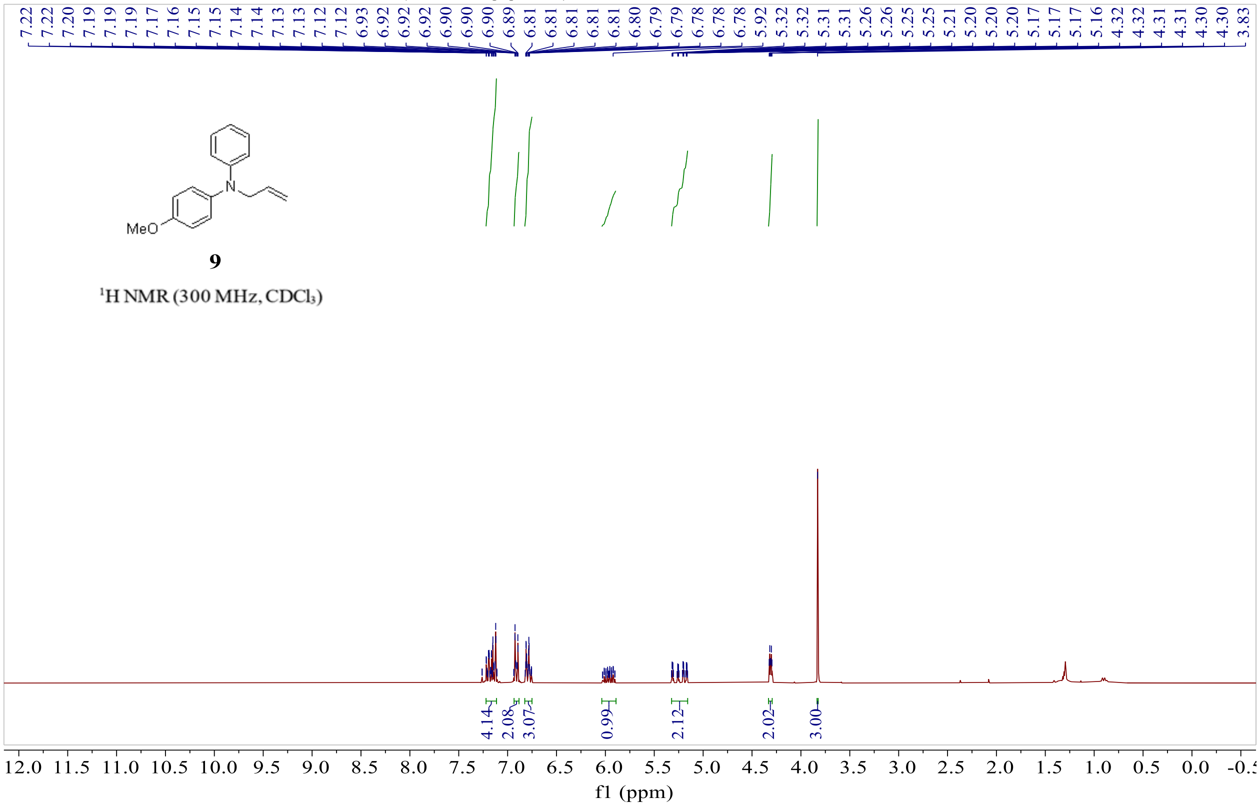

9

^{1}H NMR (300 MHz, $CDCl_3$)

220628.f344.11.fid — Zhao/ ZY-200-3 — C13CPD CDCl3 {C:\Bruker\TopSpin3.6.2} 2206 44 — 75.49 MHz

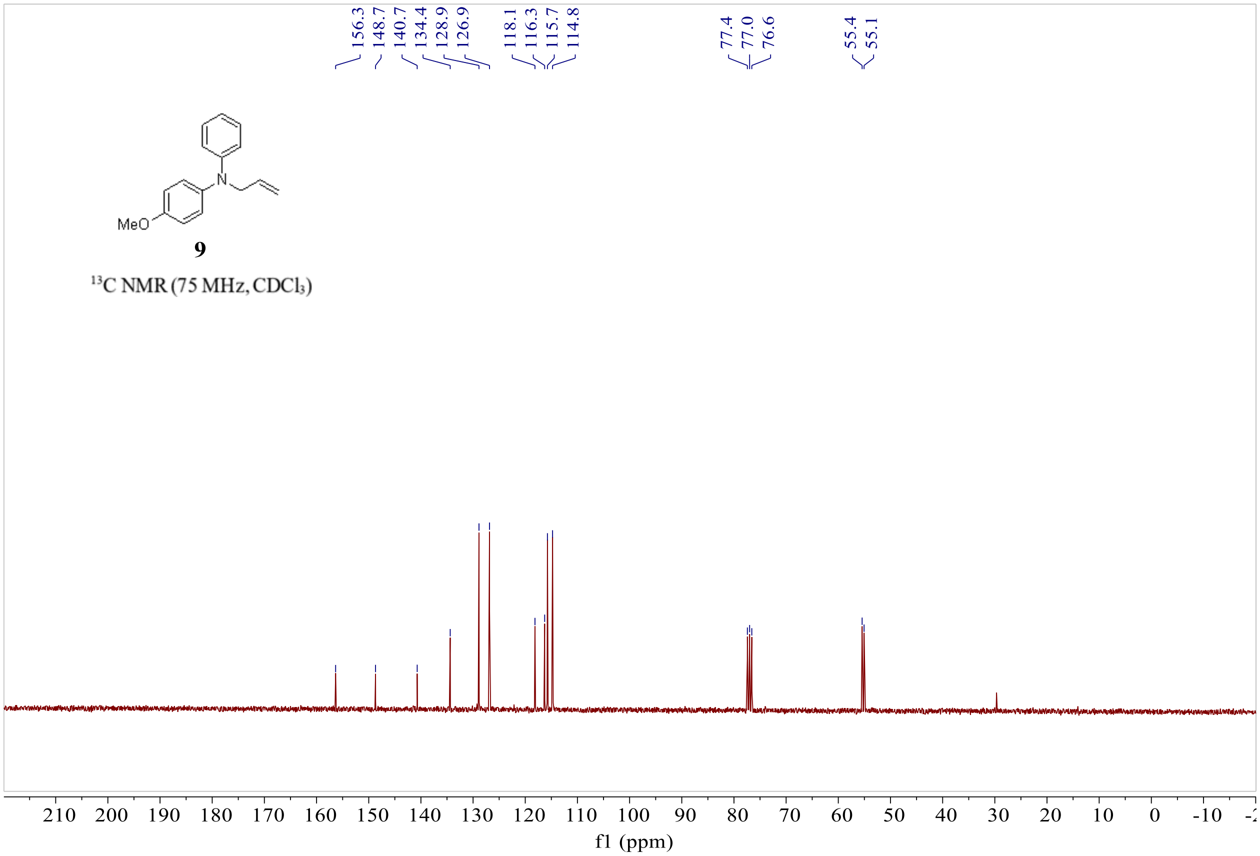

9

^{13}C NMR (75 MHz, $CDCl_3$)

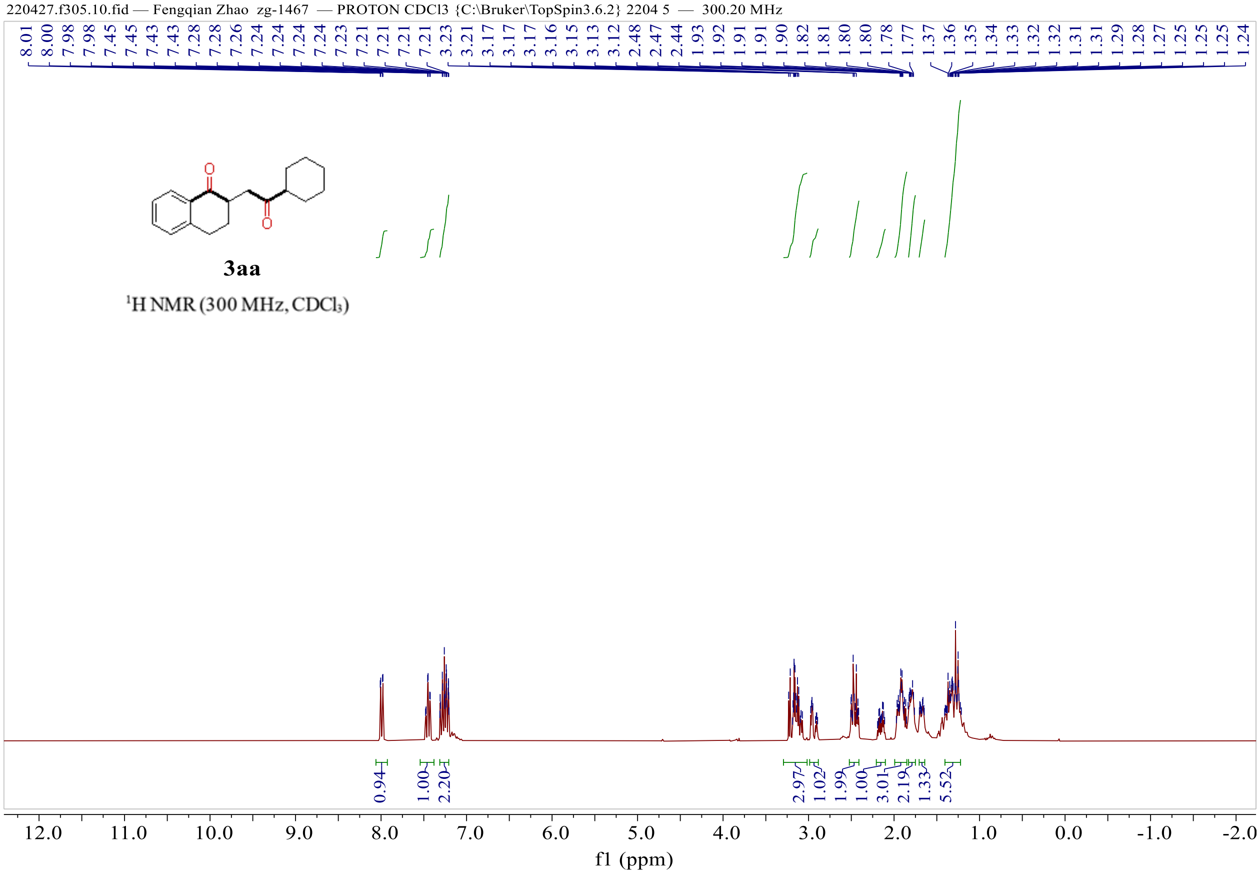
220427.f305.10.fid — Fengqian Zhao zg-1467 — PROTON CDCl3 {C:\Bruker\TopSpin3.6.2} 2204 5 — 300.20 MHz
8.01 8.00 7.98 7.98 7.45 7.45 7.43 7.43 7.28 7.28 7.26 7.24 7.24 7.24 7.24 7.23 7.21 7.21 7.21 7.21 3.23 3.21 3.17 3.17 3.17 3.16 3.15 3.13 3.12 2.48 2.47 2.44 1.93 1.92 1.91 1.91 1.90 1.82 1.81 1.80 1.80 1.78 1.77 1.37 1.36 1.35 1.34 1.33 1.32 1.32 1.31 1.31 1.29 1.28 1.27 1.25 1.25 1.25 1.24
3aa
1H NMR (300 MHz, CDCl3)
0.94 1.00 2.20 2.97 1.02 1.99 1.00 3.01 2.19 1.33 5.52
12.0 11.0 10.0 9.0 8.0 7.0 6.0 5.0 4.0 3.0 2.0 1.0 0.0 -1.0 -2.0
f1 (ppm)

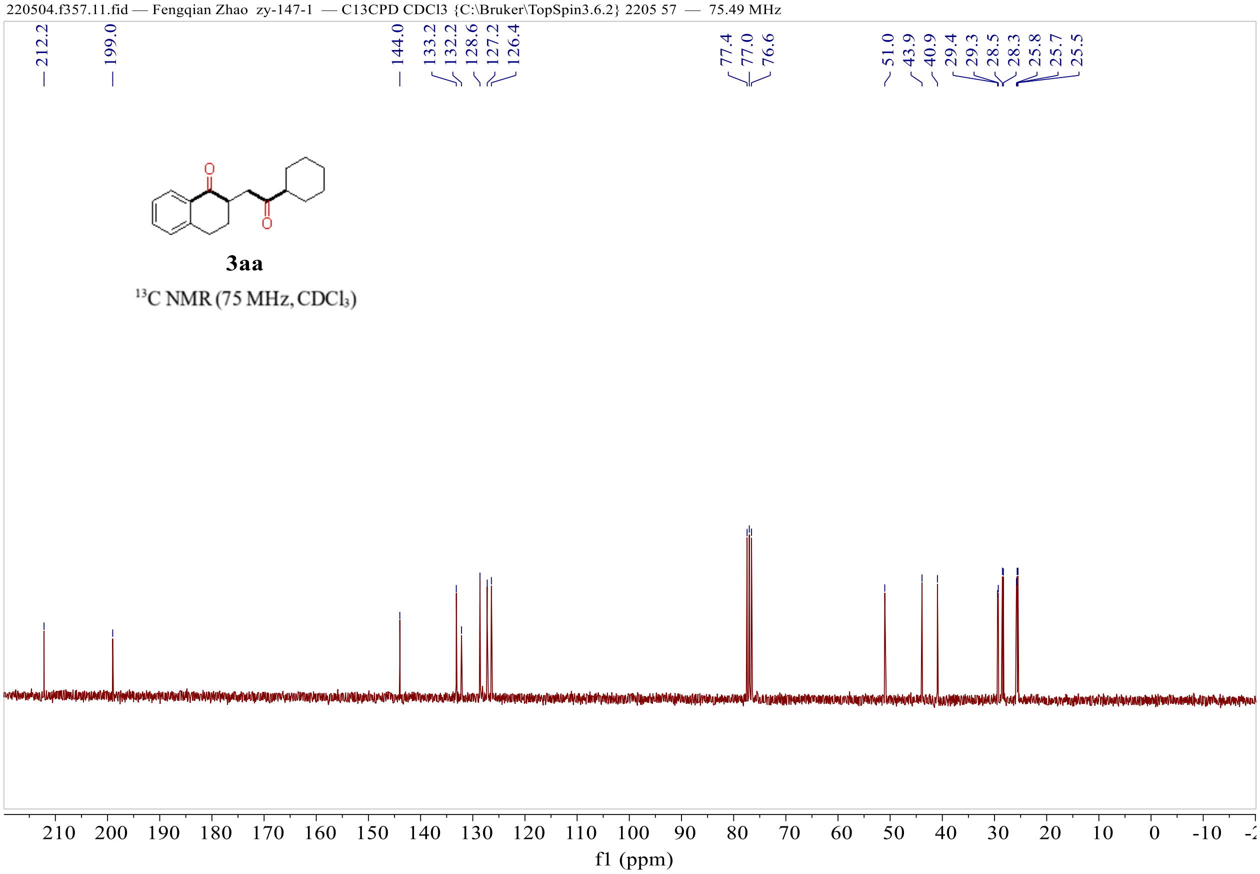
220504.f357.11.fid — Fengqian Zhao zy-147-1 — C13CPD CDCl3 {C:\Bruker\TopSpin3.6.2} 2205 57 — 75.49 MHz
212.2 199.0 144.0 133.2 132.2 128.6 127.2 126.4 77.4 77.0 76.6 51.0 43.9 40.9 29.4 29.3 28.5 28.3 25.8 25.7 25.5
3aa
13C NMR (75 MHz, CDCl3)
210 200 190 180 170 160 150 140 130 120 110 100 90 80 70 60 50 40 30 20 10 0 -10
f1 (ppm)

220517.333.10.fid — Patrizio Russo, 29-154-3 — Au1H CDCl3 {C:\Bruker\TopSpin3.6.2} 2205 33 — 300.13 MHz

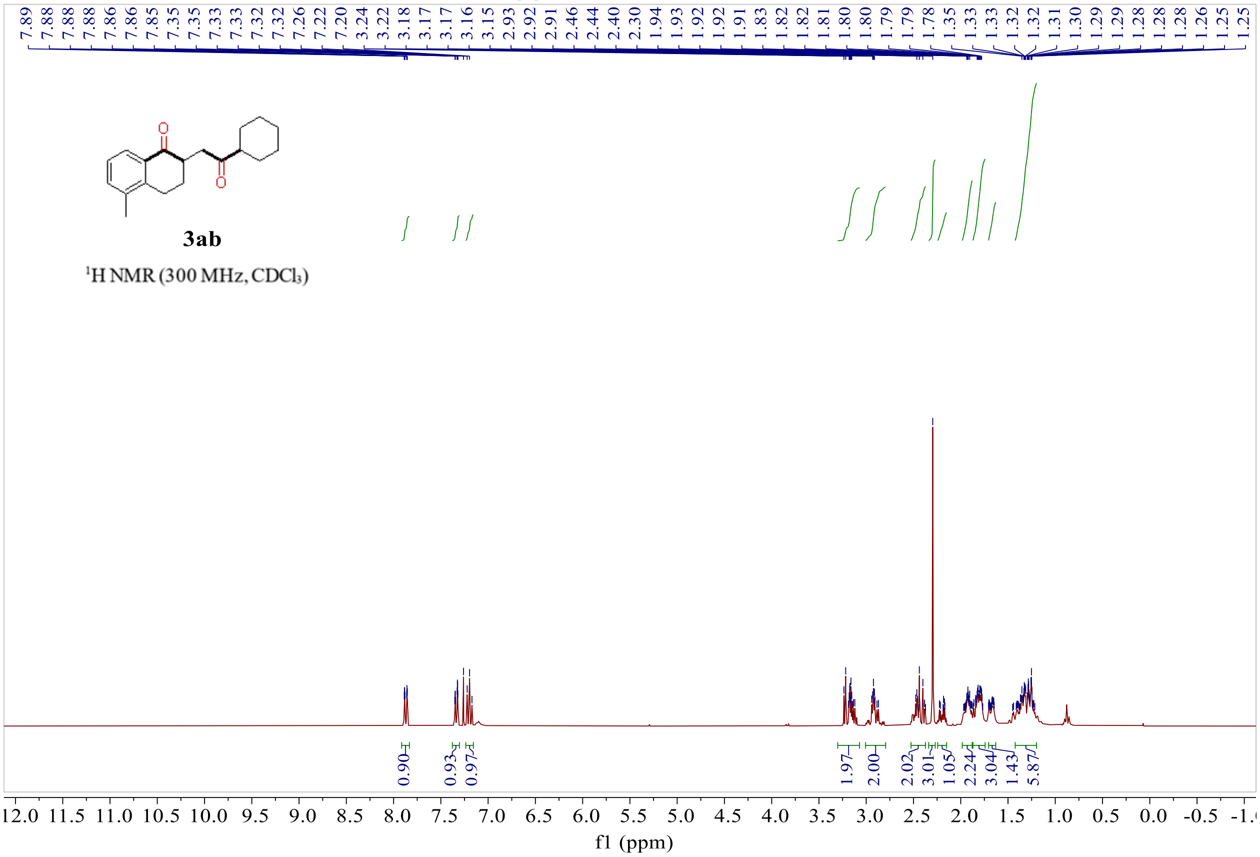

220517.333.11.fid — Patrizio Russo, 29-154-3 — Au13C CDCl3 {C:\Bruker\TopSpin3.6.2} 2205 33 — 75.48 MHz

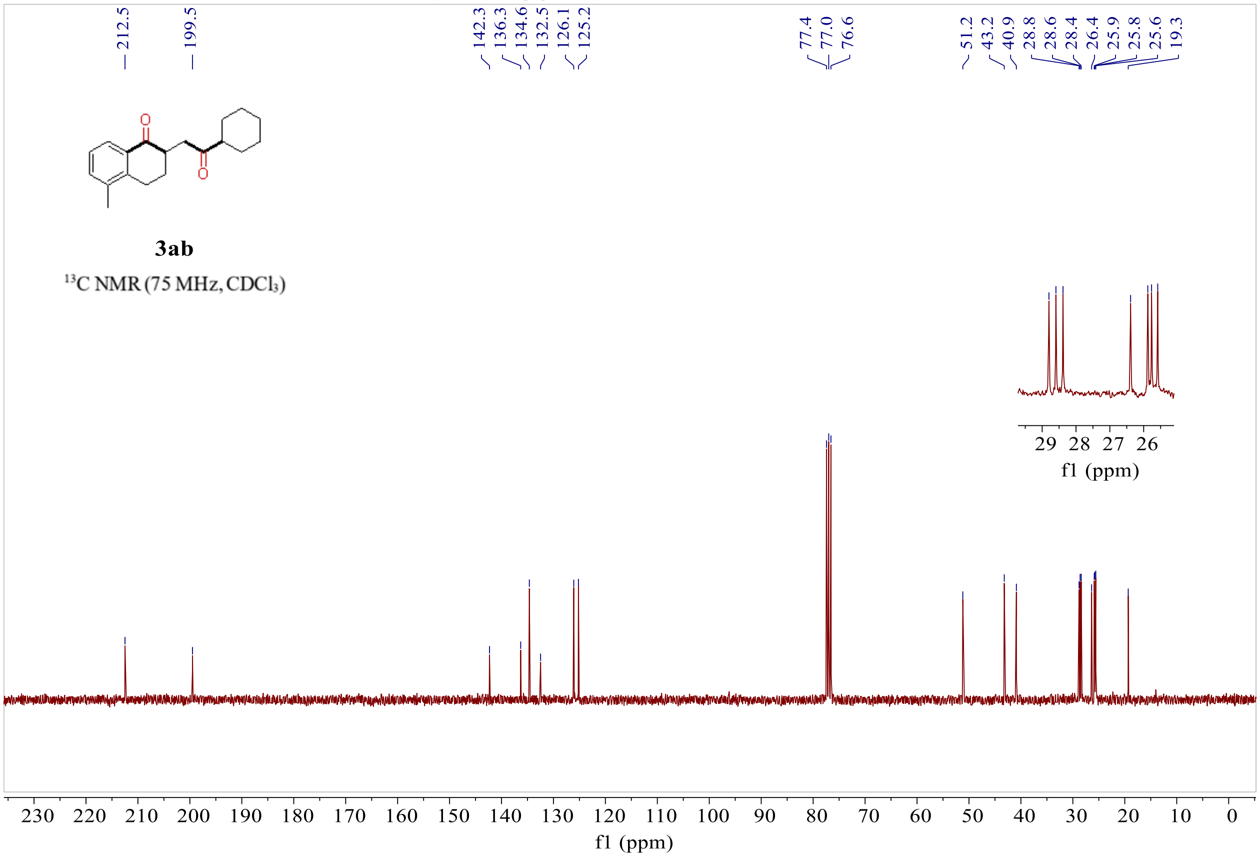

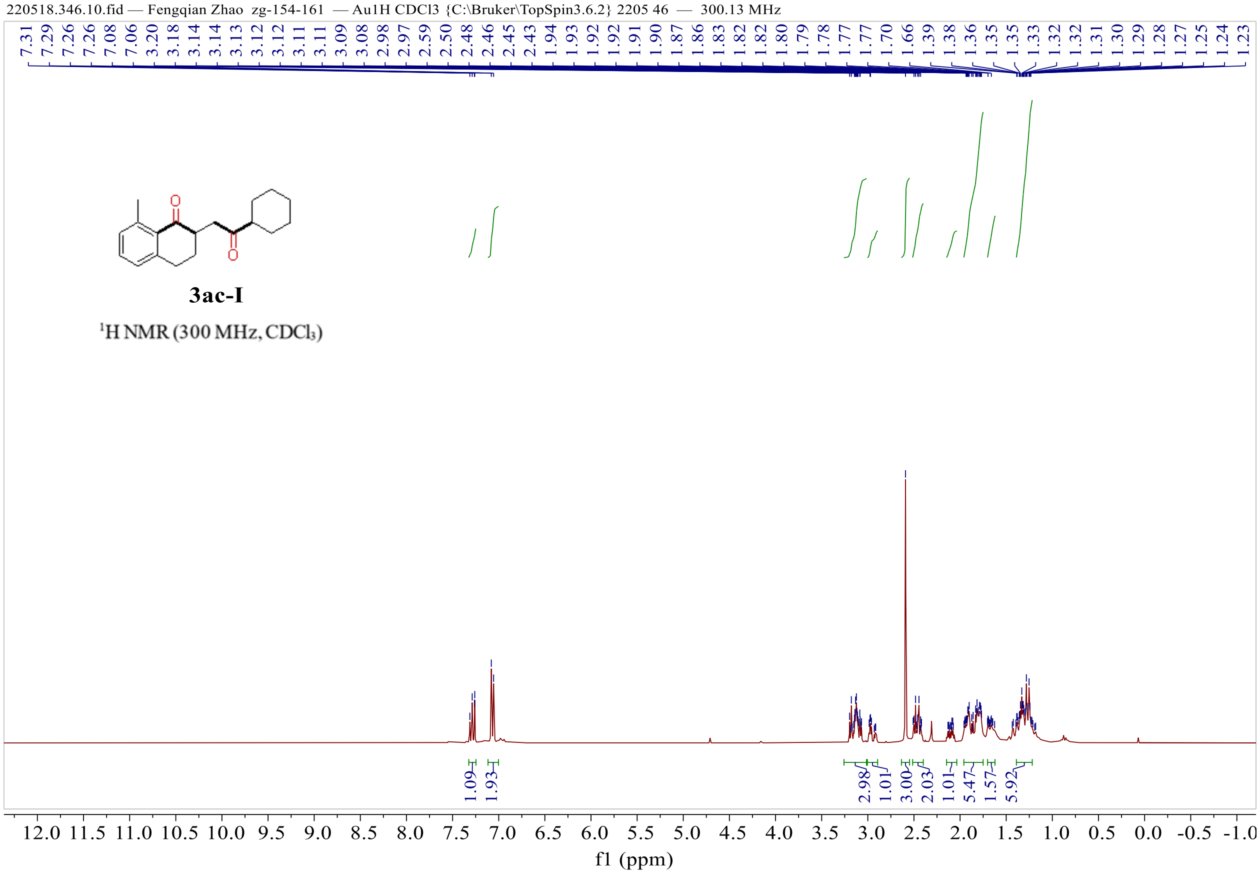
220518.346.10.fid — Fengqian Zhao zg-154-161 — Au1H CDCl3 {C:\Bruker\TopSpin3.6.2} 2205 46 — 300.13 MHz
3ac-I
^{1}H NMR (300 MHz, CDCl$_3$)
f1 (ppm)

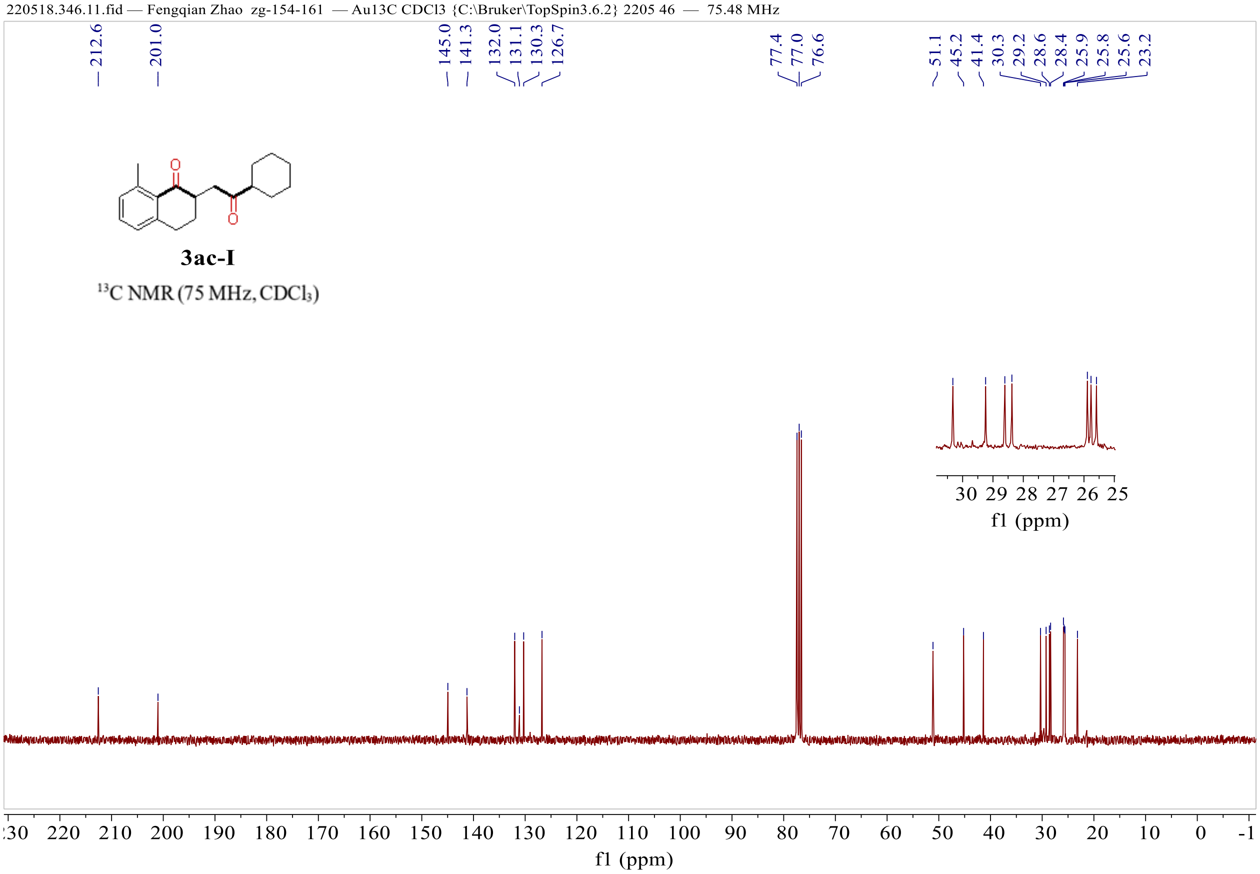
220518.346.11.fid — Fengqian Zhao zg-154-161 — Au13C CDCl3 {C:\Bruker\TopSpin3.6.2} 2205 46 — 75.48 MHz
212.6
201.0
145.0
141.3
132.0
131.1
130.3
126.7
77.4
77.0
76.6
51.1
45.2
41.4
30.3
29.2
28.6
28.4
25.9
25.8
25.6
23.2
3ac-I
^{13}C NMR (75 MHz, CDCl$_3$)
f1 (ppm)

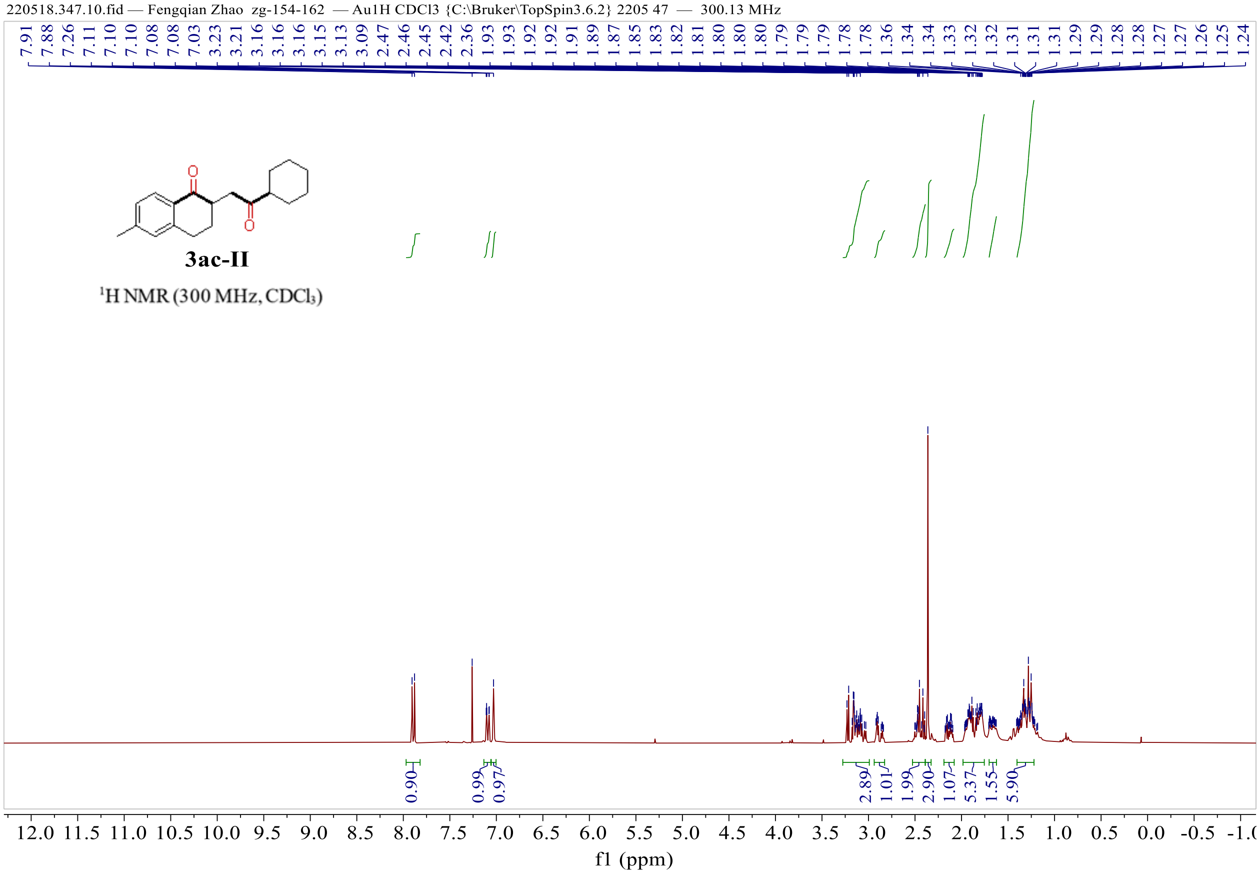
220518.347.10.fid — Fengqian Zhao zg-154-162 — Au1H CDCl3 {C:\Bruker\TopSpin3.6.2} 2205 47 — 300.13 MHz
3ac-II
1H NMR (300 MHz, CDCl3)
f1 (ppm)

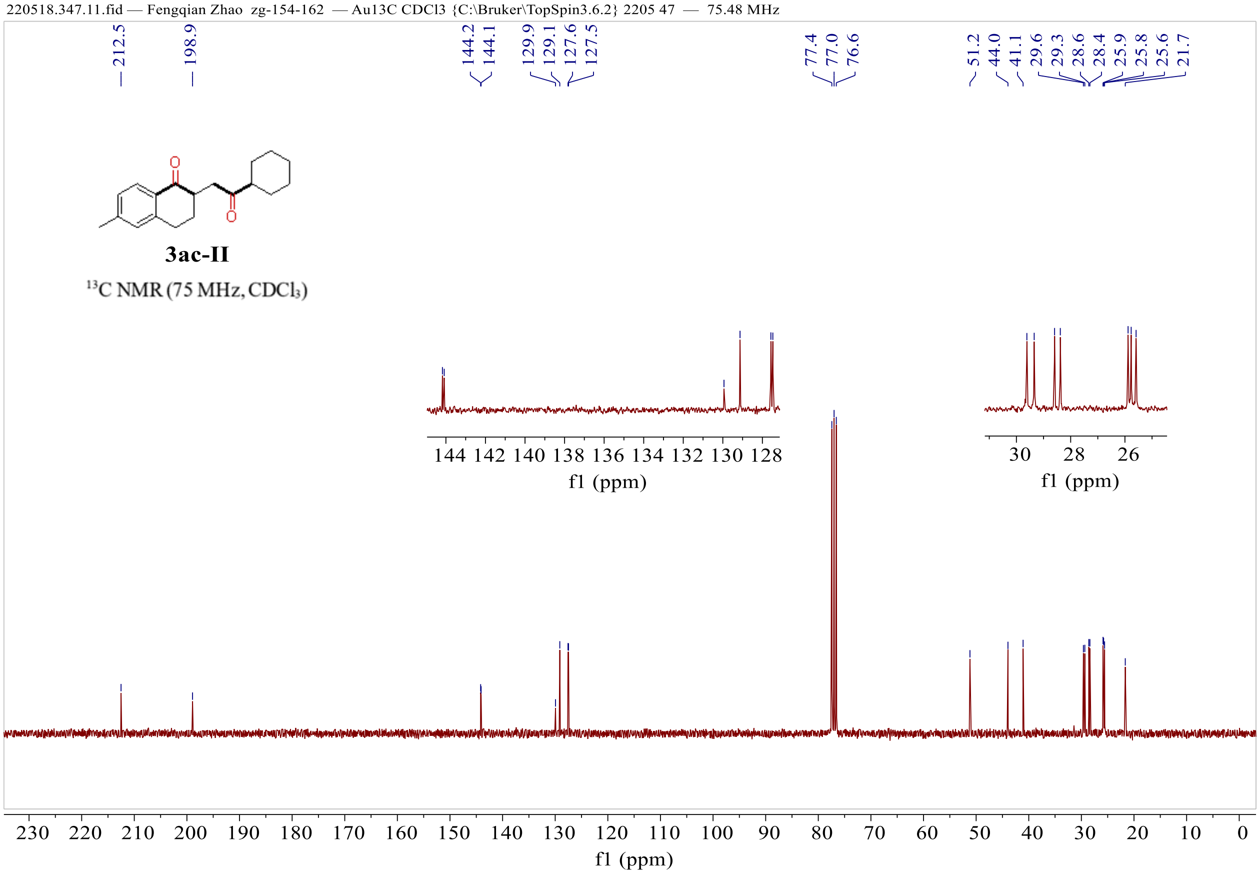
220518.347.11.fid — Fengqian Zhao zg-154-162 — Au13C CDCl3 {C:\Bruker\TopSpin3.6.2} 2205 47 — 75.48 MHz
212.5
198.9
144.2
144.1
129.9
129.1
127.6
127.5
77.4
77.0
76.6
51.2
44.0
41.1
29.6
29.3
28.6
28.4
25.9
25.8
25.6
21.7
3ac-II
13C NMR (75 MHz, CDCl3)
f1 (ppm)

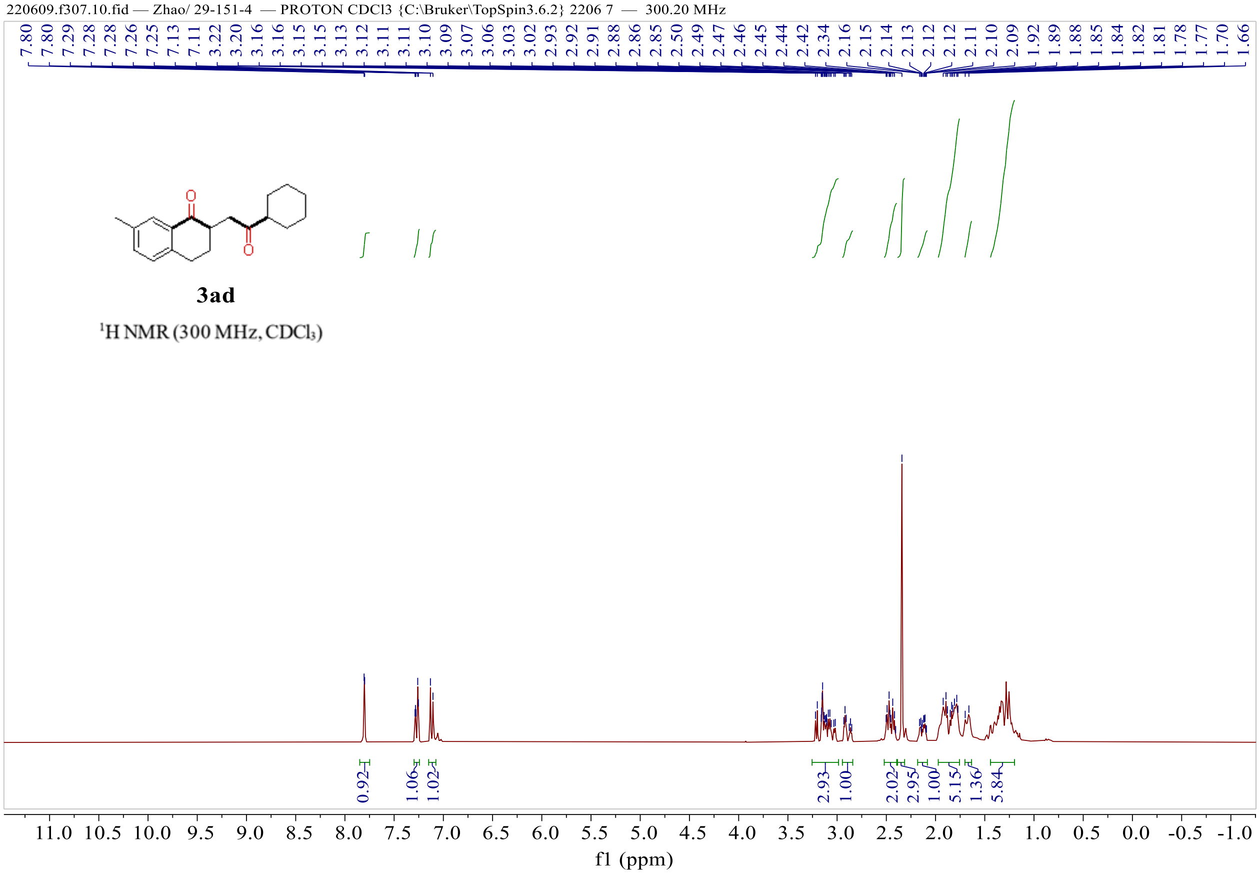
220609.f307.10.fid — Zhao/ 29-151-4 — PROTON CDCl3 {C:\Bruker\TopSpin3.6.2} 2206 7 — 300.20 MHz
3ad
¹H NMR (300 MHz, CDCl₃)
f1 (ppm)

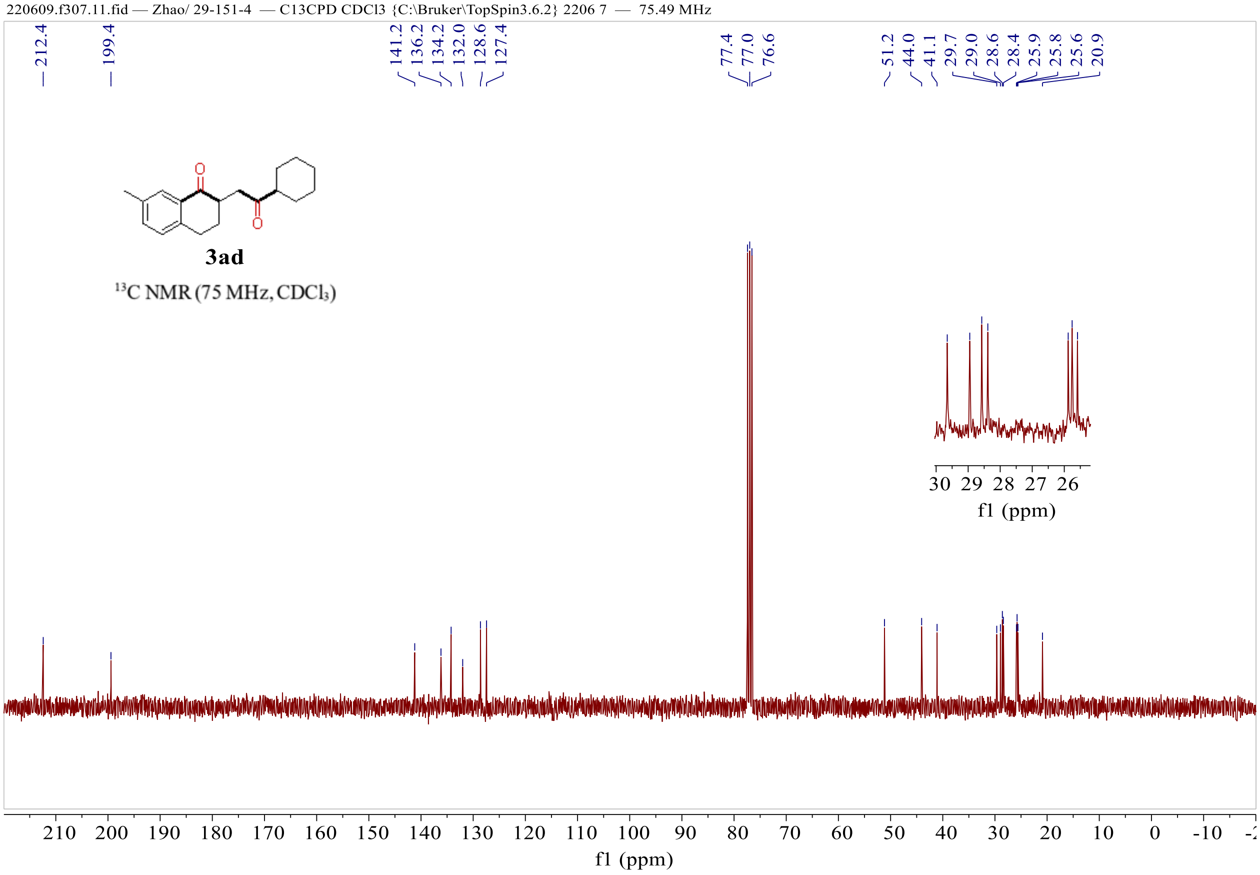
220609.f307.11.fid — Zhao/ 29-151-4 — C13CPD CDCl3 {C:\Bruker\TopSpin3.6.2} 2206 7 — 75.49 MHz
212.4
199.4
141.2
136.2
134.2
132.0
128.6
127.4
77.4
77.0
76.6
51.2
44.0
41.1
29.7
29.0
28.6
28.4
25.9
25.8
25.6
20.9
3ad
¹³C NMR (75 MHz, CDCl₃)
f1 (ppm)

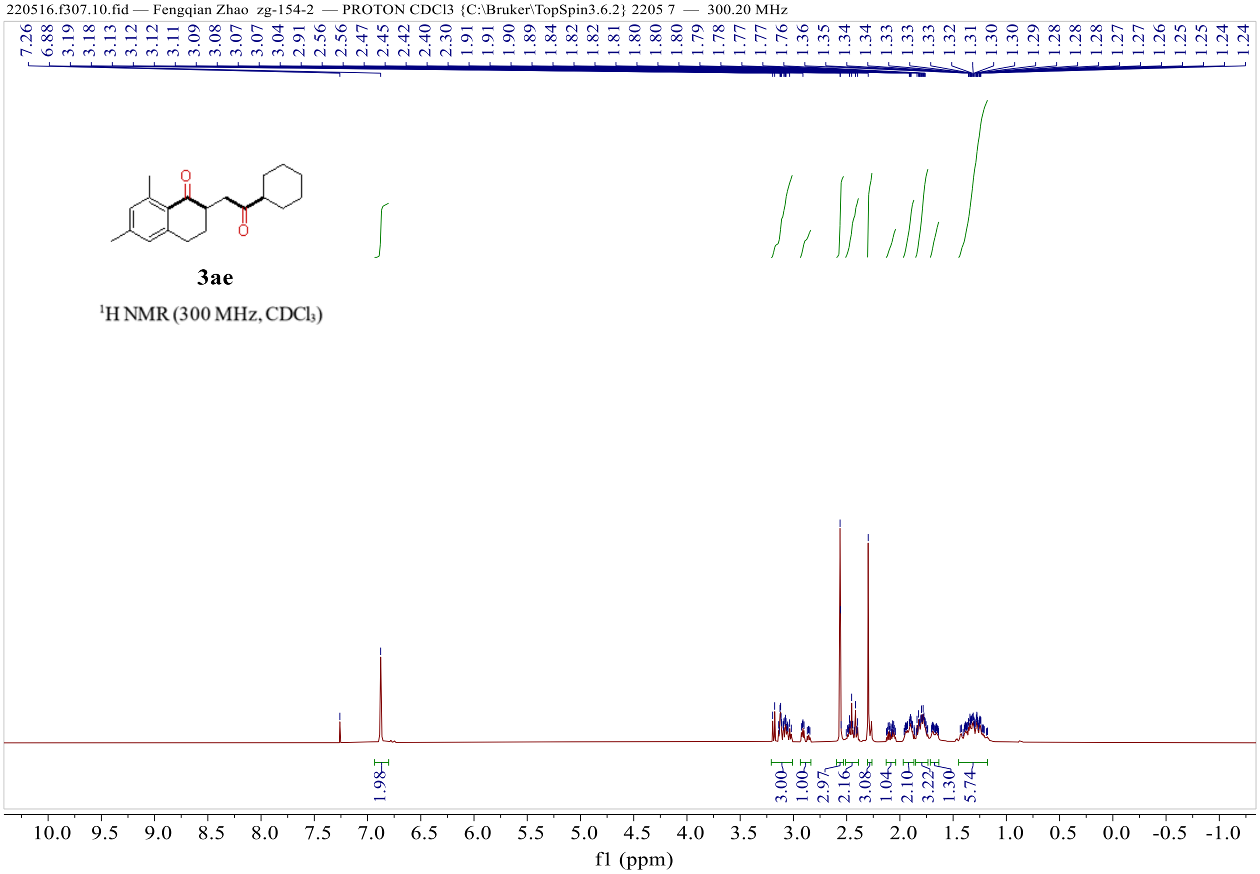
220516.f307.10.fid — Fengqian Zhao zg-154-2 — PROTON CDCl3 {C:\Bruker\TopSpin3.6.2} 2205 7 — 300.20 MHz
7.26 6.88 3.19 3.18 3.13 3.12 3.12 3.11 3.09 3.08 3.07 3.07 3.04 2.91 2.56 2.56 2.47 2.45 2.42 2.40 2.30 1.91 1.91 1.90 1.89 1.84 1.82 1.82 1.81 1.80 1.80 1.80 1.79 1.78 1.77 1.77 1.76 1.36 1.35 1.34 1.34 1.33 1.33 1.33 1.32 1.31 1.30 1.30 1.29 1.28 1.28 1.28 1.27 1.27 1.26 1.25 1.25 1.24 1.24
O
O
3ae
1H NMR (300 MHz, CDCl3)
1.98
3.00 1.00 2.97 2.16 3.08 1.04 2.10 3.22 1.30 5.74
10.0 9.5 9.0 8.5 8.0 7.5 7.0 6.5 6.0 5.5 5.0 4.5 4.0 3.5 3.0 2.5 2.0 1.5 1.0 0.5 0.0 -0.5 -1.0
f1 (ppm)

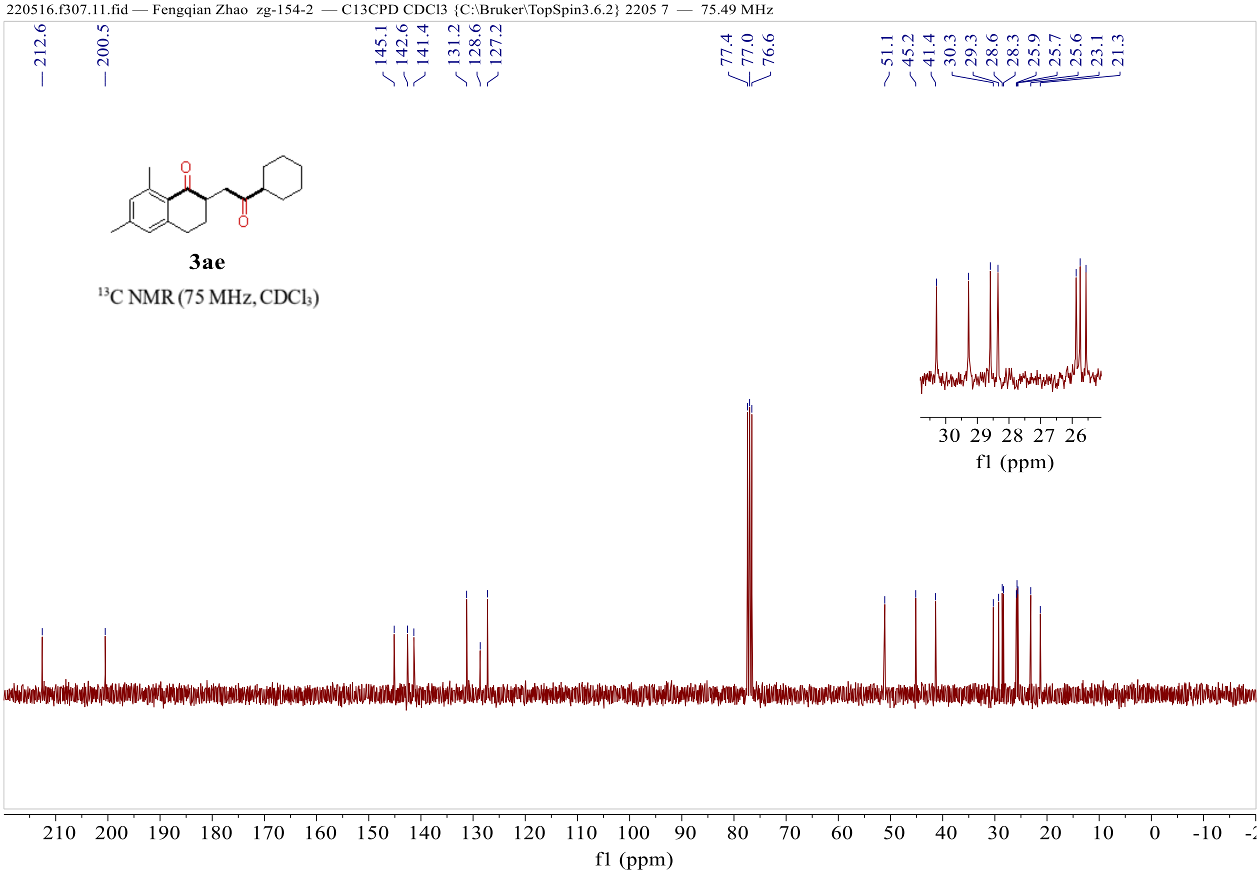
220516.f307.11.fid — Fengqian Zhao zg-154-2 — C13CPD CDCl3 {C:\Bruker\TopSpin3.6.2} 2205 7 — 75.49 MHz
212.6 200.5 145.1 142.6 141.4 131.2 128.6 127.2 77.4 77.0 76.6 51.1 45.2 41.4 30.3 29.3 28.6 28.3 25.9 25.7 25.6 23.1 21.3
O
O
3ae
13C NMR (75 MHz, CDCl3)
30 29 28 27 26
f1 (ppm)
210 200 190 180 170 160 150 140 130 120 110 100 90 80 70 60 50 40 30 20 10 0 -10
f1 (ppm)

220517.332.10.fid — Patrizio Russo, 29-152-12 — Au1H CDCl3 {C:\Bruker\TopSpin3.6.2} 2205 32 — 300.13 MHz

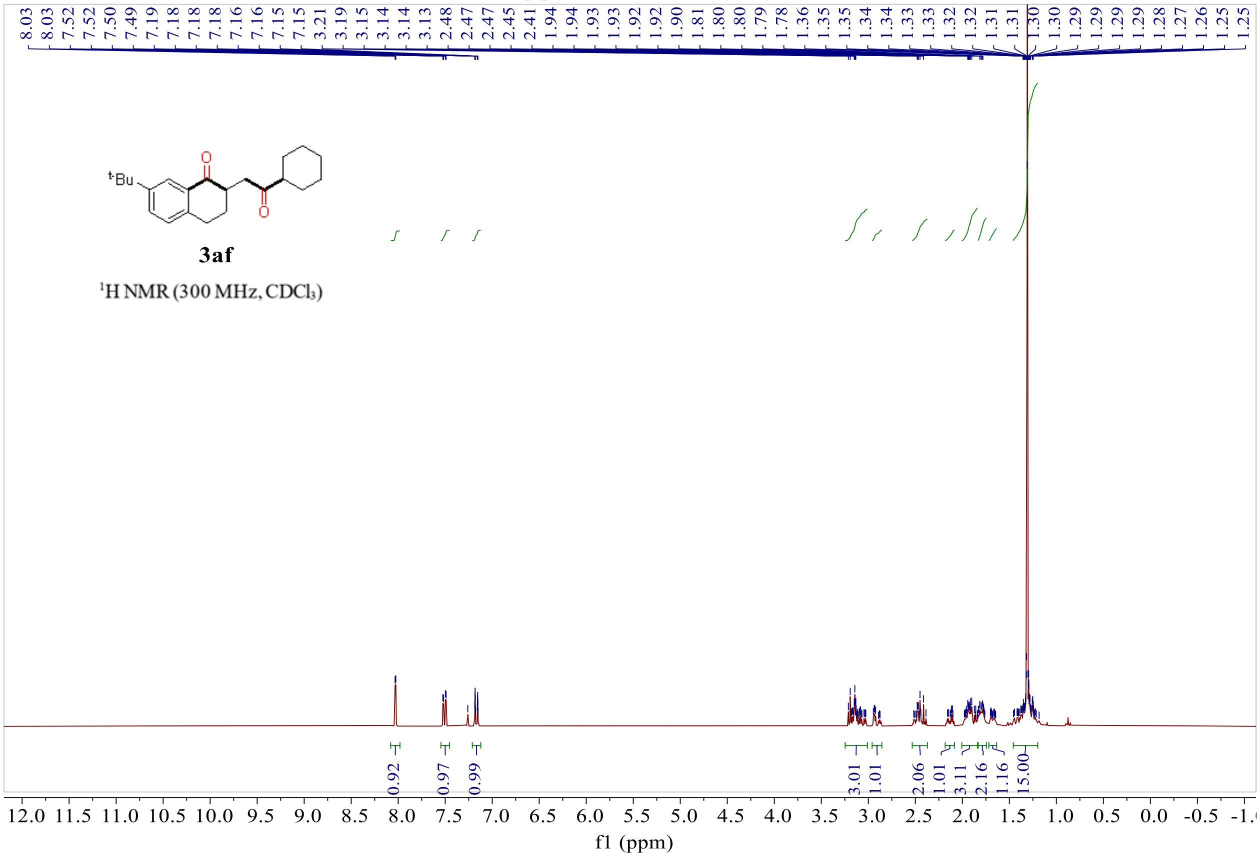

220517.332.11.fid — Patrizio Russo, 29-152-12 — Au13C CDCl3 {C:\Bruker\TopSpin3.6.2} 2205 32 — 75.48 MHz

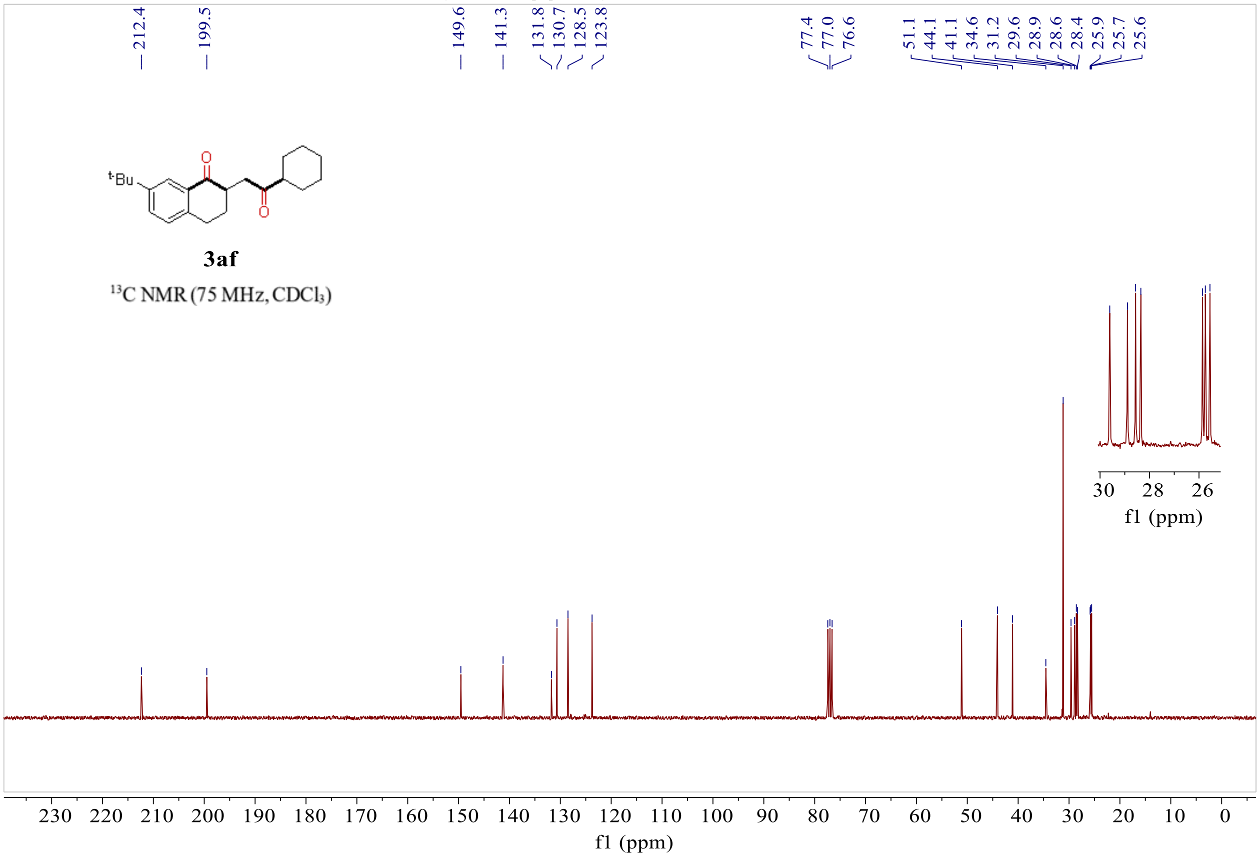

220502.f305.10.fid — Fengqian Zhao zg-147-8 — PROTON CDCl3 {C:\Bruker\TopSpin3.6.2} 2205 5 — 300.20 MHz

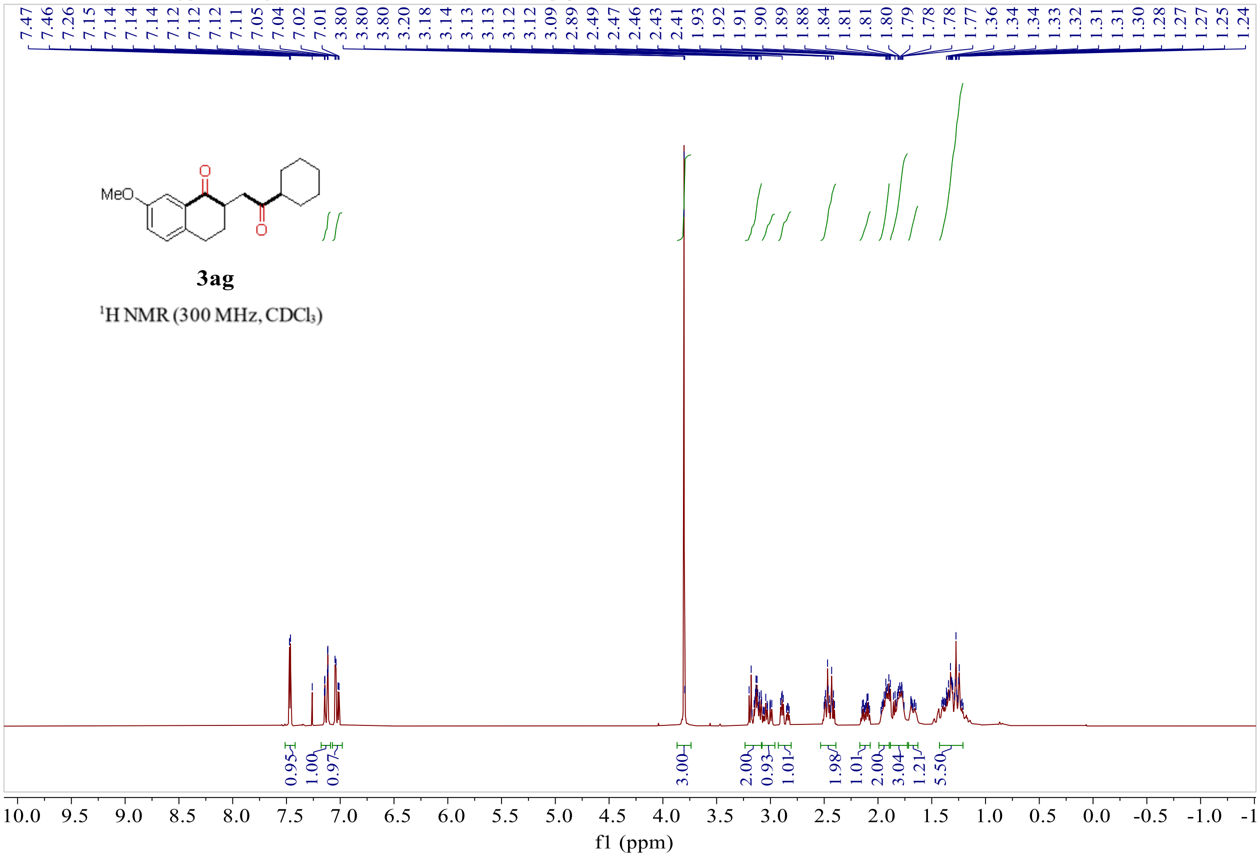

220502.f305.11.fid — Fengqian Zhao zg-147-8 — C13CPD CDCl3 {C:\Bruker\TopSpin3.6.2} 2205 5 — 75.49 MHz

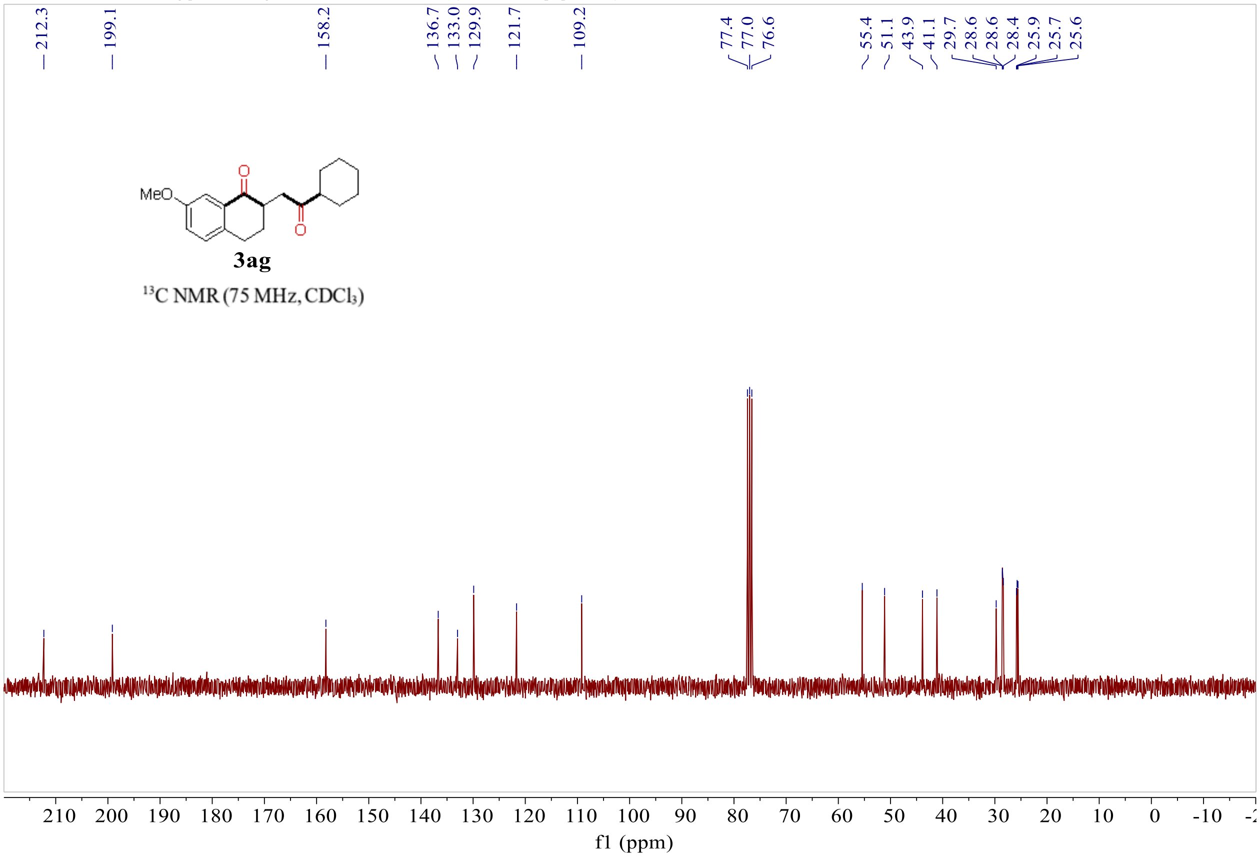

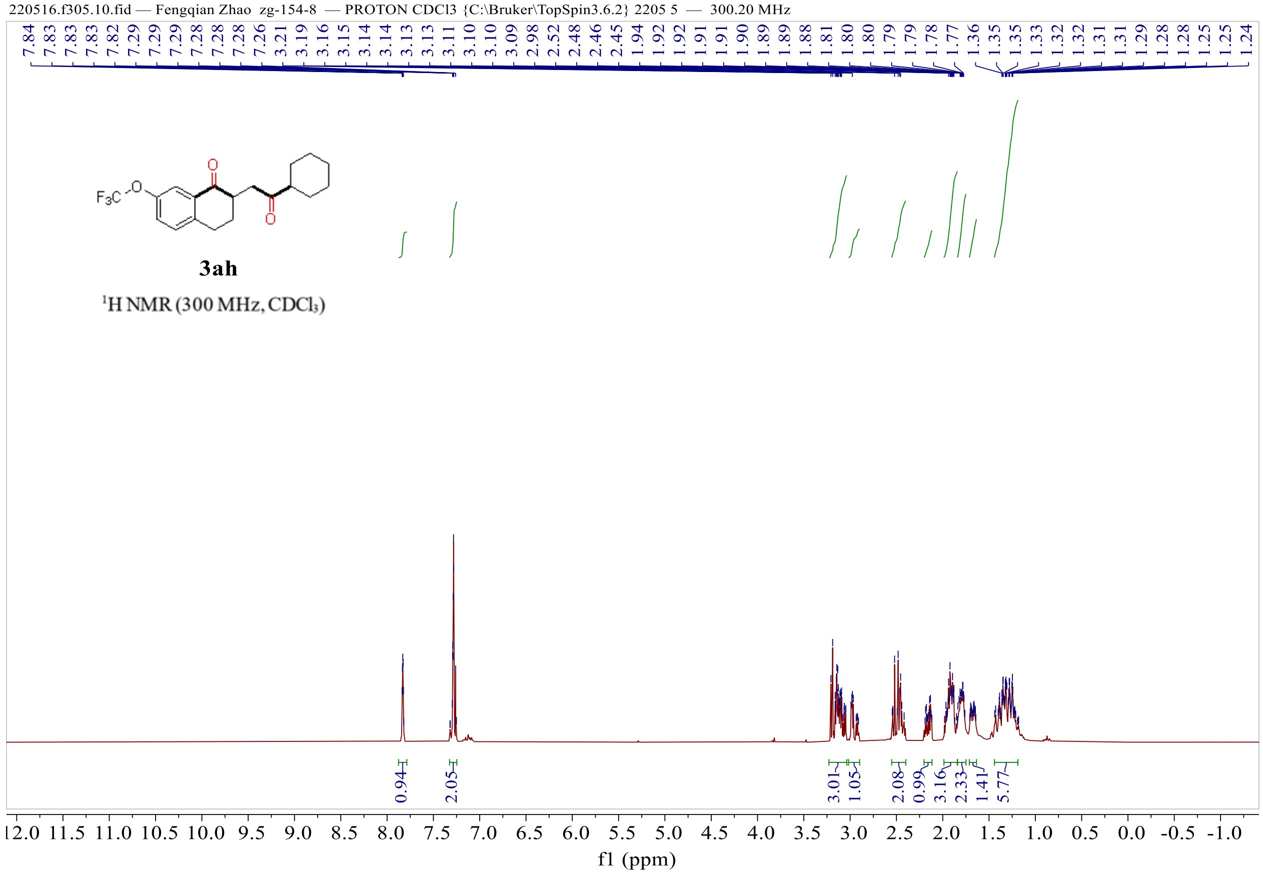
220516.f305.10.fid — Fengqian Zhao zg-154-8 — PROTON CDCl3 {C:\Bruker\TopSpin3.6.2} 2205 5 — 300.20 MHz
3ah
1H NMR (300 MHz, CDCl3)
0.94
2.05
3.01
1.05
2.08
0.99
3.16
2.33
1.41
5.77
f1 (ppm)

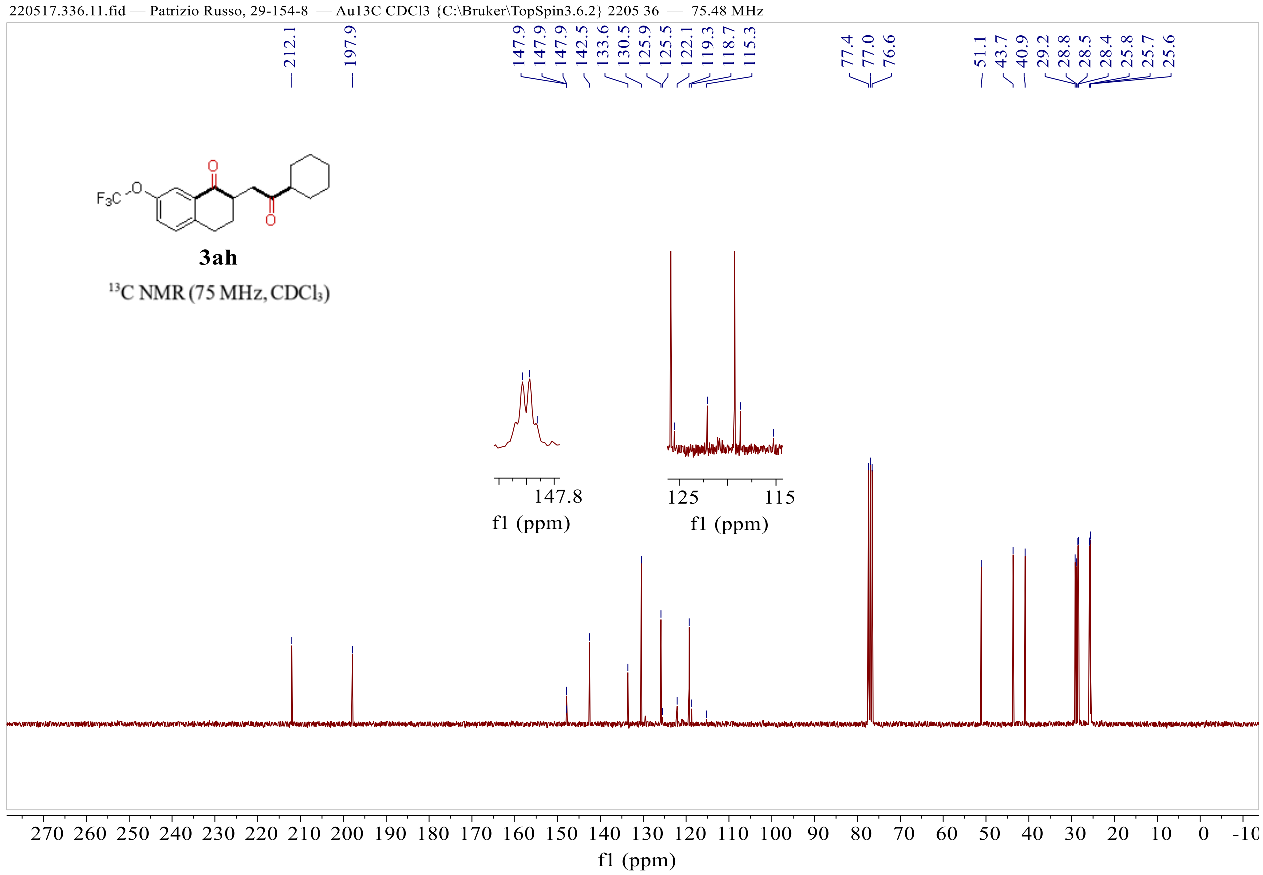
220517.336.11.fid — Patrizio Russo, 29-154-8 — Au13C CDCl3 {C:\Bruker\TopSpin3.6.2} 2205 36 — 75.48 MHz
212.1
197.9
147.9
147.9
147.9
142.5
133.6
130.5
125.9
125.5
122.1
119.3
118.7
115.3
77.4
77.0
76.6
51.1
43.7
40.9
29.2
28.8
28.5
28.4
25.8
25.7
25.6
3ah
13C NMR (75 MHz, CDCl3)
f1 (ppm)

220516.f305.12.fid — Fengqian Zhao zg-154-8 — F19 CDCl3 {C:\Bruker\TopSpin3.6.2} 2205 5 — 282.44 MHz

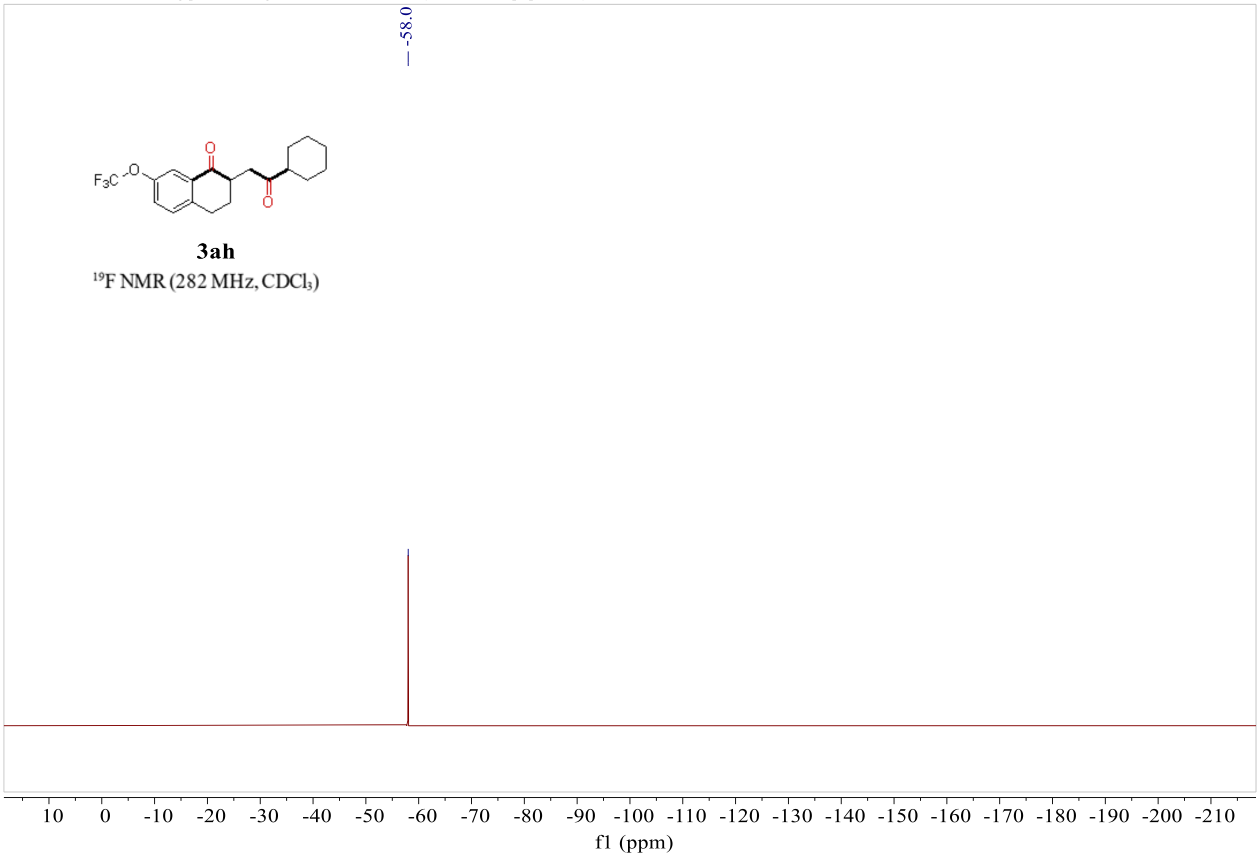

220530.342.10.fid — Fengqiang Zhao, 29-157-2 — Au1H CDCl3 {C:\Bruker\TopSpin3.6.2} 2205 42 — 300.13 MHz

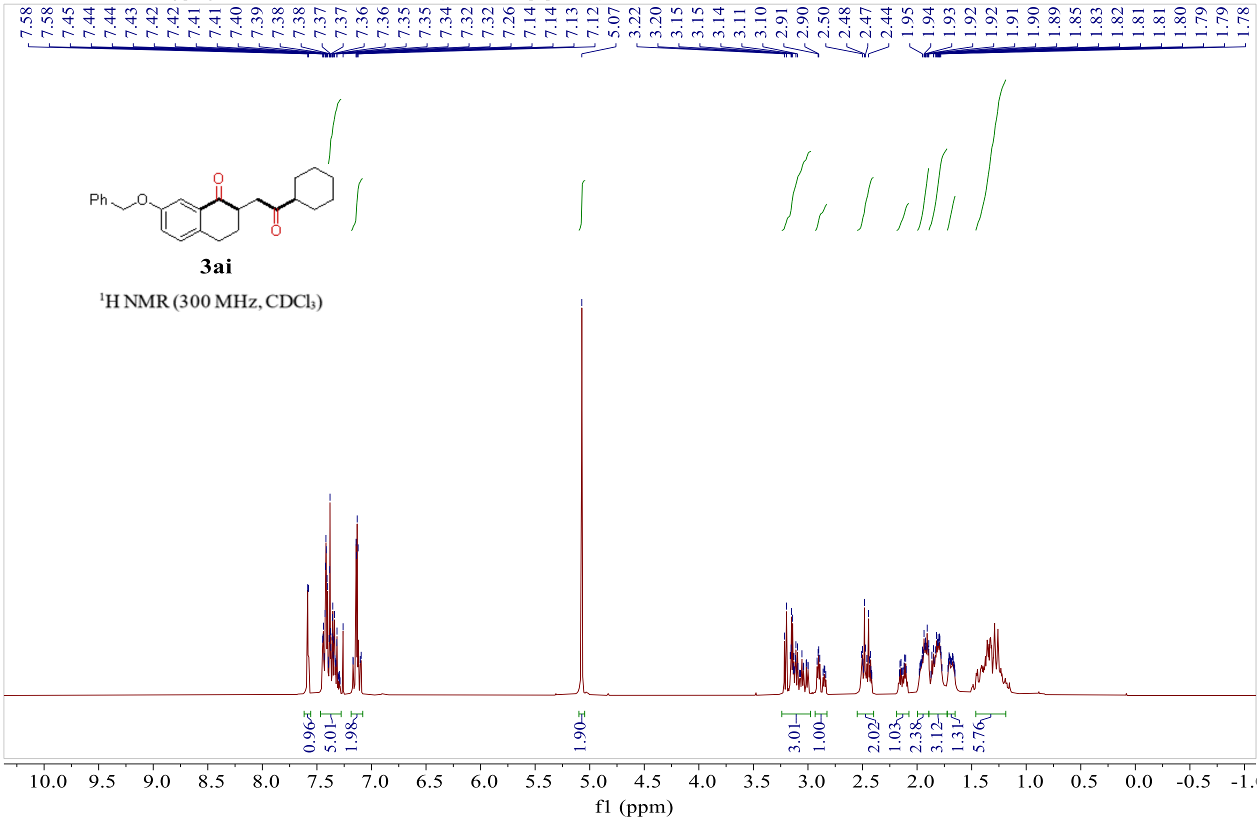

220530.342.11.fid — Fengqiang Zhao, 29-157-2 — Au13C CDCl3 {C:\Bruker\TopSpin3.6.2} 2205 42 — 75.48 MHz

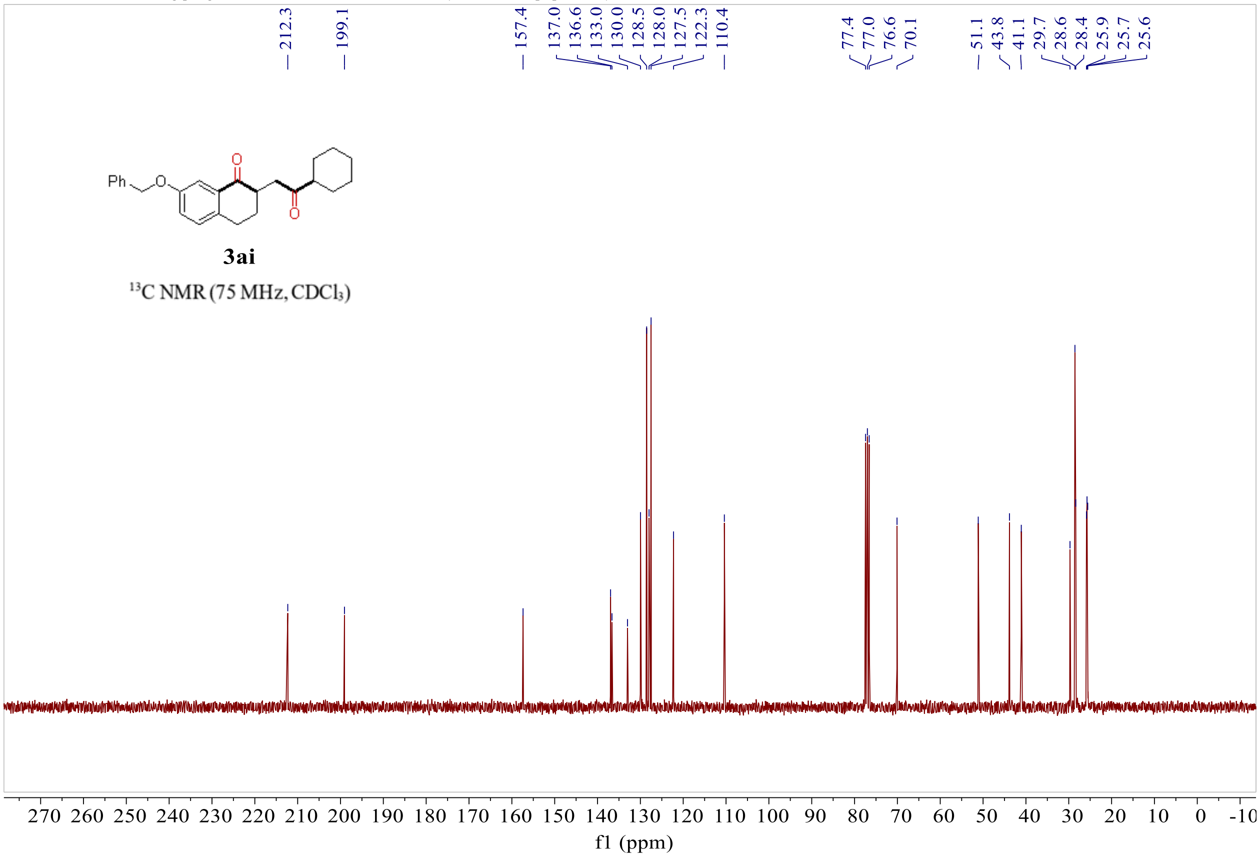

220525.415.10.fid — Fengqian Zhao zg-157-4 — Au1H CDCl3 {C:\Bruker\TopSpin3.5pl6} 2205 15 — 400.13 MHz

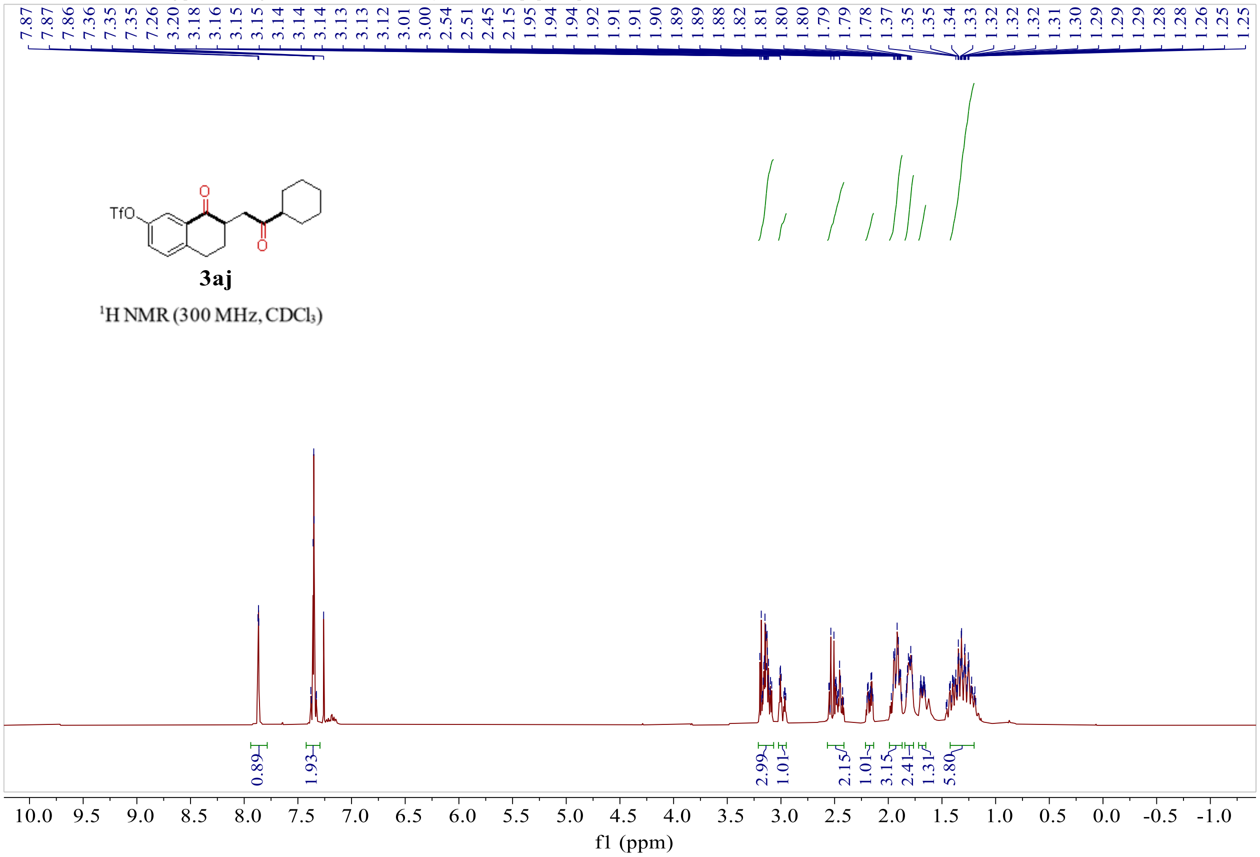

220530.343.10.fid — Fengqiang Zhao, 29-157-4 — Au13C CDCl3 {C:\Bruker\TopSpin3.6.2} 2205 43 — 75.48 MHz

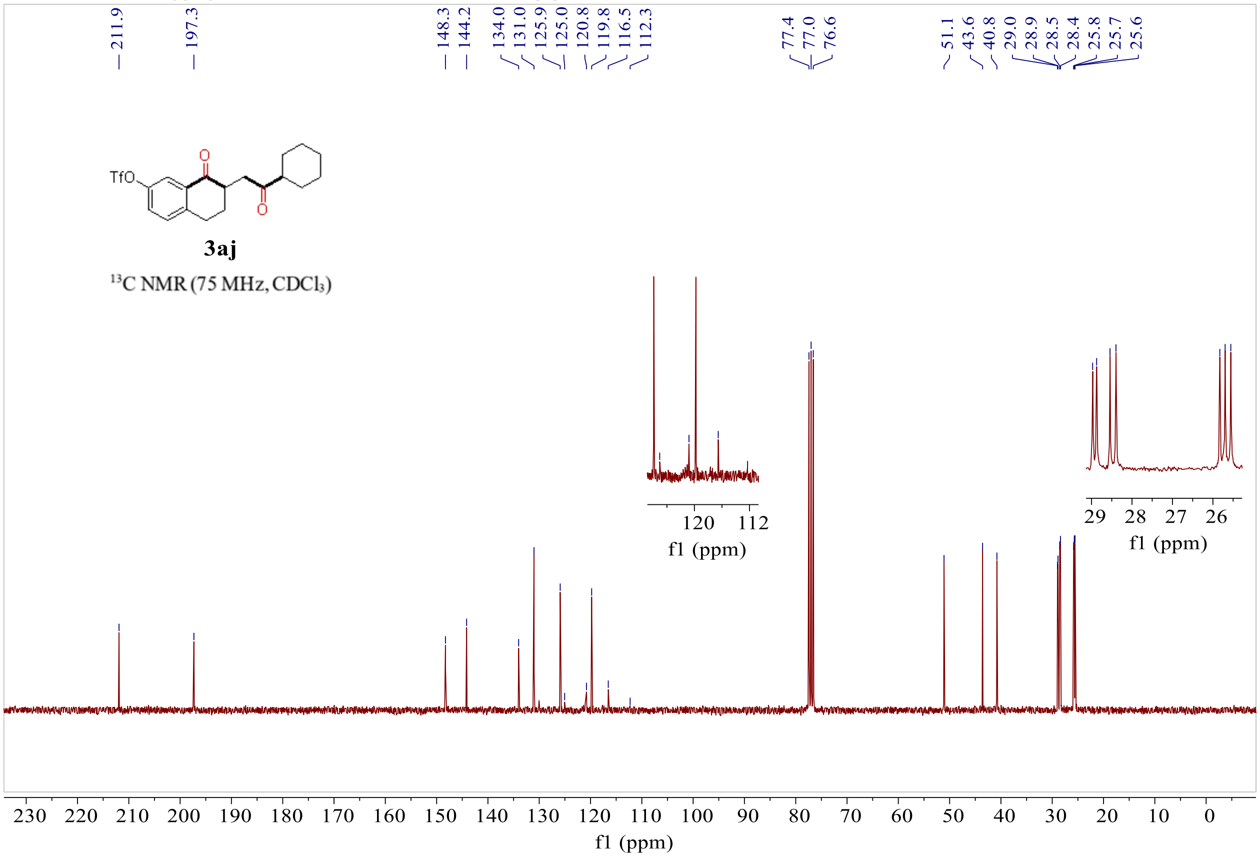

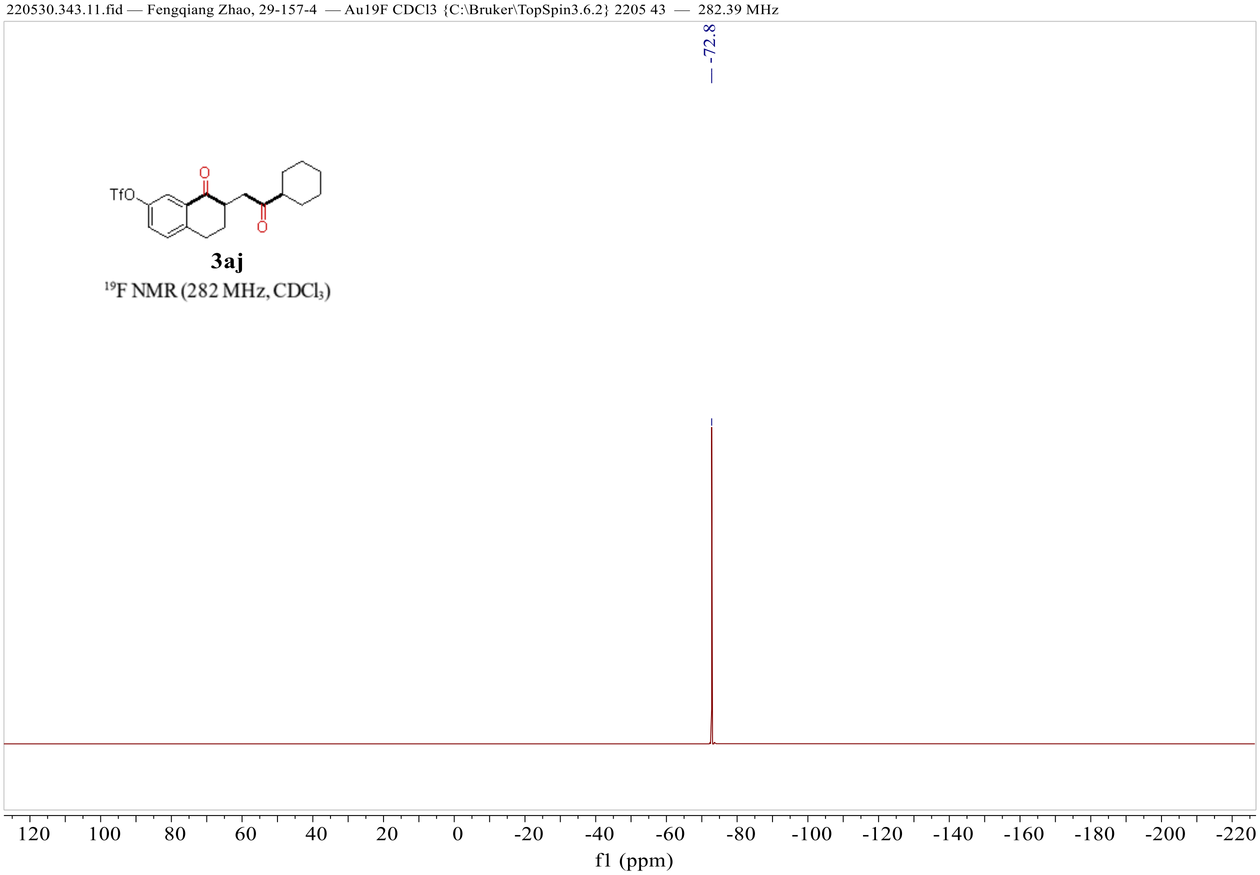
220530.343.11.fid — Fengqiang Zhao, 29-157-4 — Au19F CDCl3 {C:\Bruker\TopSpin3.6.2} 2205 43 — 282.39 MHz
-72.8
TfO
O
O
3aj
19F NMR (282 MHz, CDCl3)
120 100 80 60 40 20 0 -20 -40 -60 -80 -100 -120 -140 -160 -180 -200 -220
f1 (ppm)

220530.341.10.fid — Fengqiang Zhao, 29-156-9 — Au1H CDCl3 {C:\Bruker\TopSpin3.6.2} 2205 41 — 300.13 MHz

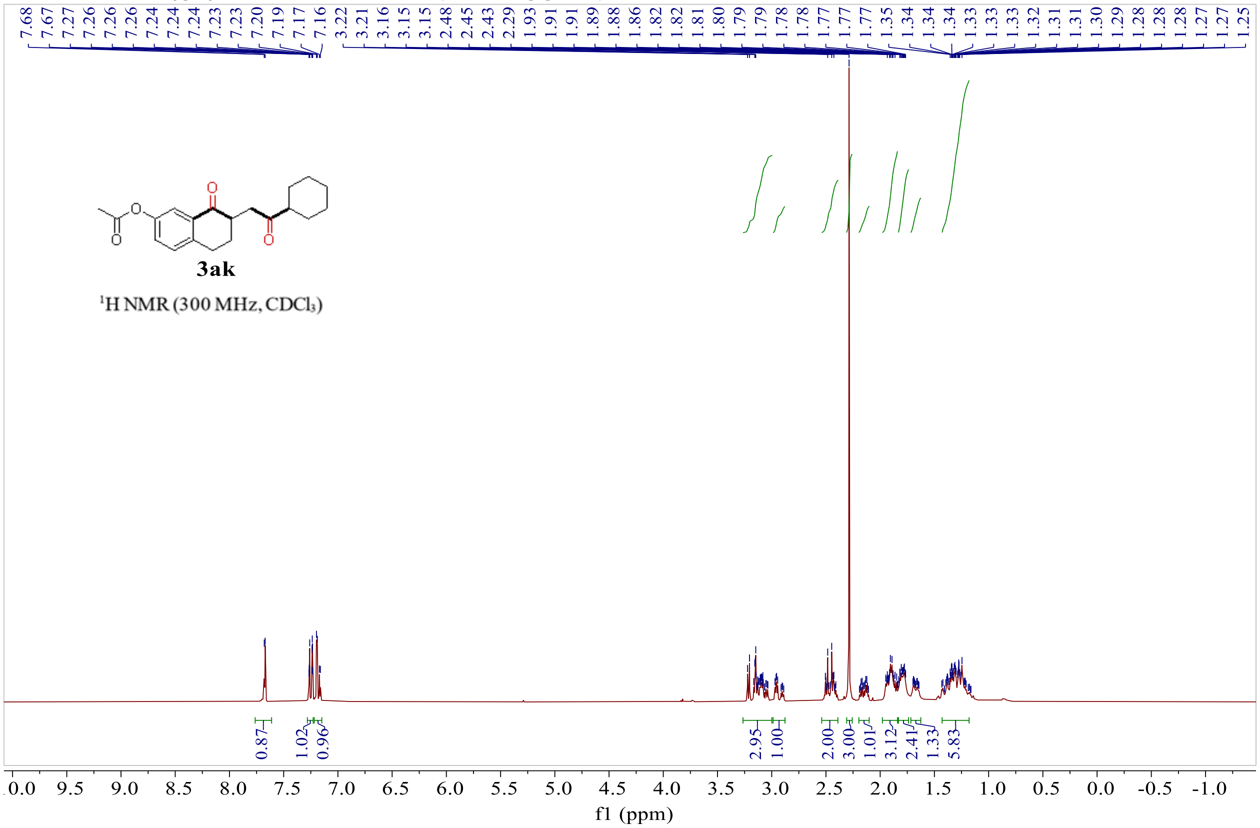

220530.341.11.fid — Fengqiang Zhao, 29-156-9 — Au13C CDCl3 {C:\Bruker\TopSpin3.6.2} 2205 41 — 75.48 MHz

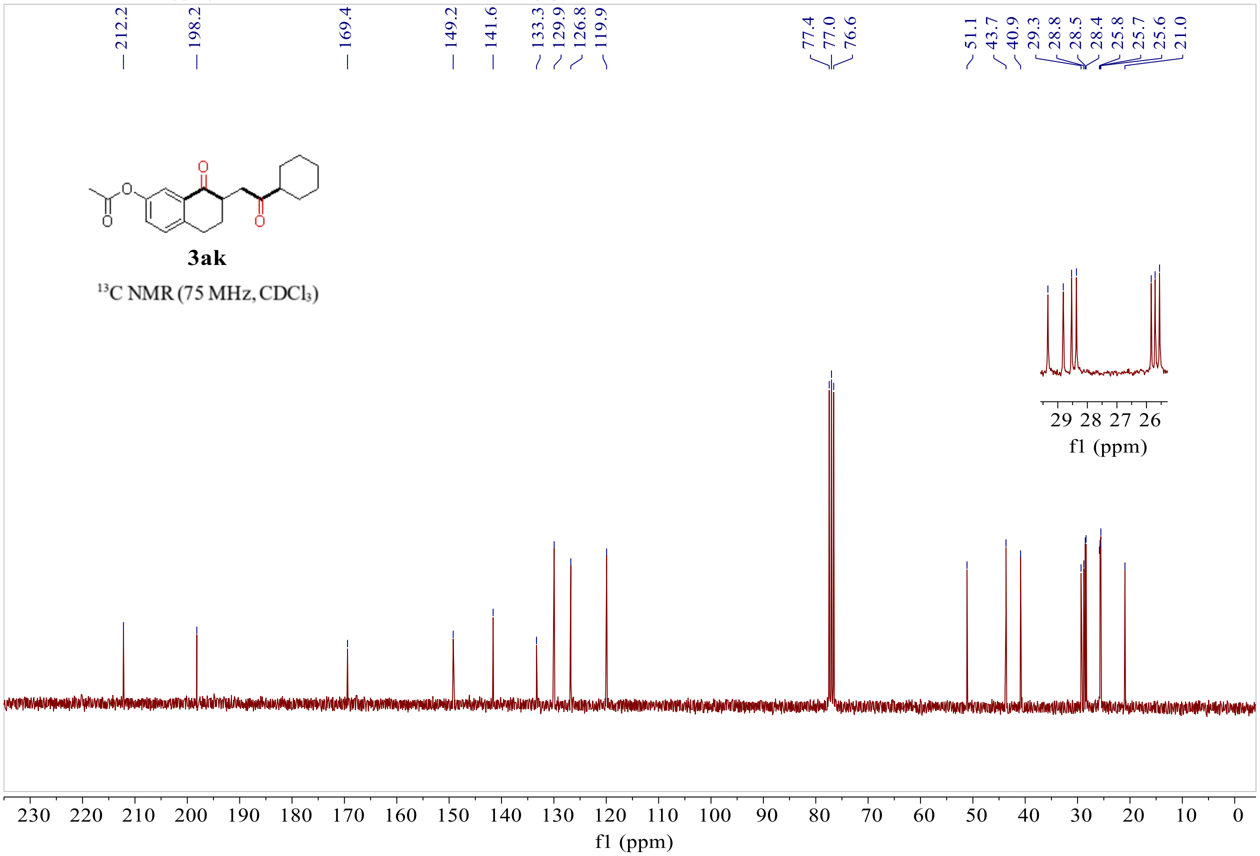

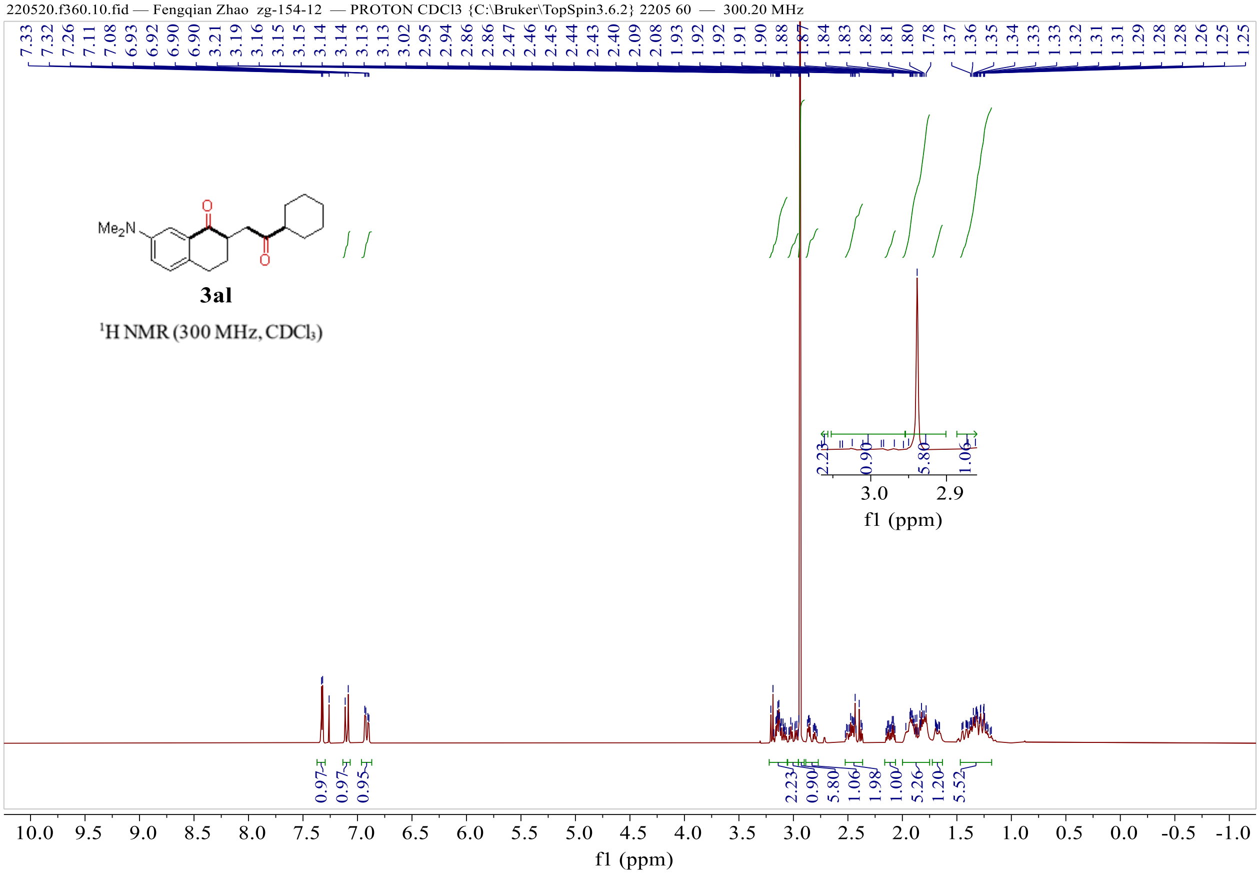
220520.f360.10.fid — Fengqian Zhao zg-154-12 — PROTON CDCl3 {C:\Bruker\TopSpin3.6.2} 2205 60 — 300.20 MHz
Me2N
3al
1H NMR (300 MHz, CDCl3)
f1 (ppm)

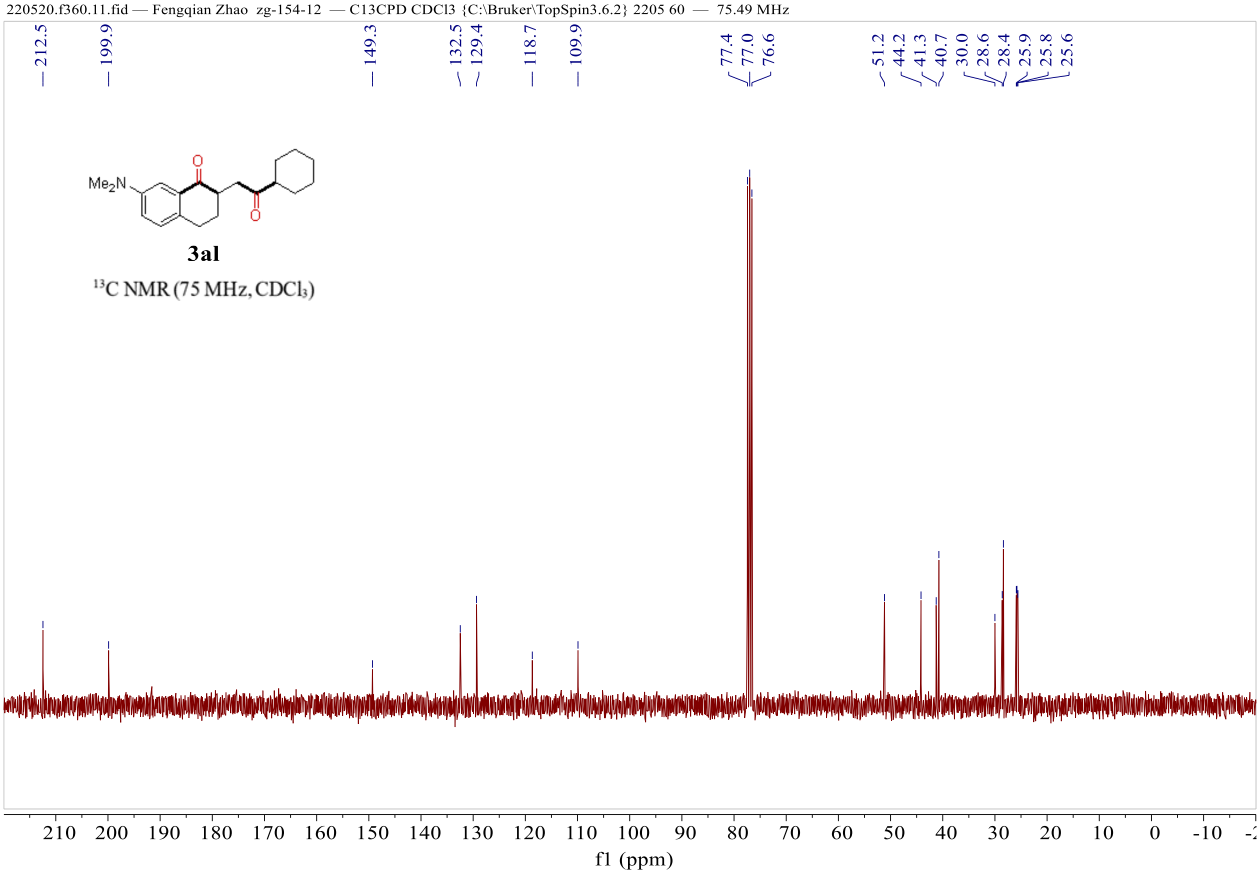
220520.f360.11.fid — Fengqian Zhao zg-154-12 — C13CPD CDCl3 {C:\Bruker\TopSpin3.6.2} 2205 60 — 75.49 MHz
212.5
199.9
149.3
132.5
129.4
118.7
109.9
77.4
77.0
76.6
51.2
44.2
41.3
40.7
30.0
28.6
28.4
25.9
25.8
25.6
Me2N
3al
13C NMR (75 MHz, CDCl3)
f1 (ppm)

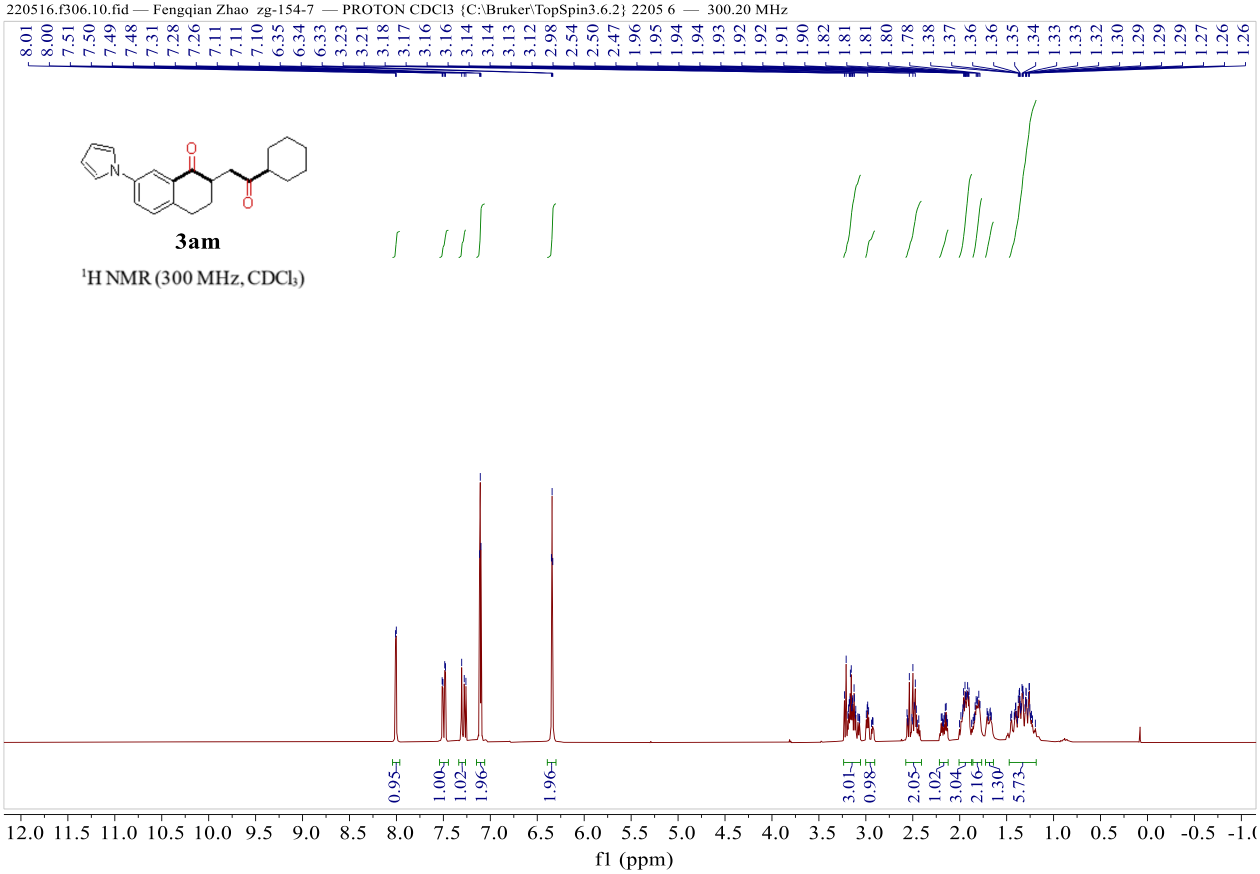
220516.f306.10.fid — Fengqian Zhao zg-154-7 — PROTON CDCl3 {C:\Bruker\TopSpin3.6.2} 2205 6 — 300.20 MHz
8.01 8.00 7.51 7.50 7.49 7.48 7.31 7.28 7.26 7.11 7.11 7.10 6.35 6.34 6.33 3.23 3.21 3.18 3.17 3.16 3.16 3.14 3.14 3.13 3.12 2.98 2.54 2.50 2.47 1.96 1.95 1.94 1.94 1.93 1.92 1.92 1.91 1.90 1.82 1.81 1.81 1.80 1.78 1.38 1.37 1.36 1.36 1.35 1.34 1.33 1.33 1.32 1.30 1.29 1.29 1.27 1.26 1.26
3am
1H NMR (300 MHz, CDCl3)
0.95 1.00 1.02 1.96 1.96 3.01 0.98 2.05 1.02 3.04 2.16 1.30 5.73
f1 (ppm)

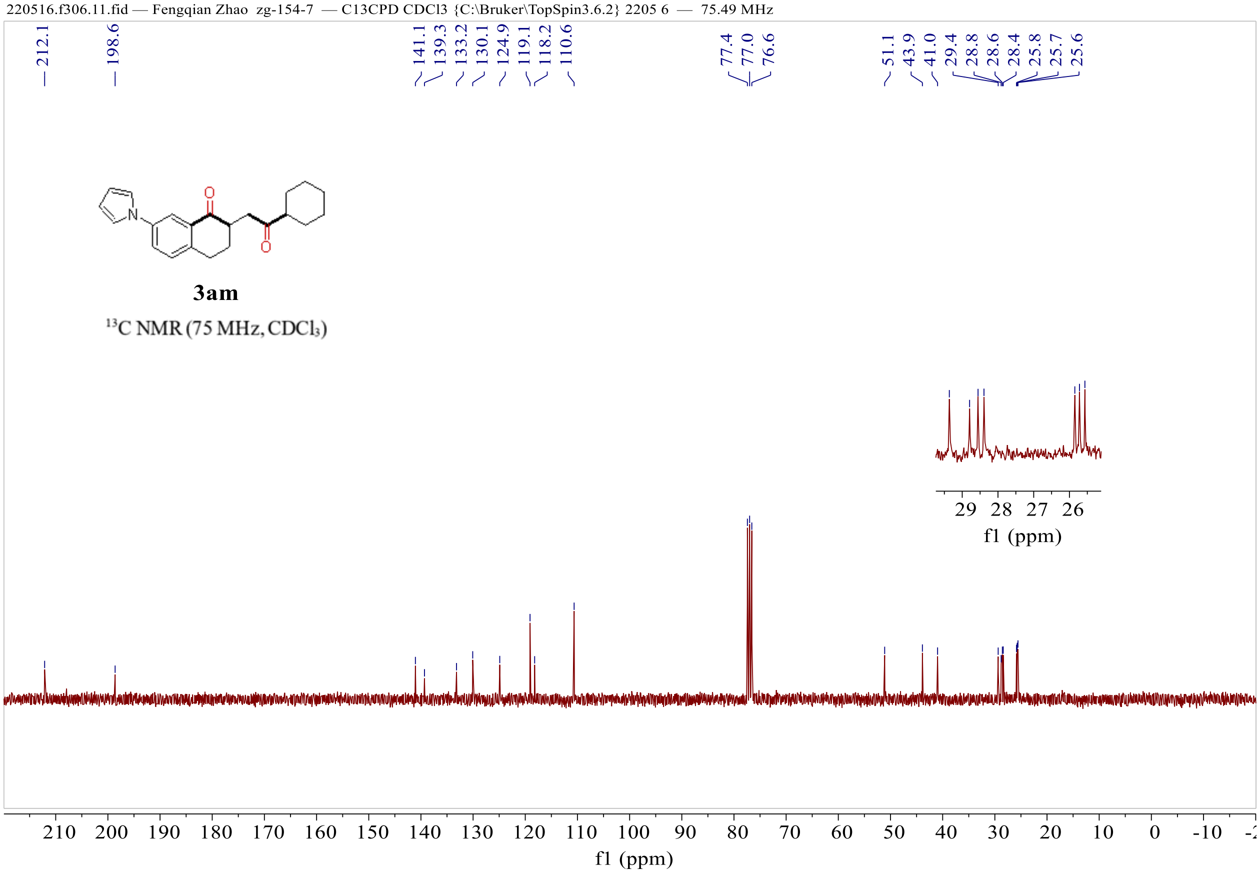
220516.f306.11.fid — Fengqian Zhao zg-154-7 — C13CPD CDCl3 {C:\Bruker\TopSpin3.6.2} 2205 6 — 75.49 MHz
212.1 198.6 141.1 139.3 133.2 130.1 124.9 119.1 118.2 110.6 77.4 77.0 76.6 51.1 43.9 41.0 29.4 28.8 28.6 28.4 25.8 25.7 25.6
3am
13C NMR (75 MHz, CDCl3)
f1 (ppm)

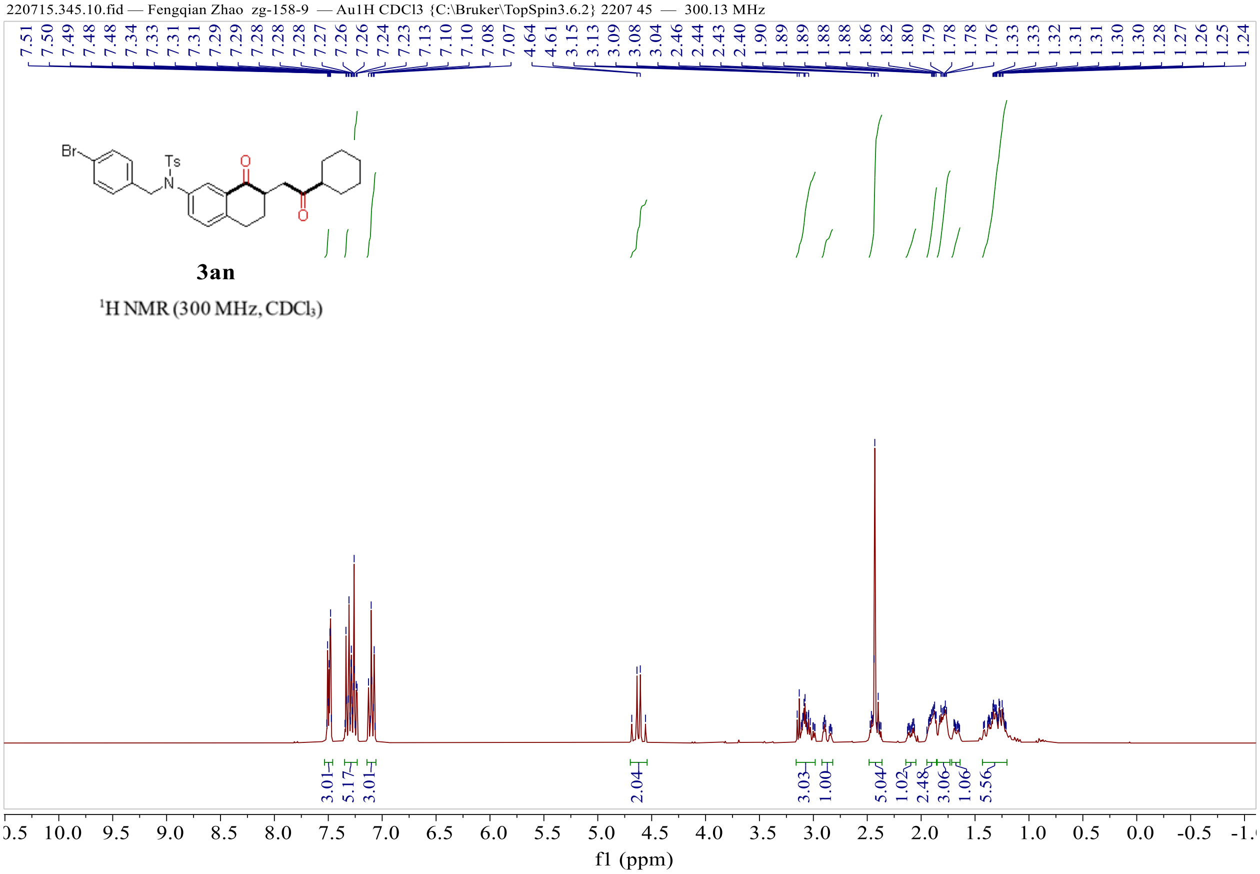
220715.345.10.fid — Fengqian Zhao zg-158-9 — Au1H CDCl3 {C:\Bruker\TopSpin3.6.2} 2207 45 — 300.13 MHz
7.51 7.50 7.49 7.48 7.48 7.34 7.33 7.31 7.31 7.29 7.29 7.28 7.28 7.28 7.27 7.26 7.26 7.24 7.23 7.13 7.10 7.08 7.07 4.64 4.61 3.15 3.13 3.09 3.08 3.04 2.46 2.44 2.43 2.40 1.90 1.89 1.89 1.88 1.88 1.86 1.82 1.80 1.79 1.78 1.78 1.76 1.33 1.33 1.32 1.31 1.31 1.30 1.30 1.28 1.27 1.26 1.25 1.24
Br
Ts
N
O
O
3an
1H NMR (300 MHz, CDCl3)
3.01 5.17 3.01 2.04 3.03 1.00 5.04 1.02 2.48 3.06 1.06 5.56
0.5 10.0 9.5 9.0 8.5 8.0 7.5 7.0 6.5 6.0 5.5 5.0 4.5 4.0 3.5 3.0 2.5 2.0 1.5 1.0 0.5 0.0 -0.5 -1.
f1 (ppm)

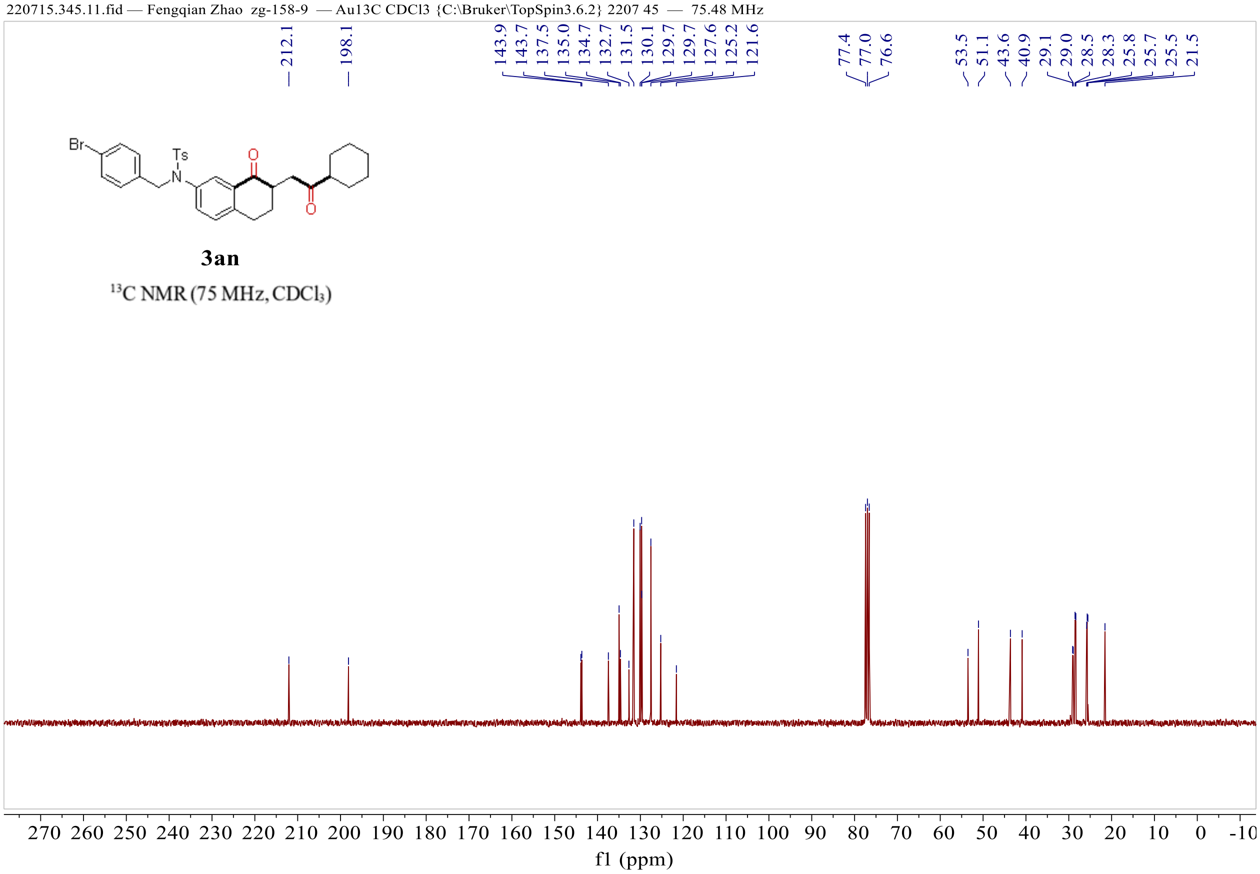
220715.345.11.fid — Fengqian Zhao zg-158-9 — Au13C CDCl3 {C:\Bruker\TopSpin3.6.2} 2207 45 — 75.48 MHz
212.1 198.1 143.9 143.7 137.5 135.0 134.7 132.7 131.5 130.1 129.7 129.7 127.6 125.2 121.6 77.4 77.0 76.6 53.5 51.1 43.6 40.9 29.1 29.0 28.5 28.3 25.8 25.7 25.5 21.5
Br
Ts
N
O
O
3an
13C NMR (75 MHz, CDCl3)
270 260 250 240 230 220 210 200 190 180 170 160 150 140 130 120 110 100 90 80 70 60 50 40 30 20 10 0 -10
f1 (ppm)

220524.306.10.fid — Fengqian Zhao zg-156-7 — Au1H CDCl3 {C:\Bruker\TopSpin3.6.2} 2205 6 — 300.13 MHz

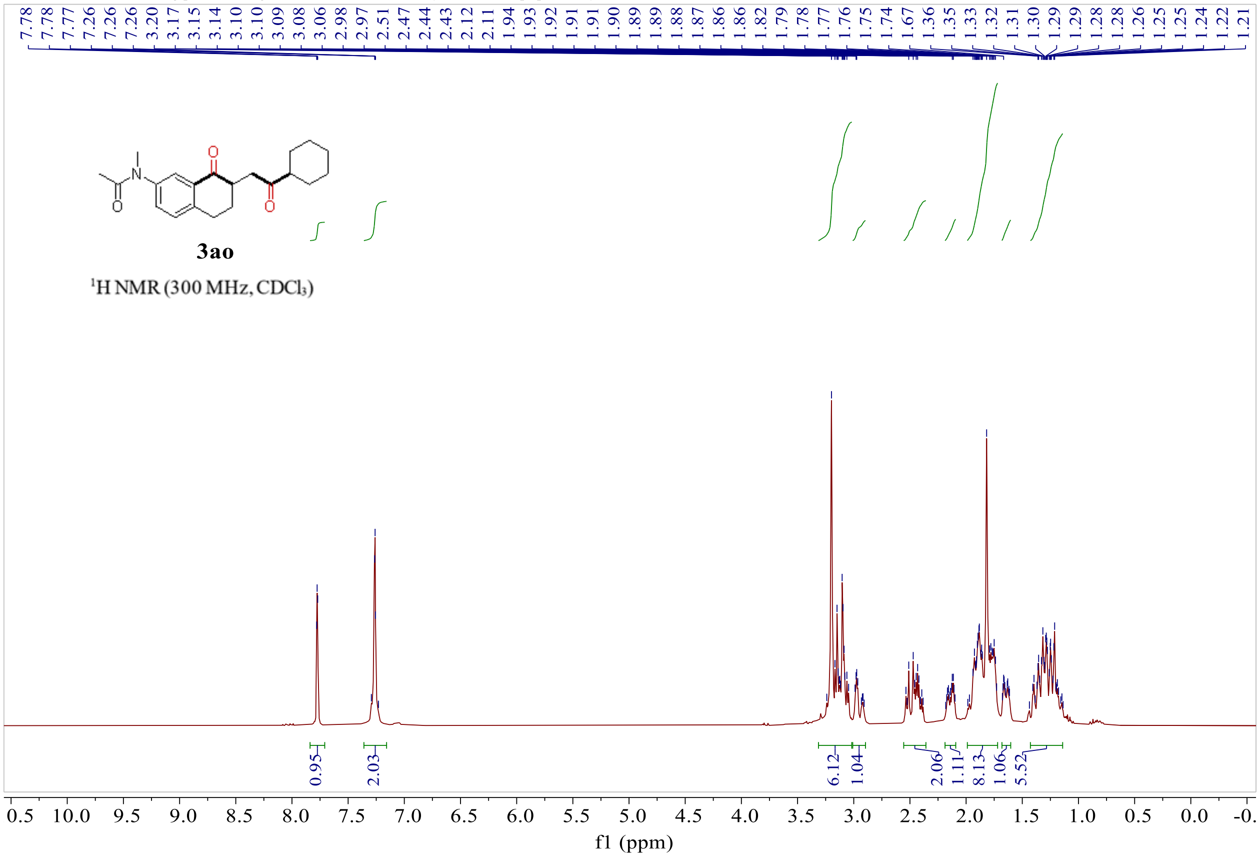

220524.306.11.fid — Fengqian Zhao zg-156-7 — Au13C CDCl3 {C:\Bruker\TopSpin3.6.2} 2205 6 — 75.48 MHz

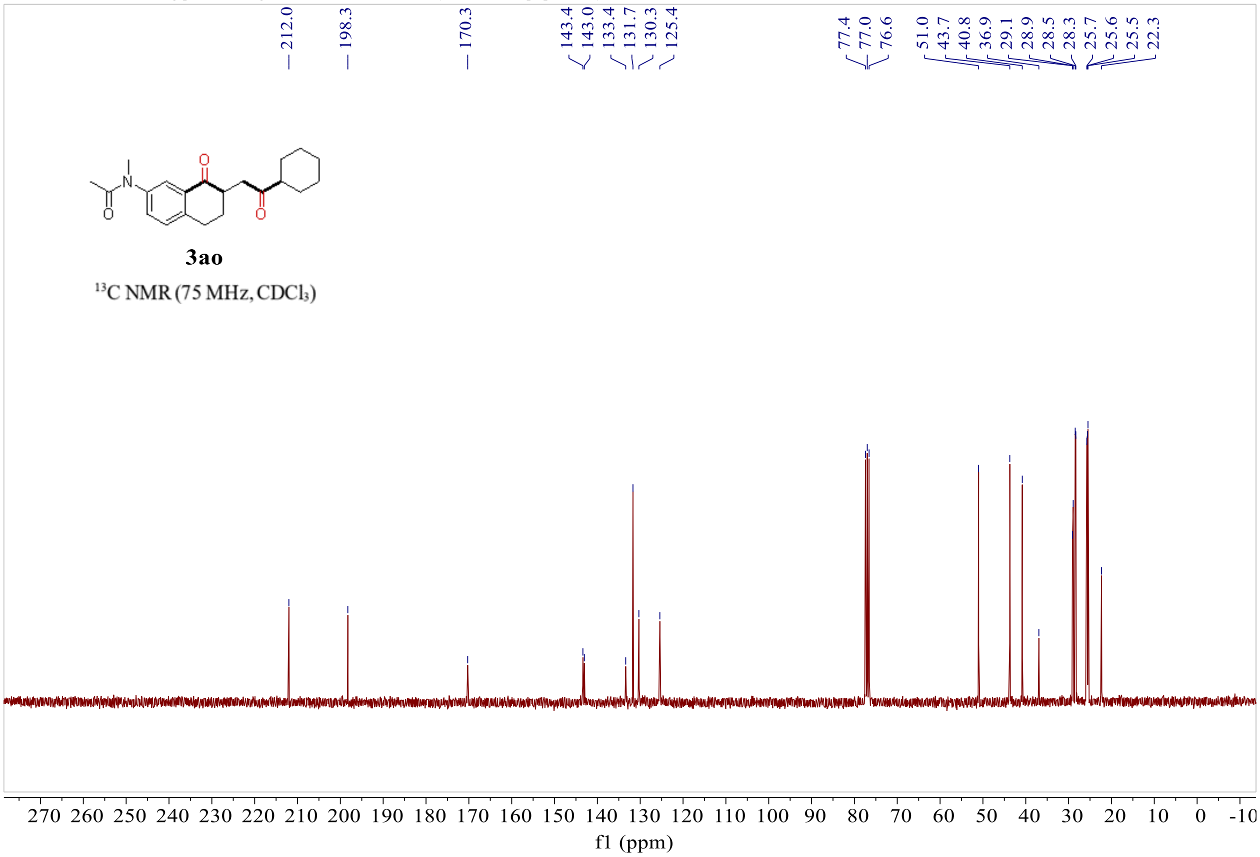

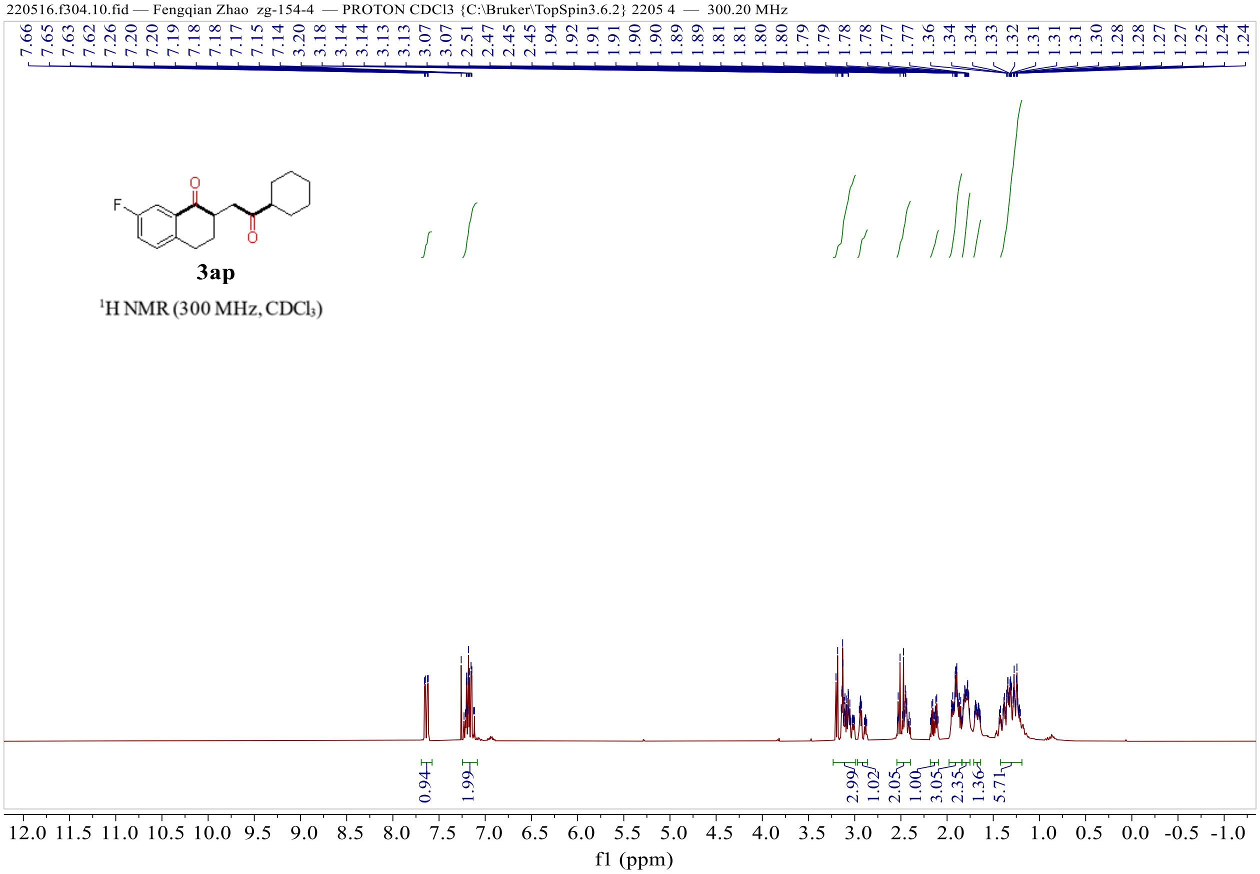
220516.f304.10.fid — Fengqian Zhao zg-154-4 — PROTON CDCl3 {C:\Bruker\TopSpin3.6.2} 2205 4 — 300.20 MHz
F
O
O
3ap
^{1}H NMR (300 MHz, CDCl$_3$)
f1 (ppm)

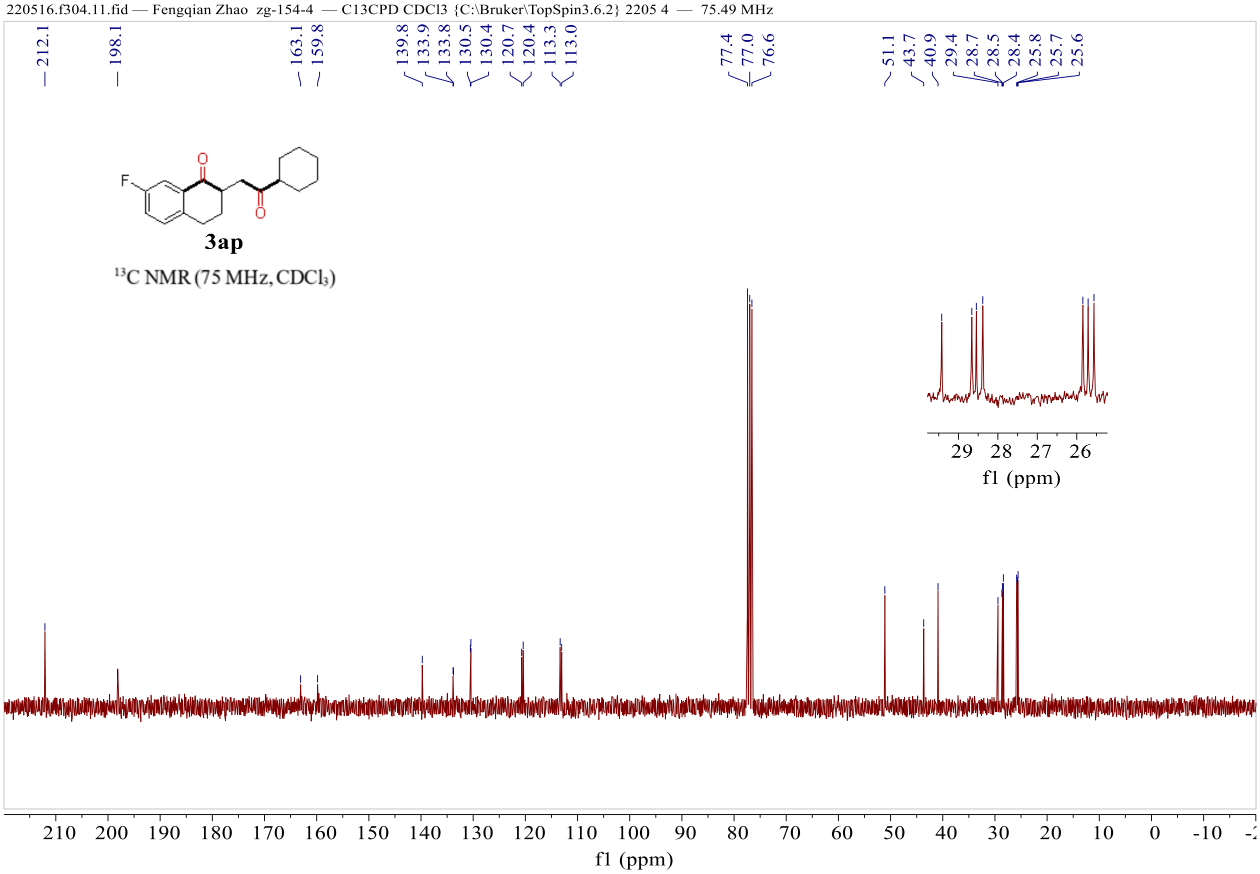
220516.f304.11.fid — Fengqian Zhao zg-154-4 — C13CPD CDCl3 {C:\Bruker\TopSpin3.6.2} 2205 4 — 75.49 MHz
212.1
198.1
163.1
159.8
139.8
133.9
133.8
130.5
130.4
120.7
120.4
113.3
113.0
77.4
77.0
76.6
51.1
43.7
40.9
29.4
28.7
28.5
28.4
25.8
25.7
25.6
F
O
O
3ap
^{13}C NMR (75 MHz, CDCl$_3$)
f1 (ppm)

220516.f304.12.fid — Fengqian Zhao zg-154-4 — F19 CDCl3 {C:\Bruker\TopSpin3.6.2} 2205 4 — 282.44 MHz

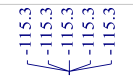

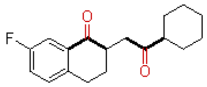

3ap

^{19}F NMR (282 MHz, CDCl$_3$)

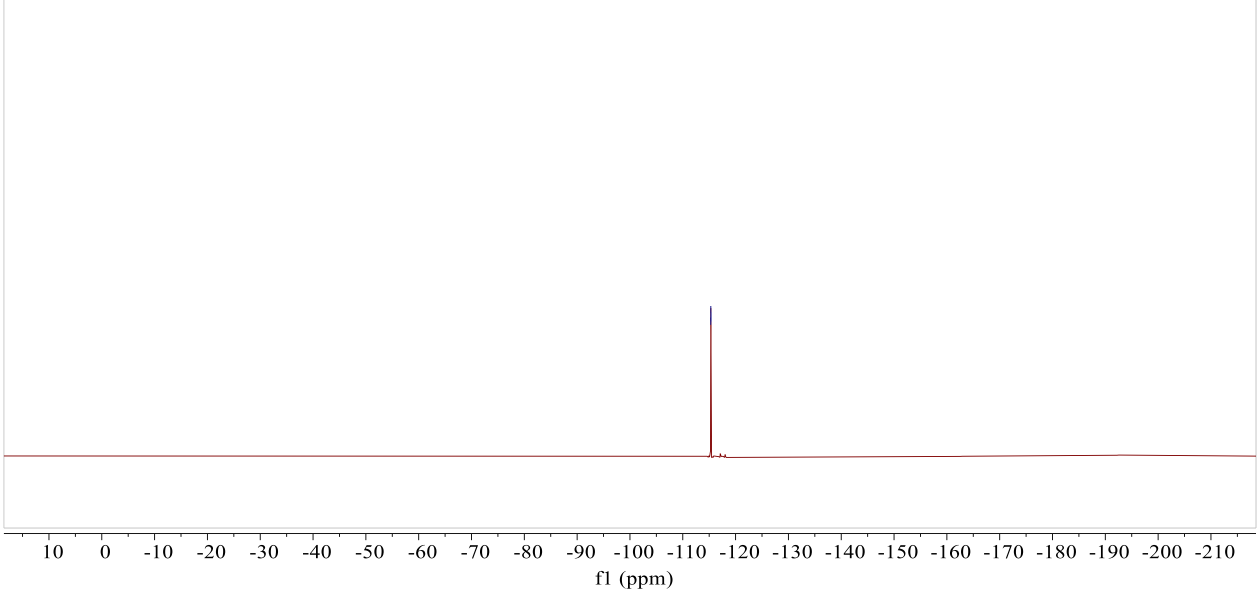

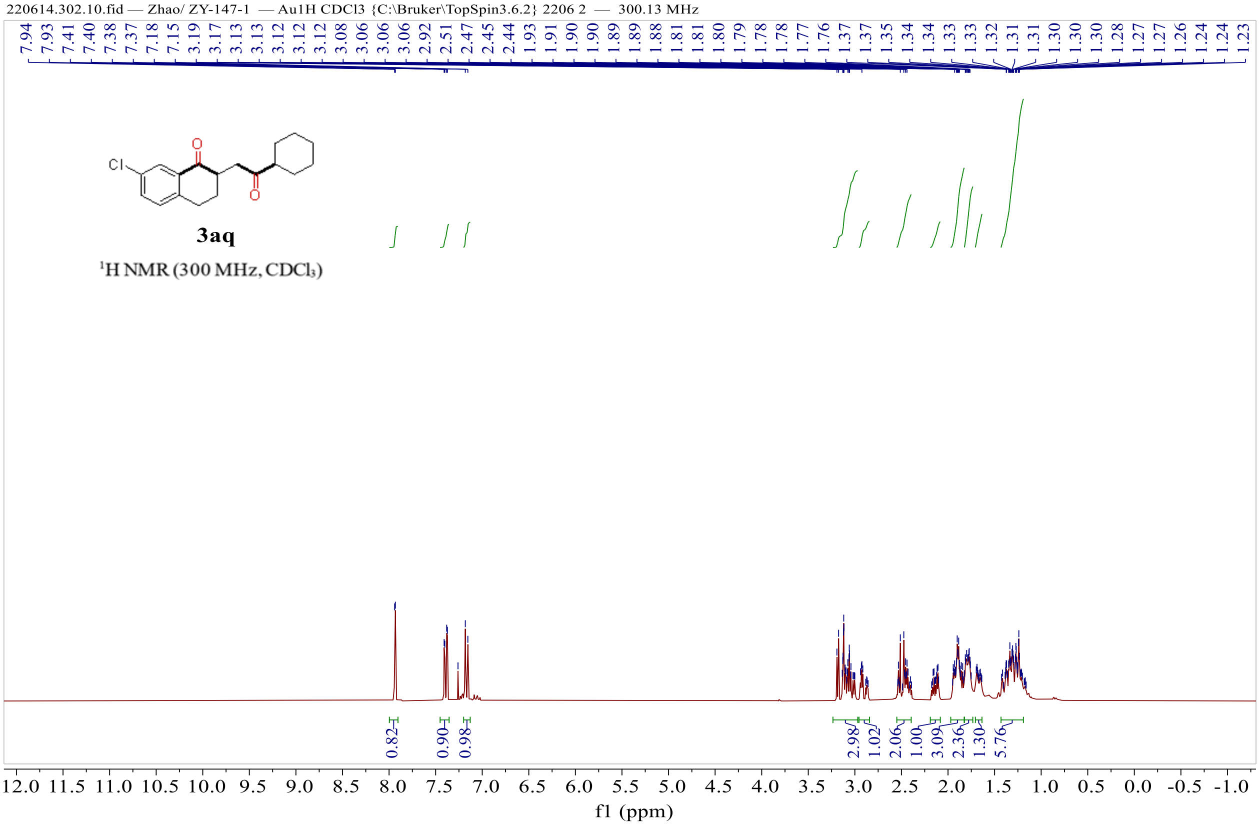
220614.302.10.fid — Zhao/ ZY-147-1 — Au1H CDCl3 {C:\Bruker\TopSpin3.6.2} 2206 2 — 300.13 MHz
7.94 7.93 7.41 7.40 7.38 7.37 7.18 7.15 3.19 3.17 3.13 3.13 3.12 3.12 3.12 3.08 3.06 3.06 3.06 2.92 2.51 2.47 2.45 2.44 1.93 1.91 1.90 1.89 1.89 1.88 1.81 1.81 1.80 1.79 1.78 1.78 1.77 1.76 1.37 1.37 1.35 1.34 1.34 1.33 1.33 1.32 1.31 1.31 1.30 1.30 1.30 1.28 1.27 1.27 1.26 1.24 1.24 1.23
Cl
O
O
3aq
1H NMR (300 MHz, CDCl3)
0.82 0.90 0.98 2.98 1.02 2.06 1.00 3.09 2.36 1.30 5.76
12.0 11.5 11.0 10.5 10.0 9.5 9.0 8.5 8.0 7.5 7.0 6.5 6.0 5.5 5.0 4.5 4.0 3.5 3.0 2.5 2.0 1.5 1.0 0.5 0.0 -0.5 -1.0
f1 (ppm)

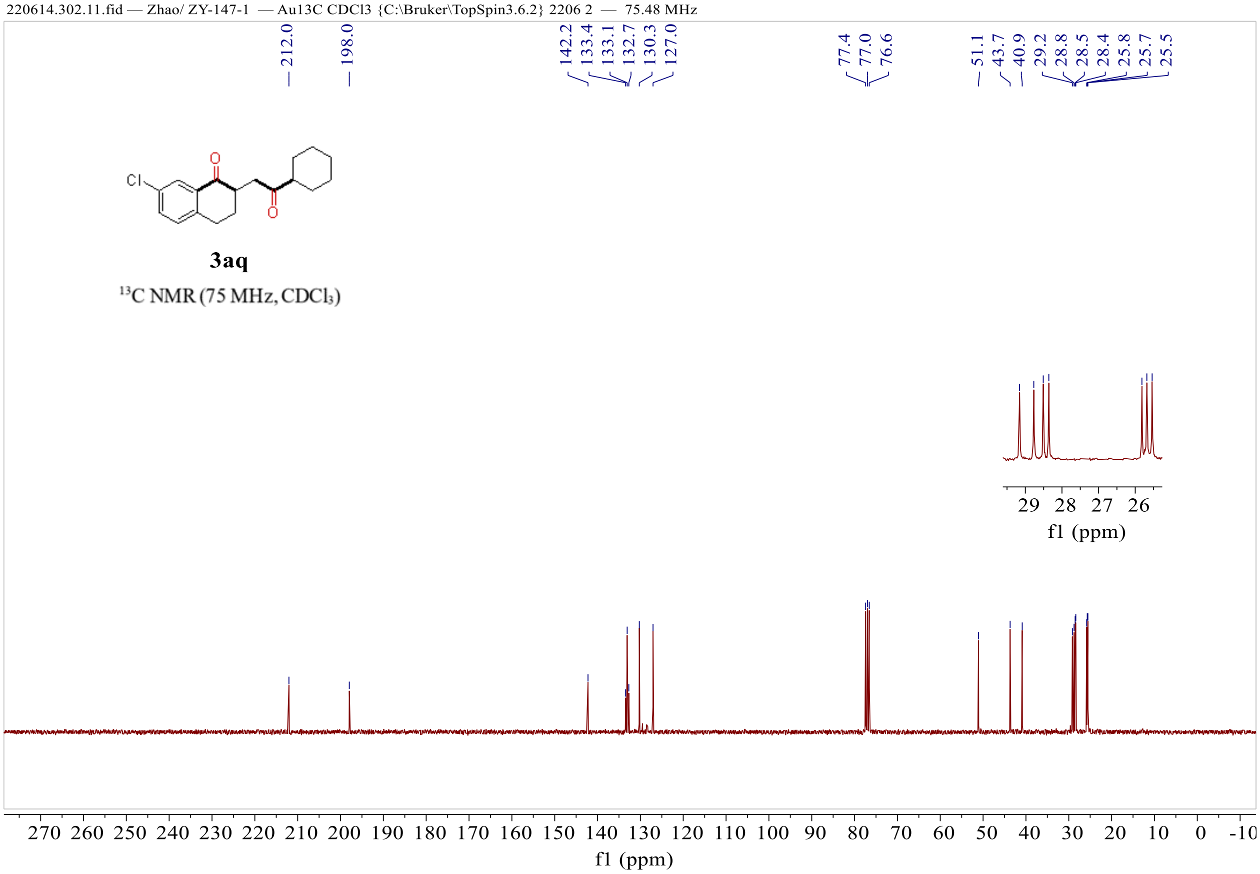
220614.302.11.fid — Zhao/ ZY-147-1 — Au13C CDCl3 {C:\Bruker\TopSpin3.6.2} 2206 2 — 75.48 MHz
212.0 198.0 142.2 133.4 133.1 132.7 130.3 127.0 77.4 77.0 76.6 51.1 43.7 40.9 29.2 28.8 28.5 28.4 25.8 25.7 25.5
Cl
O
O
3aq
13C NMR (75 MHz, CDCl3)
29 28 27 26
f1 (ppm)
270 260 250 240 230 220 210 200 190 180 170 160 150 140 130 120 110 100 90 80 70 60 50 40 30 20 10 0 -10
f1 (ppm)

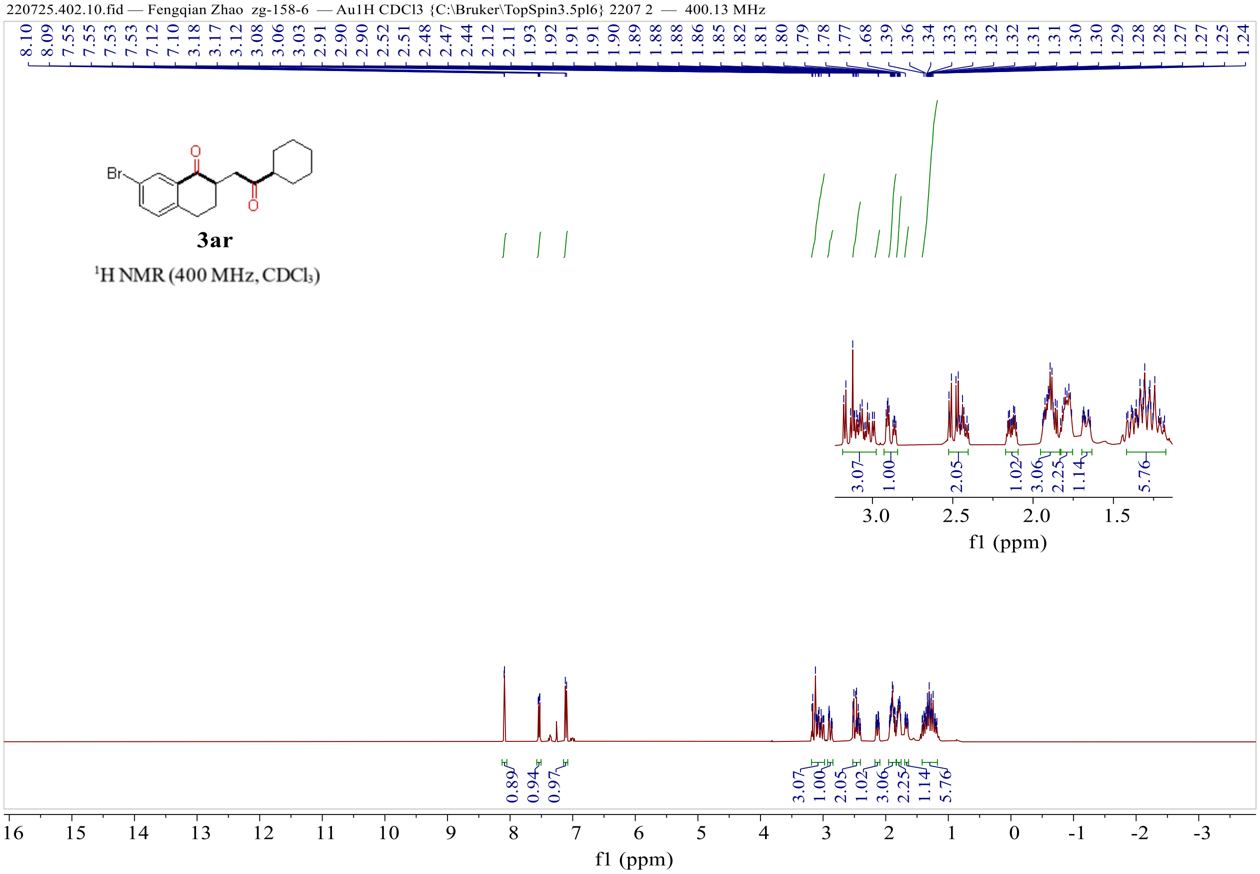
220725.402.10.fid — Fengqian Zhao zg-158-6 — Au1H CDCl3 {C:\Bruker\TopSpin3.5pl6} 2207 2 — 400.13 MHz
8.10 8.09 7.55 7.55 7.53 7.53 7.12 7.10 3.18 3.17 3.12 3.08 3.06 3.03 2.91 2.90 2.90 2.52 2.51 2.48 2.47 2.44 2.12 2.11 1.93 1.92 1.91 1.91 1.90 1.89 1.88 1.88 1.86 1.85 1.82 1.81 1.80 1.79 1.78 1.77 1.68 1.39 1.36 1.34 1.33 1.33 1.32 1.32 1.31 1.31 1.30 1.30 1.29 1.28 1.28 1.27 1.27 1.25 1.24
Br
O
O
3ar
1H NMR (400 MHz, CDCl3)
3.07 1.00 2.05 1.02 3.06 2.25 1.14 5.76
0.89 0.94 0.97
f1 (ppm)

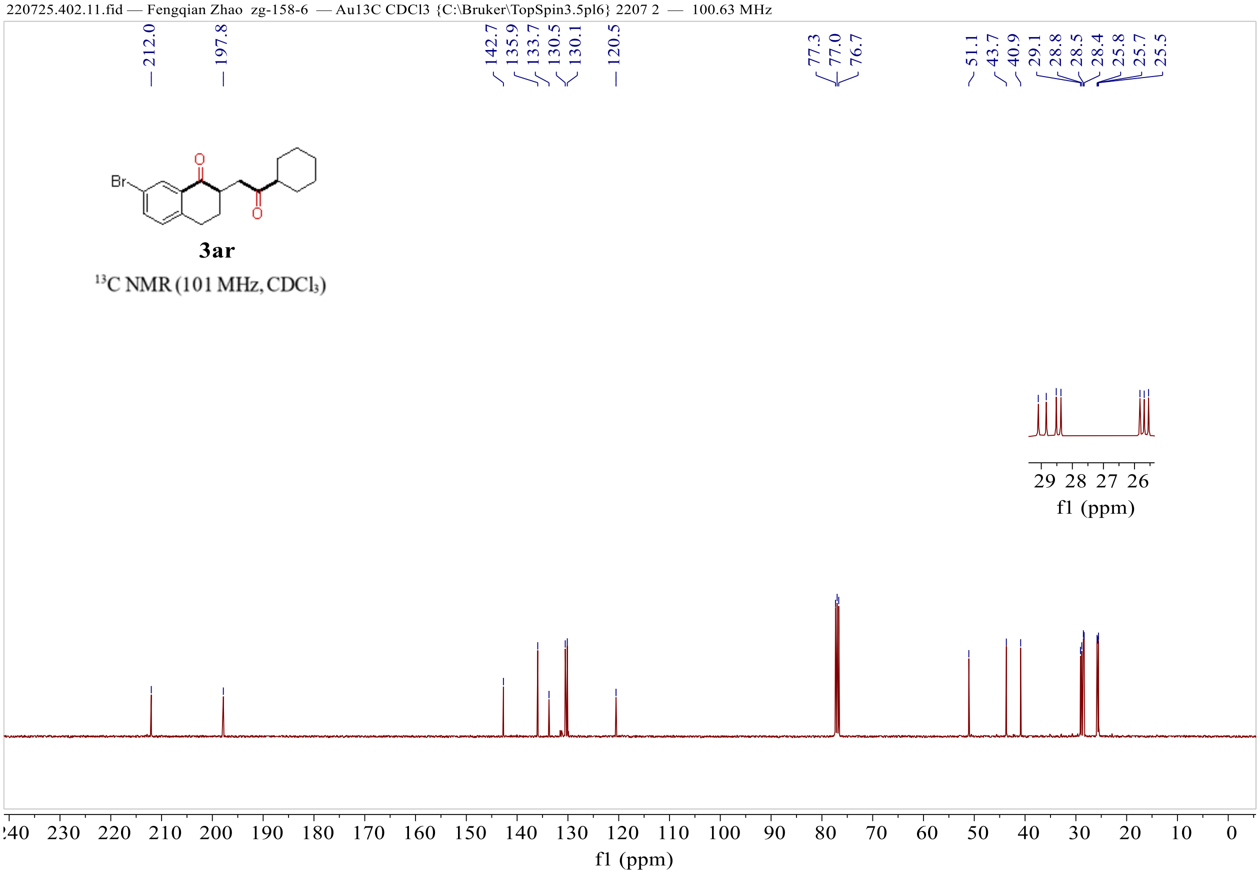
220725.402.11.fid — Fengqian Zhao zg-158-6 — Au13C CDCl3 {C:\Bruker\TopSpin3.5pl6} 2207 2 — 100.63 MHz
212.0 197.8 142.7 135.9 133.7 130.5 130.1 120.5 77.3 77.0 76.7 51.1 43.7 40.9 29.1 28.8 28.5 28.4 25.8 25.7 25.5
Br
O
O
3ar
13C NMR (101 MHz, CDCl3)
f1 (ppm)

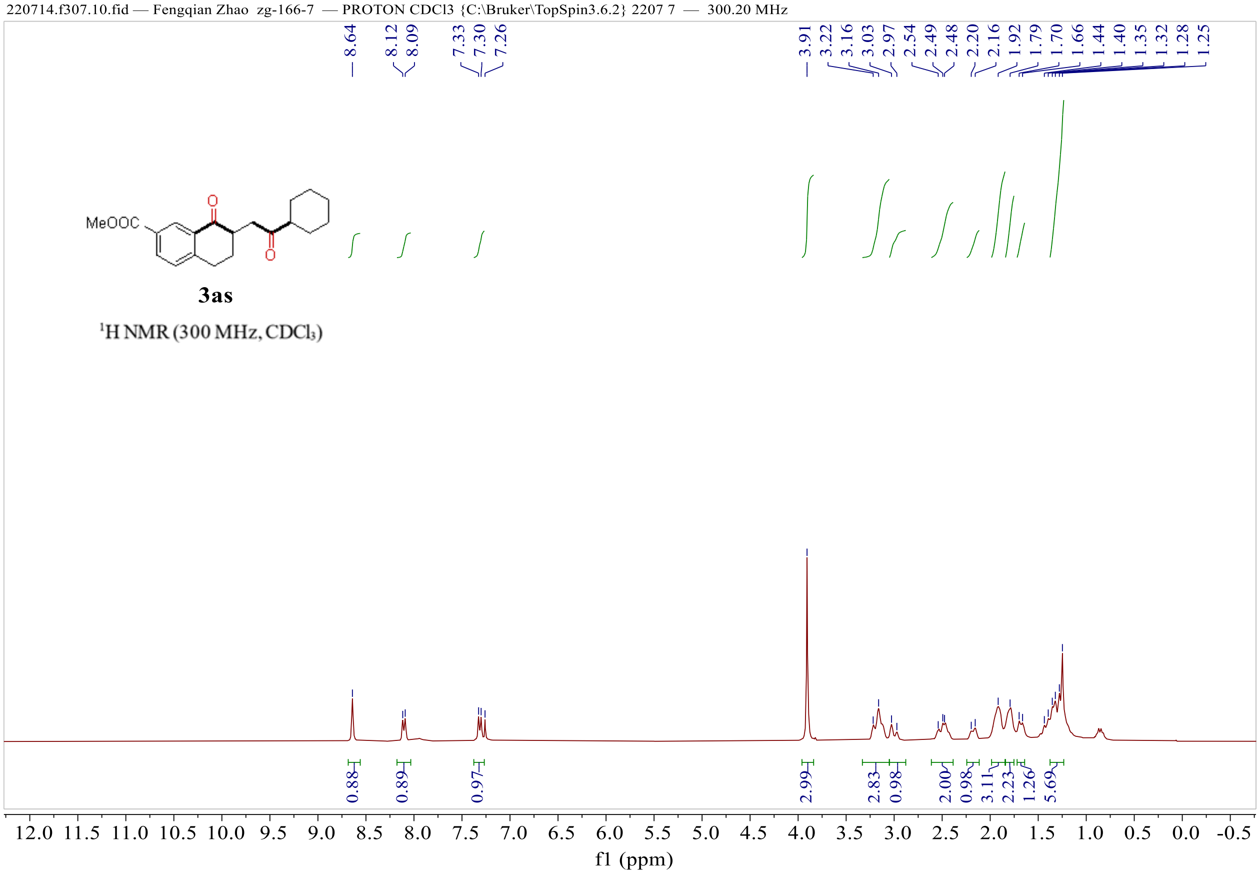

220714.f307.10.fid — Fengqian Zhao zg-166-7 — PROTON CDCl3 {C:\Bruker\TopSpin3.6.2} 2207 7 — 300.20 MHz
MeOOC
O
O
3as
¹H NMR (300 MHz, CDCl₃)
f1 (ppm)

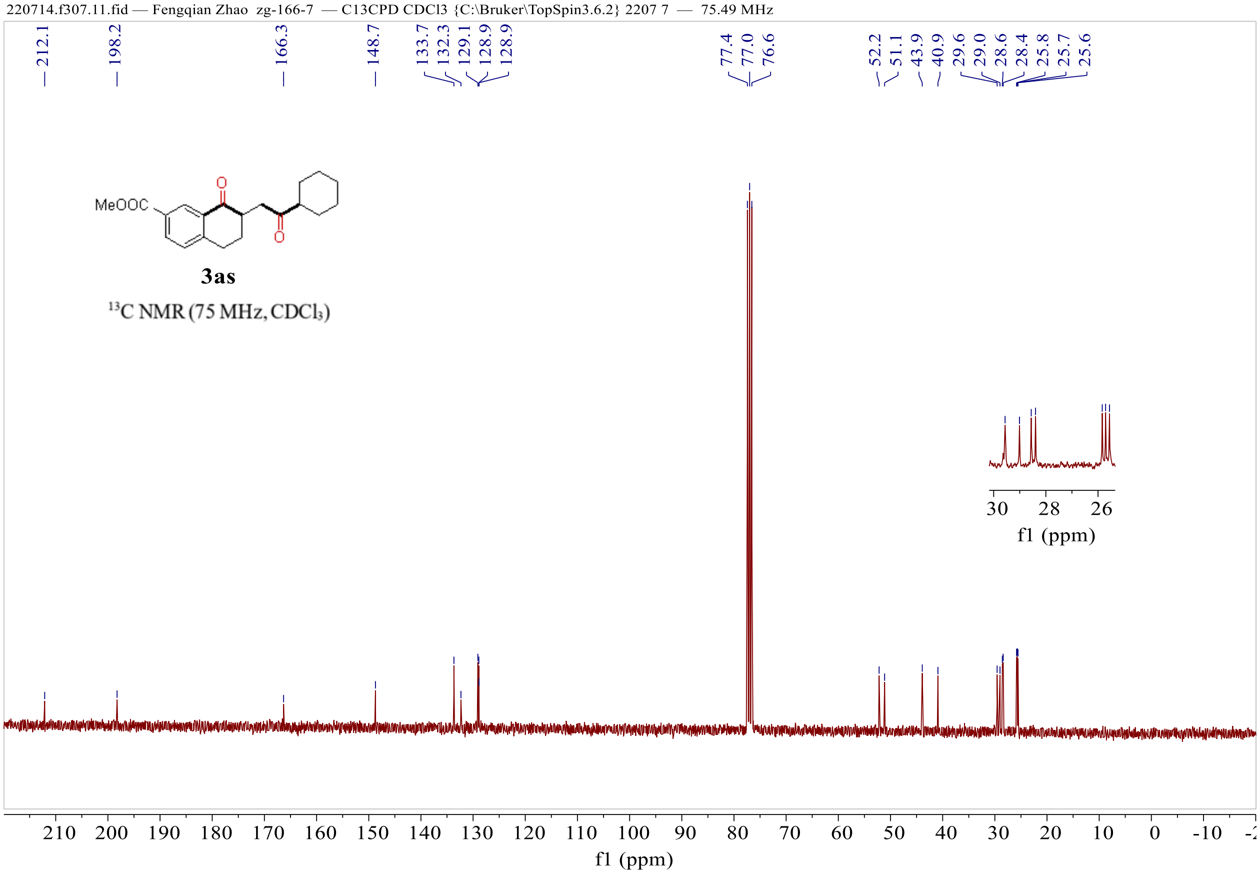

220714.f307.11.fid — Fengqian Zhao zg-166-7 — C13CPD CDCl3 {C:\Bruker\TopSpin3.6.2} 2207 7 — 75.49 MHz
MeOOC
O
O
3as
¹³C NMR (75 MHz, CDCl₃)
f1 (ppm)

220516.f303.10.fid — Fengqian Zhao zg-153-6 — PROTON CDCl3 {C:\Bruker\TopSpin3.6.2} 2205 3 — 300.20 MHz

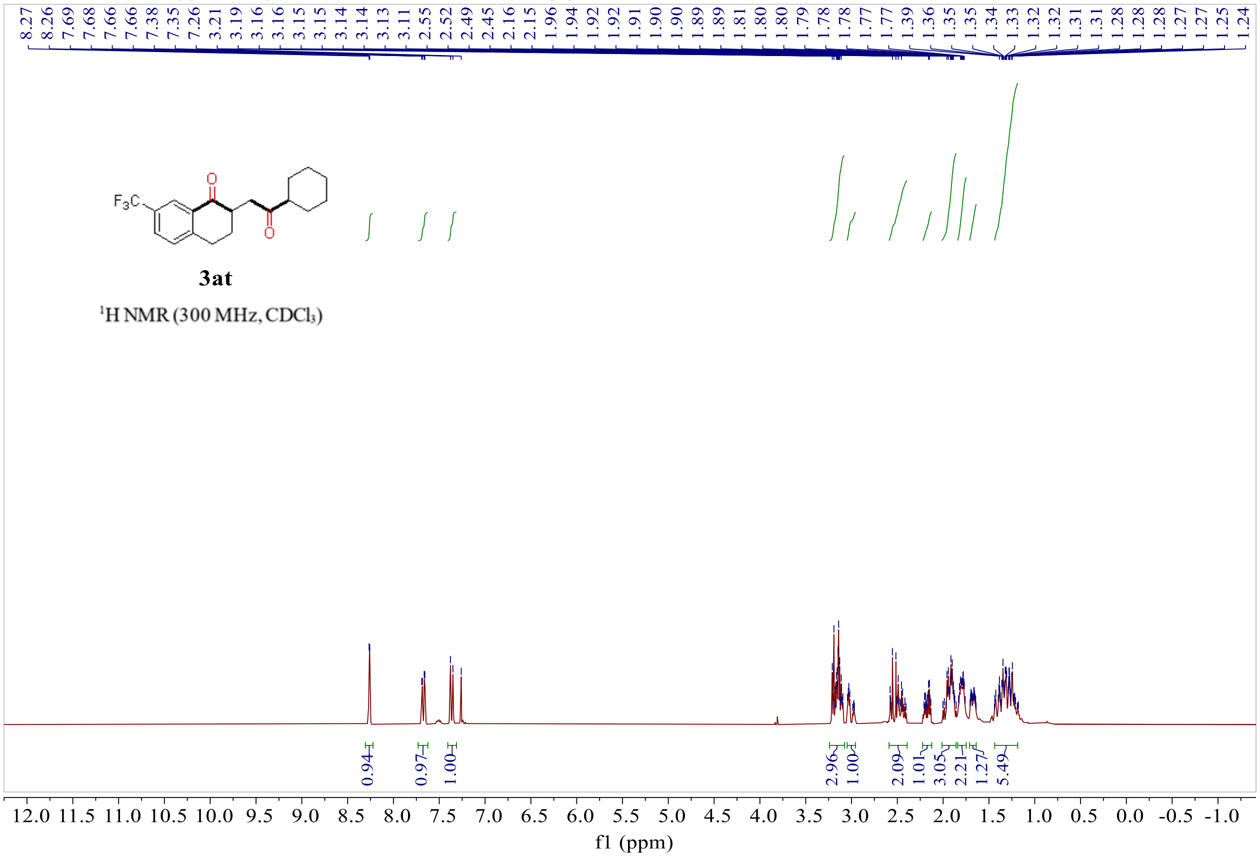

220517.337.11.fid — Patrizio Russo, 29-153-6 — Au13C CDCl3 {C:\Bruker\TopSpin3.6.2} 2205 37 — 75.48 MHz

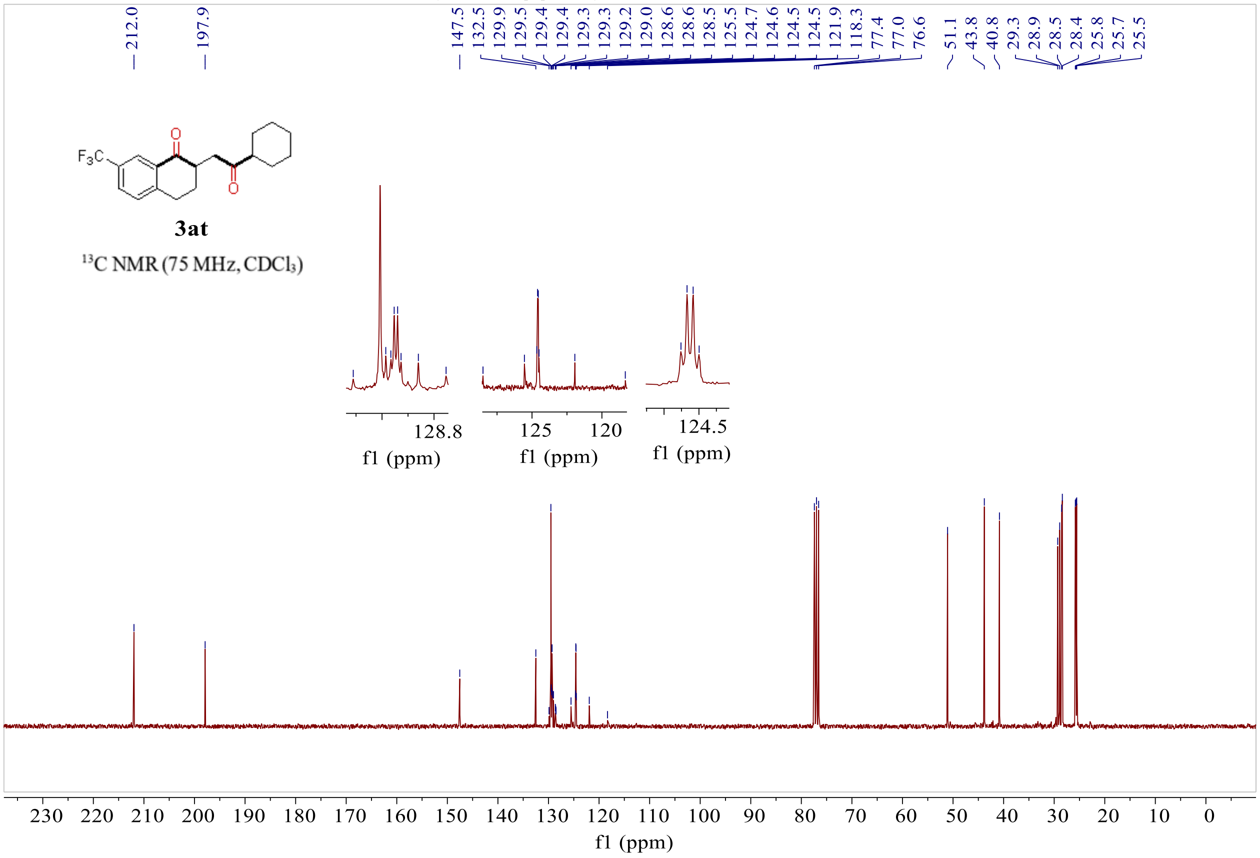

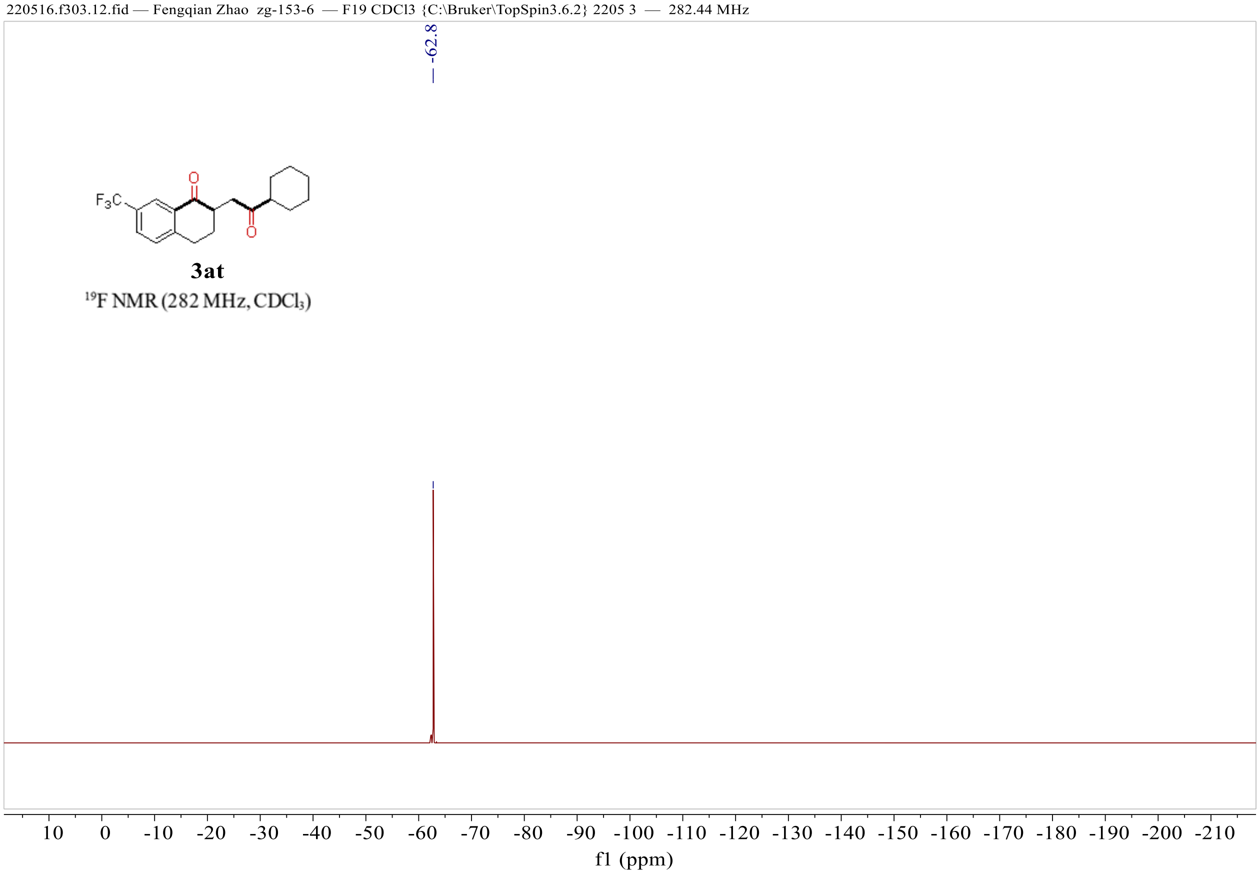
220516.f303.12.fid — Fengqian Zhao zg-153-6 — F19 CDCl3 {C:\Bruker\TopSpin3.6.2} 2205 3 — 282.44 MHz
-62.8
F3C
O
O
3at
19F NMR (282 MHz, CDCl3)
10 0 -10 -20 -30 -40 -50 -60 -70 -80 -90 -100 -110 -120 -130 -140 -150 -160 -170 -180 -190 -200 -210
f1 (ppm)

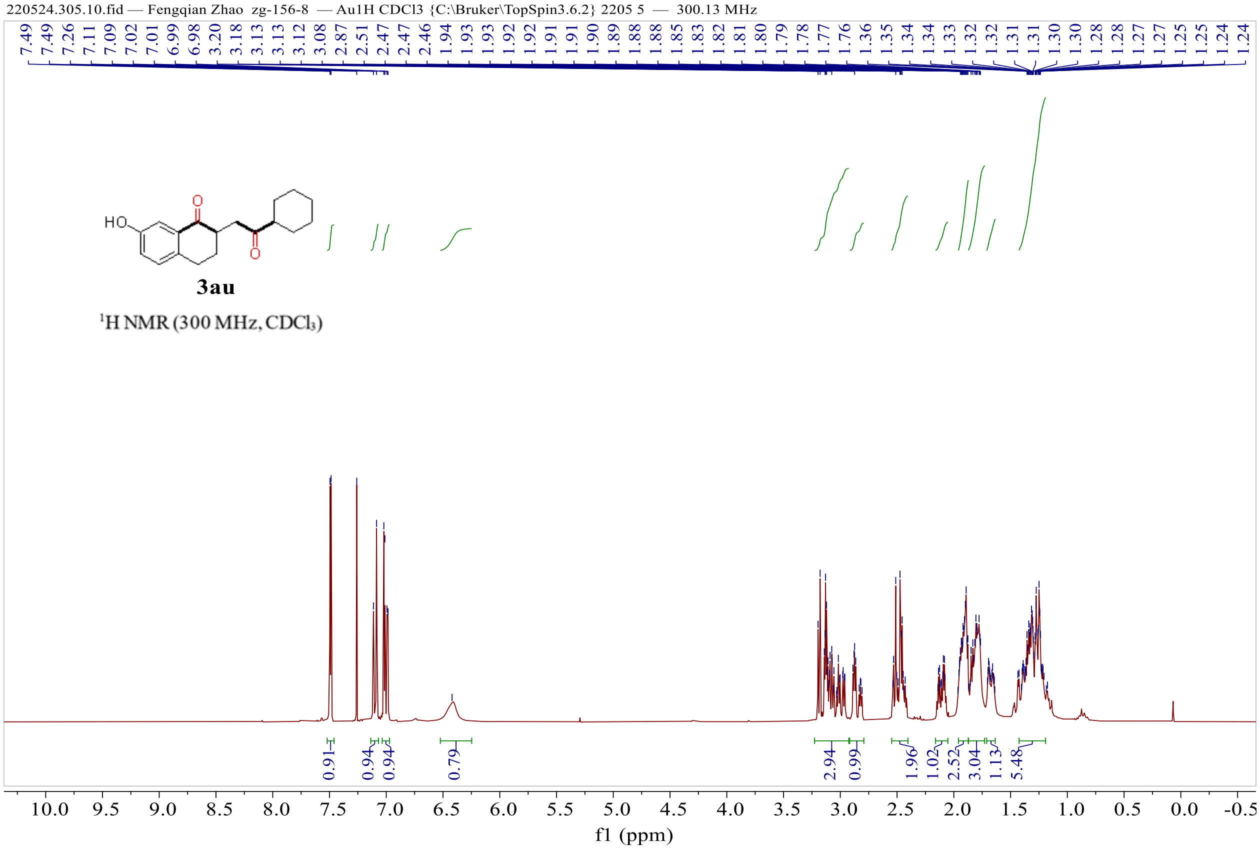

220524.305.10.fid — Fengqian Zhao zg-156-8 — Au1H CDCl3 {C:\Bruker\TopSpin3.6.2} 2205 5 — 300.13 MHz
7.49 7.49 7.26 7.11 7.09 7.02 7.01 6.99 6.98 3.20 3.18 3.13 3.13 3.12 3.08 2.87 2.51 2.47 2.47 2.46 1.94 1.93 1.93 1.92 1.92 1.91 1.91 1.90 1.89 1.88 1.88 1.85 1.83 1.82 1.81 1.80 1.79 1.78 1.77 1.76 1.36 1.35 1.34 1.34 1.33 1.32 1.32 1.31 1.31 1.30 1.28 1.28 1.27 1.25 1.25 1.24 1.24
HO
O
O
3au
^{1}H NMR (300 MHz, CDCl$_3$)
0.91 0.94 0.94 0.79 2.94 0.99 1.96 1.02 2.52 3.04 1.13 5.48
10.0 9.5 9.0 8.5 8.0 7.5 7.0 6.5 6.0 5.5 5.0 4.5 4.0 3.5 3.0 2.5 2.0 1.5 1.0 0.5 0.0 -0.5
f1 (ppm)

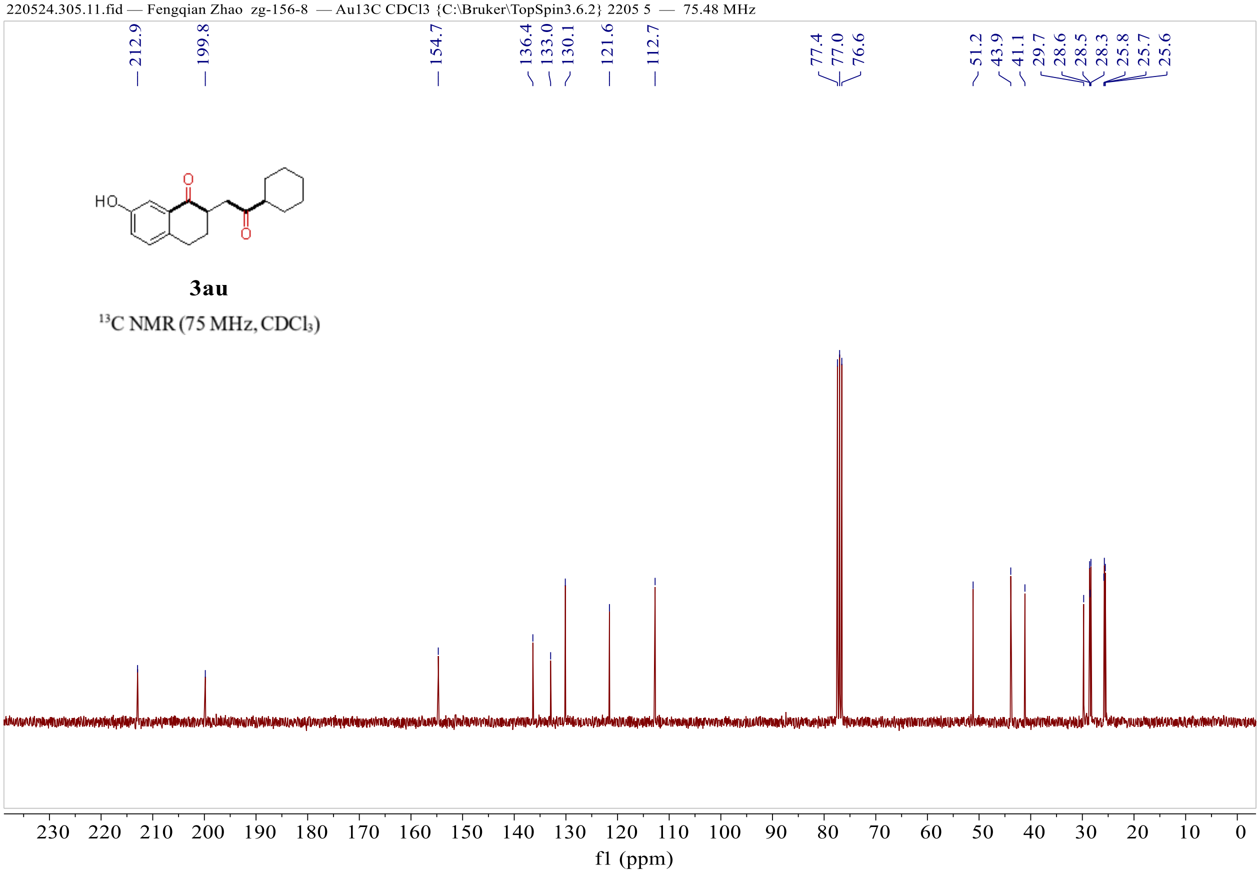

220524.305.11.fid — Fengqian Zhao zg-156-8 — Au13C CDCl3 {C:\Bruker\TopSpin3.6.2} 2205 5 — 75.48 MHz
212.9 199.8 154.7 136.4 133.0 130.1 121.6 112.7 77.4 77.0 76.6 51.2 43.9 41.1 29.7 28.6 28.5 28.3 25.8 25.7 25.6
HO
O
O
3au
^{13}C NMR (75 MHz, CDCl$_3$)
230 220 210 200 190 180 170 160 150 140 130 120 110 100 90 80 70 60 50 40 30 20 10 0
f1 (ppm)

220707.318.10.fid — Fengqian Zhao zg-162-6 — Au1H CDCl3 {C:\Bruker\TopSpin3.6.2} 2207 18 — 300.13 MHz

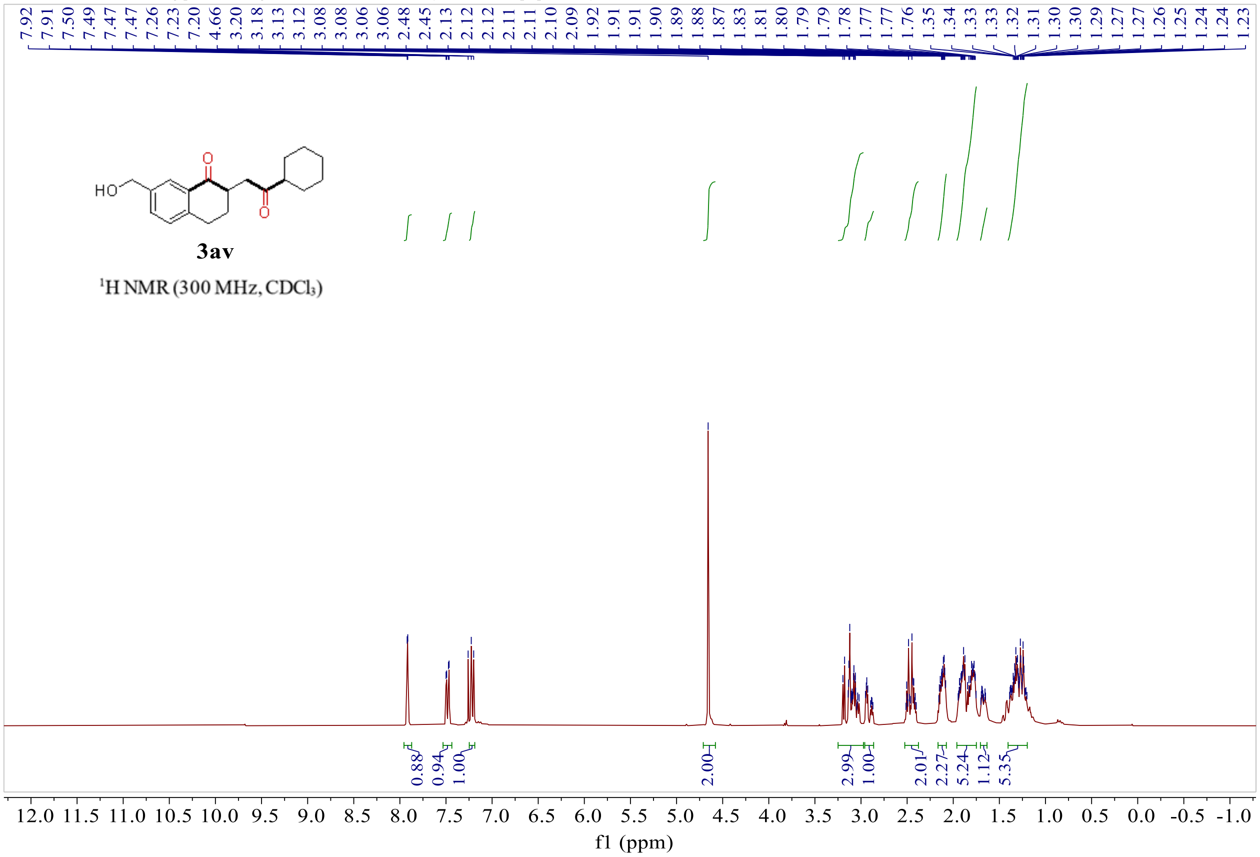

220707.318.6.fid — Fengqian Zhao zg-162-6 — Au13C CDCl3 {C:\Bruker\TopSpin3.6.2} 2207 18 — 75.48 MHz

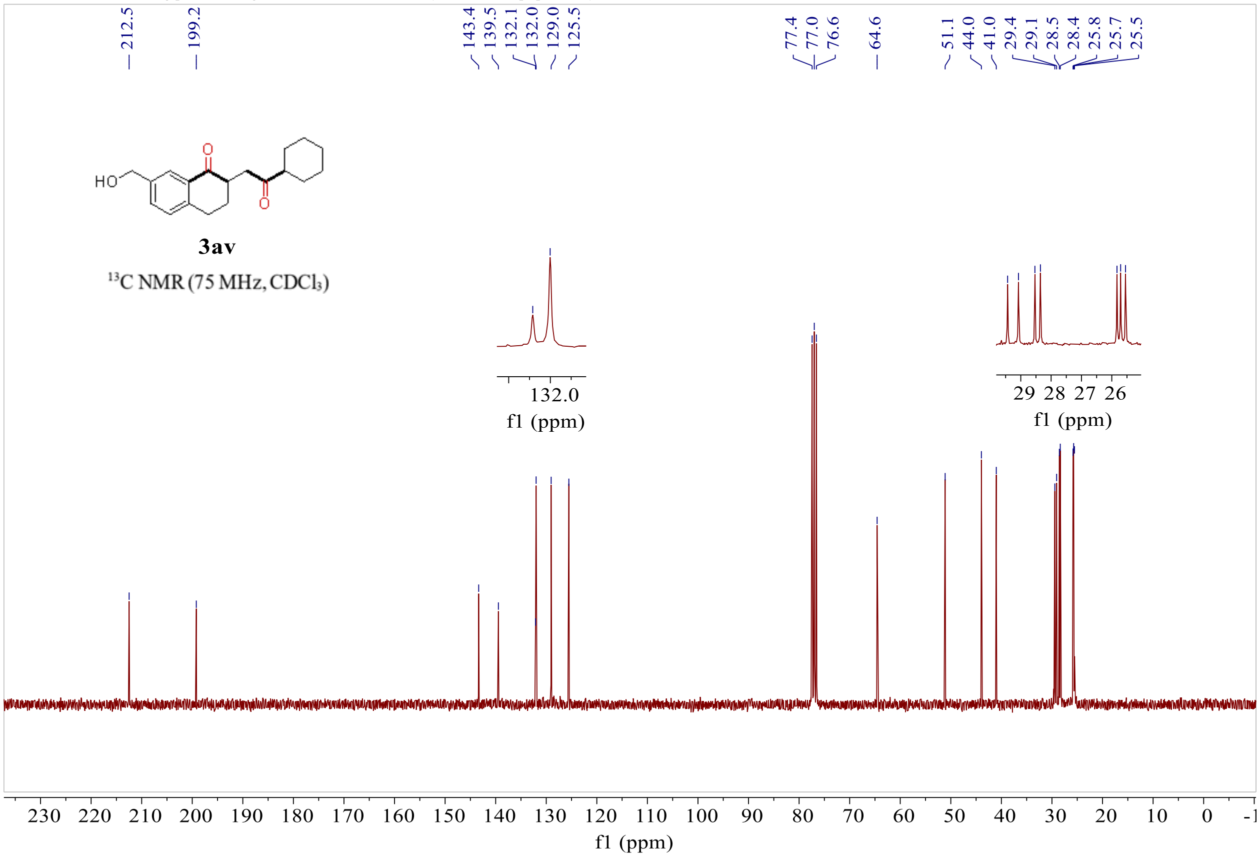

220714.f308.10.fid — Fengqian Zhao zg-166-1 — PROTON CDCl3 {C:\Bruker\TopSpin3.6.2} 2207 8 — 300.20 MHz

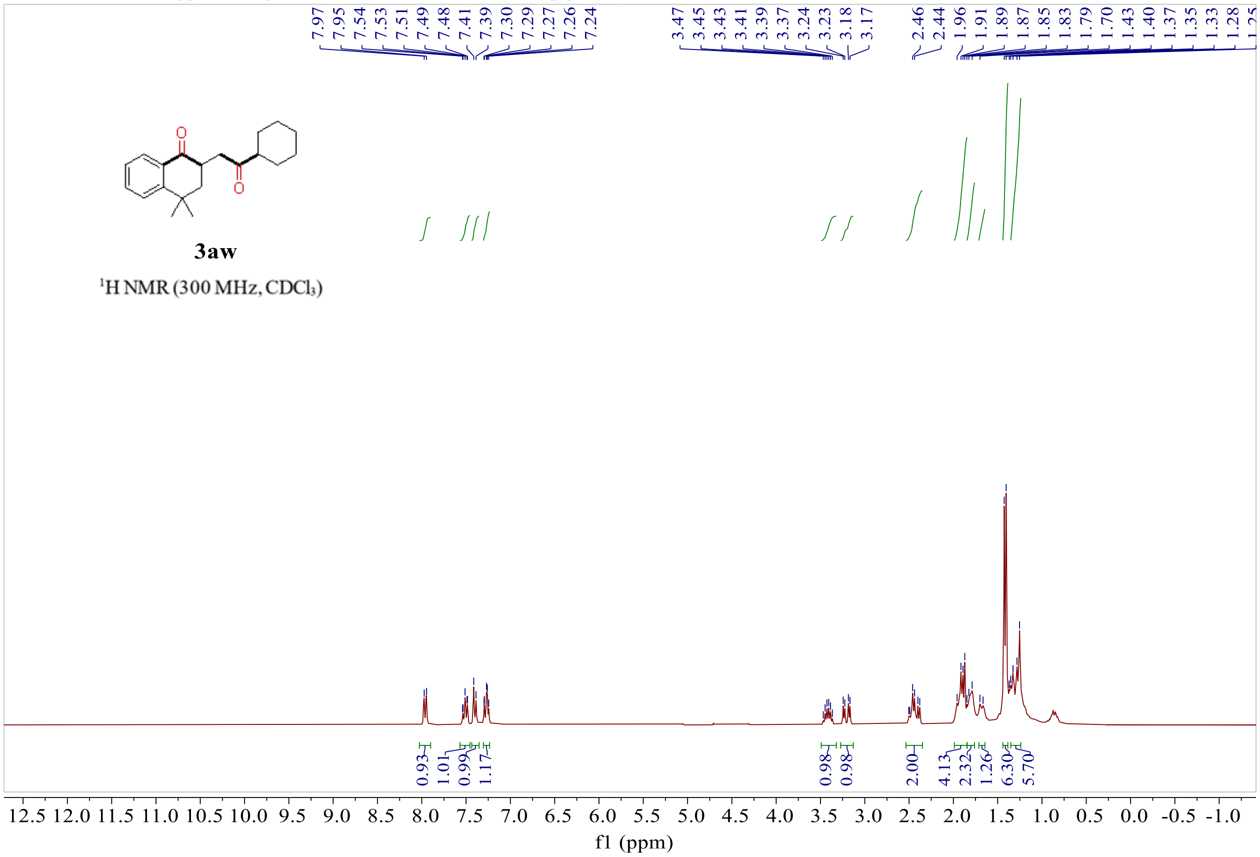

220714.f308.11.fid — Fengqian Zhao zg-166-1 — C13CPD CDCl3 {C:\Bruker\TopSpin3.6.2} 2207 8 — 75.49 MHz

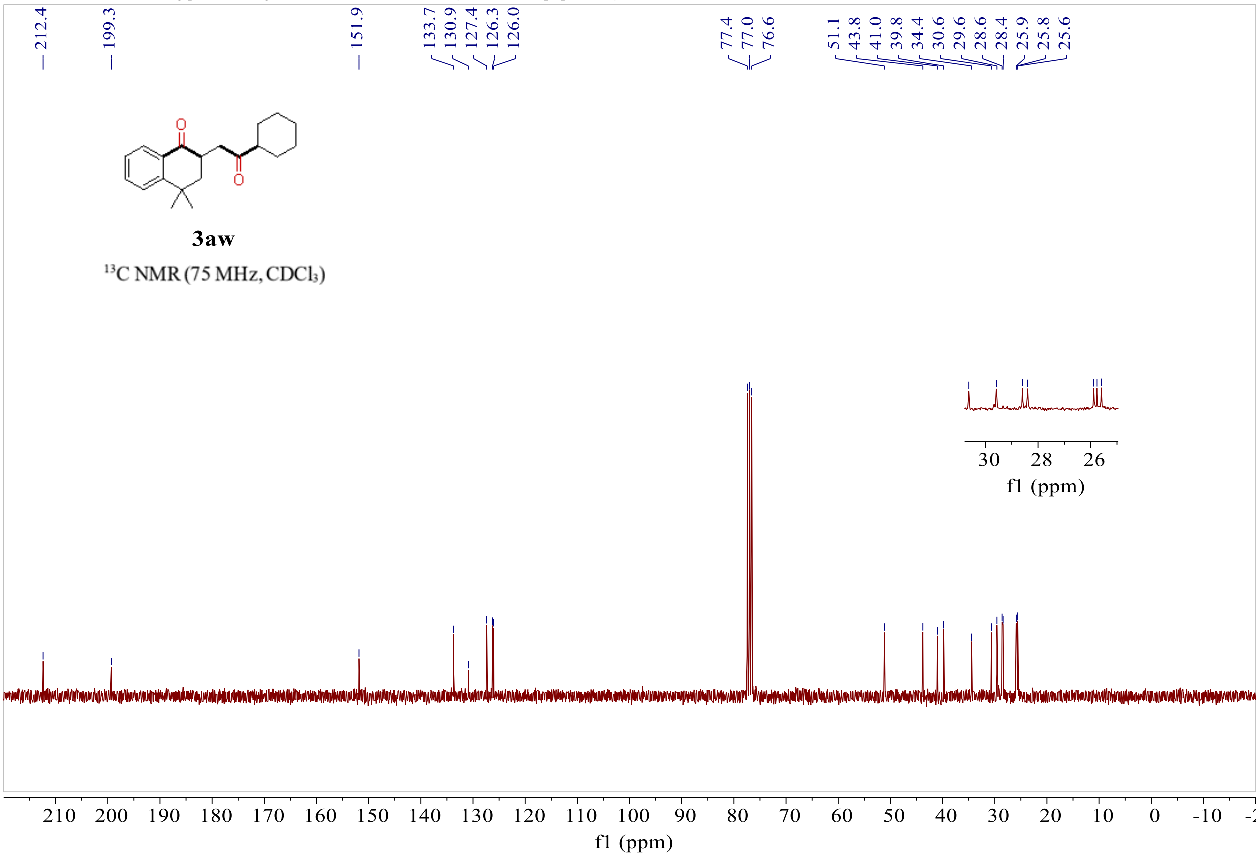

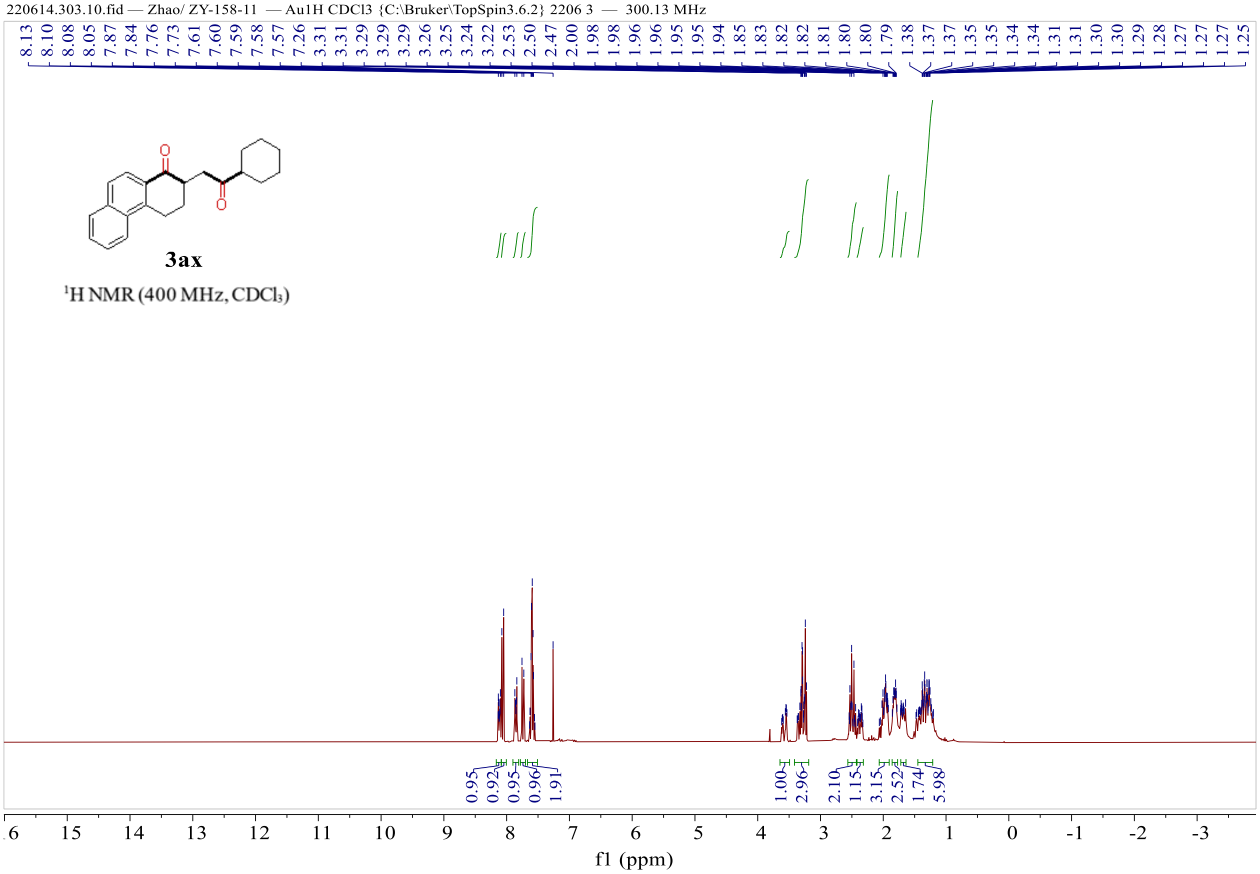

220614.303.10.fid — Zhao/ ZY-158-11 — Au1H CDCl3 {C:\Bruker\TopSpin3.6.2} 2206 3 — 300.13 MHz
8.13 8.10 8.08 8.05 7.87 7.84 7.76 7.73 7.61 7.60 7.59 7.58 7.57 7.26 3.31 3.31 3.29 3.29 3.29 3.26 3.25 3.24 3.22 2.53 2.50 2.47 2.00 1.98 1.98 1.96 1.96 1.95 1.95 1.94 1.85 1.83 1.82 1.82 1.81 1.80 1.80 1.79 1.38 1.37 1.37 1.35 1.35 1.34 1.34 1.31 1.31 1.30 1.30 1.29 1.28 1.27 1.27 1.27 1.25
3ax
1H NMR (400 MHz, CDCl3)
0.95 0.92 0.95 0.96 1.91 1.00 2.96 2.10 1.15 3.15 2.52 1.74 5.98
f1 (ppm)

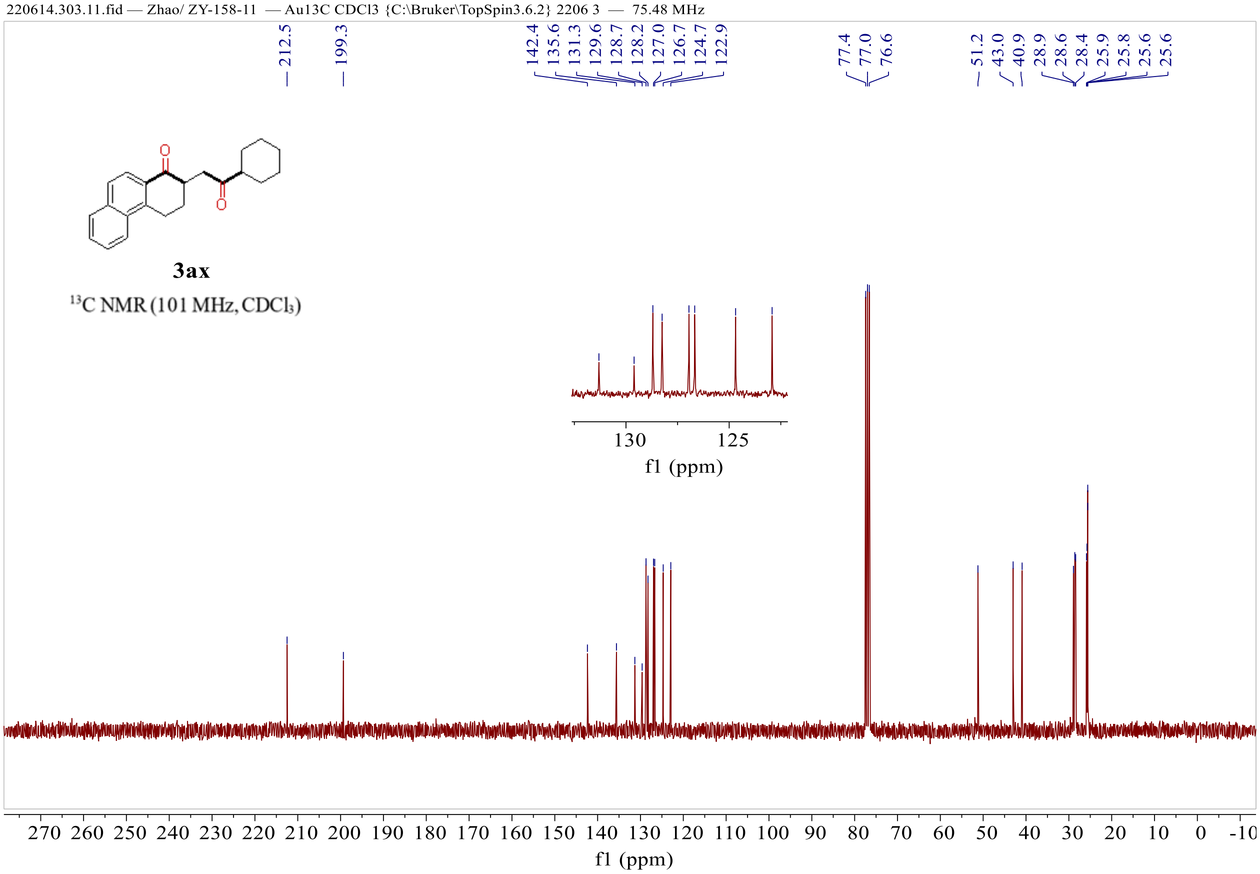

220614.303.11.fid — Zhao/ ZY-158-11 — Au13C CDCl3 {C:\Bruker\TopSpin3.6.2} 2206 3 — 75.48 MHz
212.5 199.3 142.4 135.6 131.3 129.6 128.7 128.2 127.0 126.7 124.7 122.9 77.4 77.0 76.6 51.2 43.0 40.9 28.9 28.6 28.4 25.9 25.8 25.6 25.6
3ax
13C NMR (101 MHz, CDCl3)
f1 (ppm)

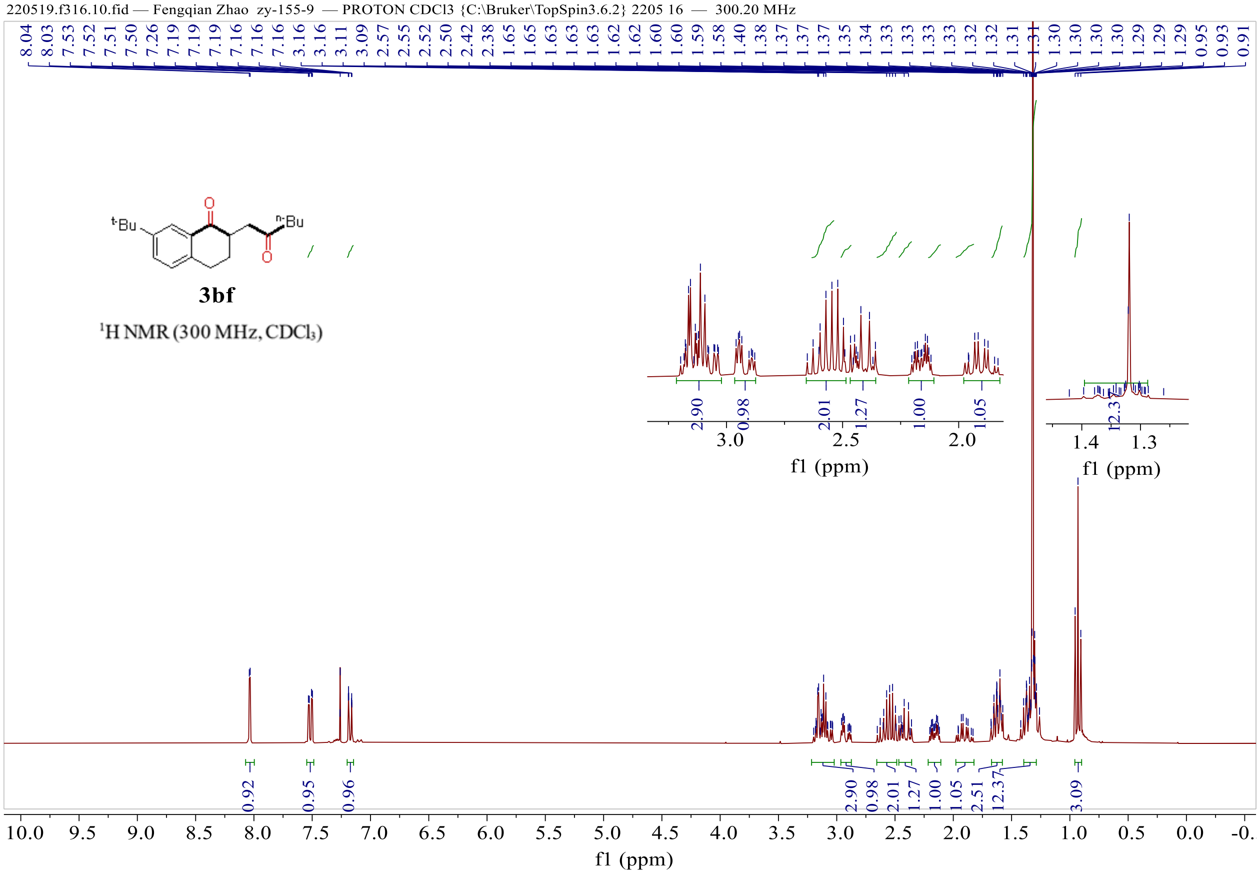
220519.f316.10.fid — Fengqian Zhao zy-155-9 — PROTON CDCl3 {C:\Bruker\TopSpin3.6.2} 2205 16 — 300.20 MHz
t-Bu
n-Bu
3bf
¹H NMR (300 MHz, CDCl₃)
f1 (ppm)

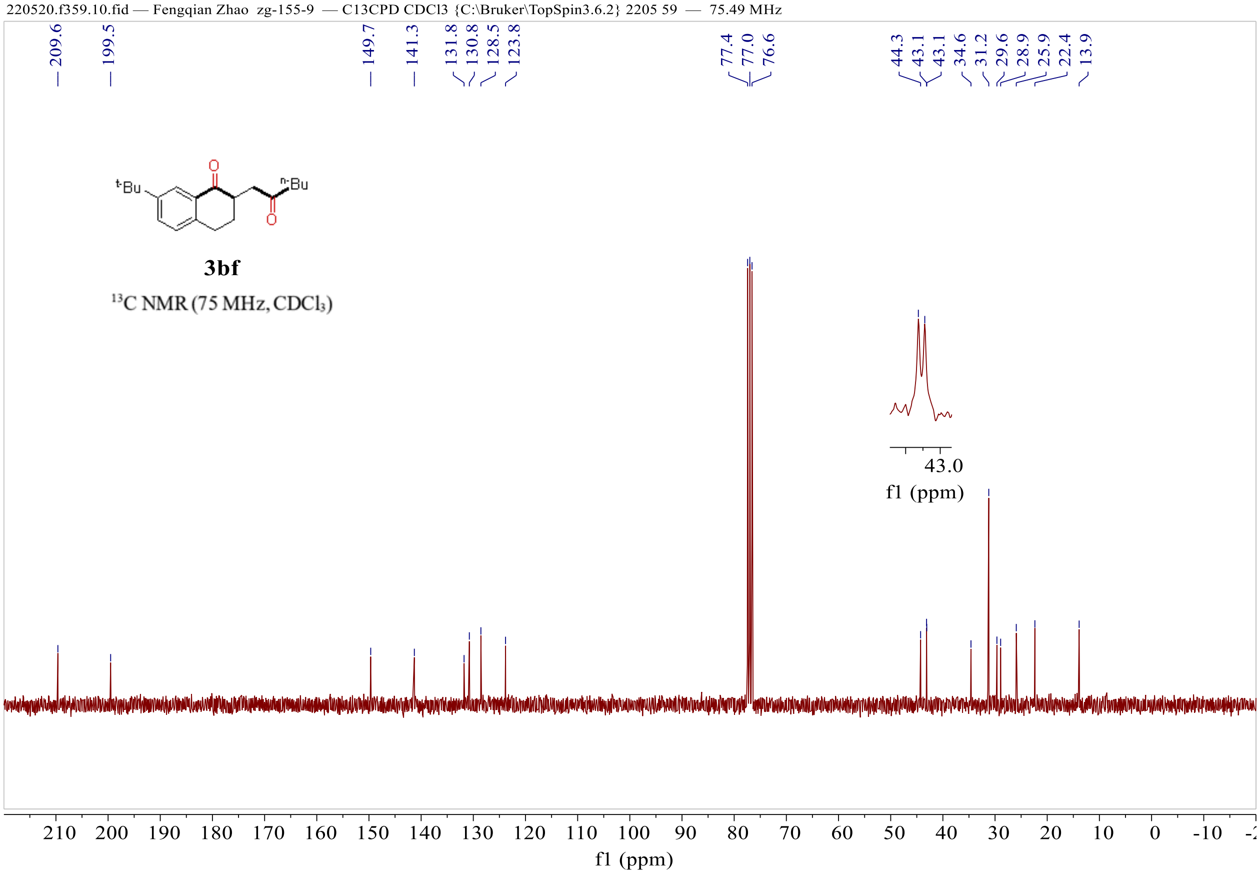
220520.f359.10.fid — Fengqian Zhao zg-155-9 — C13CPD CDCl3 {C:\Bruker\TopSpin3.6.2} 2205 59 — 75.49 MHz
209.6
199.5
149.7
141.3
131.8
130.8
128.5
123.8
77.4
77.0
76.6
44.3
43.1
43.1
34.6
31.2
29.6
28.9
25.9
22.4
13.9
t-Bu
n-Bu
3bf
¹³C NMR (75 MHz, CDCl₃)
f1 (ppm)

220705.315.10.fid — Fengqian Zhao zg-164-5 — Au1H CDCl3 {C:\Bruker\TopSpin3.6.2} 2207 15 — 300.13 MHz

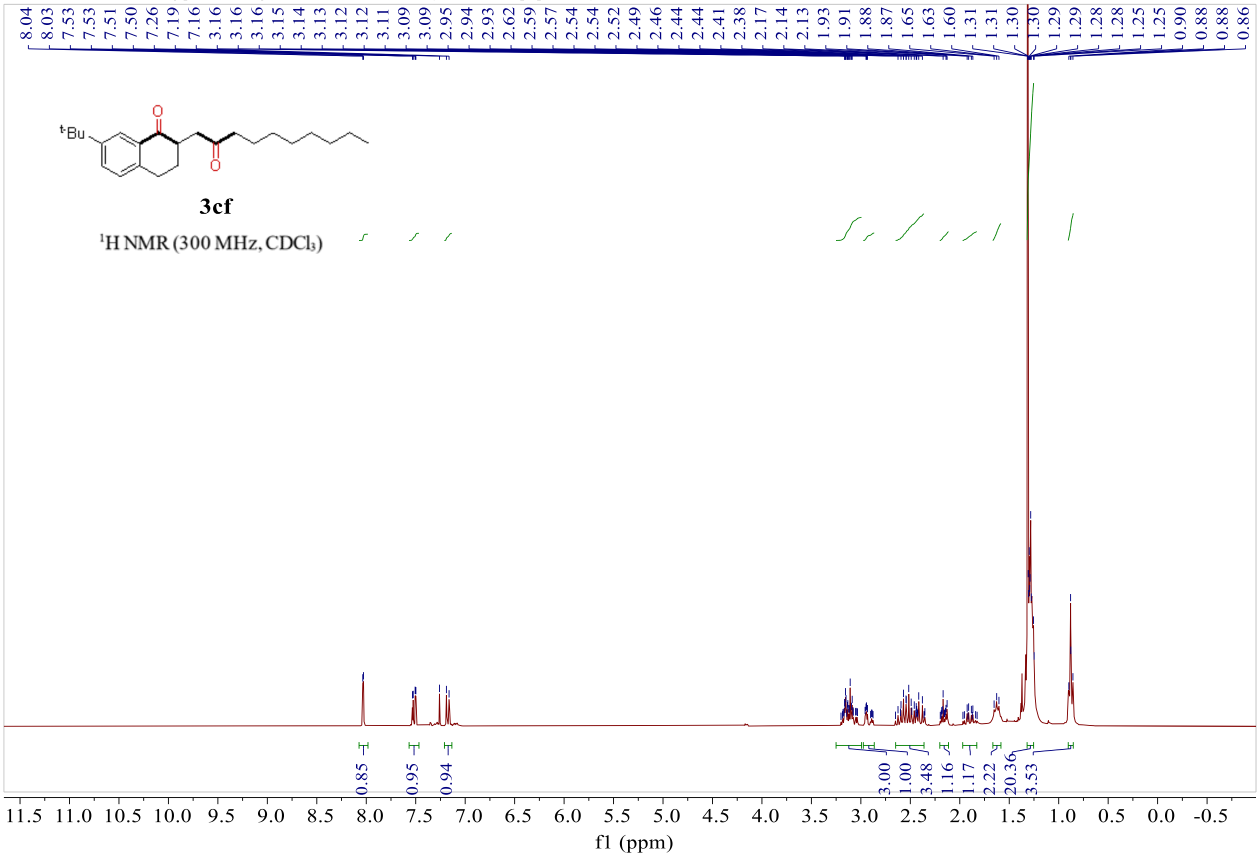

3cf

^{1}H NMR (300 MHz, $CDCl_3$)

220705.315.11.fid — Fengqian Zhao zg-164-5 — Au13C CDCl3 {C:\Bruker\TopSpin3.6.2} 2207 15 — 75.48 MHz

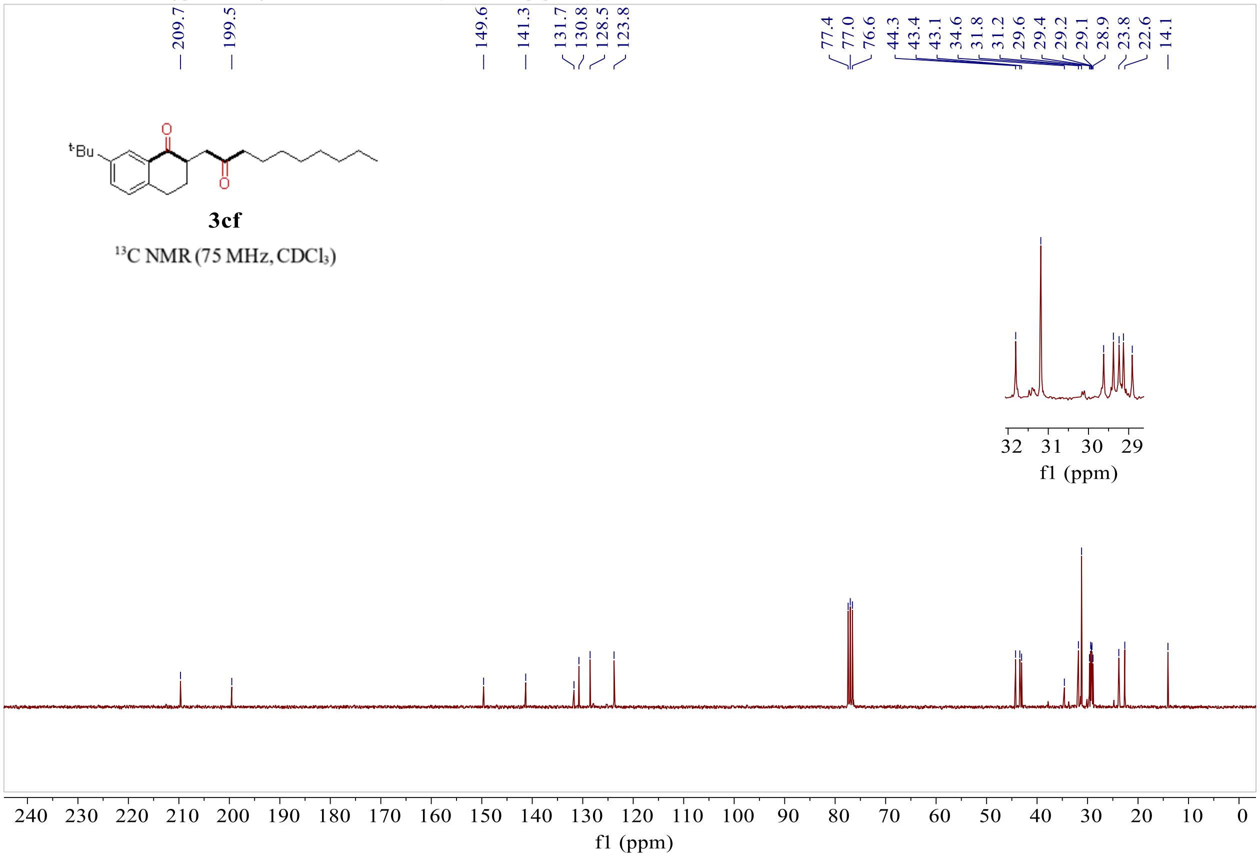

3cf

^{13}C NMR (75 MHz, $CDCl_3$)

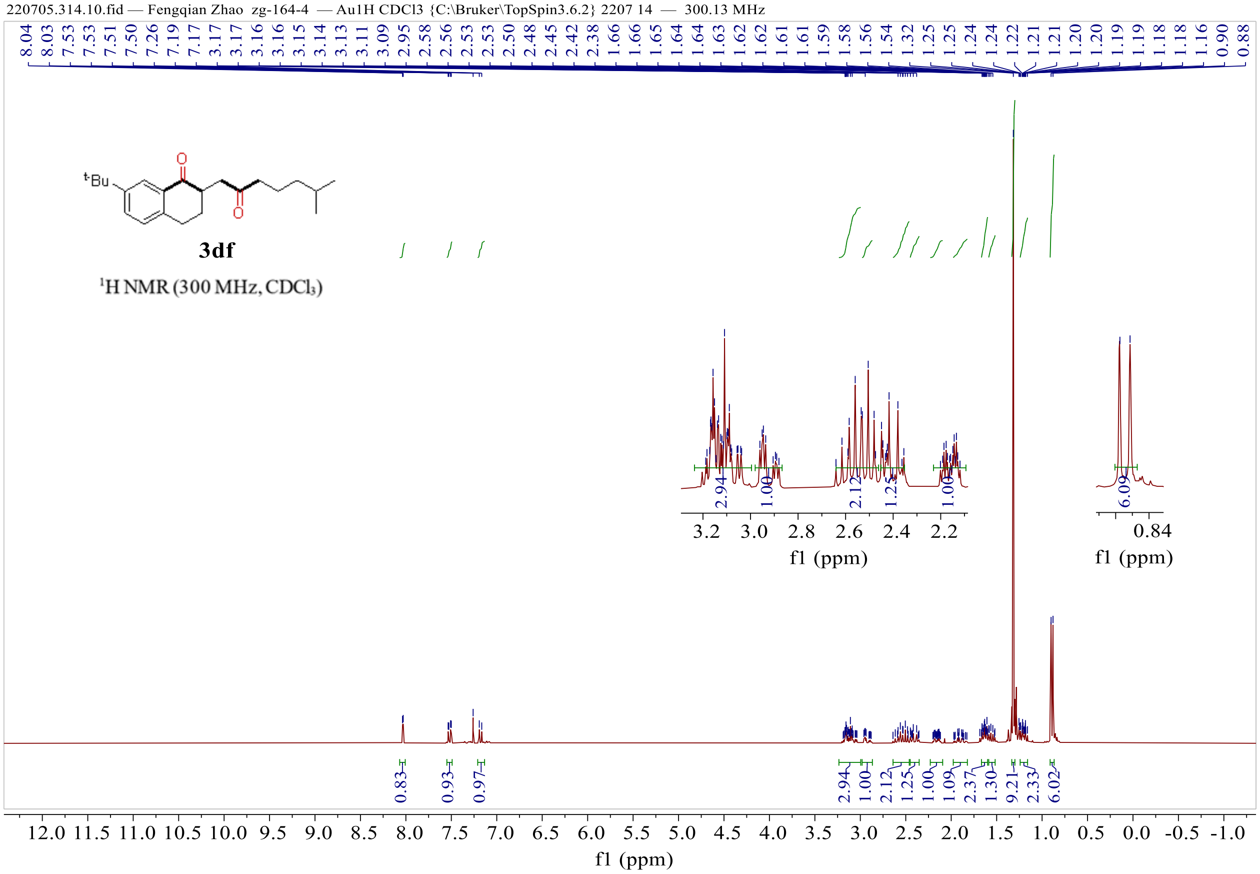
220705.314.10.fid — Fengqian Zhao zg-164-4 — Au1H CDCl3 {C:\Bruker\TopSpin3.6.2} 2207 14 — 300.13 MHz
t-Bu
3df
1H NMR (300 MHz, CDCl3)
f1 (ppm)

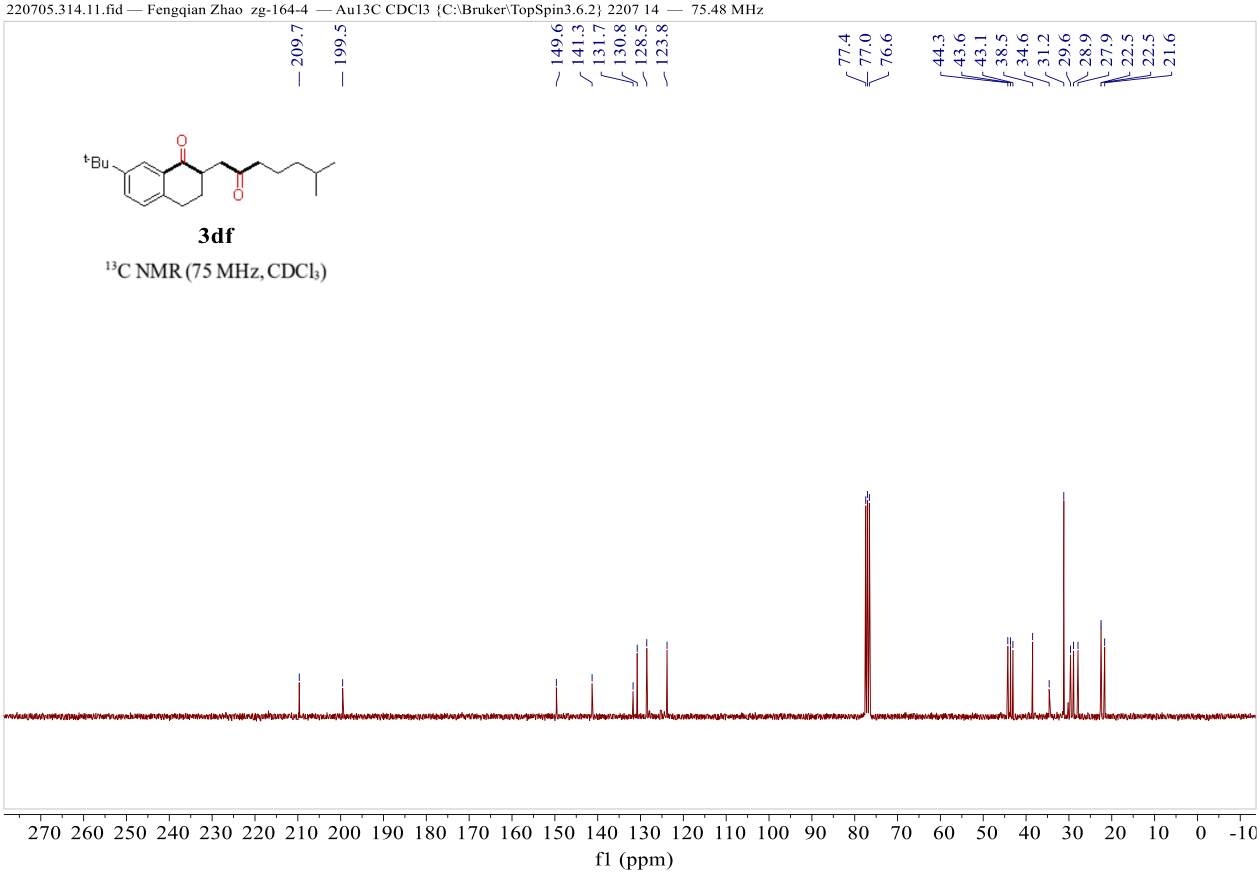
220705.314.11.fid — Fengqian Zhao zg-164-4 — Au13C CDCl3 {C:\Bruker\TopSpin3.6.2} 2207 14 — 75.48 MHz
209.7
199.5
149.6
141.3
131.7
130.8
128.5
123.8
77.4
77.0
76.6
44.3
43.6
43.1
38.5
34.6
31.2
29.6
28.9
27.9
22.5
22.5
21.6
t-Bu
3df
13C NMR (75 MHz, CDCl3)
f1 (ppm)

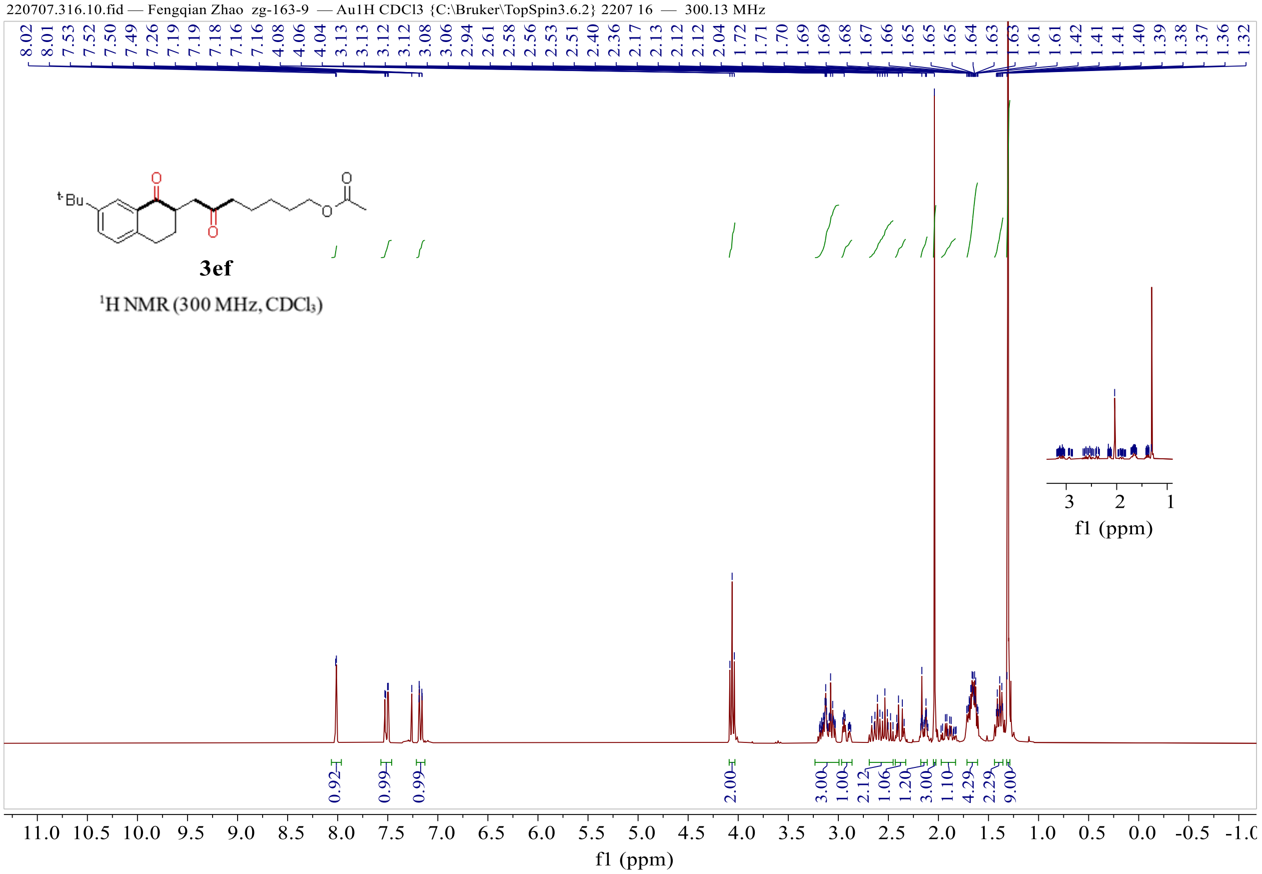
220707.316.10.fid — Fengqian Zhao zg-163-9 — Au1H CDCl3 {C:\Bruker\TopSpin3.6.2} 2207 16 — 300.13 MHz
8.02 8.01 7.53 7.52 7.50 7.49 7.26 7.19 7.19 7.18 7.16 7.16 4.08 4.06 4.04 3.13 3.13 3.12 3.12 3.08 3.06 2.94 2.61 2.58 2.56 2.53 2.51 2.40 2.36 2.17 2.13 2.12 2.12 2.04 1.72 1.71 1.70 1.69 1.69 1.68 1.67 1.66 1.65 1.65 1.65 1.64 1.63 1.63 1.61 1.61 1.42 1.41 1.41 1.40 1.39 1.38 1.37 1.36 1.32
t-Bu
3ef
1H NMR (300 MHz, CDCl3)
0.92 0.99 0.99 2.00 3.00 1.00 2.12 1.06 1.20 3.00 1.10 4.29 2.29 9.00
f1 (ppm)

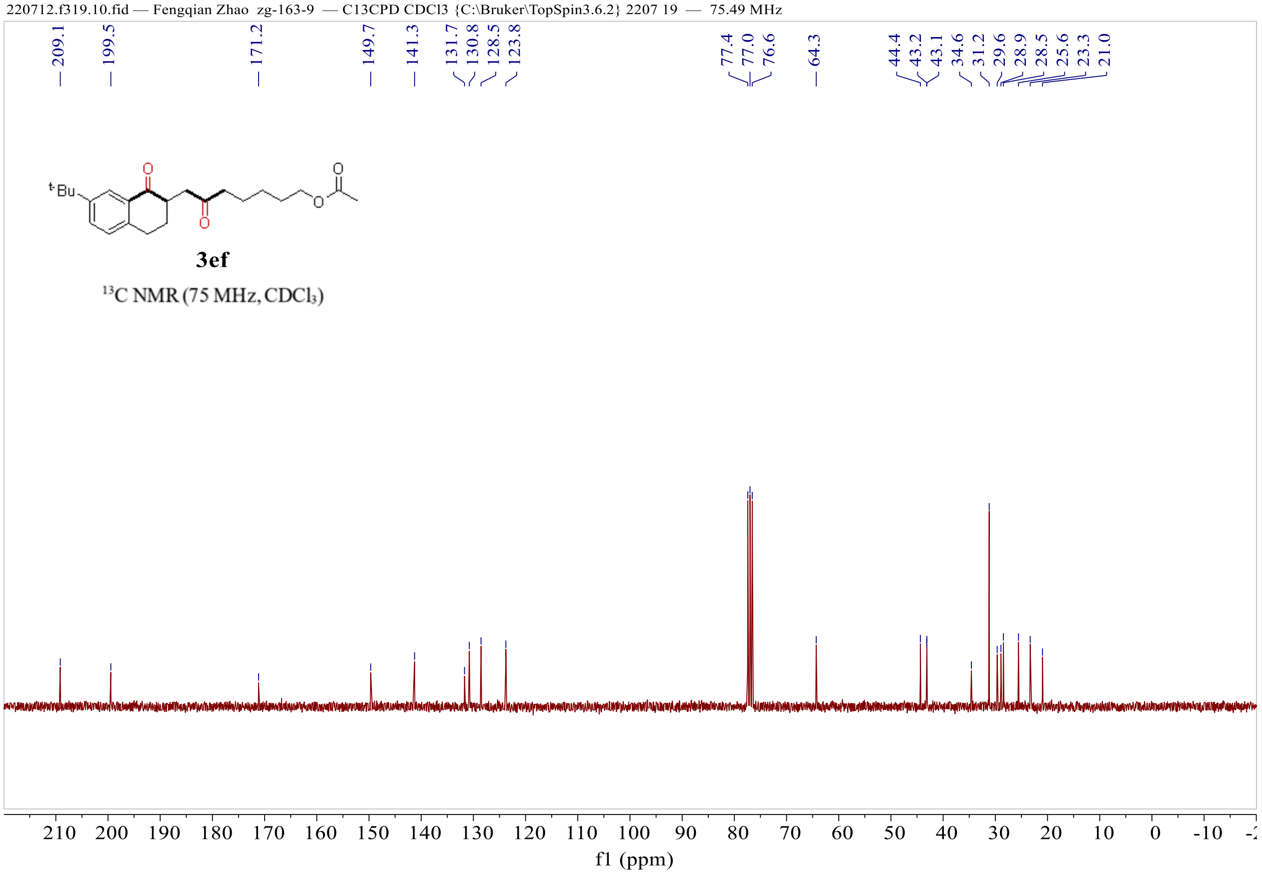
220712.f319.10.fid — Fengqian Zhao zg-163-9 — C13CPD CDCl3 {C:\Bruker\TopSpin3.6.2} 2207 19 — 75.49 MHz
209.1 199.5 171.2 149.7 141.3 131.7 130.8 128.5 123.8 77.4 77.0 76.6 64.3 44.4 43.2 43.1 34.6 31.2 29.6 28.9 28.5 25.6 23.3 21.0
t-Bu
3ef
13C NMR (75 MHz, CDCl3)
f1 (ppm)

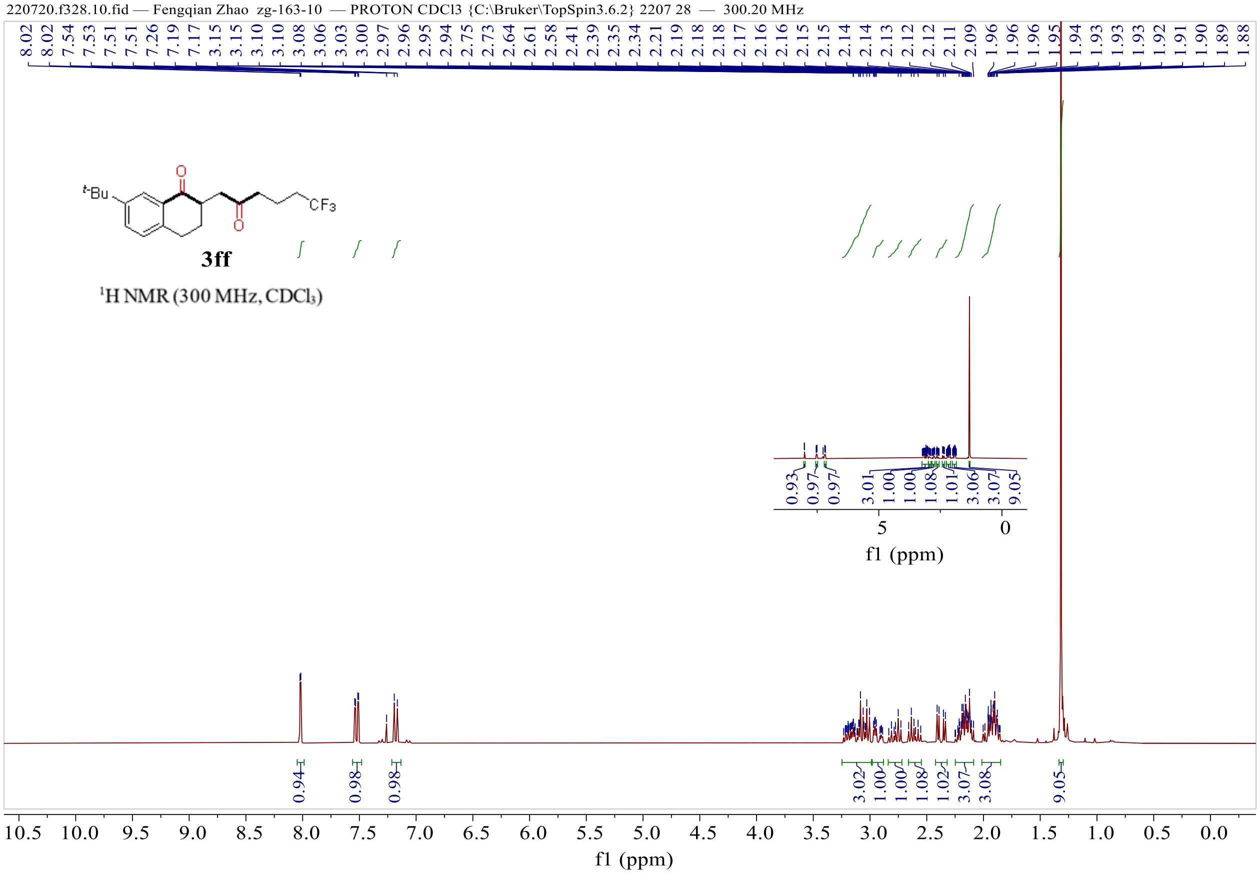
220720.f328.10.fid — Fengqian Zhao zg-163-10 — PROTON CDCl3 {C:\Bruker\TopSpin3.6.2} 2207 28 — 300.20 MHz
8.02 8.02 7.54 7.53 7.51 7.51 7.26 7.19 7.17 3.15 3.15 3.10 3.10 3.08 3.06 3.03 3.00 2.97 2.96 2.95 2.94 2.75 2.73 2.64 2.61 2.58 2.41 2.39 2.35 2.34 2.21 2.19 2.18 2.18 2.17 2.16 2.16 2.15 2.15 2.14 2.14 2.13 2.12 2.12 2.11 2.09 1.96 1.96 1.96 1.95 1.94 1.93 1.93 1.93 1.92 1.91 1.90 1.89 1.88
t-Bu
O
O
CF3
3ff
1H NMR (300 MHz, CDCl3)
0.93 0.97 0.97 3.01 1.00 1.00 1.08 1.01 3.06 3.07 9.05
5
0
f1 (ppm)
0.94 0.98 0.98 3.02 1.00 1.00 1.08 1.02 3.07 3.08 9.05
10.5 10.0 9.5 9.0 8.5 8.0 7.5 7.0 6.5 6.0 5.5 5.0 4.5 4.0 3.5 3.0 2.5 2.0 1.5 1.0 0.5 0.0
f1 (ppm)

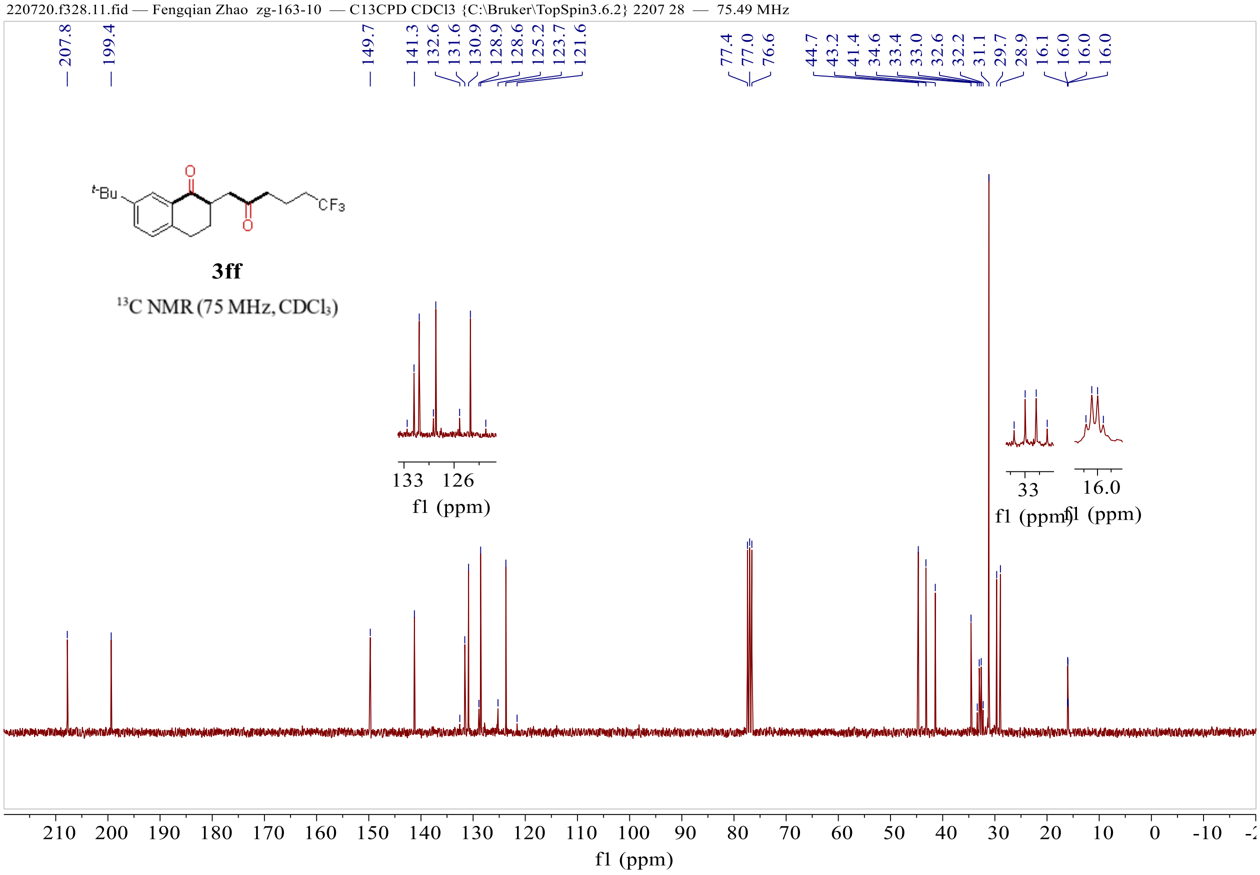
220720.f328.11.fid — Fengqian Zhao zg-163-10 — C13CPD CDCl3 {C:\Bruker\TopSpin3.6.2} 2207 28 — 75.49 MHz
207.8 199.4 149.7 141.3 132.6 131.6 130.9 128.9 128.6 125.2 123.7 121.6 77.4 77.0 76.6 44.7 43.2 41.4 34.6 33.4 33.0 32.6 32.2 31.1 29.7 28.9 16.1 16.0 16.0 16.0
t-Bu
O
O
CF3
3ff
13C NMR (75 MHz, CDCl3)
133 126
f1 (ppm)
33
f1 (ppm)
16.0
f1 (ppm)
210 200 190 180 170 160 150 140 130 120 110 100 90 80 70 60 50 40 30 20 10 0 -10
f1 (ppm)

220720.f328.12.fid — Fengqian Zhao zg-163-10 — F19 CDCl3 {C:\Bruker\TopSpin3.6.2} 2207 28 — 282.44 MHz

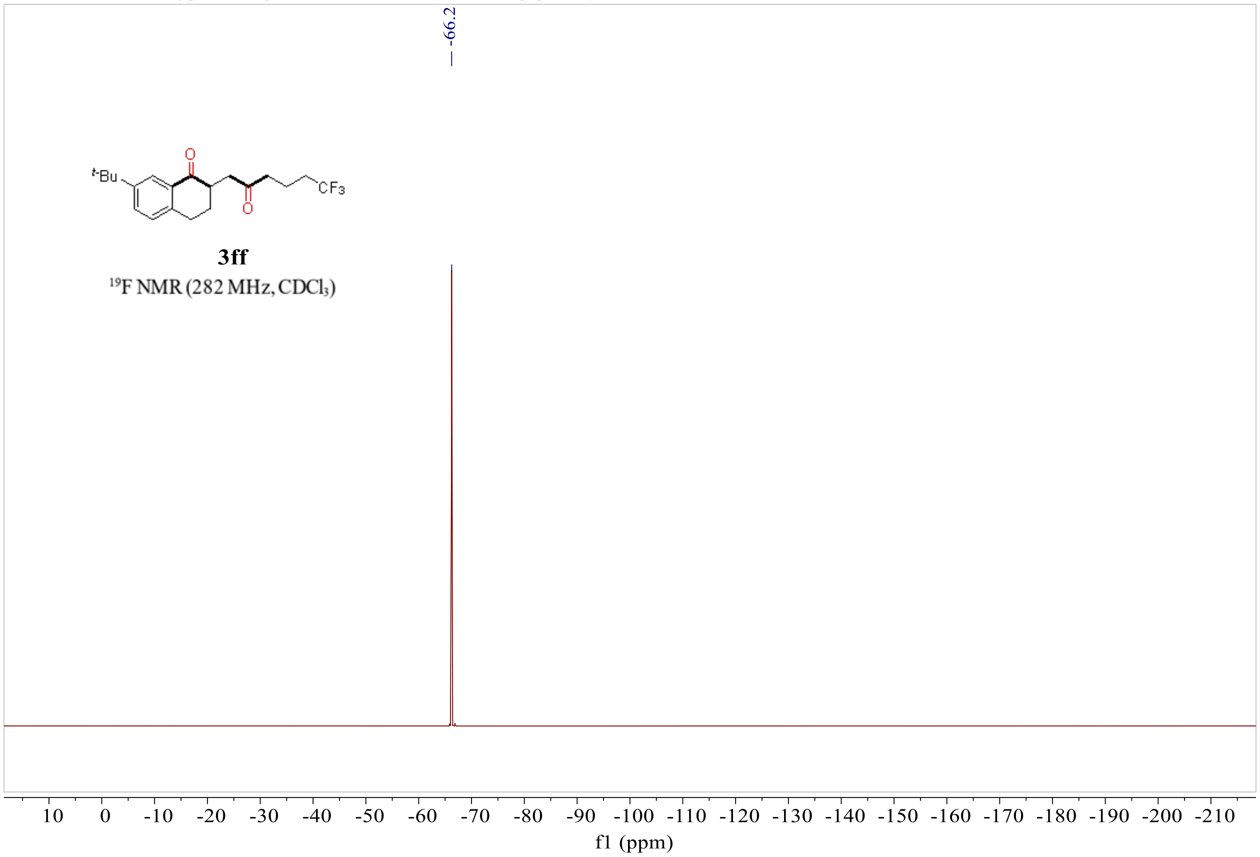

3ff

^{19}F NMR (282 MHz, $CDCl_3$)

220616.f312.10.fid — Zhao/ ZY-158-1 — PROTON MeOD {C:\Bruker\TopSpin3.6.2} 2206 12 — 300.20 MHz

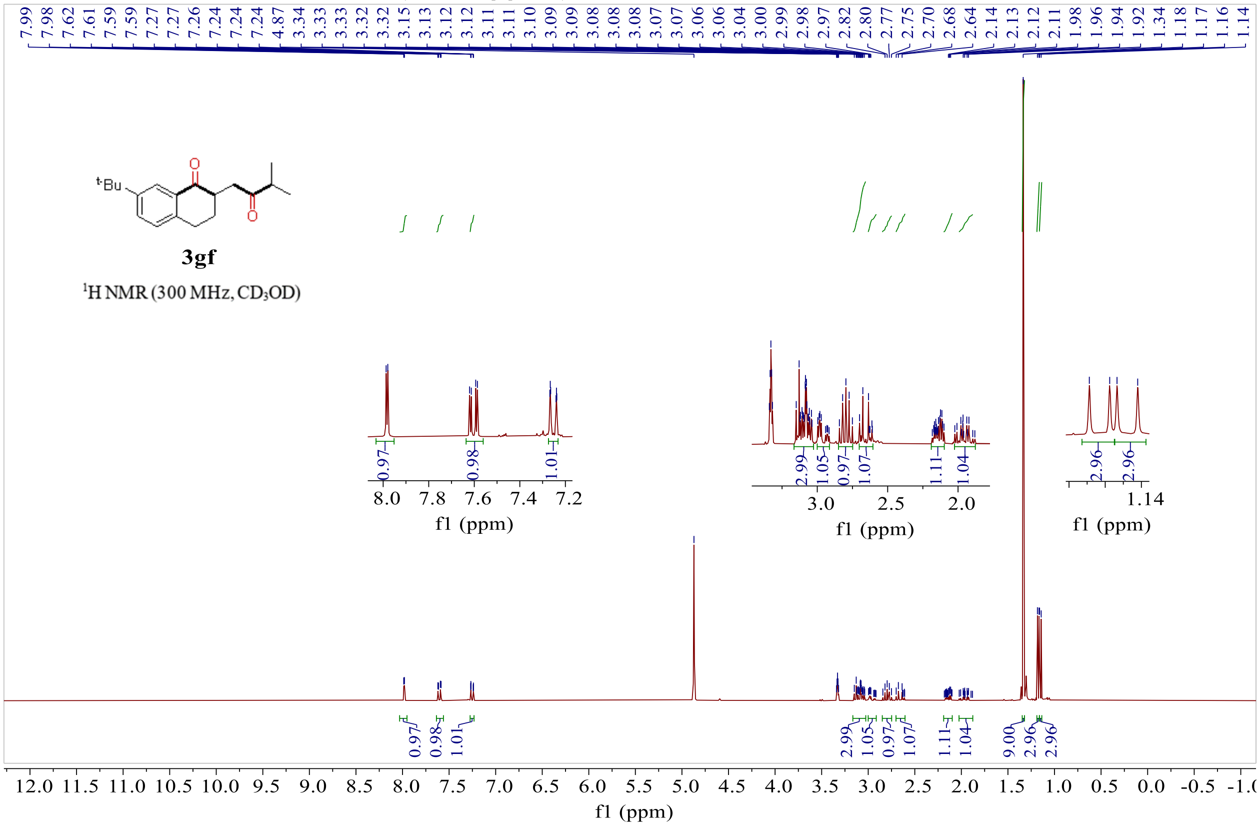

3gf

^{1}H NMR (300 MHz, CD_3OD)

220609.f306.11.fid — Zhao/ 29-151-1 — C13CPD CDCl3 {C:\Bruker\TopSpin3.6.2} 2206 6 — 75.49 MHz

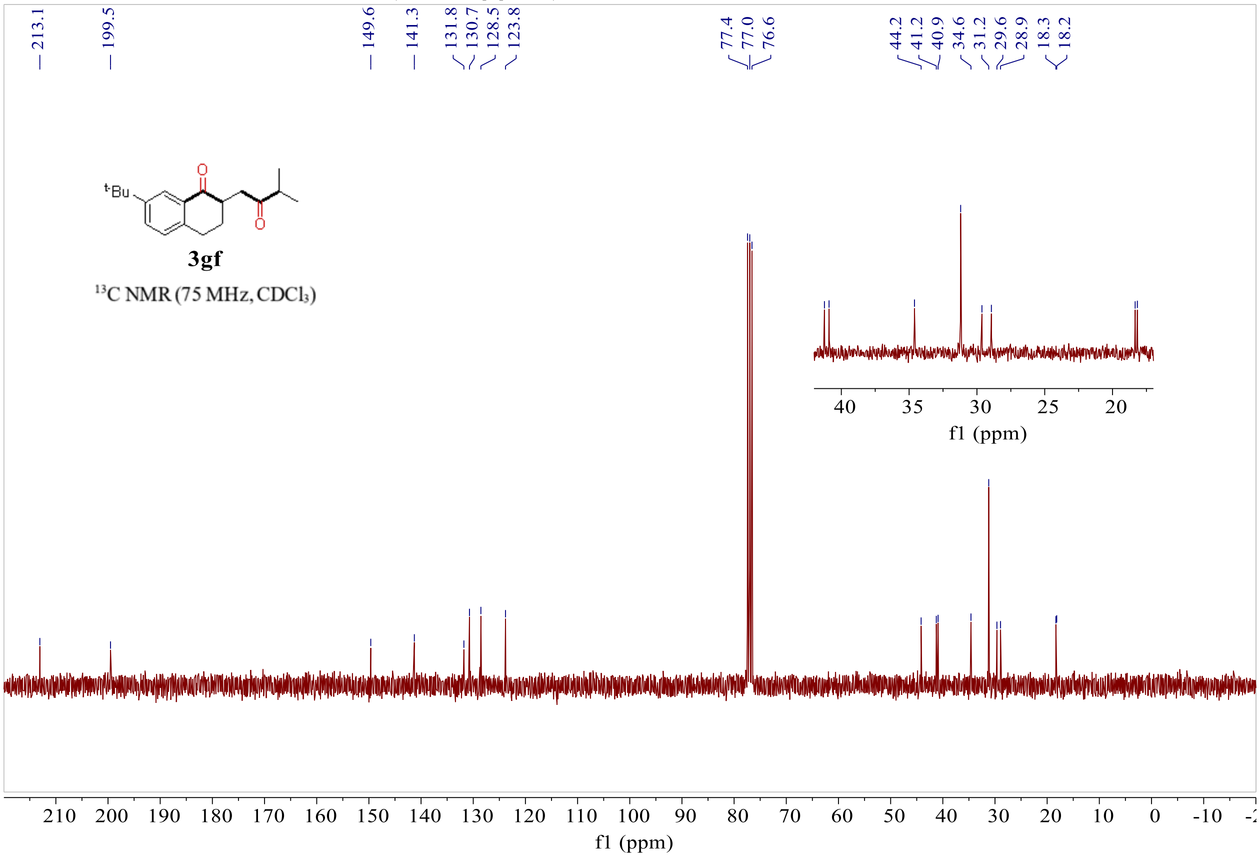

3gf

^{13}C NMR (75 MHz, $CDCl_3$)

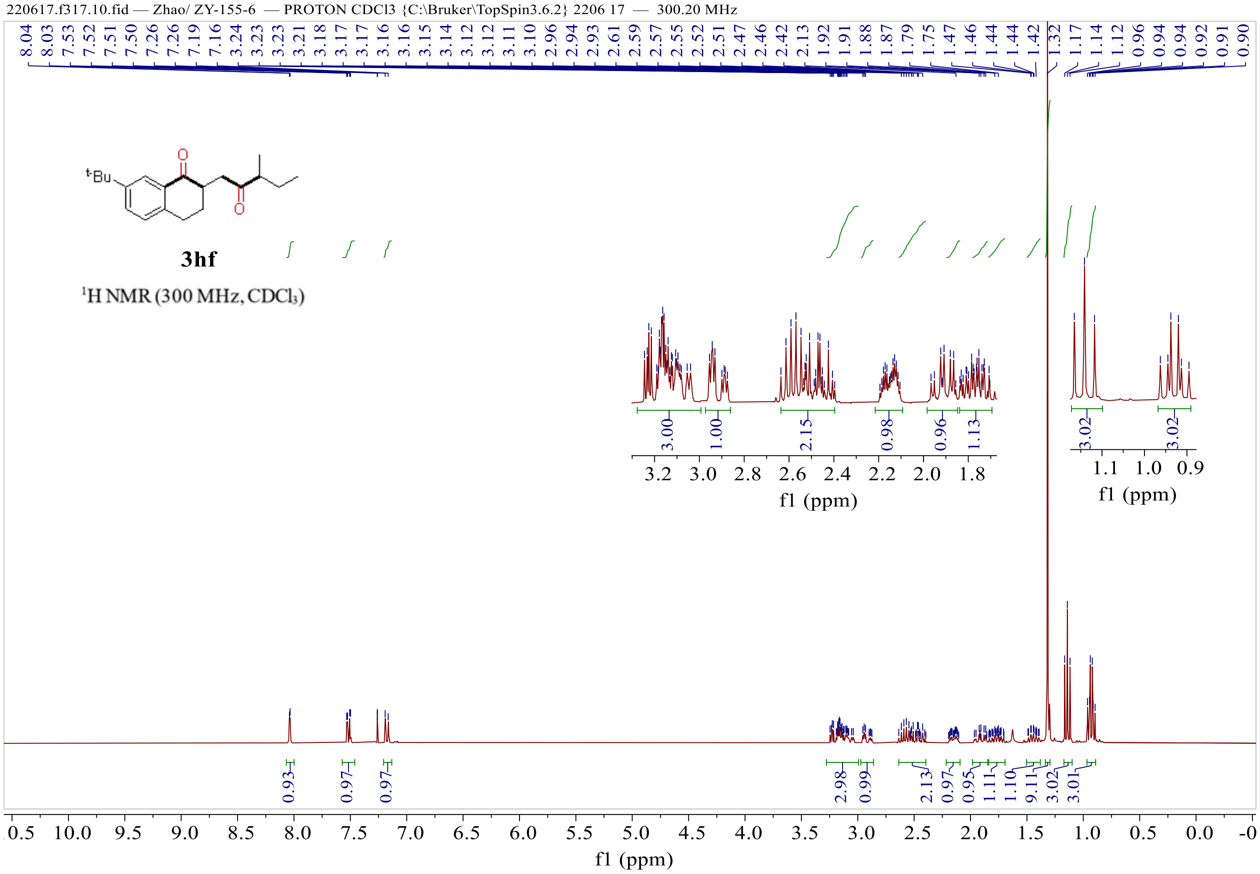
220617.f317.10.fid — Zhao/ ZY-155-6 — PROTON CDCl3 {C:\Bruker\TopSpin3.6.2} 2206 17 — 300.20 MHz
8.04 8.03 7.53 7.52 7.51 7.50 7.26 7.26 7.19 7.16 3.24 3.23 3.23 3.21 3.18 3.17 3.17 3.16 3.16 3.15 3.14 3.12 3.12 3.11 3.10 2.96 2.94 2.93 2.61 2.59 2.57 2.55 2.52 2.51 2.47 2.46 2.42 2.13 1.92 1.91 1.88 1.87 1.79 1.75 1.47 1.46 1.44 1.44 1.42 1.32 1.17 1.14 1.12 0.96 0.94 0.94 0.92 0.91 0.90
t-Bu
3hf
1H NMR (300 MHz, CDCl3)
0.93 0.97 0.97 2.98 0.99 2.13 0.97 0.95 1.11 1.10 9.11 3.02 3.01
3.00 1.00 2.15 0.98 0.96 1.13
3.02 3.02
f1 (ppm)
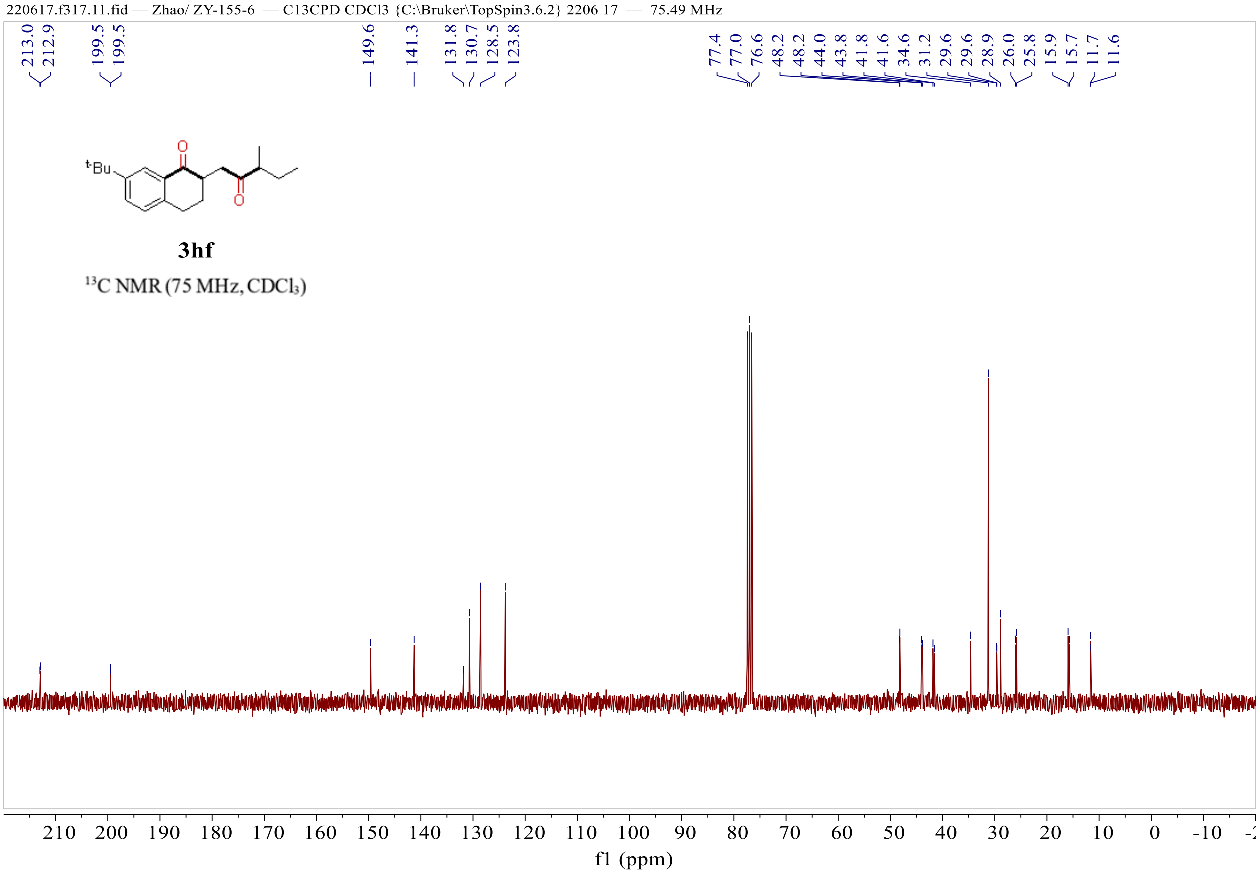
220617.f317.11.fid — Zhao/ ZY-155-6 — C13CPD CDCl3 {C:\Bruker\TopSpin3.6.2} 2206 17 — 75.49 MHz
213.0 212.9 199.5 199.5 149.6 141.3 131.8 130.7 128.5 123.8 77.4 77.0 76.6 48.2 48.2 44.0 43.8 41.8 41.6 34.6 31.2 29.6 29.6 28.9 26.0 25.8 15.9 15.7 11.7 11.6
t-Bu
3hf
13C NMR (75 MHz, CDCl3)
f1 (ppm)

220617.f318.10.fid — Zhao/ ZY-155-7 — PROTON CDCl3 {C:\Bruker\TopSpin3.6.2} 2206 18 — 300.20 MHz

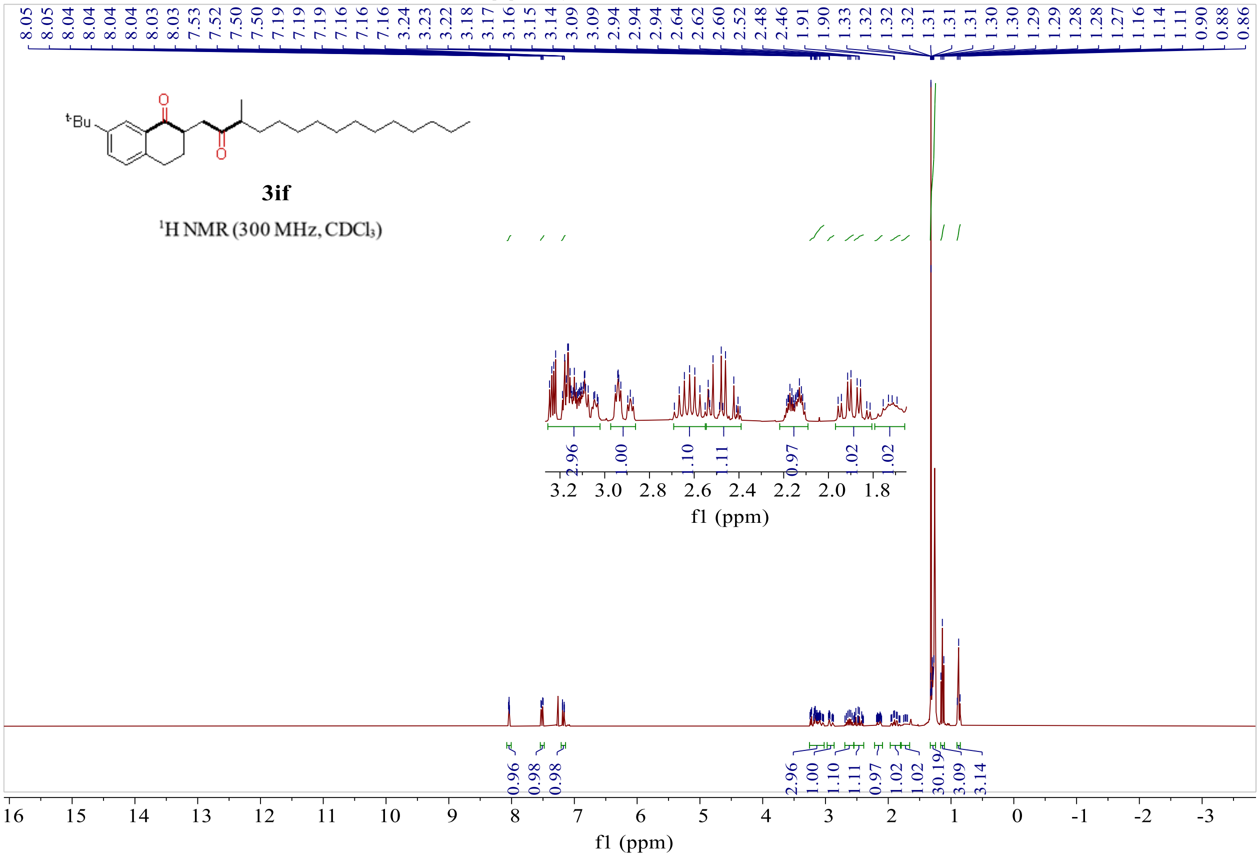

220617.f318.11.fid — Zhao/ ZY-155-7 — C13CPD CDCl3 {C:\Bruker\TopSpin3.6.2} 2206 18 — 75.49 MHz

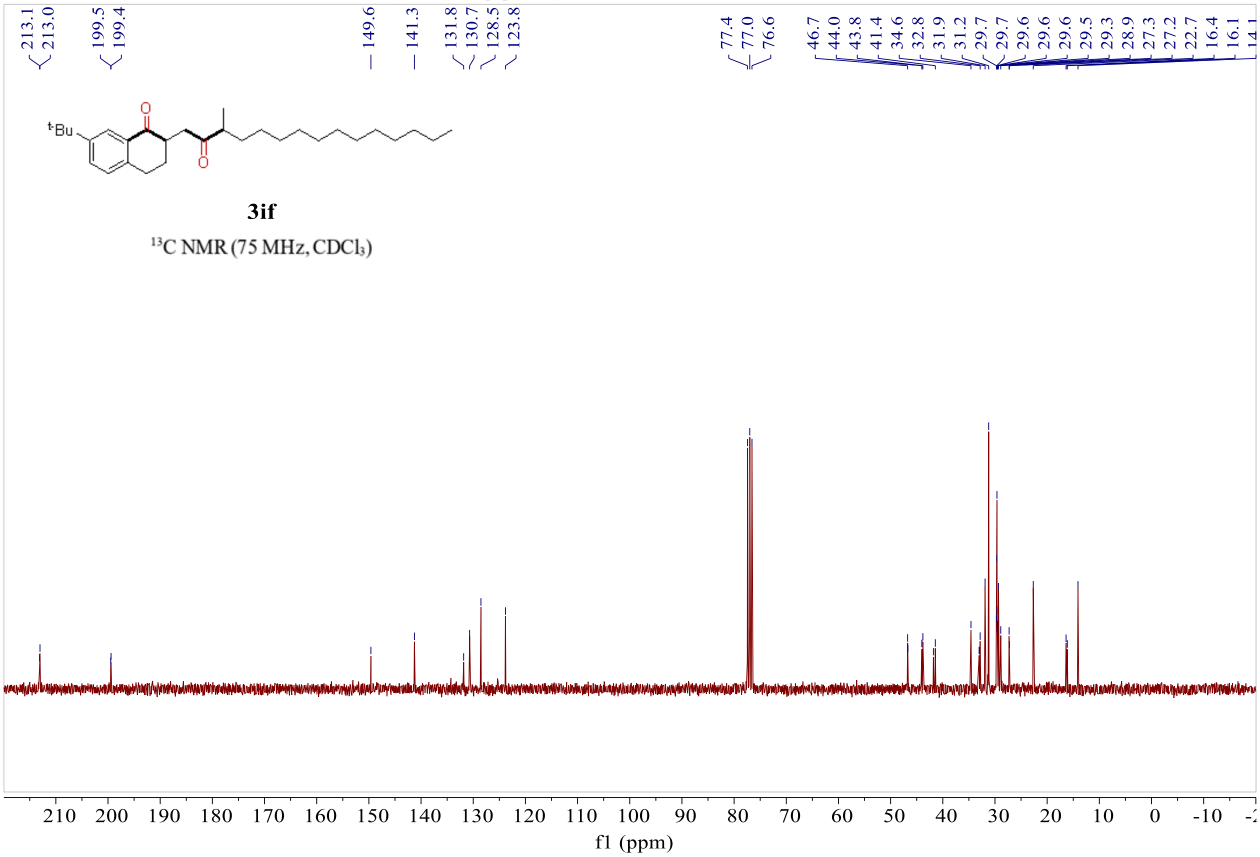

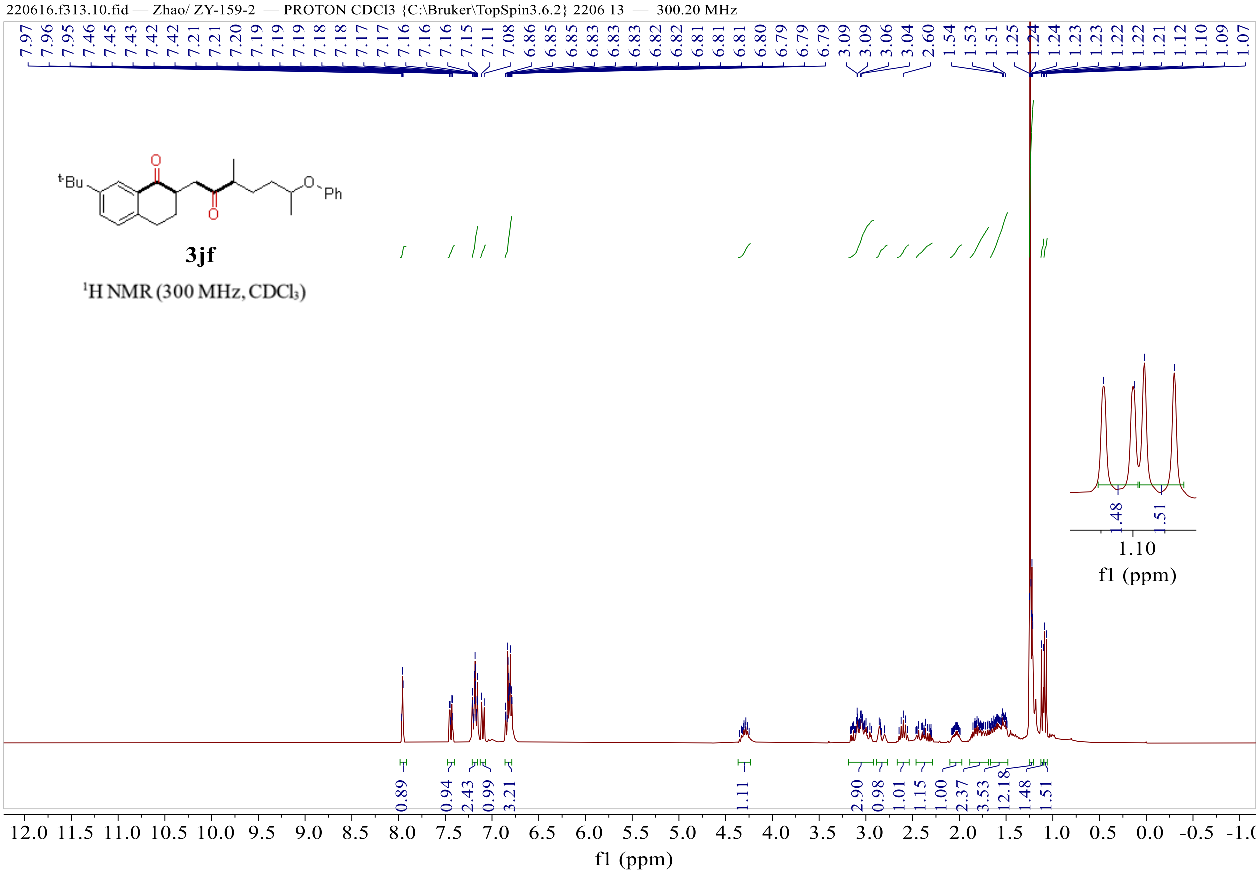
220616.f313.10.fid — Zhao/ ZY-159-2 — PROTON CDCl3 {C:\Bruker\TopSpin3.6.2} 2206 13 — 300.20 MHz
7.97 7.96 7.95 7.46 7.45 7.43 7.42 7.42 7.21 7.21 7.20 7.19 7.19 7.19 7.18 7.18 7.17 7.16 7.16 7.16 7.15 7.11 7.08 6.86 6.85 6.85 6.83 6.83 6.83 6.82 6.81 6.81 6.81 6.80 6.79 6.79 6.79 3.09 3.09 3.06 3.04 2.60 1.54 1.53 1.51 1.25 1.24 1.24 1.23 1.23 1.22 1.22 1.21 1.12 1.10 1.09 1.07
ᵗBu
O
O
O
Ph
3jf
¹H NMR (300 MHz, CDCl₃)
1.48
1.51
1.10
f1 (ppm)
0.89 0.94 2.43 0.99 3.21 1.11 2.90 0.98 1.01 1.15 1.00 2.37 3.53 12.18 1.48 1.51
12.0 11.5 11.0 10.5 10.0 9.5 9.0 8.5 8.0 7.5 7.0 6.5 6.0 5.5 5.0 4.5 4.0 3.5 3.0 2.5 2.0 1.5 1.0 0.5 0.0 -0.5
f1 (ppm)

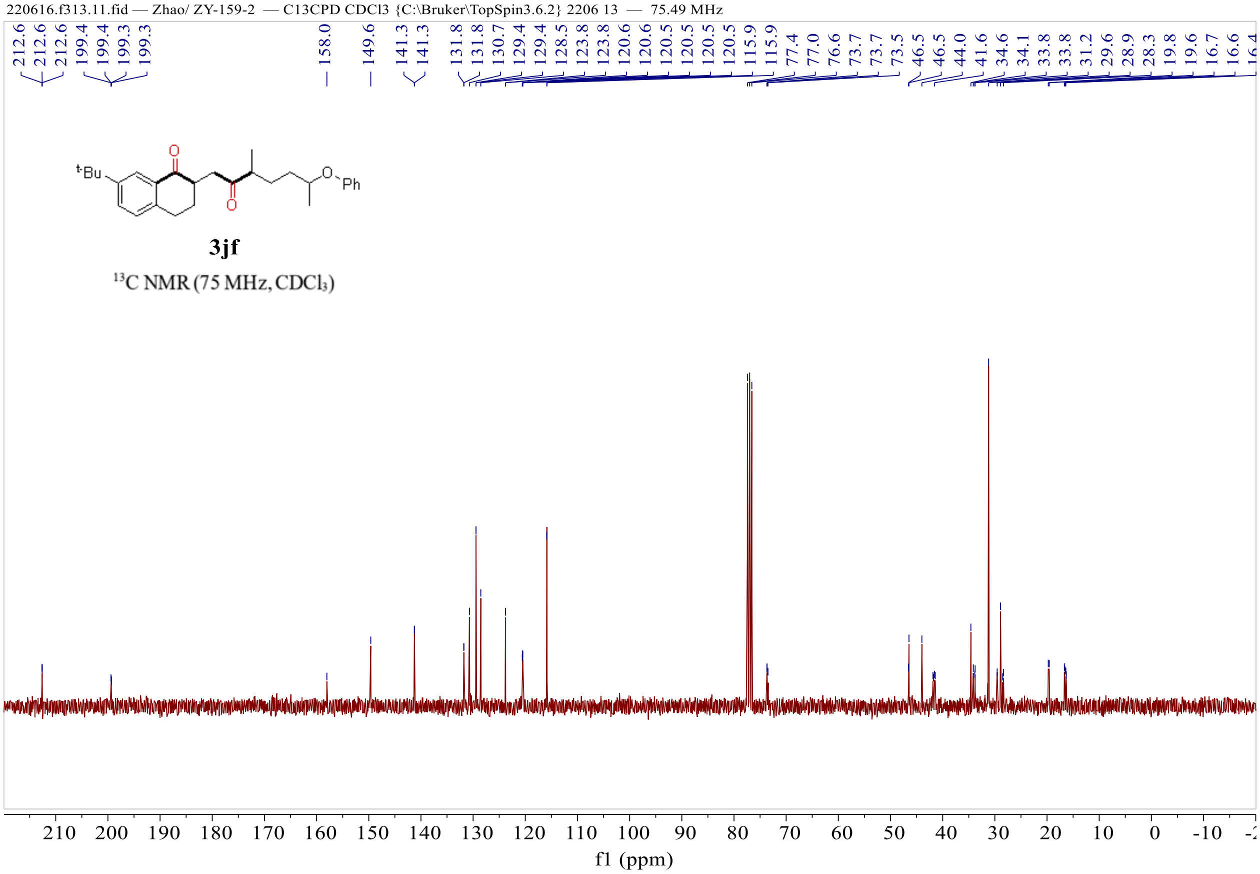
220616.f313.11.fid — Zhao/ ZY-159-2 — C13CPD CDCl3 {C:\Bruker\TopSpin3.6.2} 2206 13 — 75.49 MHz
212.6 212.6 212.6 199.4 199.4 199.3 199.3 158.0 149.6 141.3 141.3 131.8 131.8 130.7 129.4 129.4 128.5 123.8 123.8 120.6 120.6 120.5 120.5 120.5 120.5 115.9 115.9 77.4 77.0 76.6 73.7 73.7 73.5 46.5 46.5 44.0 41.6 34.6 34.1 33.8 33.8 31.2 29.6 28.9 28.3 19.8 19.6 16.7 16.6
ᵗBu
O
O
O
Ph
3jf
¹³C NMR (75 MHz, CDCl₃)
210 200 190 180 170 160 150 140 130 120 110 100 90 80 70 60 50 40 30 20 10 0 -10
f1 (ppm)

220519.f315.10.fid — Fengqian Zhao zy-155-8 — PROTON CDCl3 {C:\Bruker\TopSpin3.6.2} 2205 15 — 300.20 MHz

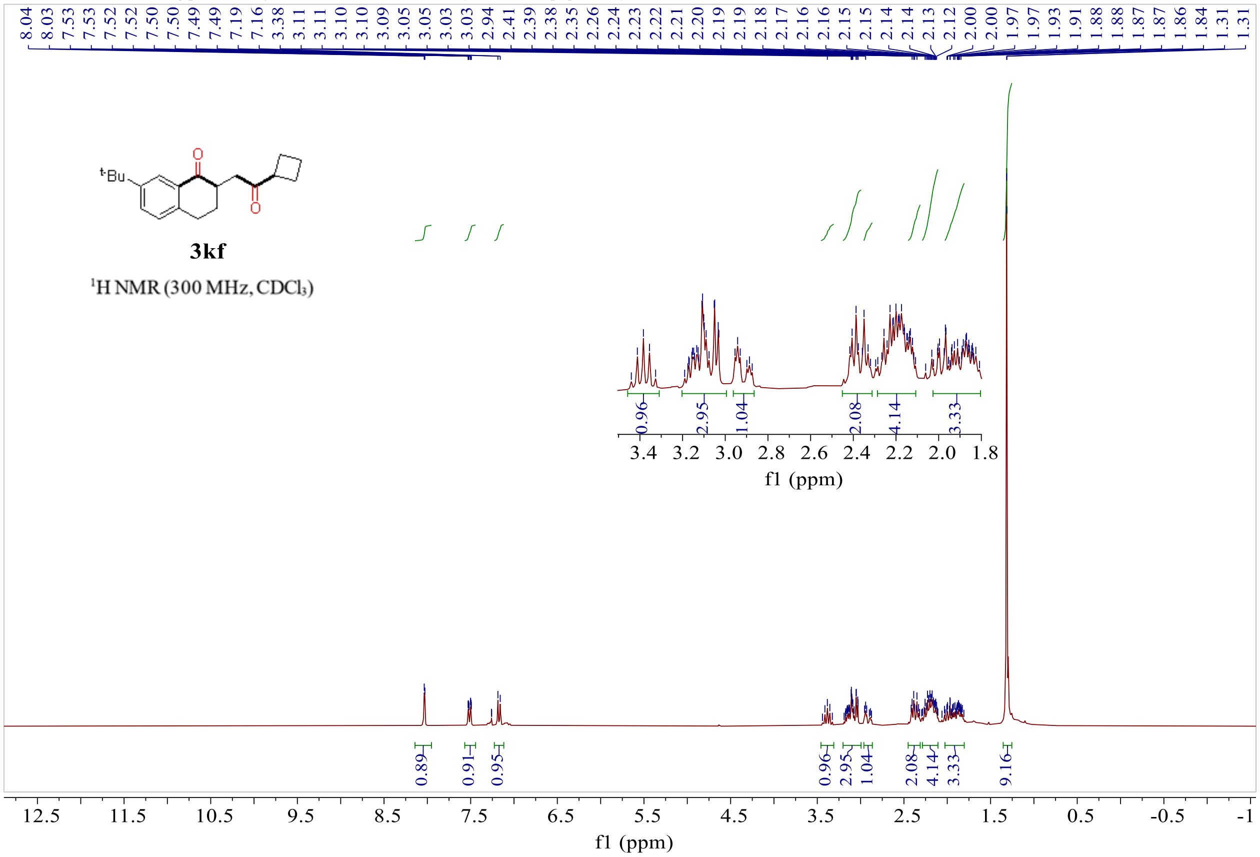

220519.f315.11.fid — Fengqian Zhao zy-155-8 — C13CPD CDCl3 {C:\Bruker\TopSpin3.6.2} 2205 15 — 75.49 MHz

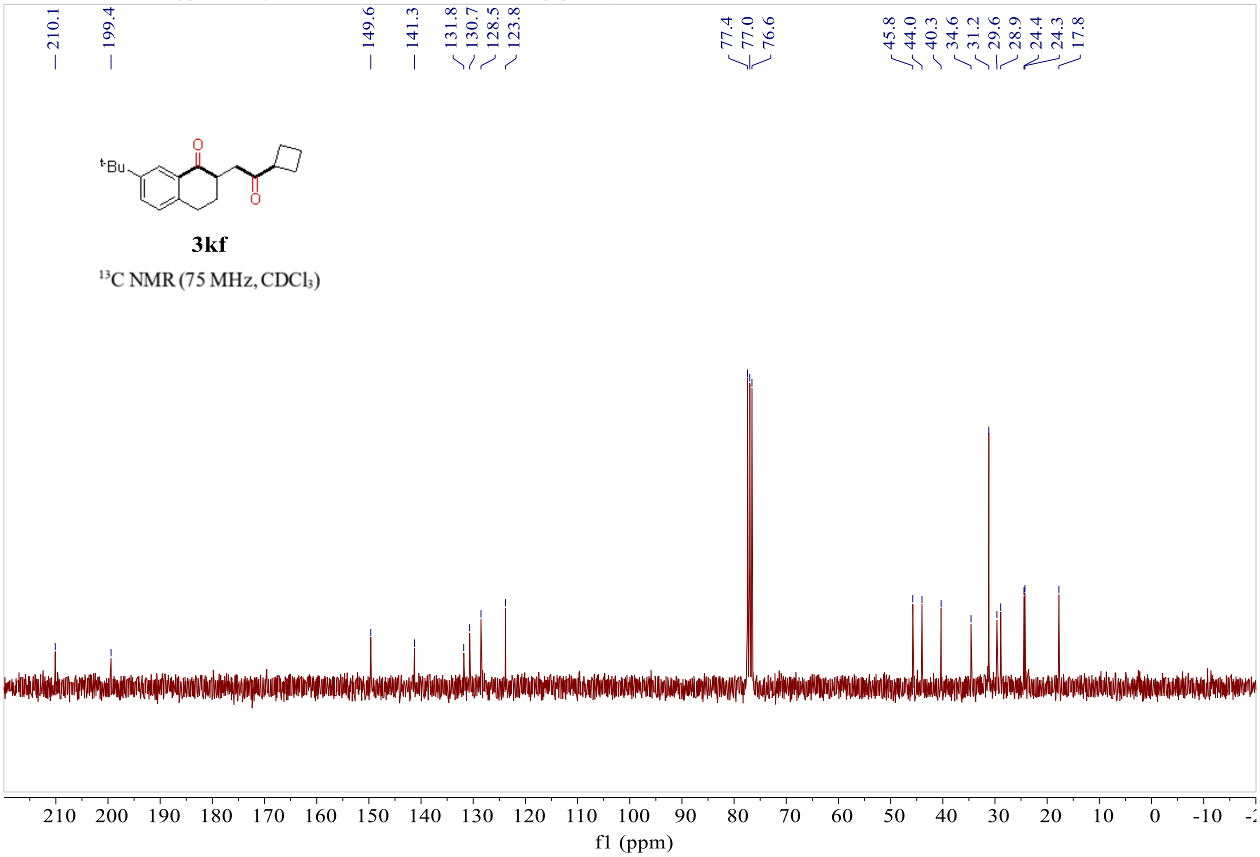

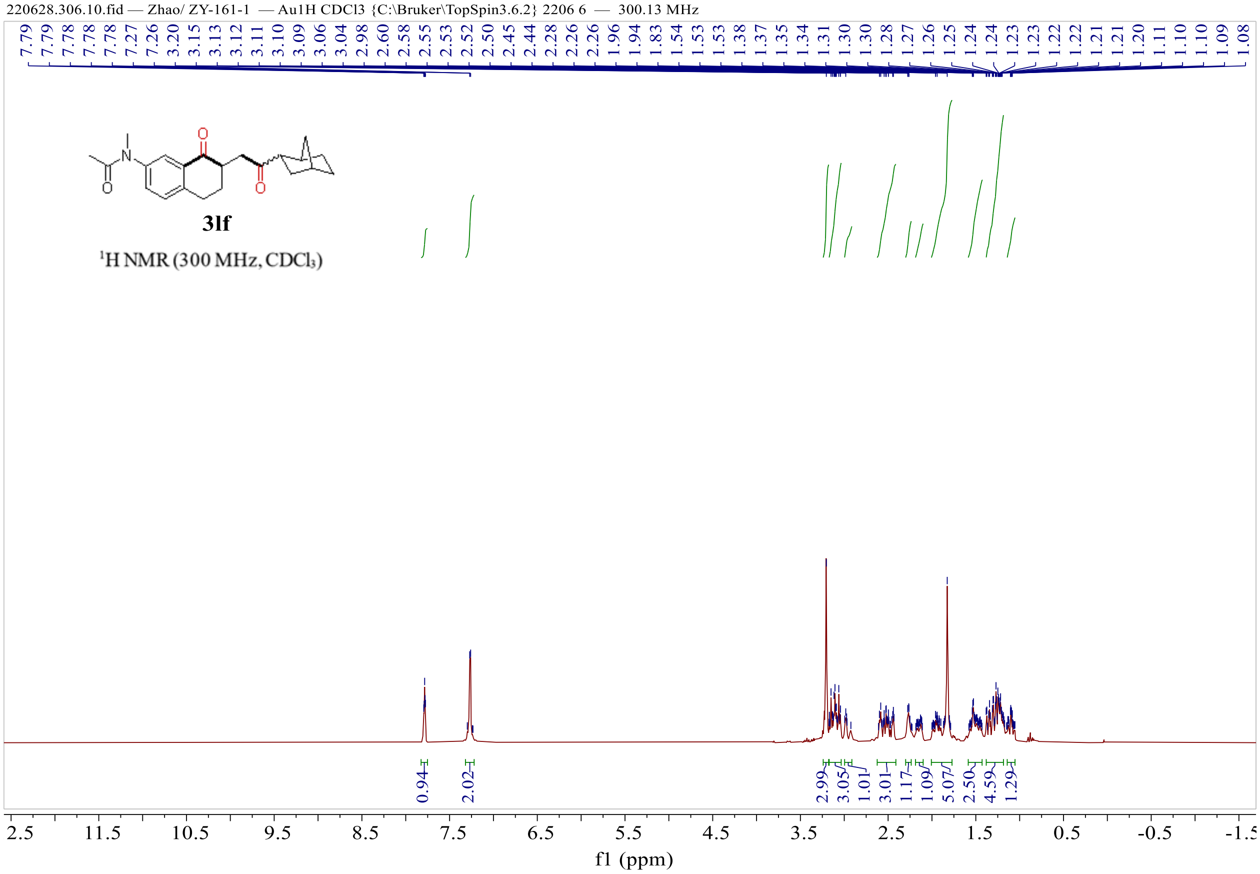
220628.306.10.fid — Zhao/ ZY-161-1 — Au1H CDCl3 {C:\Bruker\TopSpin3.6.2} 2206 6 — 300.13 MHz
7.79 7.79 7.78 7.78 7.78 7.27 7.26 3.20 3.15 3.13 3.12 3.11 3.10 3.09 3.06 3.04 2.98 2.60 2.58 2.55 2.53 2.52 2.50 2.45 2.44 2.28 2.26 2.26 1.96 1.94 1.83 1.54 1.53 1.53 1.38 1.37 1.35 1.34 1.31 1.30 1.30 1.28 1.27 1.26 1.25 1.24 1.24 1.23 1.23 1.22 1.22 1.21 1.21 1.20 1.11 1.10 1.10 1.09 1.08
3lf
1H NMR (300 MHz, CDCl3)
0.94 2.02 2.99 3.05 1.01 3.01 1.17 1.09 5.07 2.50 4.59 1.29
2.5 11.5 10.5 9.5 8.5 7.5 6.5 5.5 4.5 3.5 2.5 1.5 0.5 -0.5 -1.5
f1 (ppm)

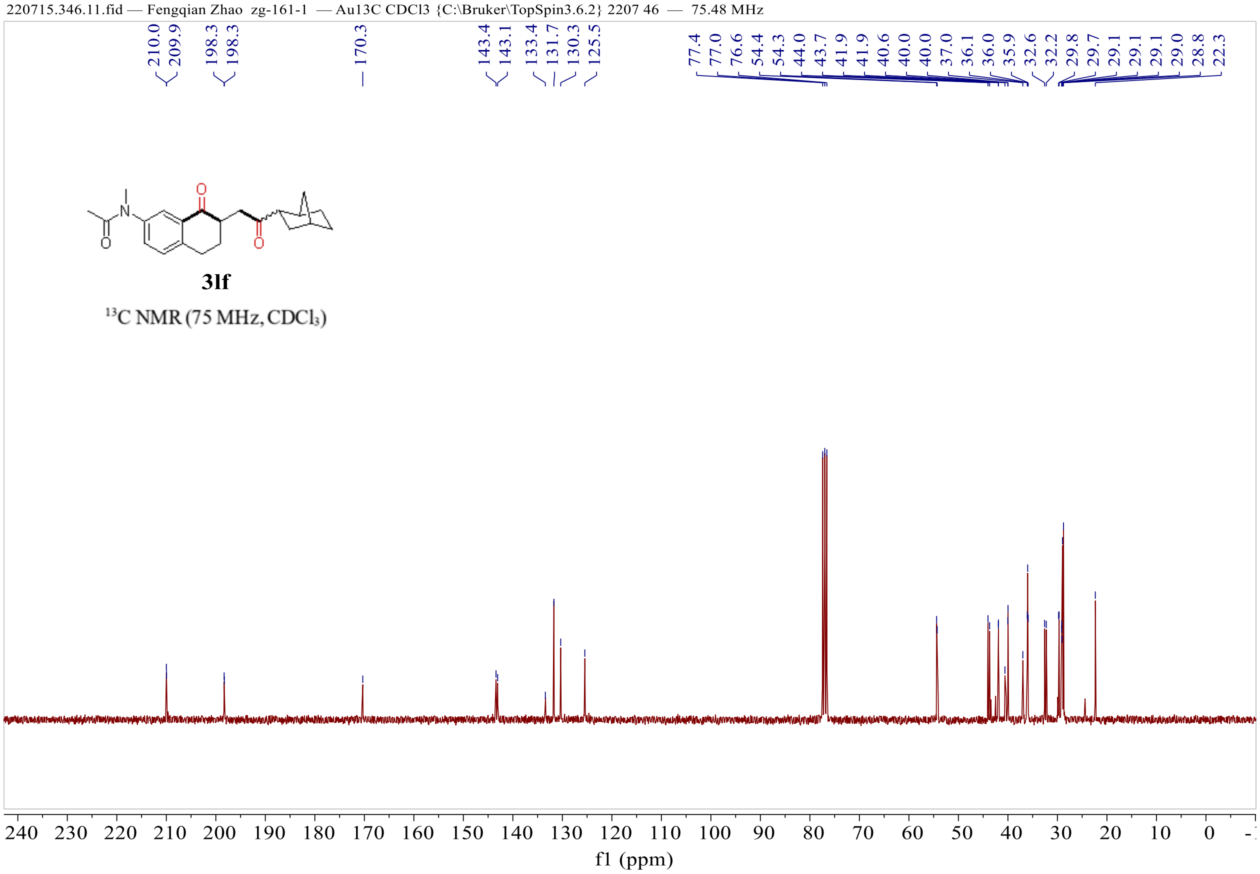
220715.346.11.fid — Fengqian Zhao zg-161-1 — Au13C CDCl3 {C:\Bruker\TopSpin3.6.2} 2207 46 — 75.48 MHz
210.0 209.9 198.3 198.3 170.3 143.4 143.1 133.4 131.7 130.3 125.5 77.4 77.0 76.6 54.4 54.3 44.0 43.7 41.9 41.9 40.6 40.0 40.0 37.0 36.1 36.0 35.9 32.6 32.2 29.8 29.7 29.1 29.1 29.1 29.0 28.8 22.3
3lf
13C NMR (75 MHz, CDCl3)
240 230 220 210 200 190 180 170 160 150 140 130 120 110 100 90 80 70 60 50 40 30 20 10 0
f1 (ppm)

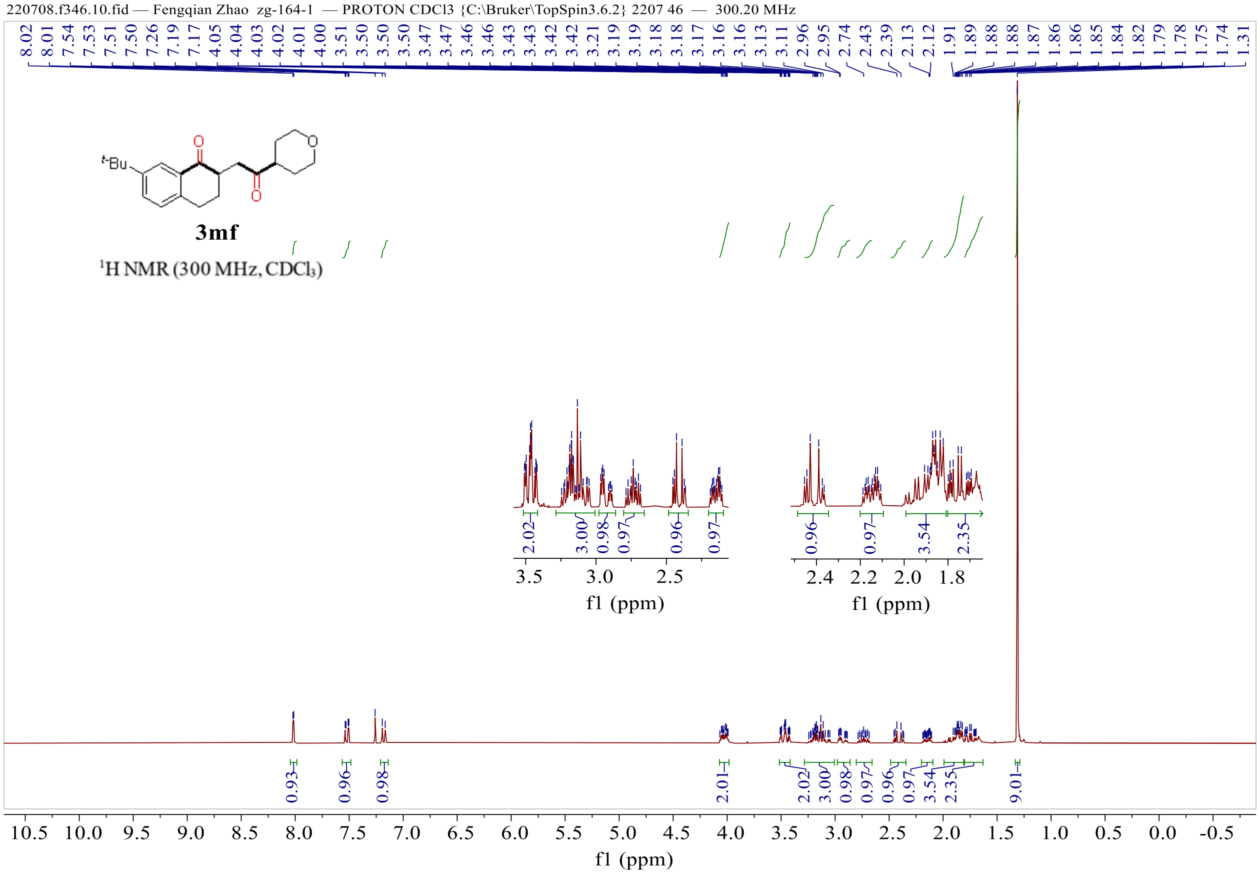
220708.f346.10.fid — Fengqian Zhao zg-164-1 — PROTON CDCl3 {C:\Bruker\TopSpin3.6.2} 2207 46 — 300.20 MHz
t-Bu
O
O
3mf
1H NMR (300 MHz, CDCl3)
f1 (ppm)

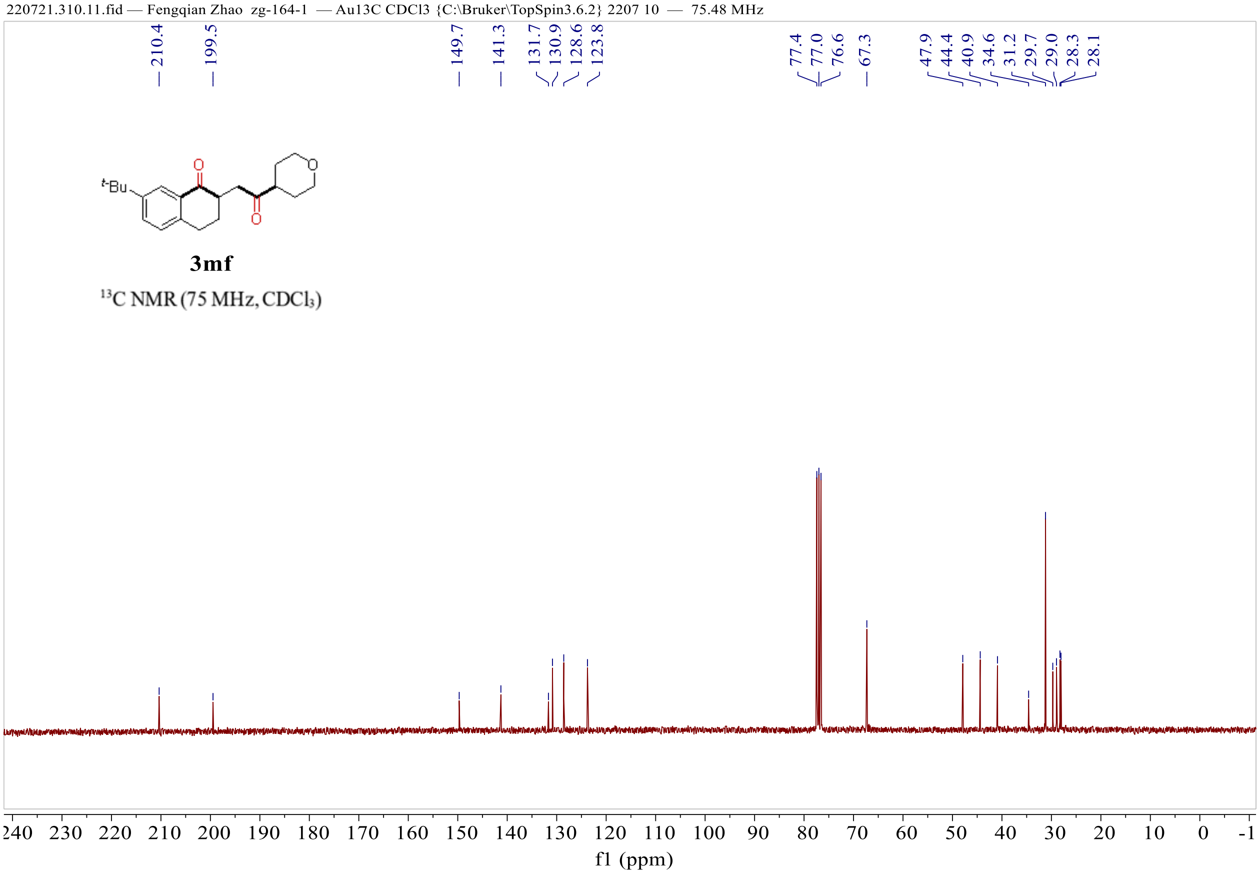
220721.310.11.fid — Fengqian Zhao zg-164-1 — Au13C CDCl3 {C:\Bruker\TopSpin3.6.2} 2207 10 — 75.48 MHz
210.4
199.5
149.7
141.3
131.7
130.9
128.6
123.8
77.4
77.0
76.6
67.3
47.9
44.4
40.9
34.6
31.2
29.7
29.0
28.3
28.1
t-Bu
O
O
3mf
13C NMR (75 MHz, CDCl3)
f1 (ppm)

220708.f347.10.fid — Fengqian Zhao zg-164-2 — PROTON CDCl3 {C:\Bruker\TopSpin3.6.2} 2207 47 — 300.20 MHz

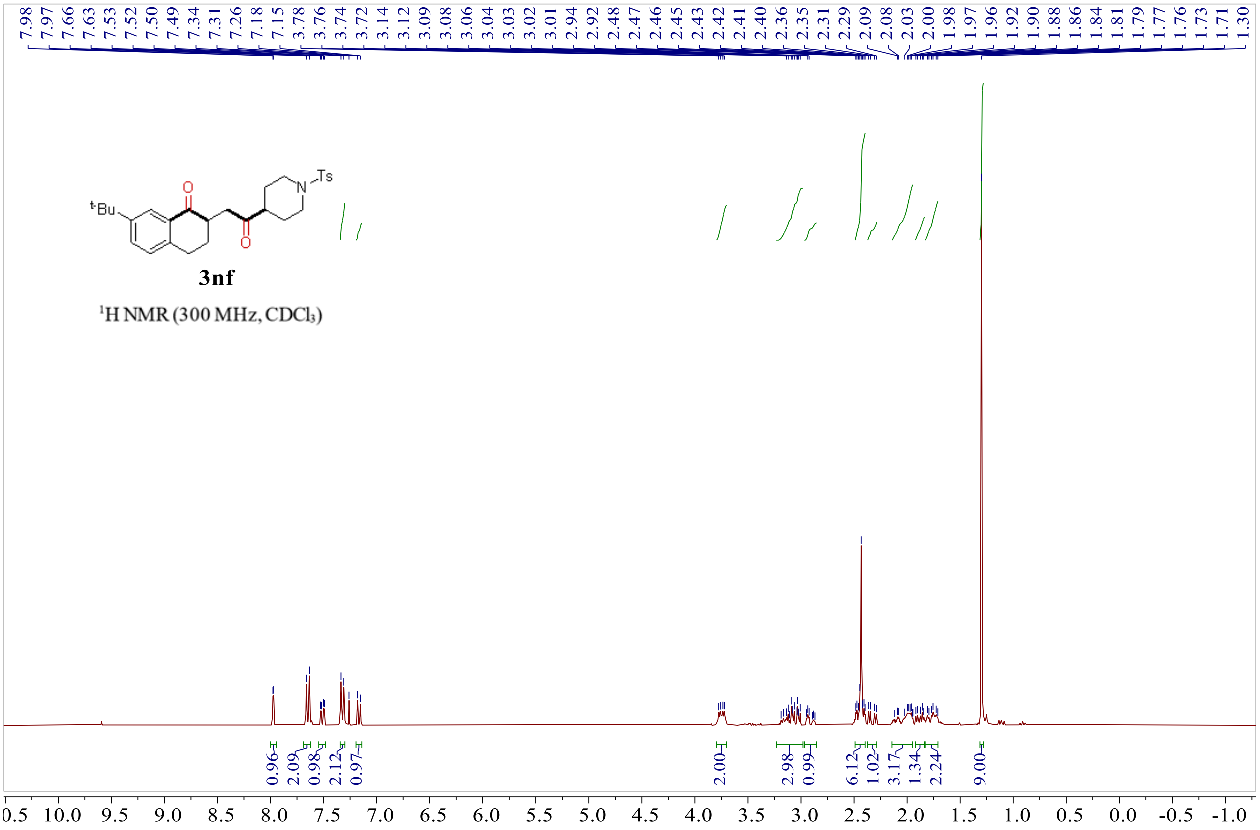

220701.f339.11.fid — Zhao/ ZY-164-2 — C13CPD CDCl3 {C:\Bruker\TopSpin3.6.2} 2207 39 — 75.49 MHz

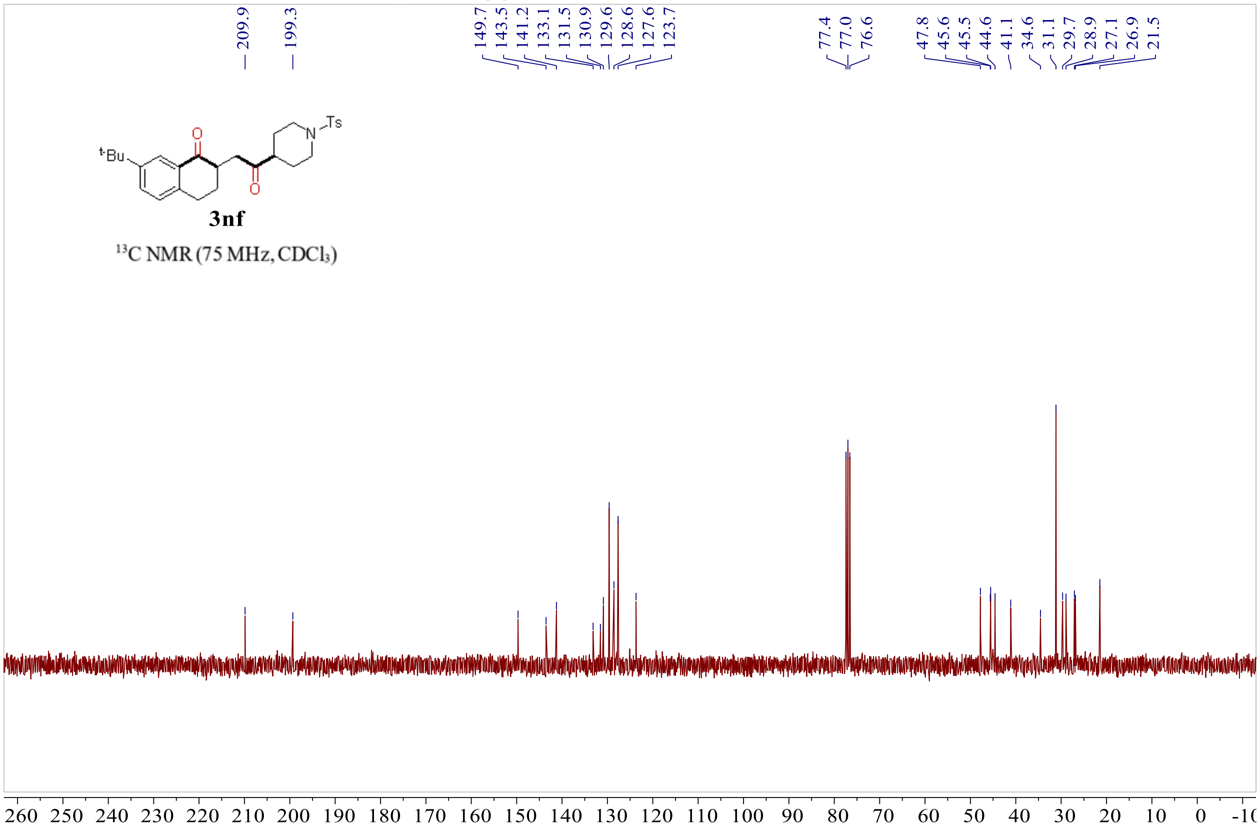

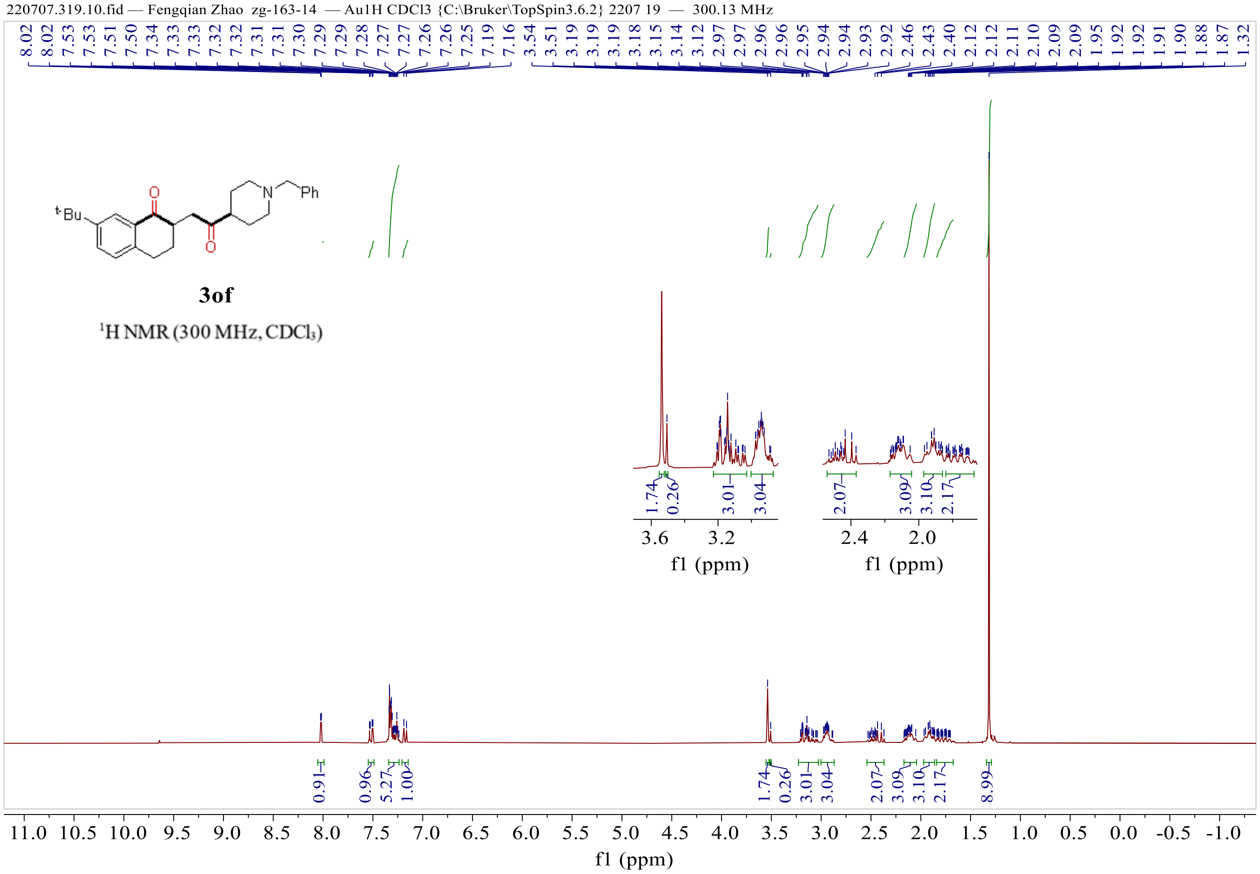
220707.319.10.fid — Fengqian Zhao zg-163-14 — Au1H CDCl3 {C:\Bruker\TopSpin3.6.2} 2207 19 — 300.13 MHz
8.02 8.02 7.53 7.53 7.51 7.50 7.34 7.33 7.33 7.32 7.32 7.31 7.31 7.30 7.29 7.29 7.28 7.27 7.27 7.26 7.26 7.25 7.19 7.16 3.54 3.51 3.19 3.19 3.19 3.18 3.15 3.14 3.12 2.97 2.97 2.96 2.96 2.95 2.94 2.94 2.93 2.92 2.46 2.43 2.40 2.12 2.12 2.11 2.10 2.09 2.09 1.95 1.92 1.92 1.91 1.90 1.88 1.87 1.32
t-Bu
O
N
Ph
3of
1H NMR (300 MHz, CDCl3)
1.74 0.26 3.01 3.04
2.07 3.09 3.10 2.17
f1 (ppm)
0.91 0.96 5.27 1.00 1.74 0.26 3.01 3.04 2.07 3.09 3.10 2.17 8.99
11.0 10.5 10.0 9.5 9.0 8.5 8.0 7.5 7.0 6.5 6.0 5.5 5.0 4.5 4.0 3.5 3.0 2.5 2.0 1.5 1.0 0.5 0.0 -0.5 -1.0
f1 (ppm)

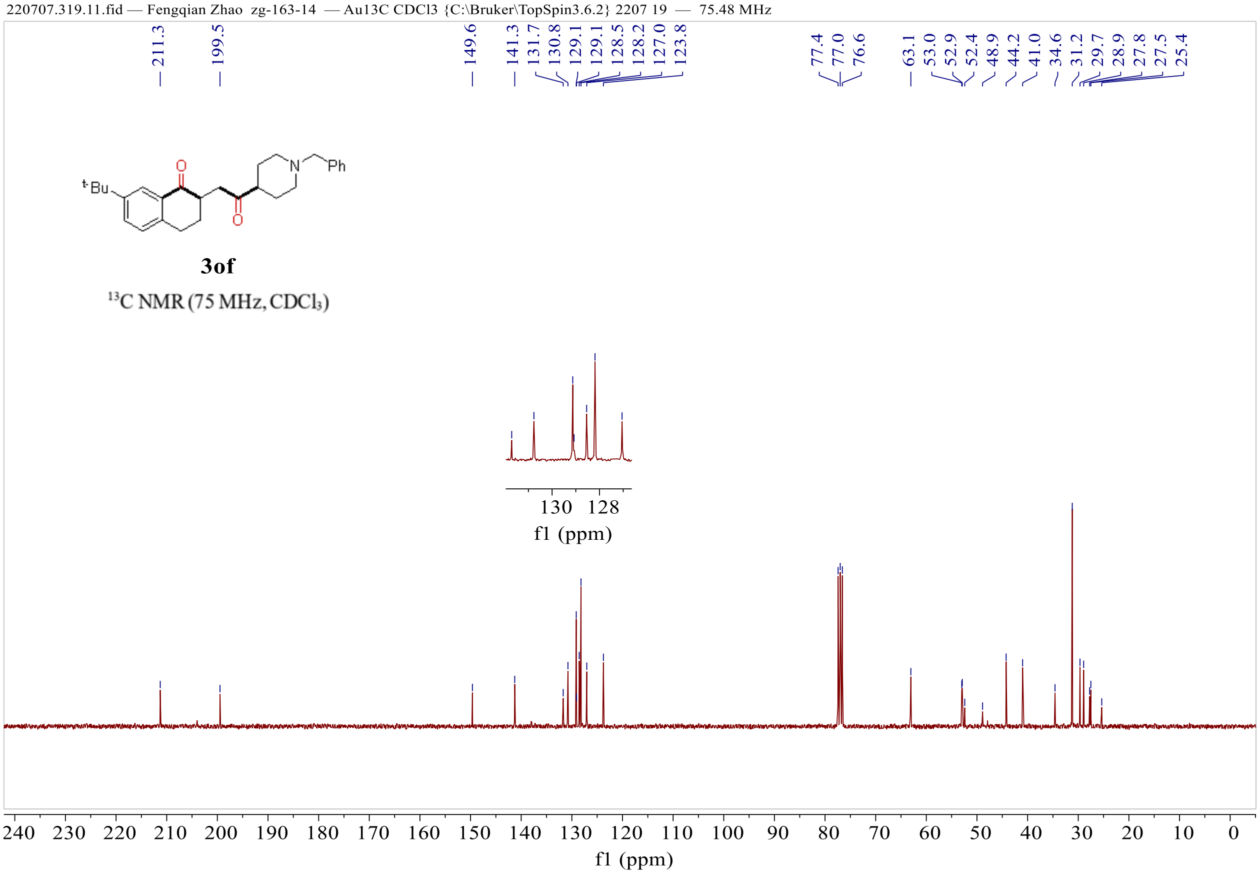
220707.319.11.fid — Fengqian Zhao zg-163-14 — Au13C CDCl3 {C:\Bruker\TopSpin3.6.2} 2207 19 — 75.48 MHz
211.3 199.5 149.6 141.3 131.7 130.8 129.1 129.1 128.5 128.2 127.0 123.8 77.4 77.0 76.6 63.1 53.0 52.9 52.4 48.9 44.2 41.0 34.6 31.2 29.7 28.9 27.8 27.5 25.4
t-Bu
O
N
Ph
3of
13C NMR (75 MHz, CDCl3)
130 128
f1 (ppm)
240 230 220 210 200 190 180 170 160 150 140 130 120 110 100 90 80 70 60 50 40 30 20 10 0
f1 (ppm)

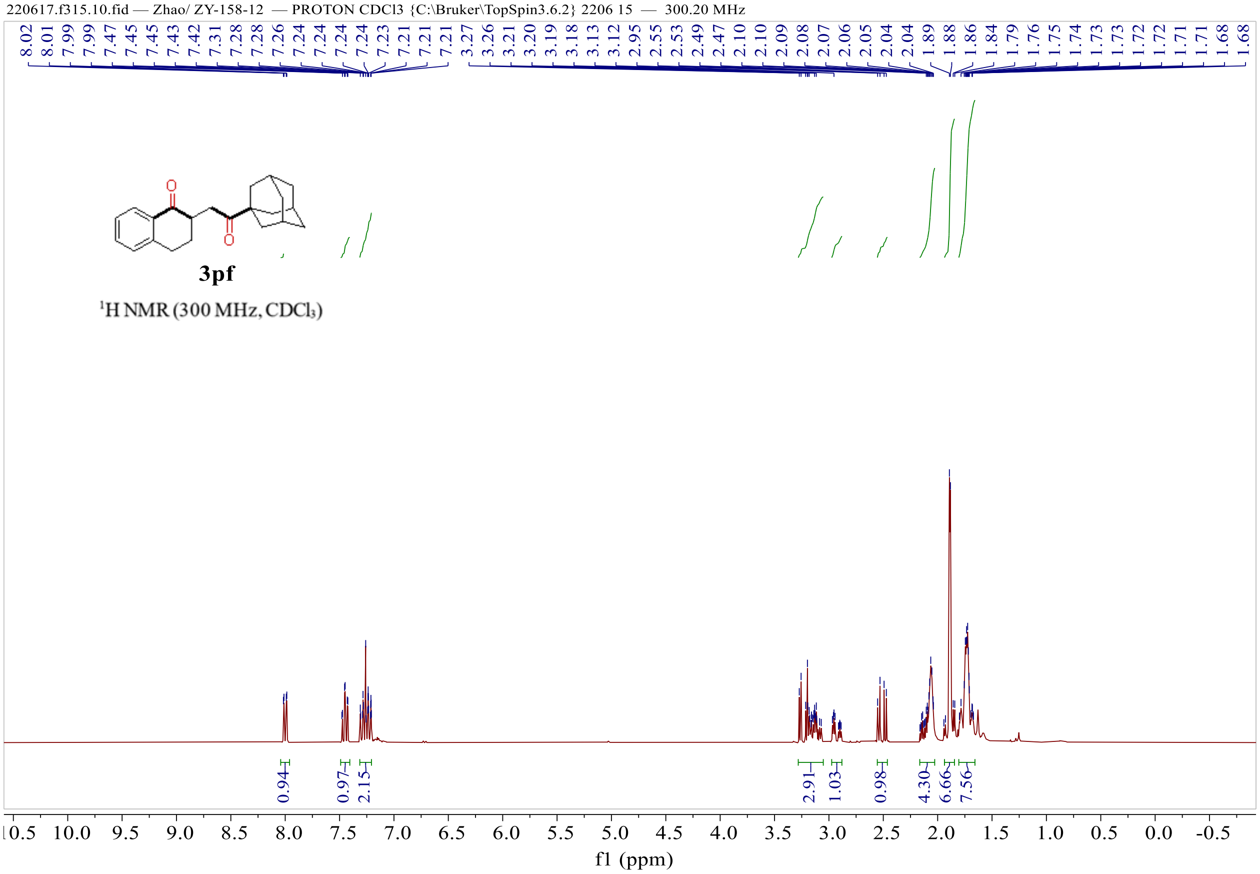
220617.f315.10.fid — Zhao/ ZY-158-12 — PROTON CDCl3 {C:\Bruker\TopSpin3.6.2} 2206 15 — 300.20 MHz
8.02 8.01 7.99 7.99 7.47 7.45 7.45 7.43 7.42 7.31 7.28 7.28 7.26 7.24 7.24 7.24 7.24 7.24 7.23 7.21 7.21 7.21 3.27 3.26 3.21 3.20 3.19 3.18 3.13 3.12 2.95 2.55 2.53 2.49 2.47 2.10 2.10 2.09 2.08 2.07 2.06 2.05 2.04 2.04 1.89 1.88 1.86 1.84 1.79 1.76 1.75 1.74 1.73 1.73 1.72 1.72 1.71 1.71 1.68 1.68
O
O
3pf
1H NMR (300 MHz, CDCl3)
0.94 0.97 2.15 2.91 1.03 0.98 4.30 6.66 7.56
10.5 10.0 9.5 9.0 8.5 8.0 7.5 7.0 6.5 6.0 5.5 5.0 4.5 4.0 3.5 3.0 2.5 2.0 1.5 1.0 0.5 0.0 -0.5
f1 (ppm)

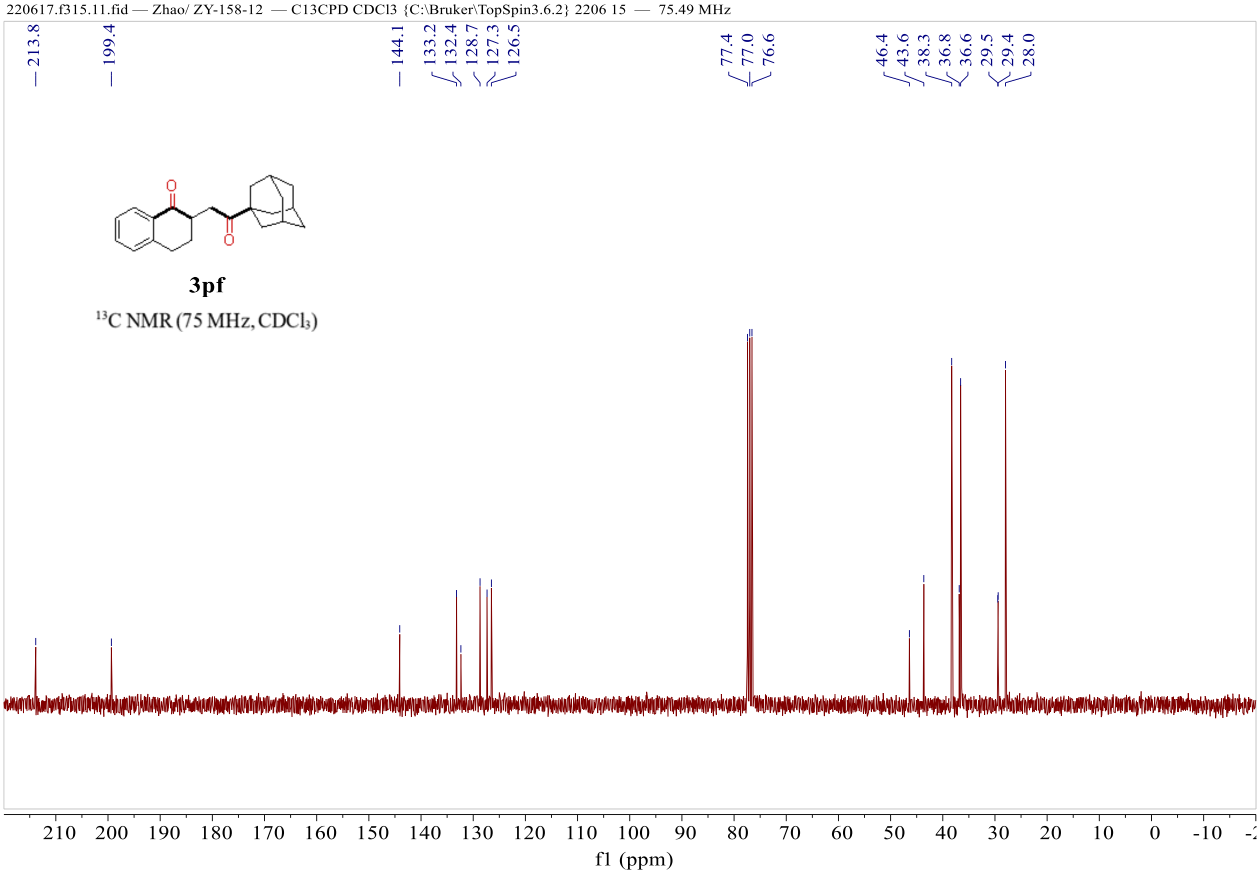
220617.f315.11.fid — Zhao/ ZY-158-12 — C13CPD CDCl3 {C:\Bruker\TopSpin3.6.2} 2206 15 — 75.49 MHz
213.8 199.4 144.1 133.2 132.4 128.7 127.3 126.5 77.4 77.0 76.6 46.4 43.6 38.3 36.8 36.6 29.5 29.4 28.0
O
O
3pf
13C NMR (75 MHz, CDCl3)
210 200 190 180 170 160 150 140 130 120 110 100 90 80 70 60 50 40 30 20 10 0 -10
f1 (ppm)

220517.335.10.fid — Patrizio Russo, 29-154-15 — Au1H CDCl3 {C:\Bruker\TopSpin3.6.2} 2205 35 — 300.13 MHz

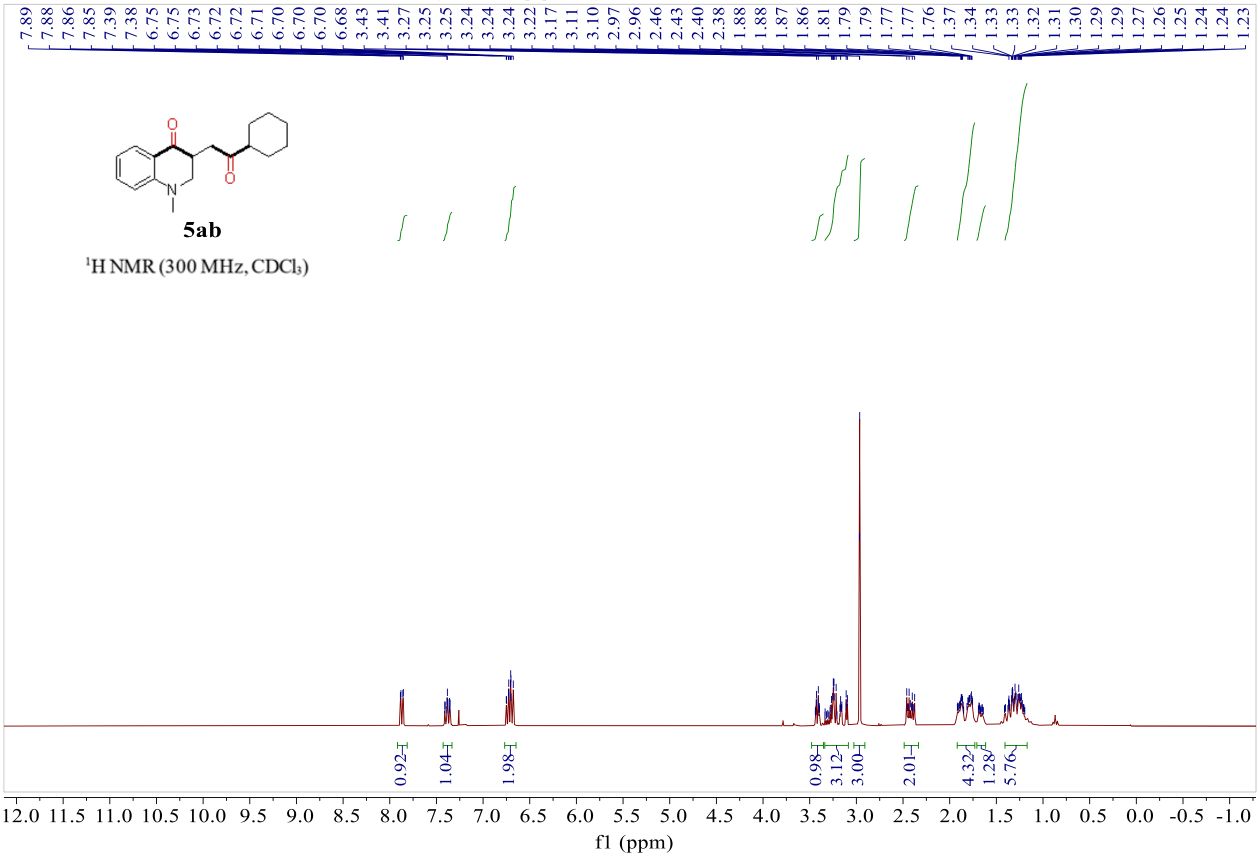

220517.335.11.fid — Patrizio Russo, 29-154-15 — Au13C CDCl3 {C:\Bruker\TopSpin3.6.2} 2205 35 — 75.48 MHz

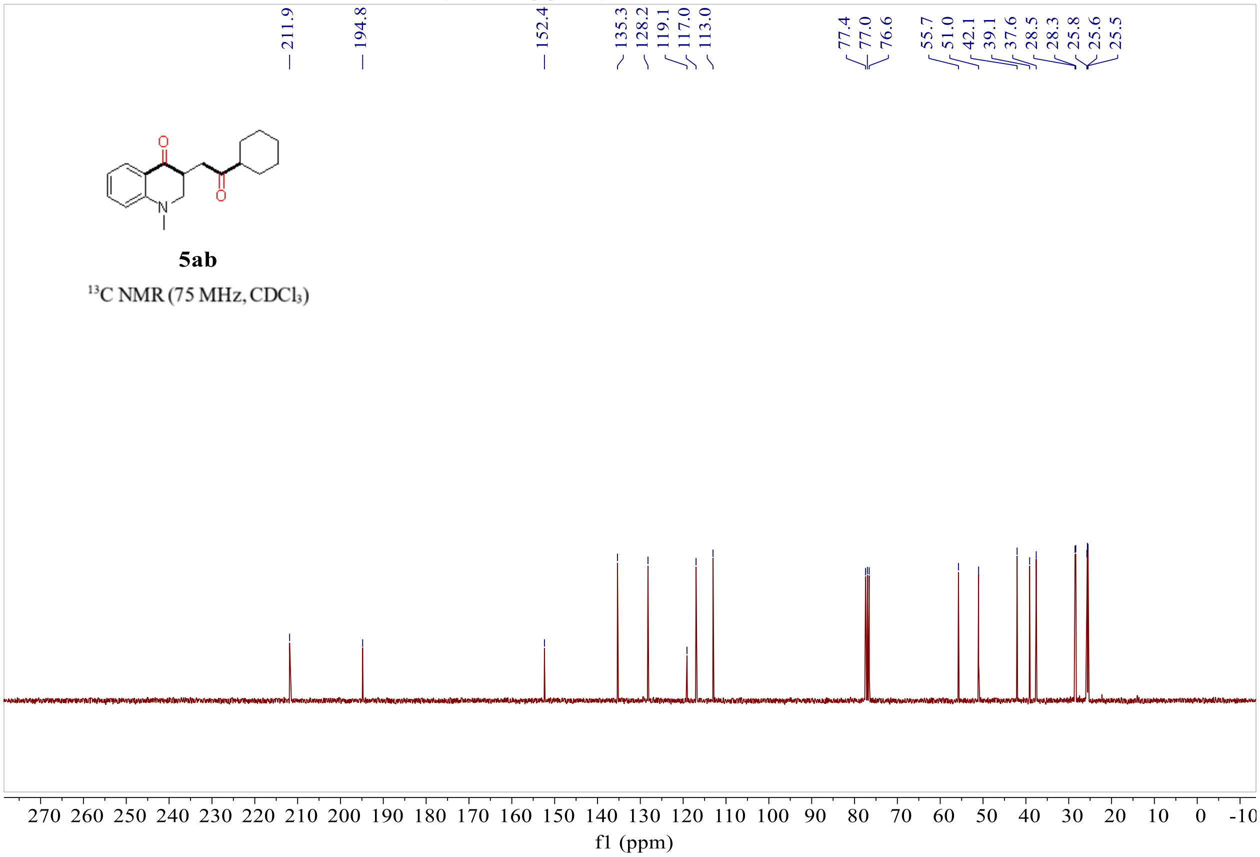

220524.304.10.fid — Fengqian Zhao zg-156-2 — Au1H CDCl3 {C:\Bruker\TopSpin3.6.2} 2205 4 — 300.13 MHz

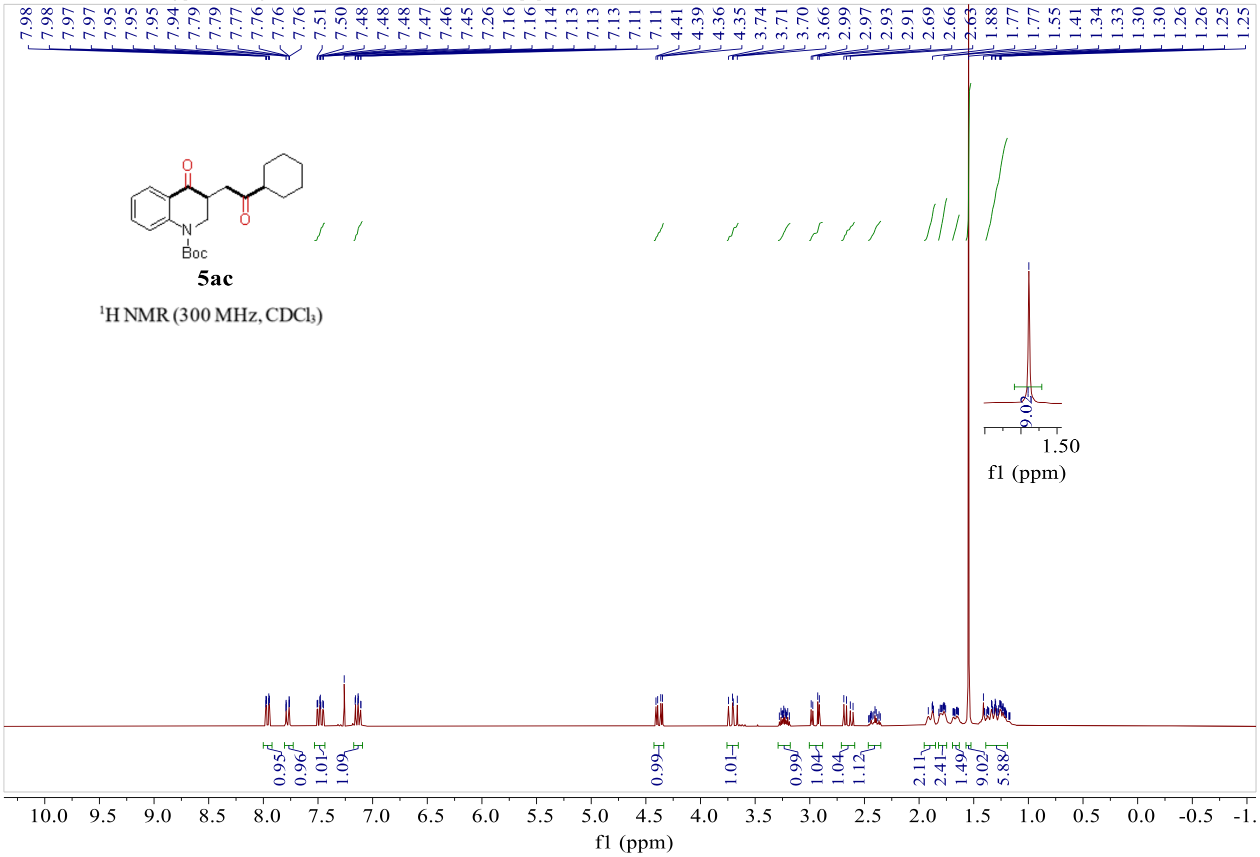

220524.304.11.fid — Fengqian Zhao zg-156-2 — Au13C CDCl3 {C:\Bruker\TopSpin3.6.2} 2205 4 — 75.48 MHz

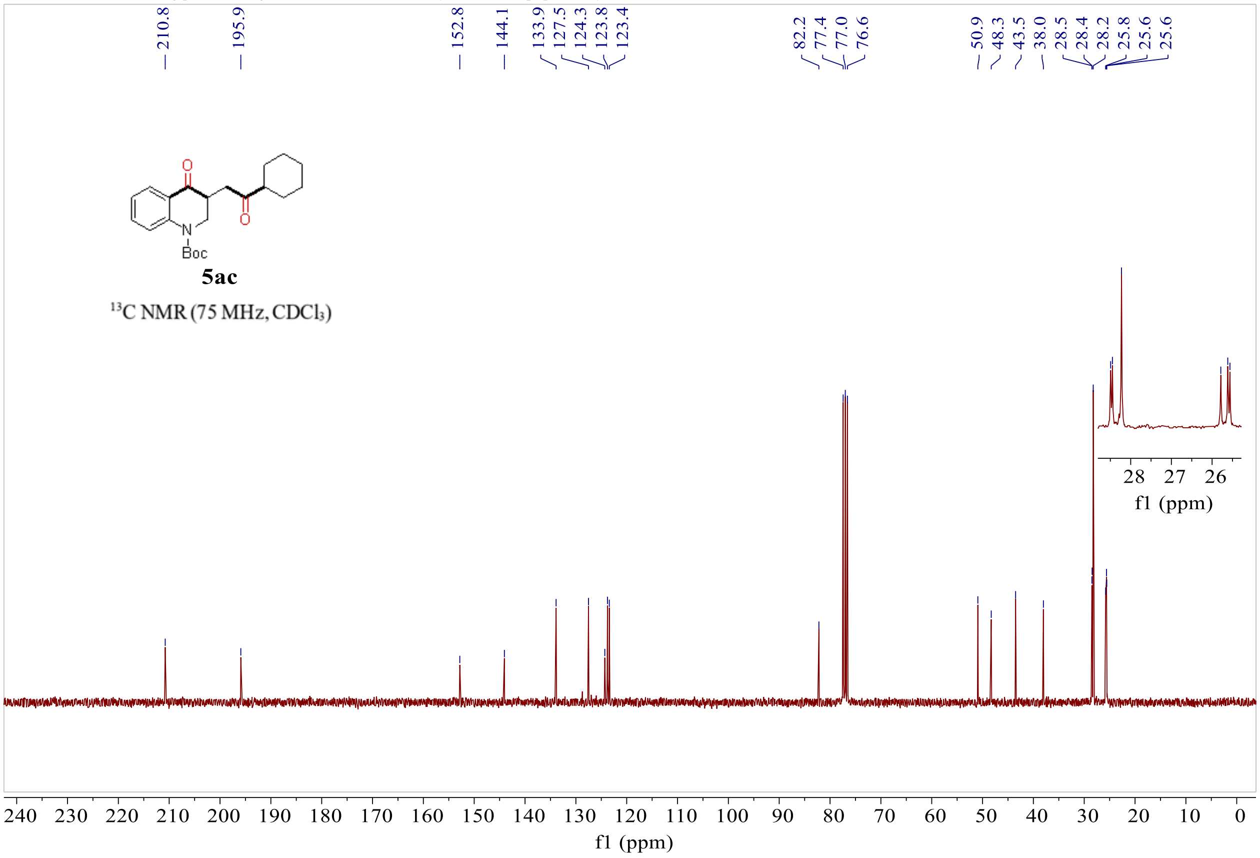

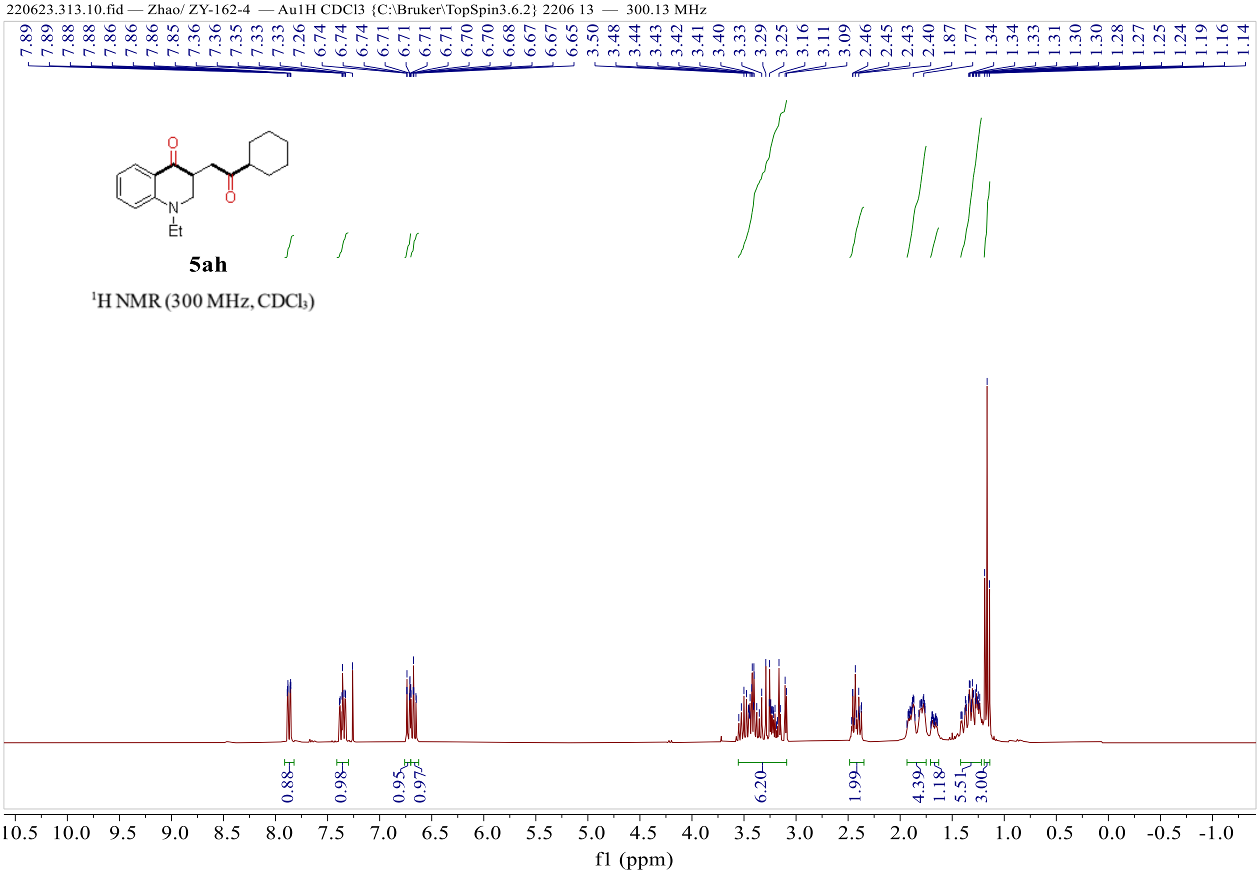
220623.313.10.fid — Zhao/ ZY-162-4 — Au1H CDCl3 {C:\Bruker\TopSpin3.6.2} 2206 13 — 300.13 MHz
5ah
1H NMR (300 MHz, CDCl3)
f1 (ppm)

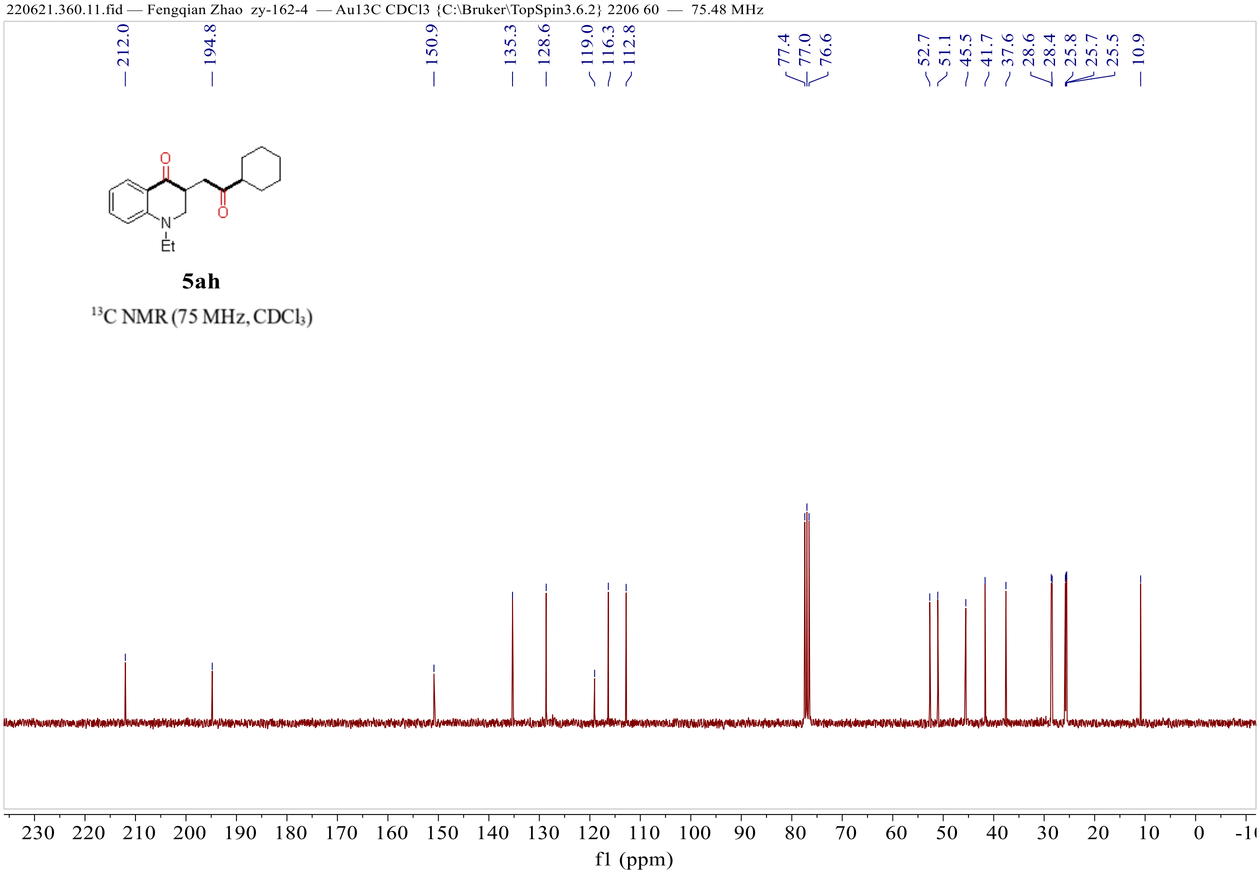
220621.360.11.fid — Fengqian Zhao zy-162-4 — Au13C CDCl3 {C:\Bruker\TopSpin3.6.2} 2206 60 — 75.48 MHz
212.0
194.8
150.9
135.3
128.6
119.0
116.3
112.8
77.4
77.0
76.6
52.7
51.1
45.5
41.7
37.6
28.6
28.4
25.8
25.7
25.5
10.9
5ah
13C NMR (75 MHz, CDCl3)
f1 (ppm)

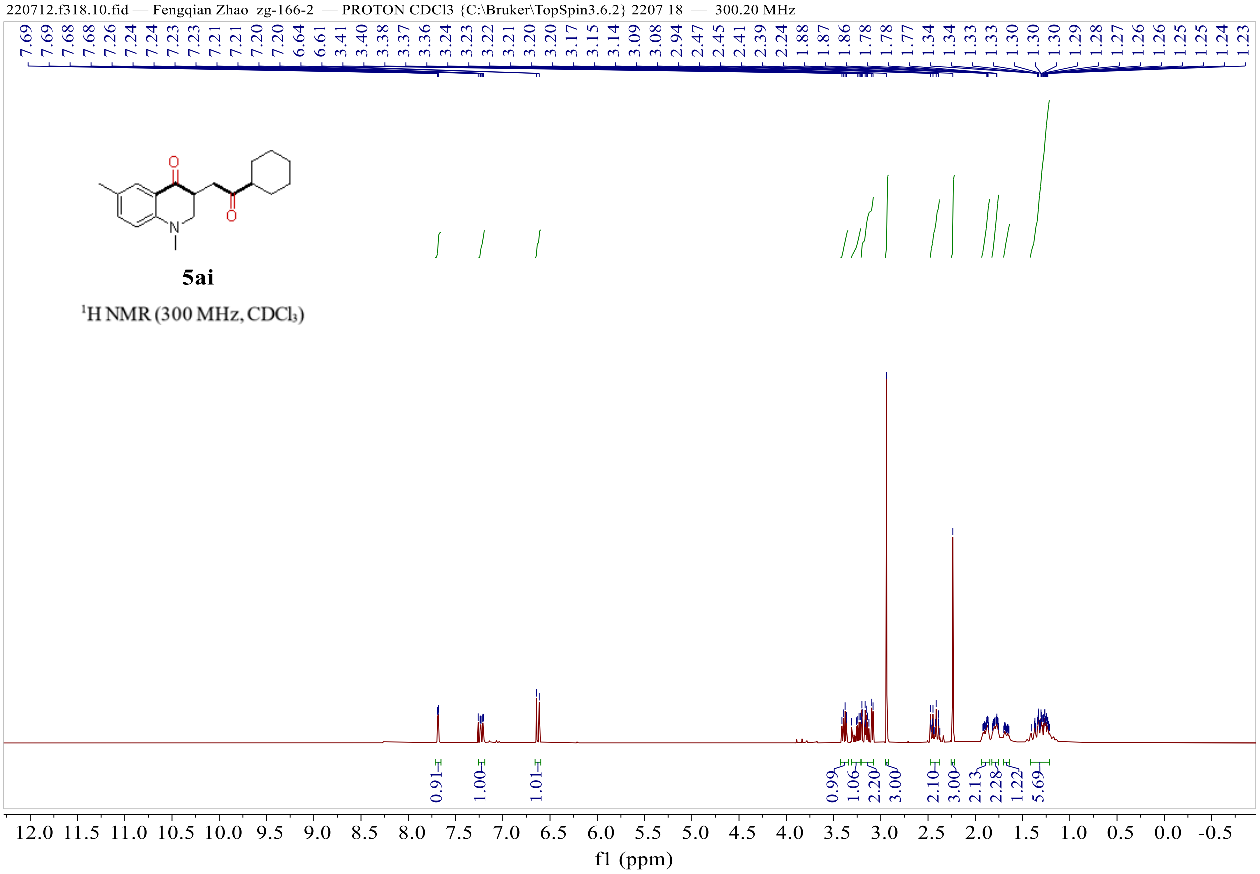
220712.f318.10.fid — Fengqian Zhao zg-166-2 — PROTON CDCl3 {C:\Bruker\TopSpin3.6.2} 2207 18 — 300.20 MHz
7.69 7.69 7.68 7.68 7.26 7.24 7.24 7.23 7.23 7.21 7.21 7.20 7.20 6.64 6.61 3.41 3.40 3.38 3.37 3.36 3.24 3.23 3.22 3.21 3.20 3.20 3.17 3.15 3.14 3.09 3.08 2.94 2.47 2.45 2.41 2.39 2.24 1.88 1.87 1.86 1.78 1.78 1.77 1.34 1.34 1.33 1.33 1.30 1.30 1.30 1.29 1.28 1.27 1.26 1.26 1.25 1.25 1.24 1.23
5ai
^{1}H NMR (300 MHz, $CDCl_3$)
0.91 1.00 1.01 0.99 1.06 2.20 3.00 2.10 3.00 2.13 2.28 1.22 5.69
12.0 11.5 11.0 10.5 10.0 9.5 9.0 8.5 8.0 7.5 7.0 6.5 6.0 5.5 5.0 4.5 4.0 3.5 3.0 2.5 2.0 1.5 1.0 0.5 0.0 -0.5
f1 (ppm)

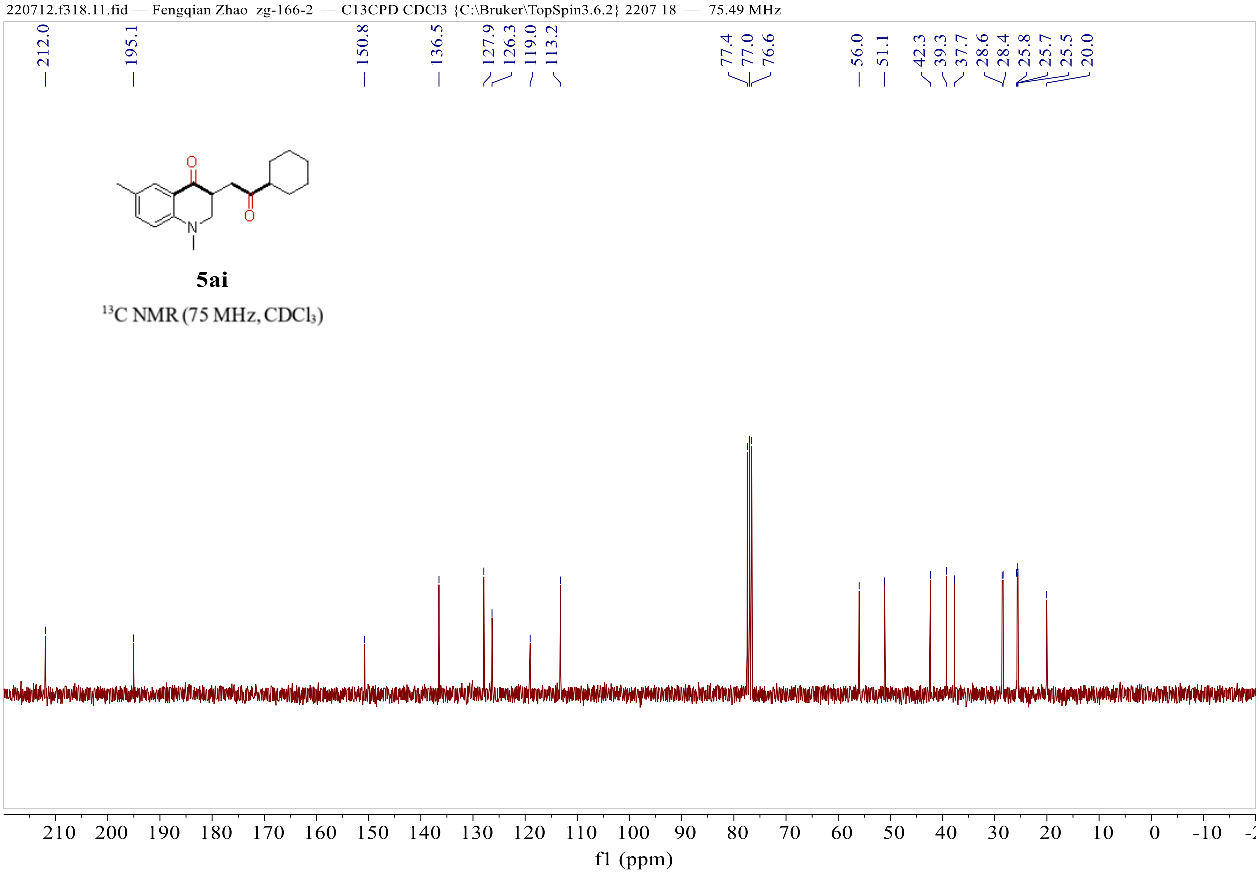
220712.f318.11.fid — Fengqian Zhao zg-166-2 — C13CPD CDCl3 {C:\Bruker\TopSpin3.6.2} 2207 18 — 75.49 MHz
212.0 195.1 150.8 136.5 127.9 126.3 119.0 113.2 77.4 77.0 76.6 56.0 51.1 42.3 39.3 37.7 28.6 28.4 25.8 25.7 25.5 20.0
5ai
^{13}C NMR (75 MHz, $CDCl_3$)
210 200 190 180 170 160 150 140 130 120 110 100 90 80 70 60 50 40 30 20 10 0 -10
f1 (ppm)

220715.341.10.fid — Fengqian Zhao zg-161-4 — Au1H CDCl3 {C:\Bruker\TopSpin3.6.2} 2207 41 — 300.13 MHz

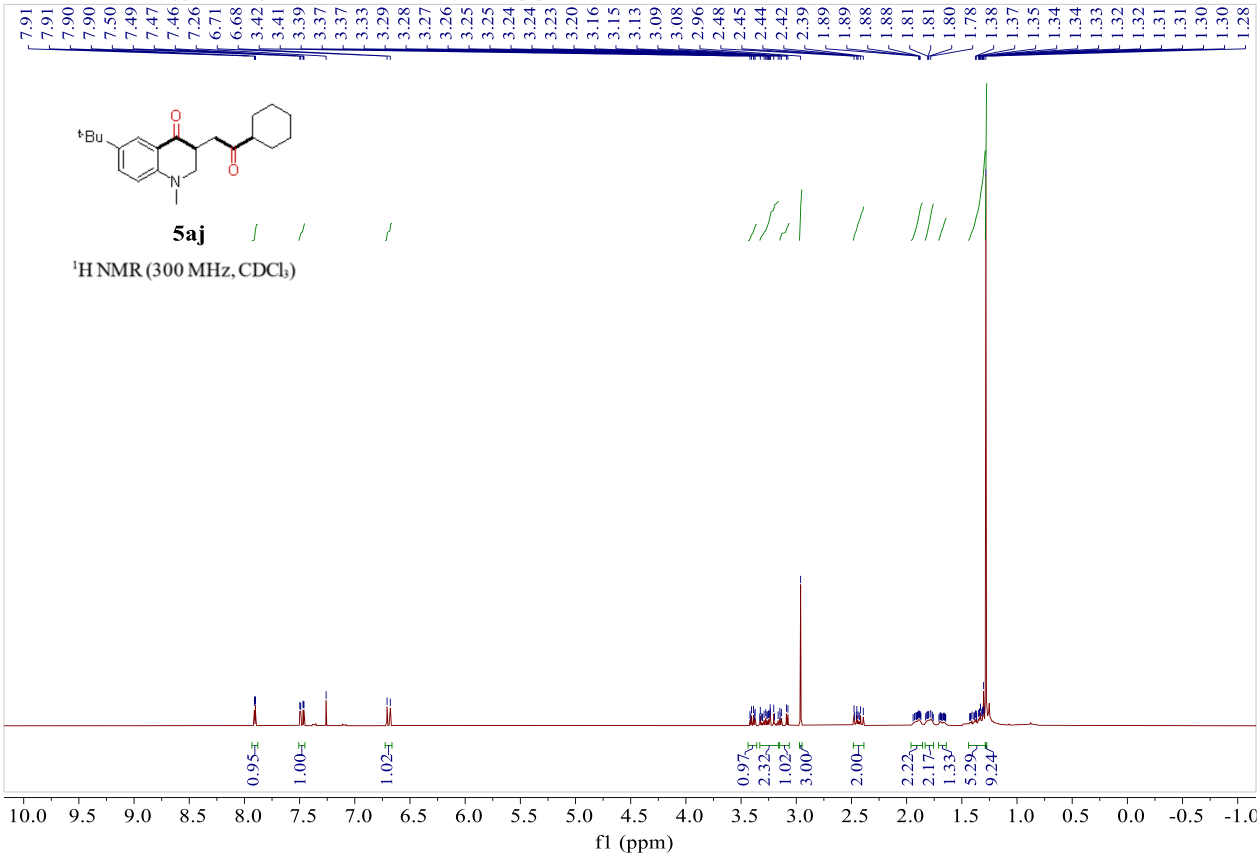

220628.308.11.fid — Zhao/ ZY-161-4 — Au13C CDCl3 {C:\Bruker\TopSpin3.6.2} 2206 8 — 75.48 MHz

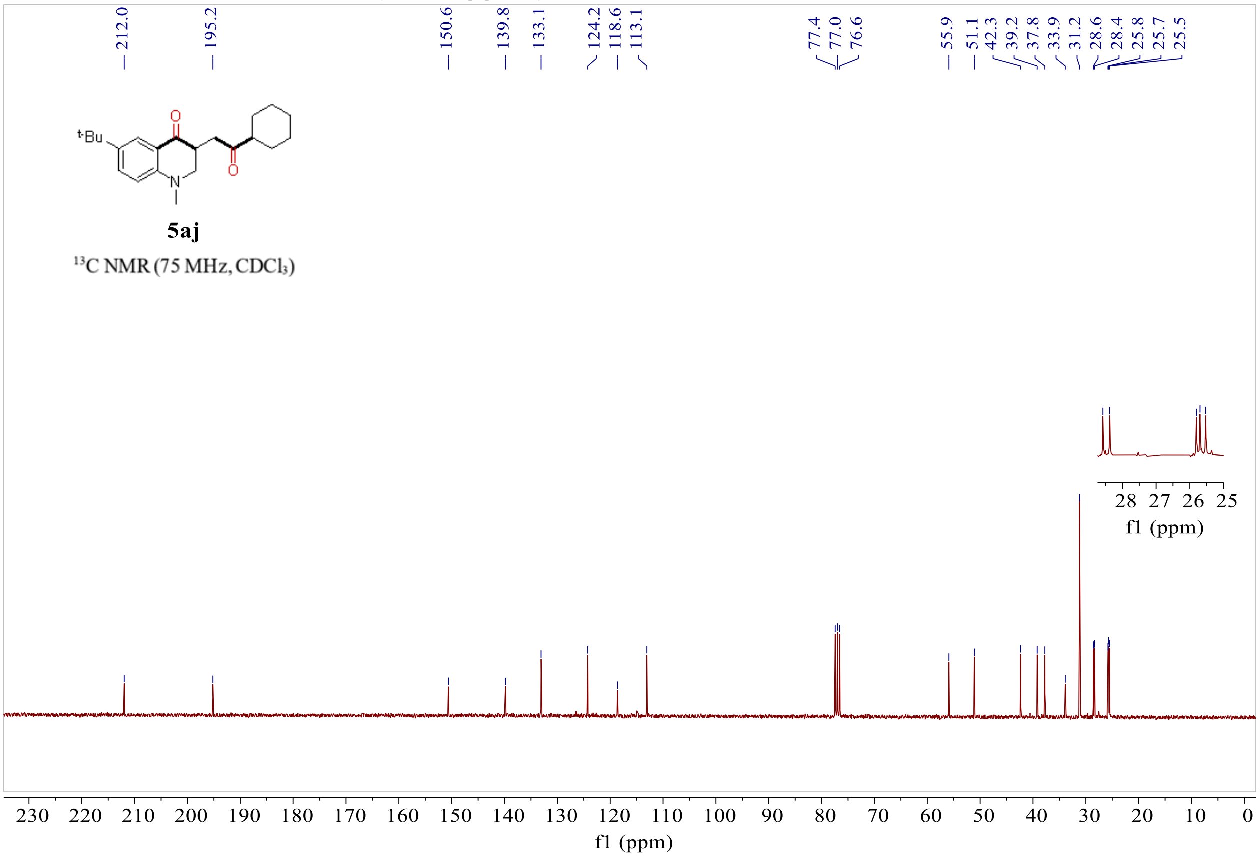

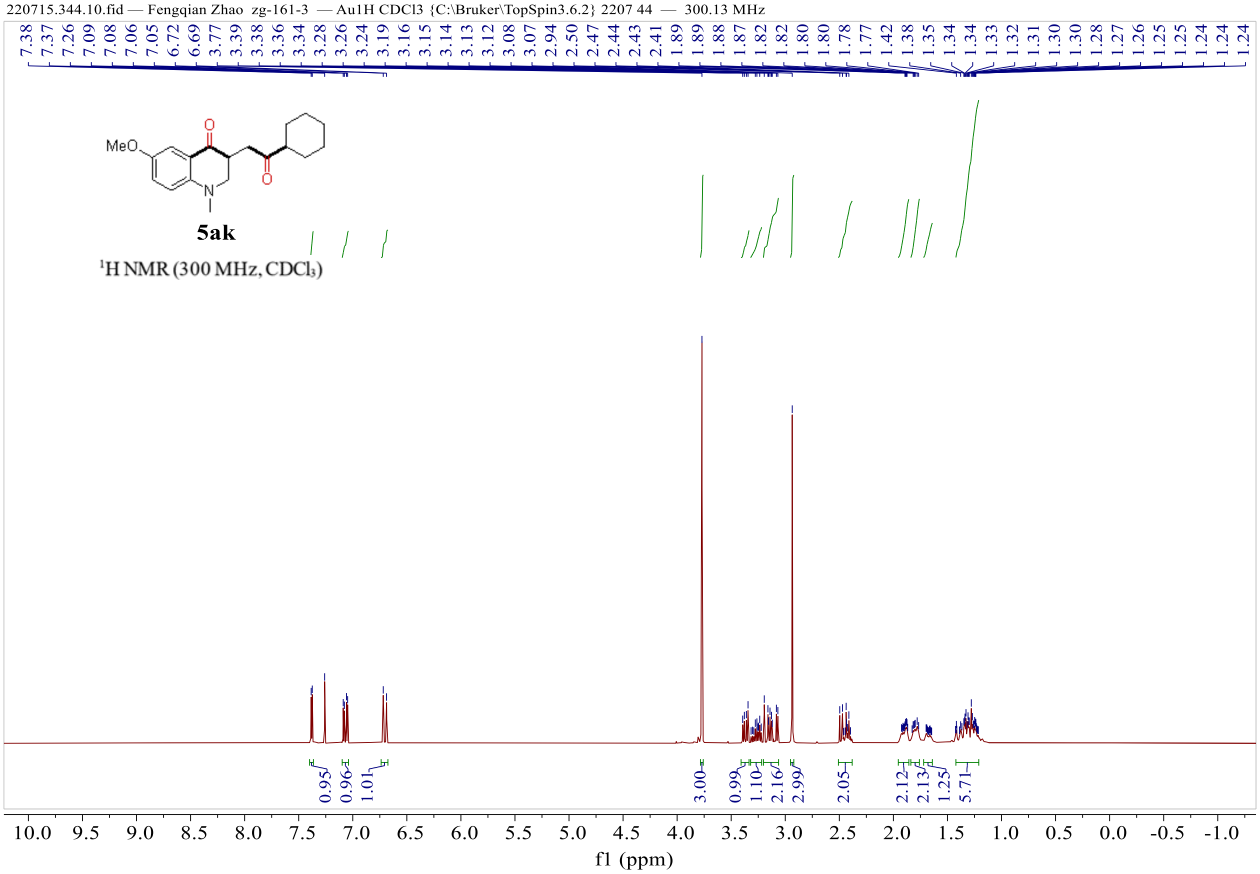

220715.344.10.fid — Fengqian Zhao zg-161-3 — Au1H CDCl3 {C:\Bruker\TopSpin3.6.2} 2207 44 — 300.13 MHz
MeO
O
N
5ak
^{1}H NMR (300 MHz, CDCl$_3$)
f1 (ppm)

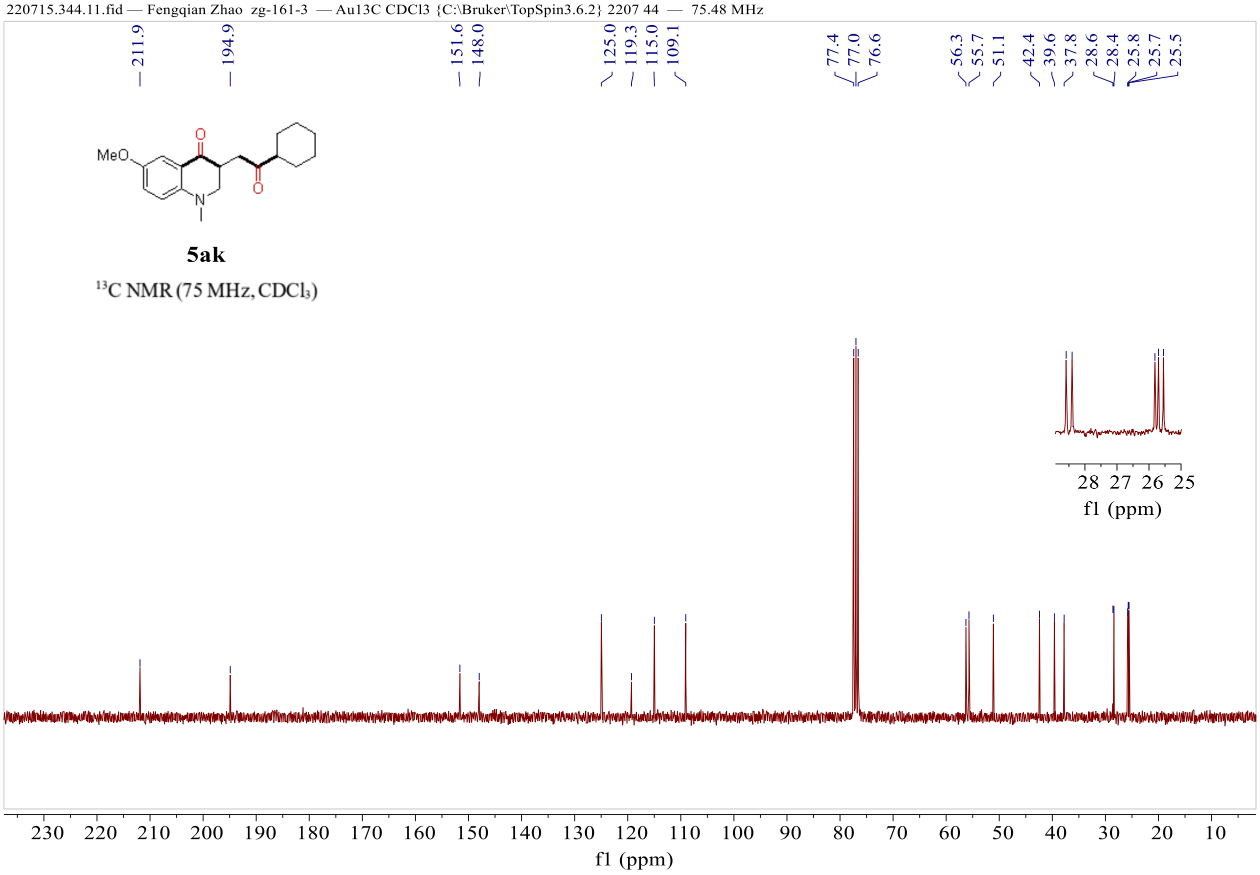

220715.344.11.fid — Fengqian Zhao zg-161-3 — Au13C CDCl3 {C:\Bruker\TopSpin3.6.2} 2207 44 — 75.48 MHz
211.9
194.9
151.6
148.0
125.0
119.3
115.0
109.1
77.4
77.0
76.6
56.3
55.7
51.1
42.4
39.6
37.8
28.6
28.4
25.8
25.7
25.5
MeO
O
N
5ak
^{13}C NMR (75 MHz, CDCl$_3$)
f1 (ppm)

220705.312.10.fid — Fengqian Zhao zg-164-7 — Au1H CDCl3 {C:\Bruker\TopSpin3.6.2} 2207 12 — 300.13 MHz

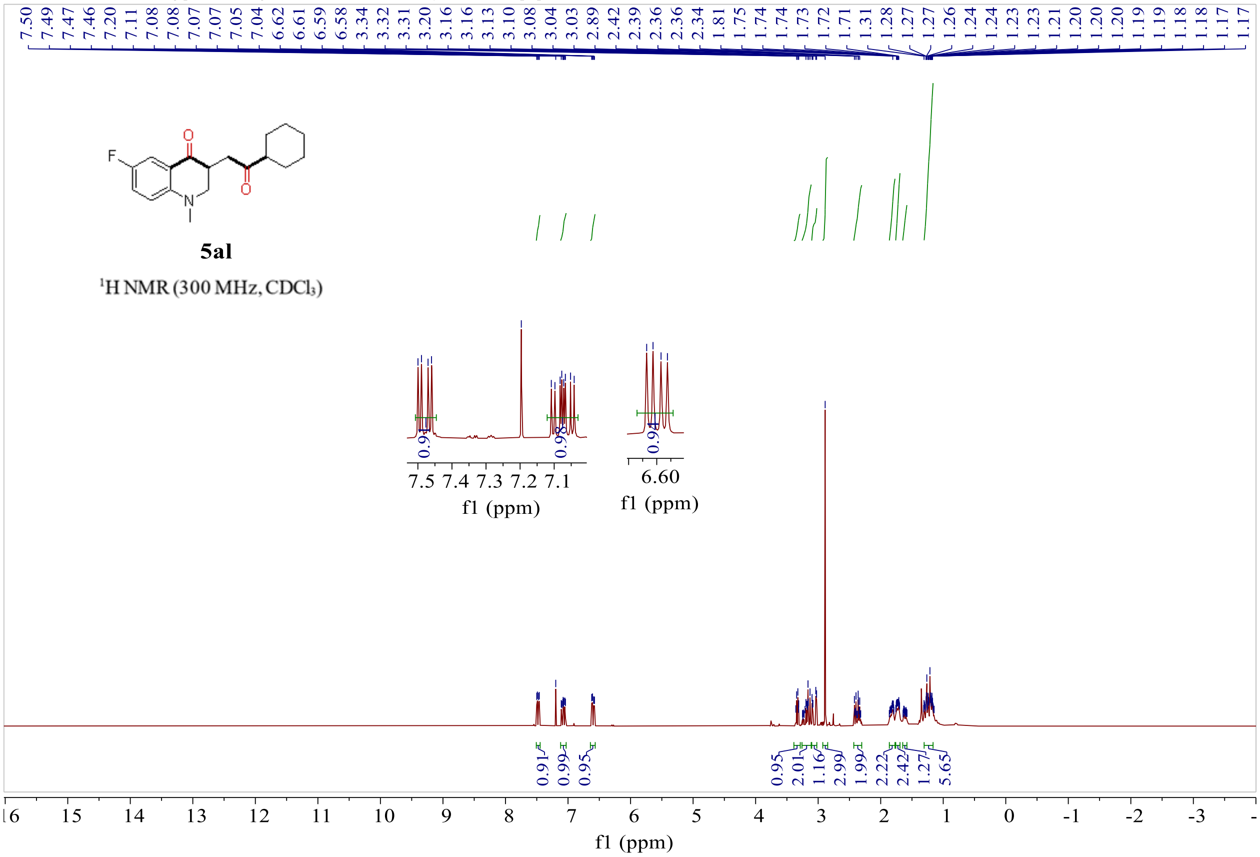

220706.304.10.fid — Fengqian Zhao zg-164-7 — Au13C CDCl3 {C:\Bruker\TopSpin3.6.2} 2207 4 — 75.48 MHz

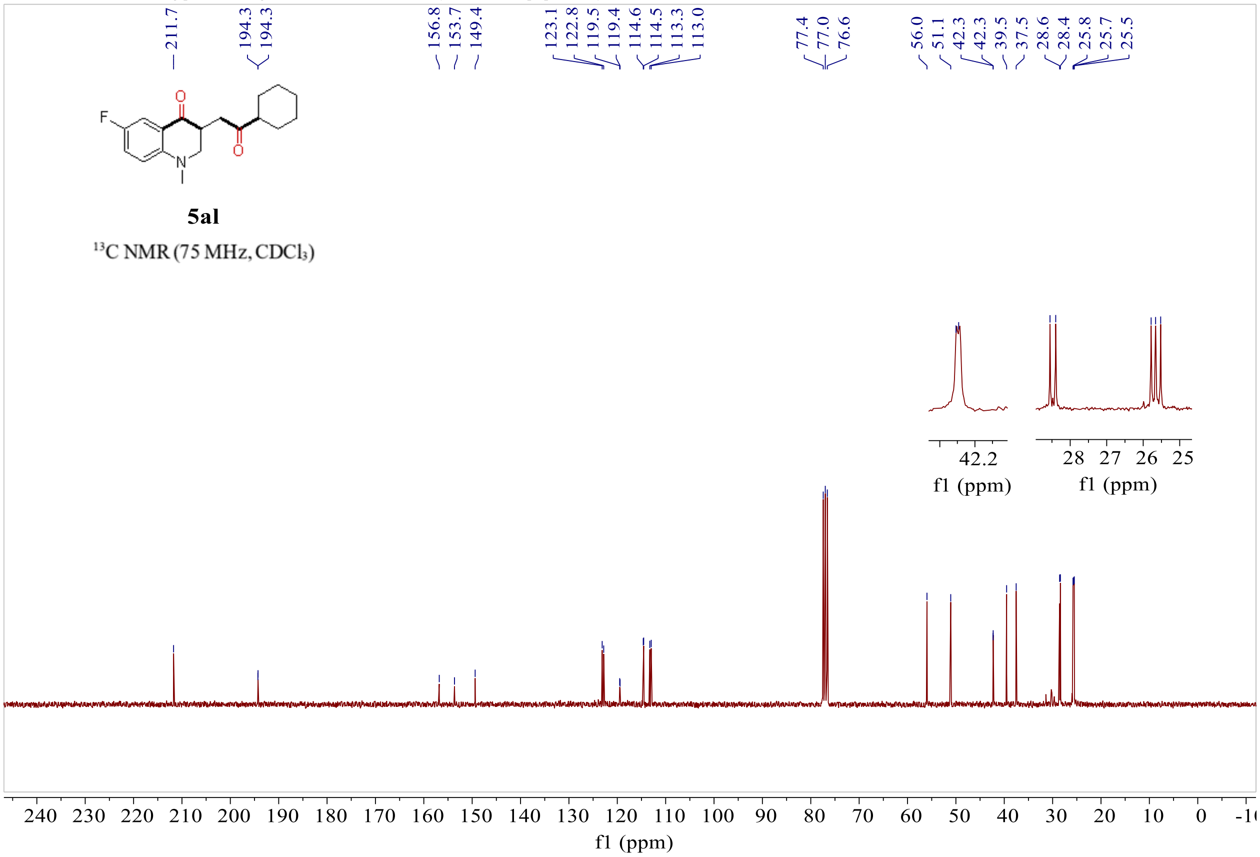

220705.312.11.fid — Fengqian Zhao zg-164-7 — Au19F CDCl3 {C:\Bruker\TopSpin3.6.2} 2207 12 — 282.39 MHz

5al

^{19}F NMR (282 MHz, CDCl$_3$)

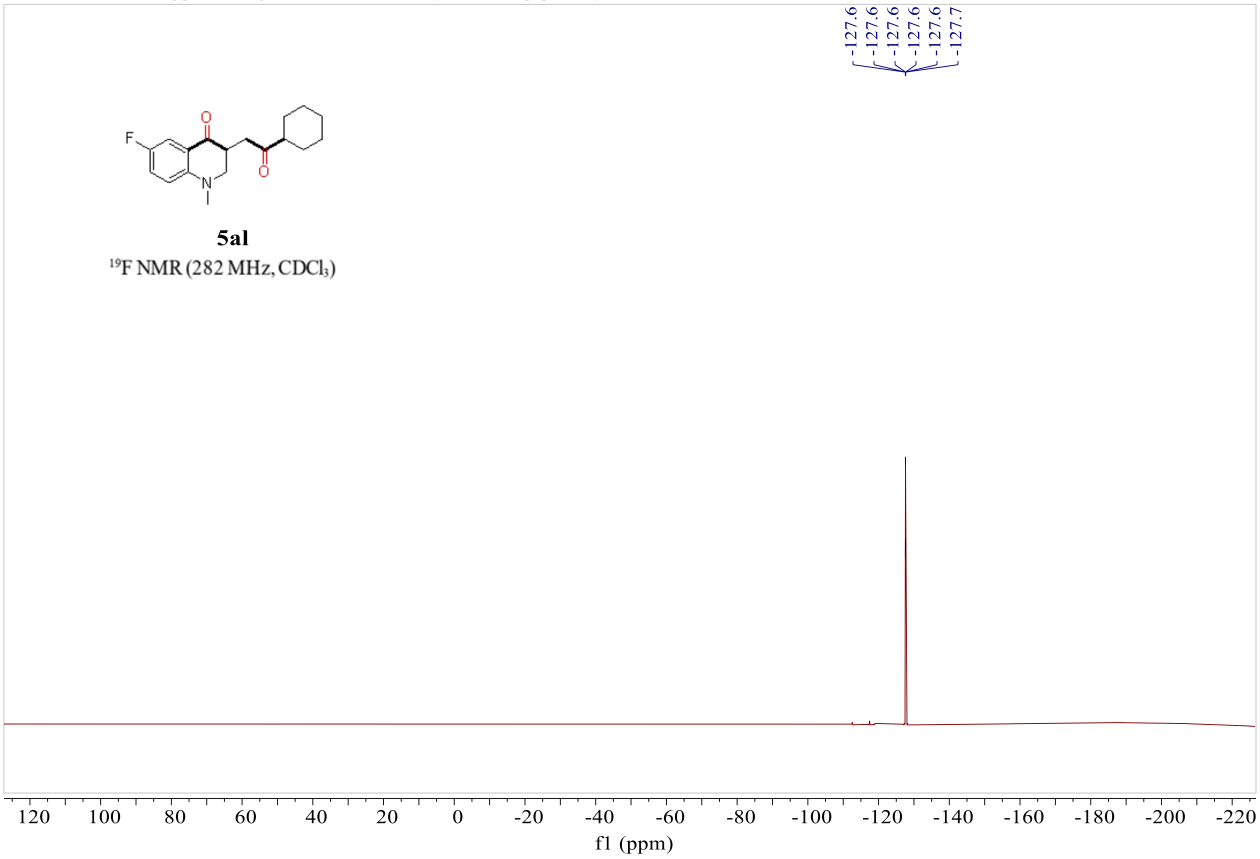

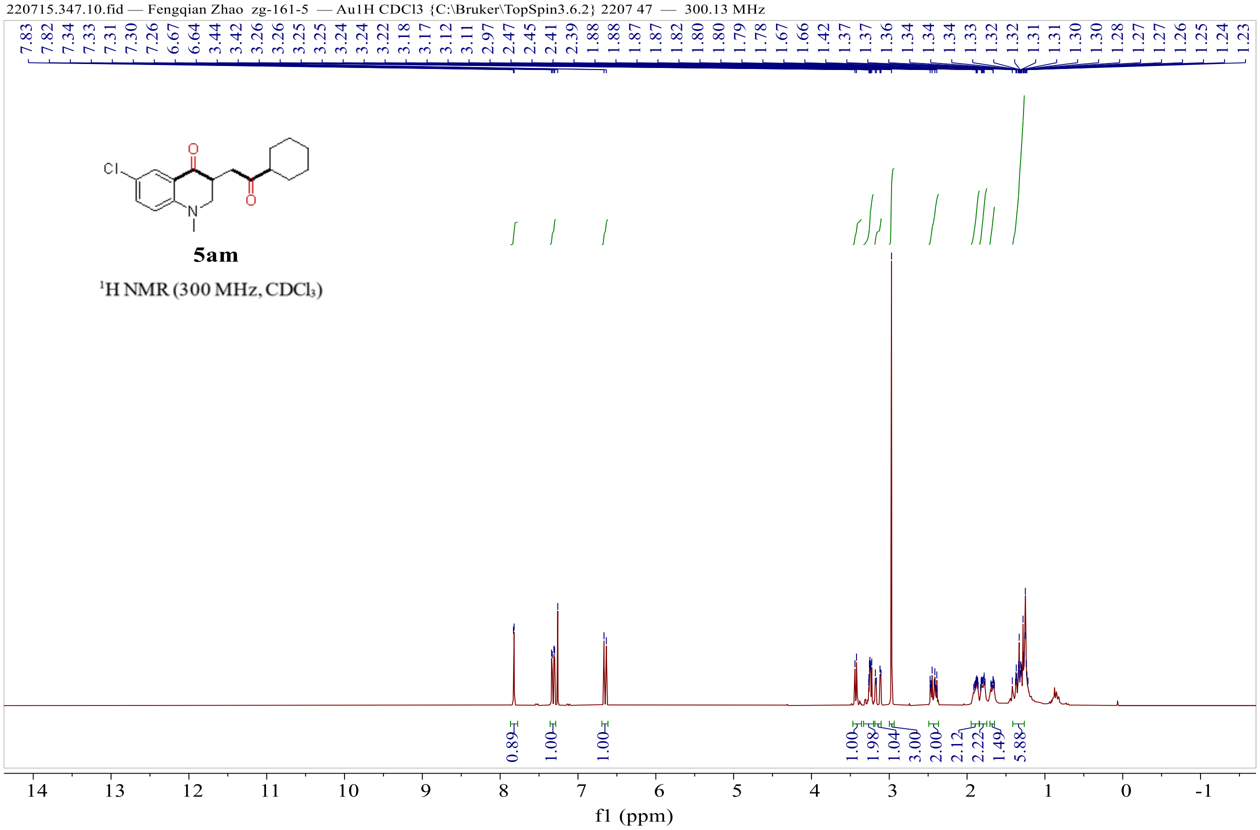
220715.347.10.fid — Fengqian Zhao zg-161-5 — Au1H CDCl3 {C:\Bruker\TopSpin3.6.2} 2207 47 — 300.13 MHz
5am
1H NMR (300 MHz, CDCl3)
f1 (ppm)

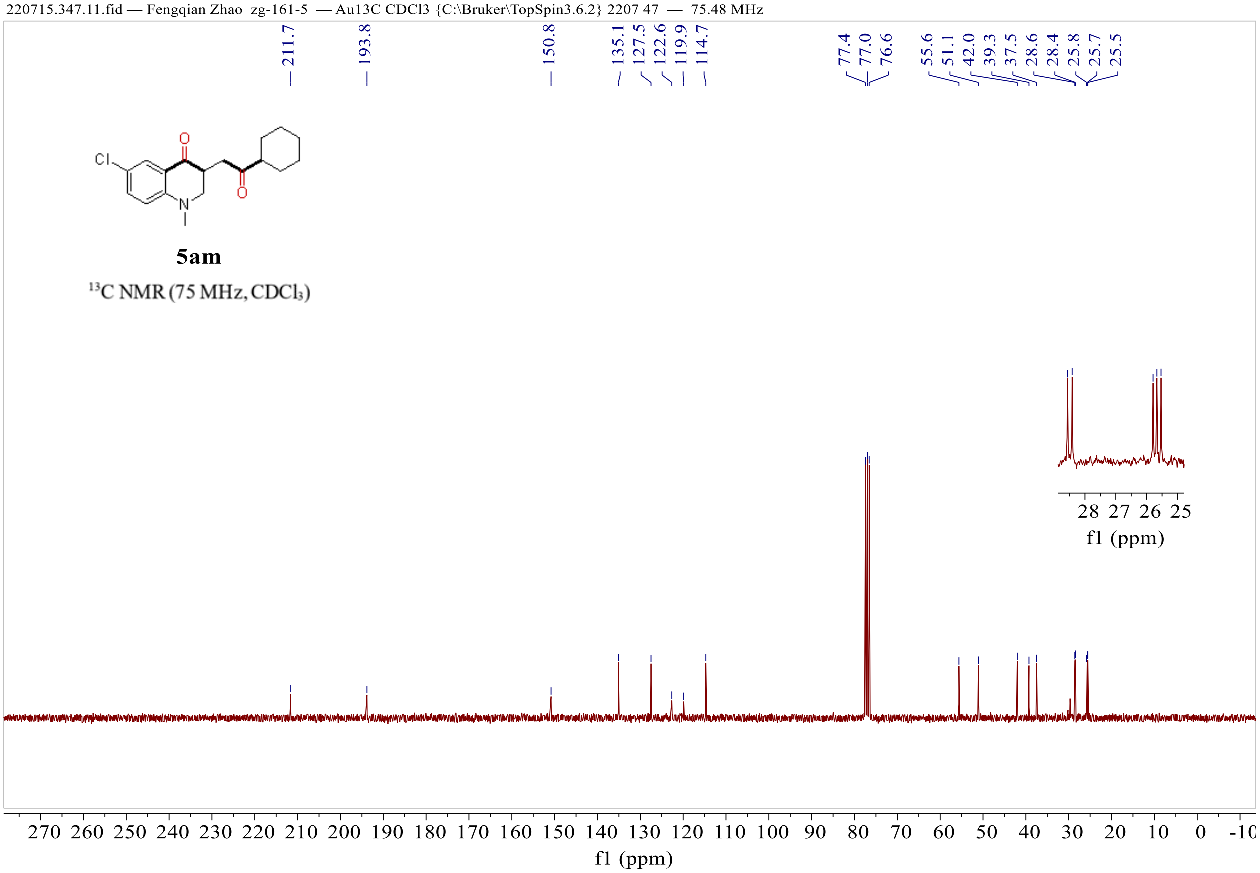
220715.347.11.fid — Fengqian Zhao zg-161-5 — Au13C CDCl3 {C:\Bruker\TopSpin3.6.2} 2207 47 — 75.48 MHz
211.7
193.8
150.8
135.1
127.5
122.6
119.9
114.7
77.4
77.0
76.6
55.6
51.1
42.0
39.3
37.5
28.6
28.4
25.8
25.7
25.5
5am
13C NMR (75 MHz, CDCl3)
f1 (ppm)

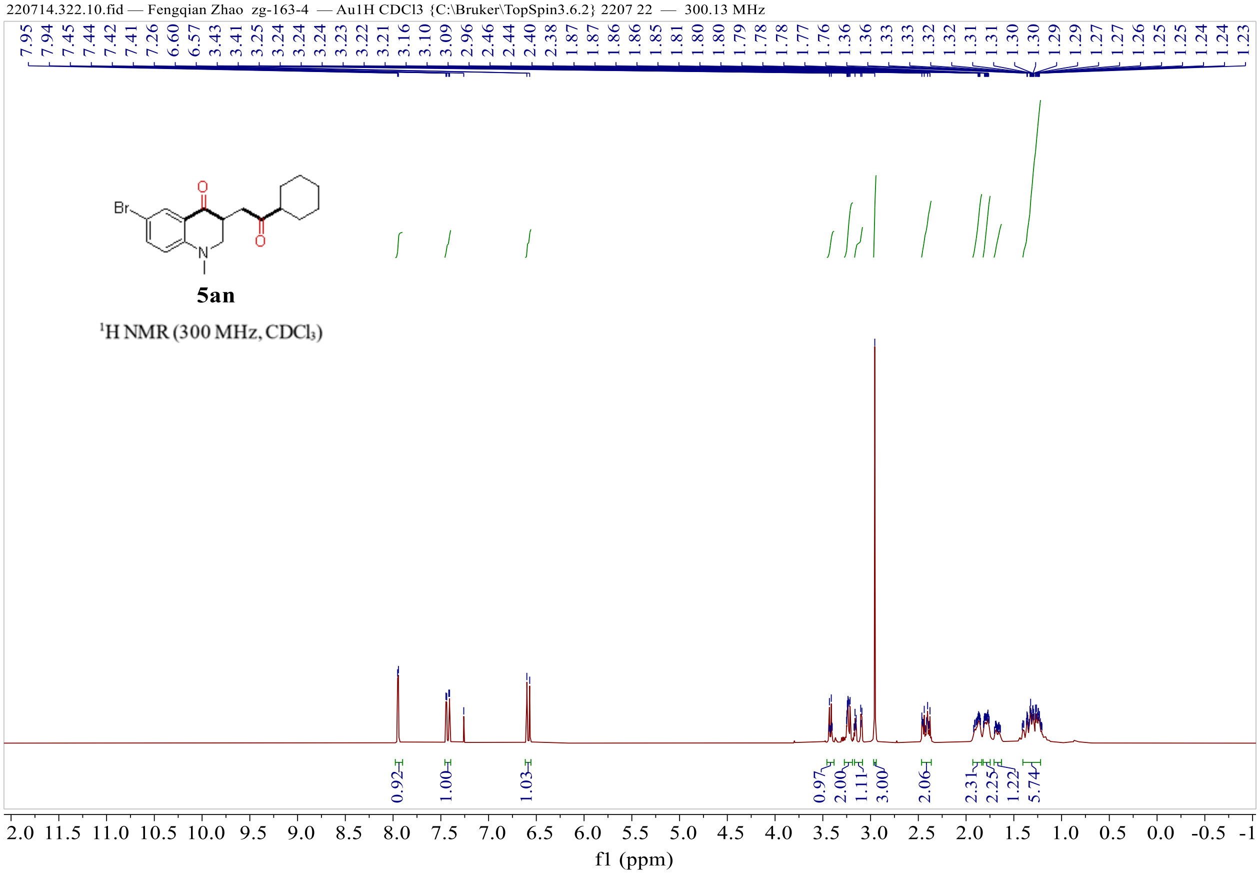
220714.322.10.fid — Fengqian Zhao zg-163-4 — Au1H CDCl3 {C:\Bruker\TopSpin3.6.2} 2207 22 — 300.13 MHz
7.95 7.94 7.45 7.44 7.42 7.41 7.26 6.60 6.57 3.43 3.41 3.25 3.24 3.24 3.24 3.23 3.22 3.21 3.16 3.10 3.09 2.96 2.46 2.44 2.40 2.38 1.87 1.87 1.86 1.86 1.85 1.81 1.80 1.80 1.79 1.78 1.78 1.77 1.76 1.36 1.36 1.33 1.33 1.32 1.32 1.31 1.31 1.30 1.30 1.29 1.29 1.27 1.27 1.26 1.25 1.25 1.24 1.24 1.23
Br
O
N
5an
¹H NMR (300 MHz, CDCl₃)
0.92 1.00 1.03 0.97 2.00 1.11 3.00 2.06 2.31 2.25 1.22 5.74
f1 (ppm)

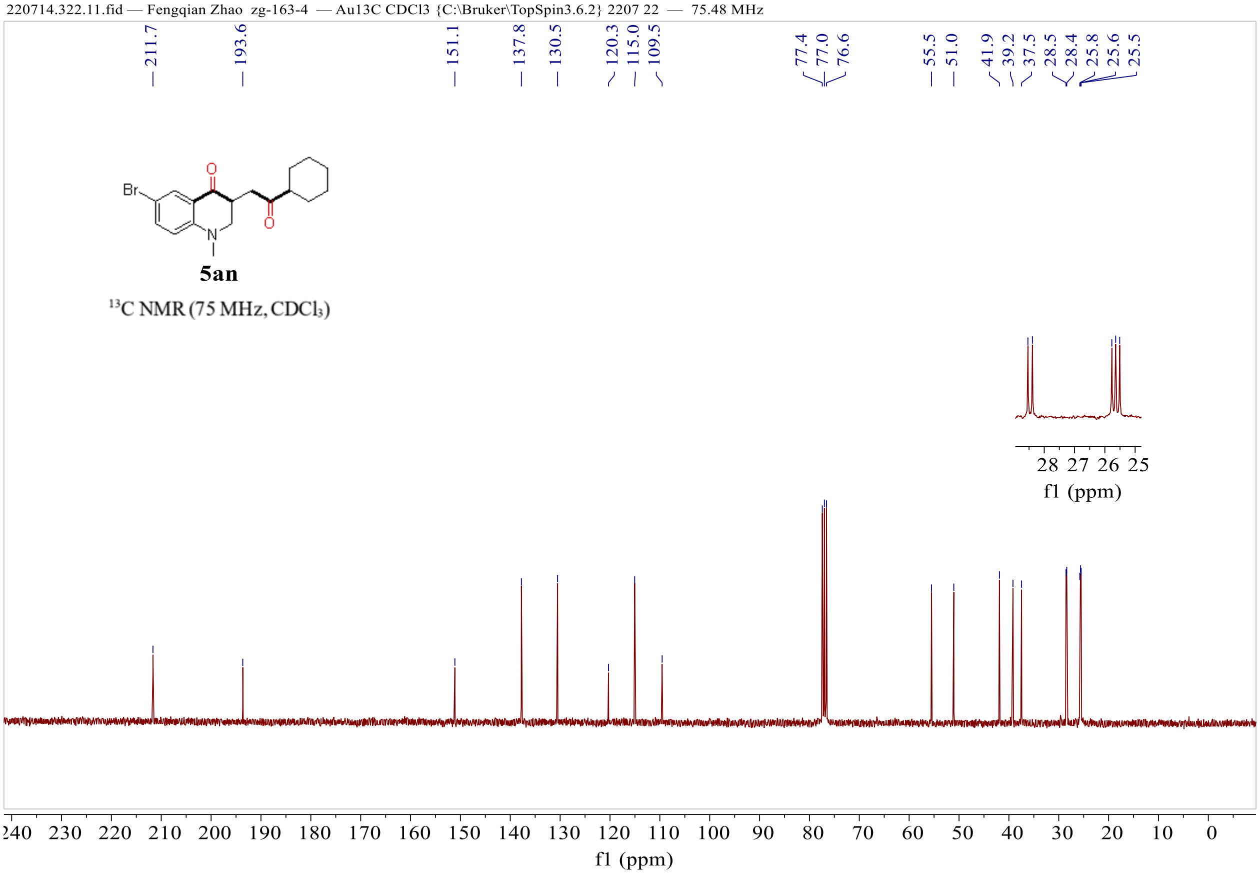
220714.322.11.fid — Fengqian Zhao zg-163-4 — Au13C CDCl3 {C:\Bruker\TopSpin3.6.2} 2207 22 — 75.48 MHz
211.7 193.6 151.1 137.8 130.5 120.3 115.0 109.5 77.4 77.0 76.6 55.5 51.0 41.9 39.2 37.5 28.5 28.4 25.8 25.6 25.5
Br
O
N
5an
¹³C NMR (75 MHz, CDCl₃)
28 27 26 25
f1 (ppm)

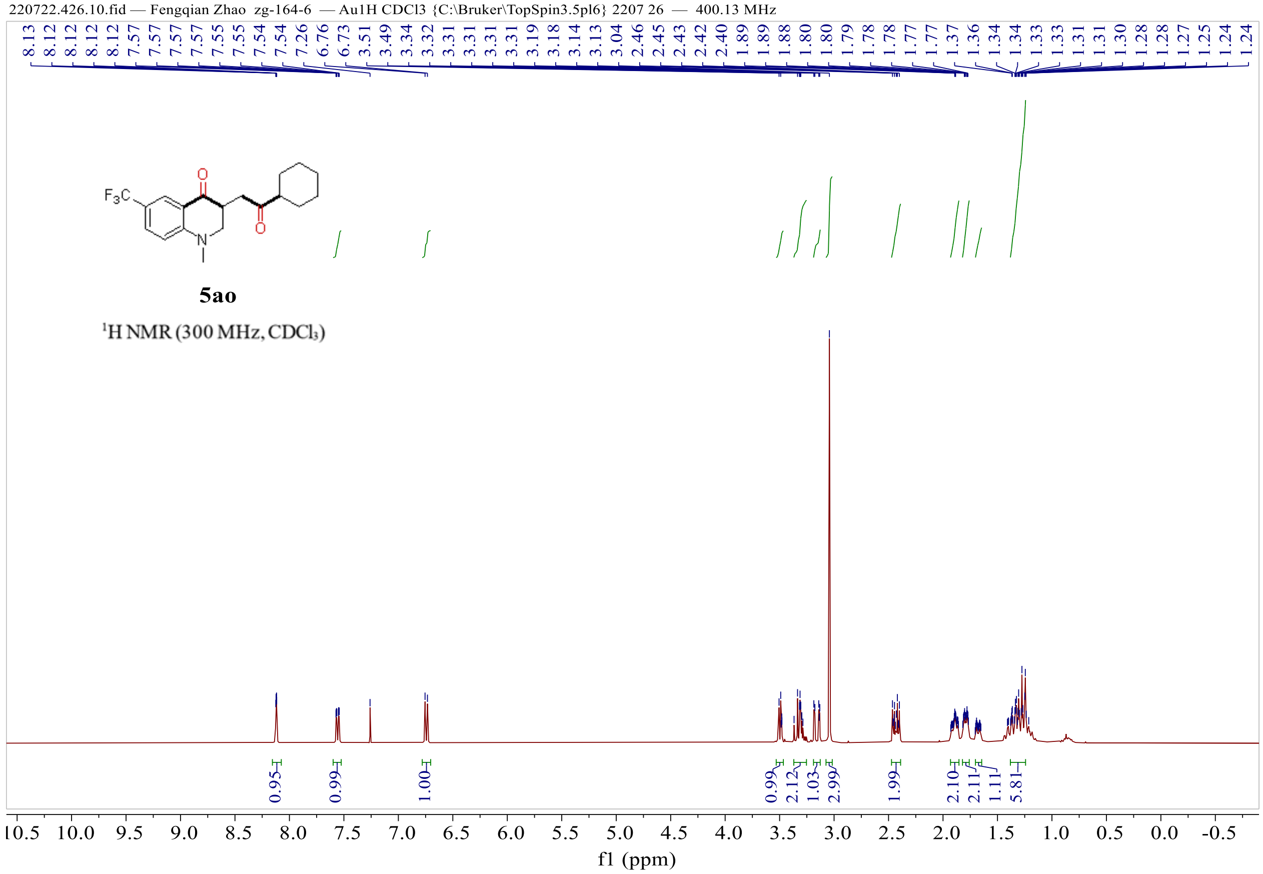
220722.426.10.fid — Fengqian Zhao zg-164-6 — Au1H CDCl3 {C:\Bruker\TopSpin3.5pl6} 2207 26 — 400.13 MHz
5ao
1H NMR (300 MHz, CDCl3)
f1 (ppm)

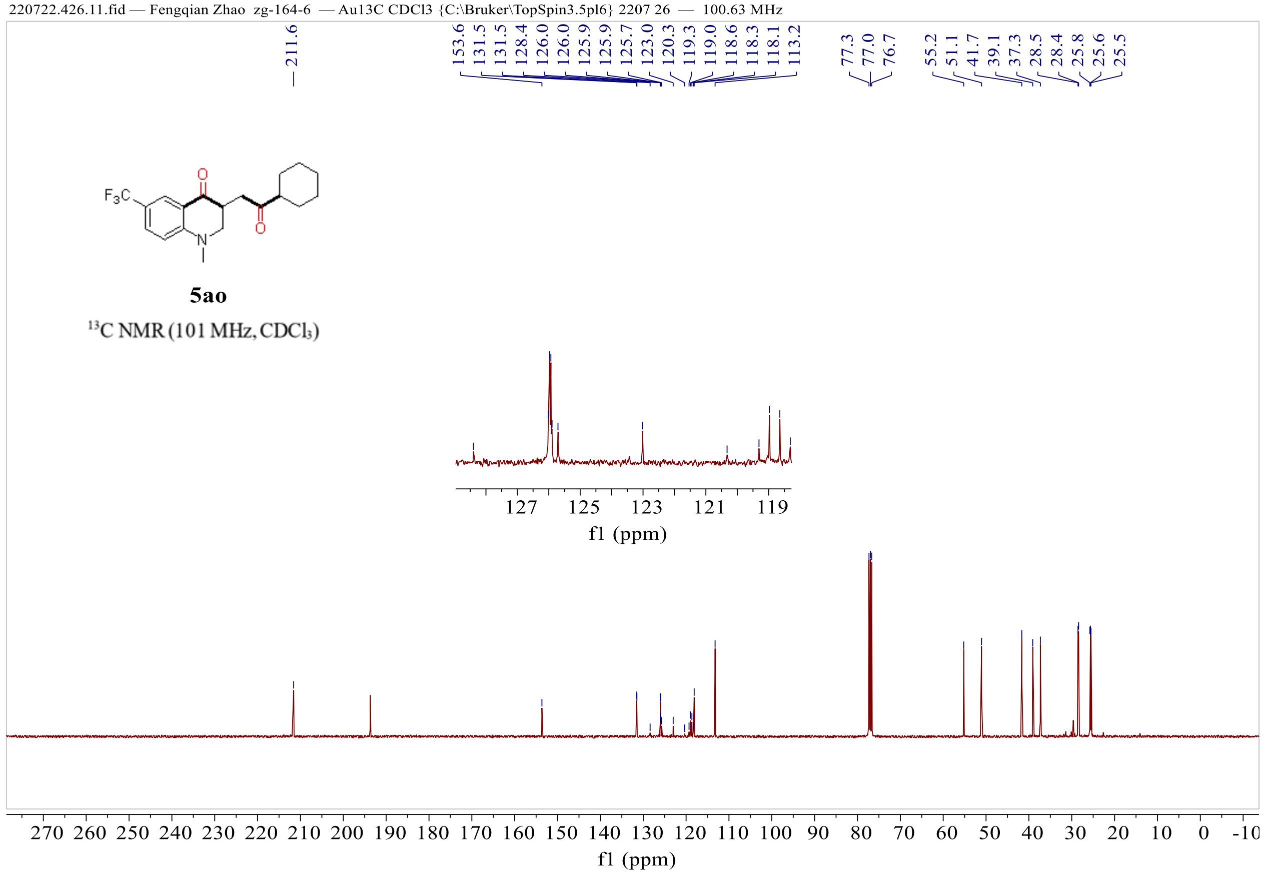
220722.426.11.fid — Fengqian Zhao zg-164-6 — Au13C CDCl3 {C:\Bruker\TopSpin3.5pl6} 2207 26 — 100.63 MHz
5ao
13C NMR (101 MHz, CDCl3)
f1 (ppm)

220705.313.11.fid — Fengqian Zhao zg-164-6 — Au19F CDCl3 {C:\Bruker\TopSpin3.6.2} 2207 13 — 282.39 MHz

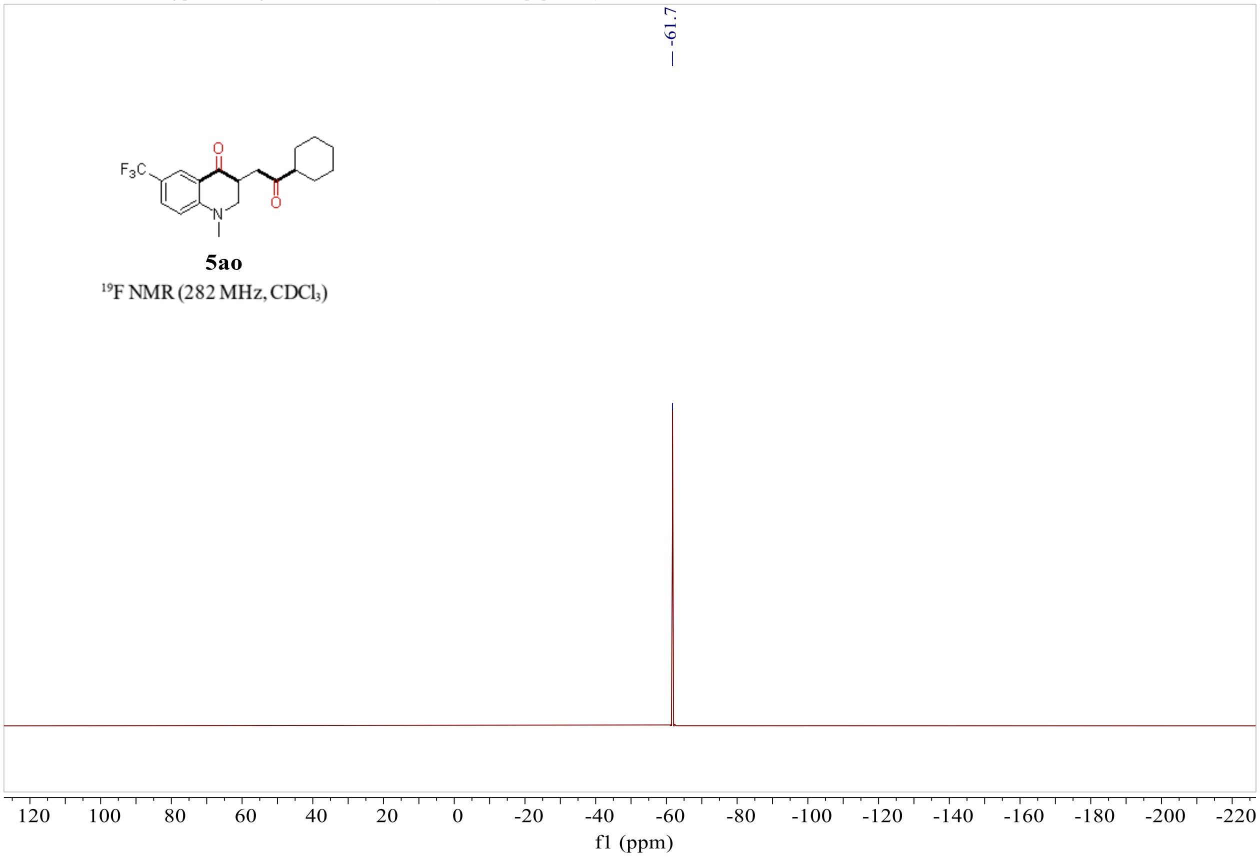

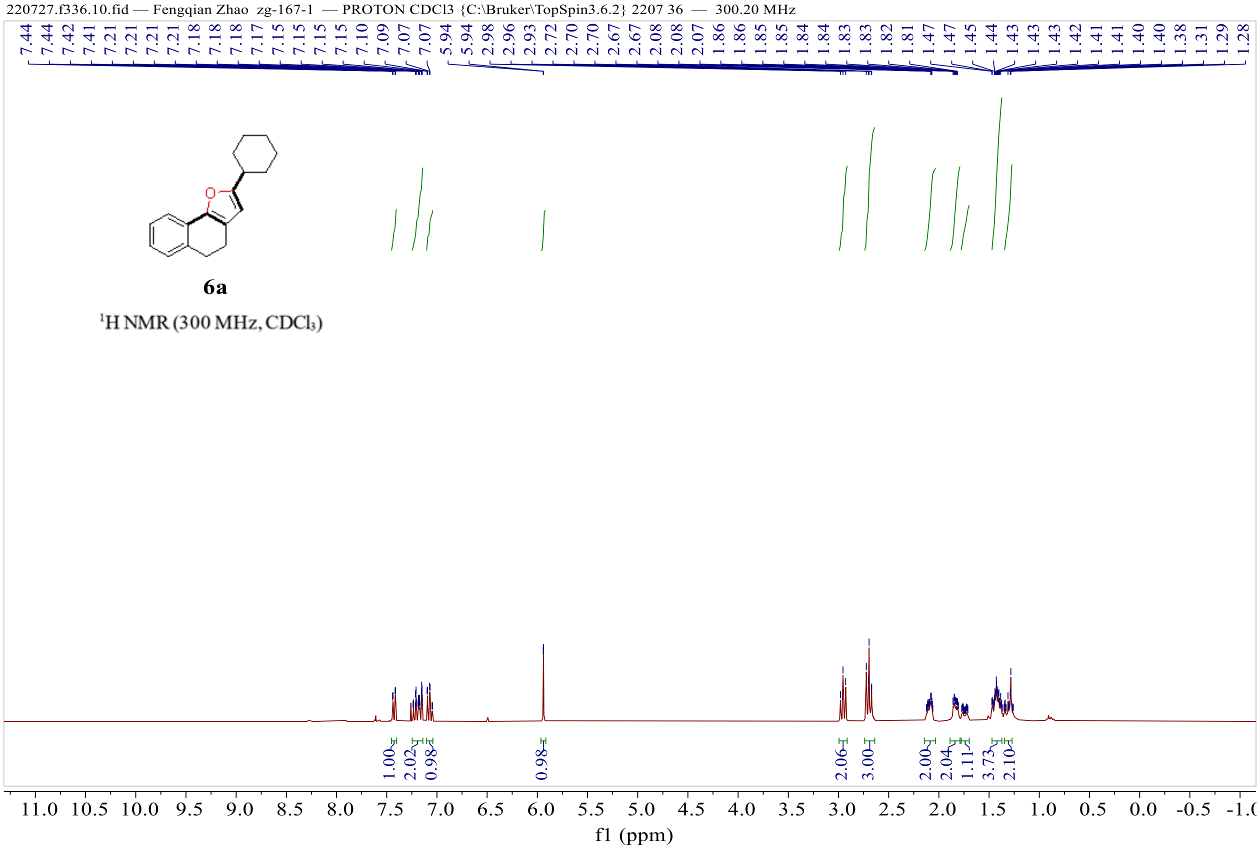
220727.f336.10.fid — Fengqian Zhao zg-167-1 — PROTON CDCl3 {C:\Bruker\TopSpin3.6.2} 2207 36 — 300.20 MHz
6a
^{1}H NMR (300 MHz, $CDCl_3$)
f1 (ppm)

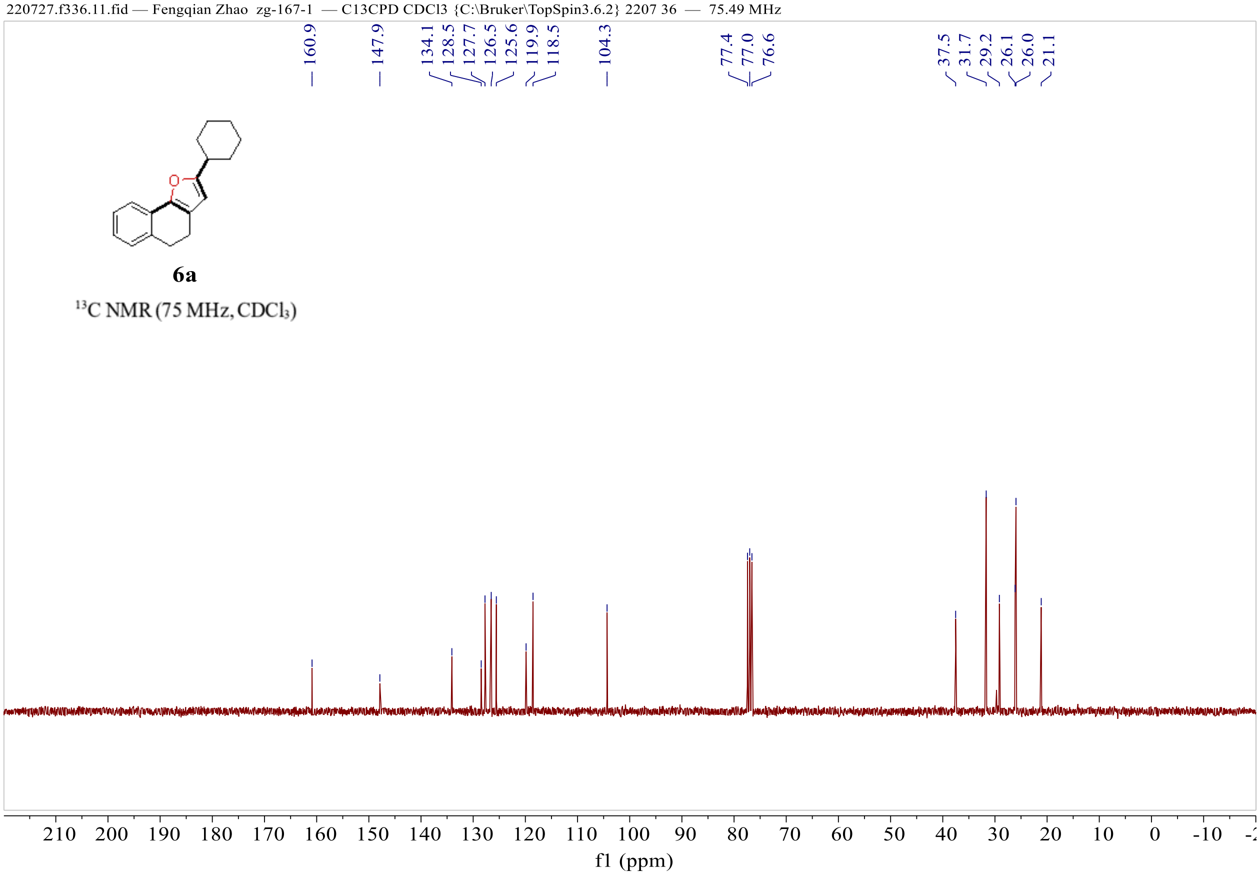
220727.f336.11.fid — Fengqian Zhao zg-167-1 — C13CPD CDCl3 {C:\Bruker\TopSpin3.6.2} 2207 36 — 75.49 MHz
160.9
147.9
134.1
128.5
127.7
126.5
125.6
119.9
118.5
104.3
77.4
77.0
76.6
37.5
31.7
29.2
26.1
26.0
21.1
6a
^{13}C NMR (75 MHz, $CDCl_3$)
f1 (ppm)

220804.314.10.fid — Fengqian Zhao zg-167-6 — Au1H CDCl3 {C:\Bruker\TopSpin3.6.2} 2208 14 — 300.13 MHz

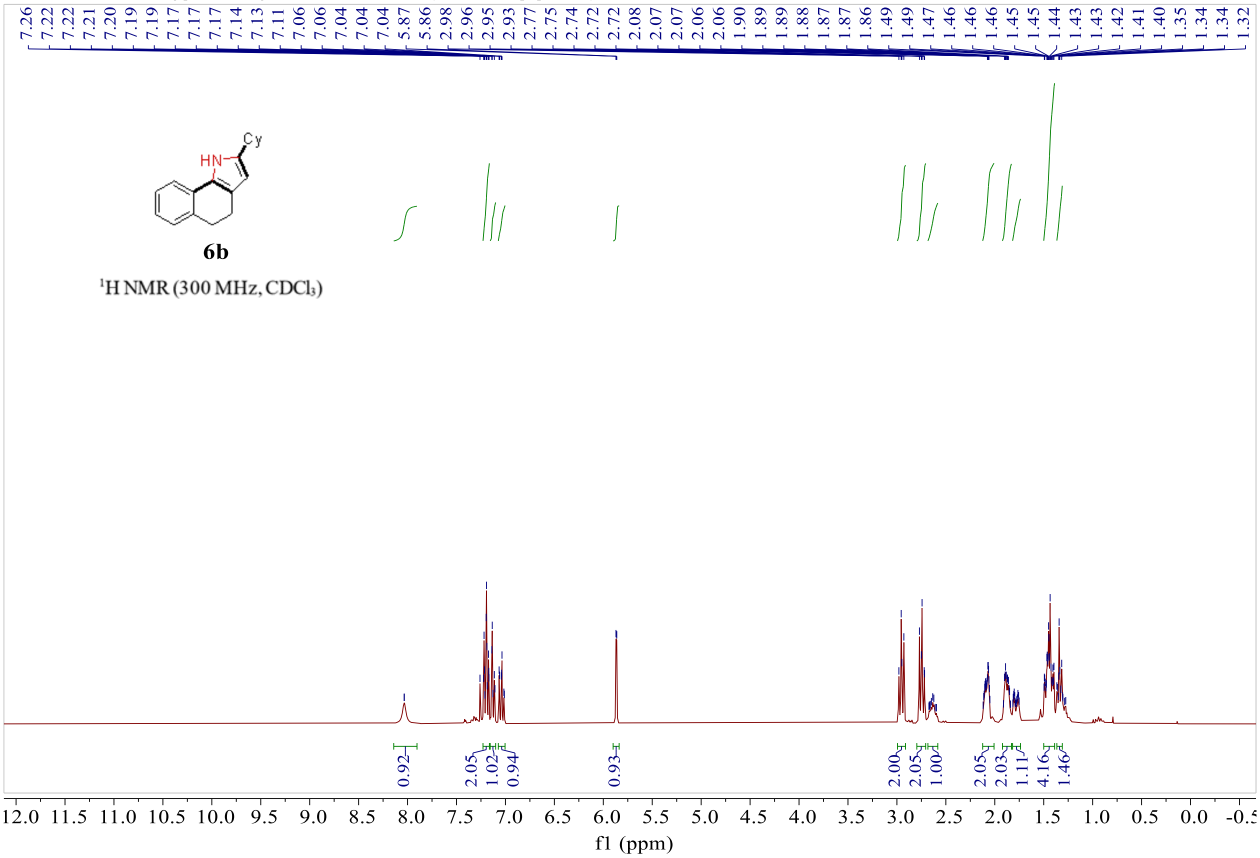

220804.314.11.fid — Fengqian Zhao zg-167-6 — Au13C CDCl3 {C:\Bruker\TopSpin3.6.2} 2208 14 — 75.48 MHz

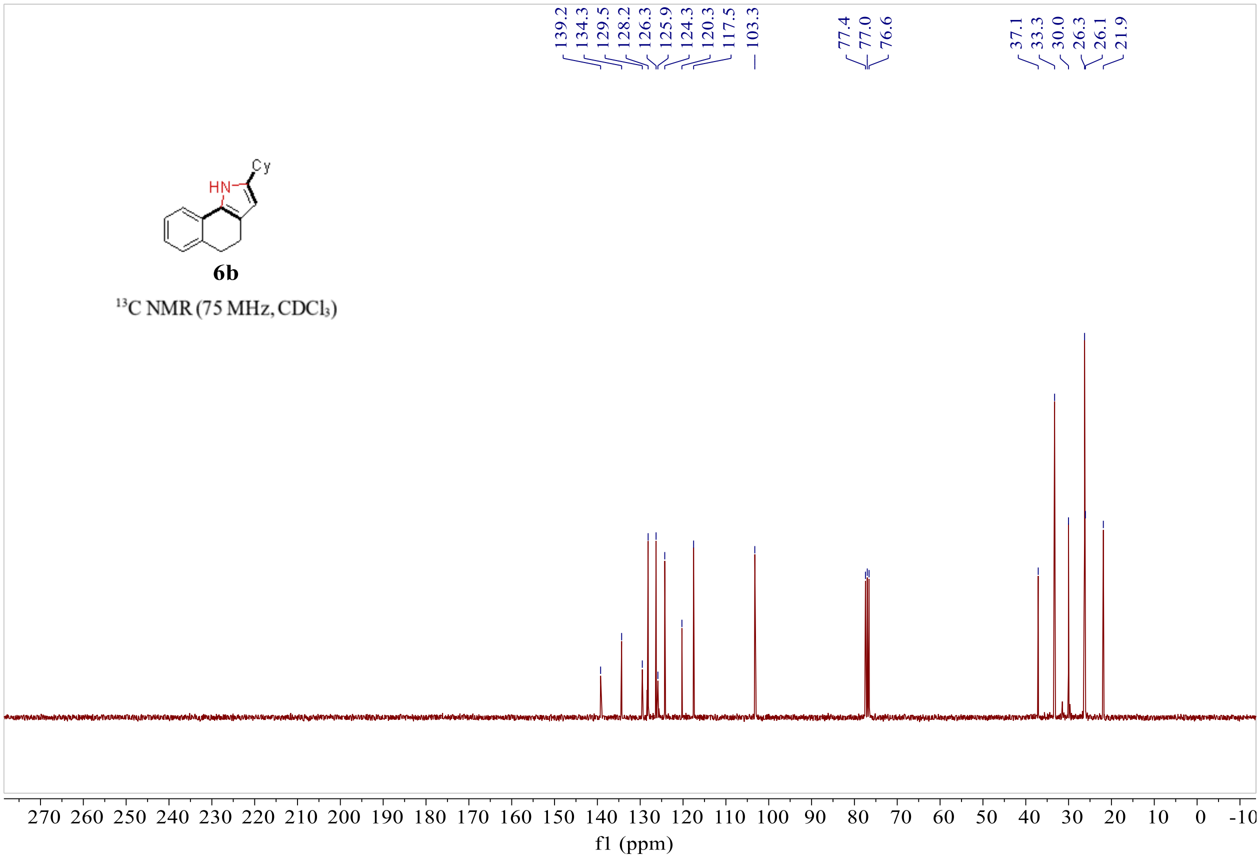

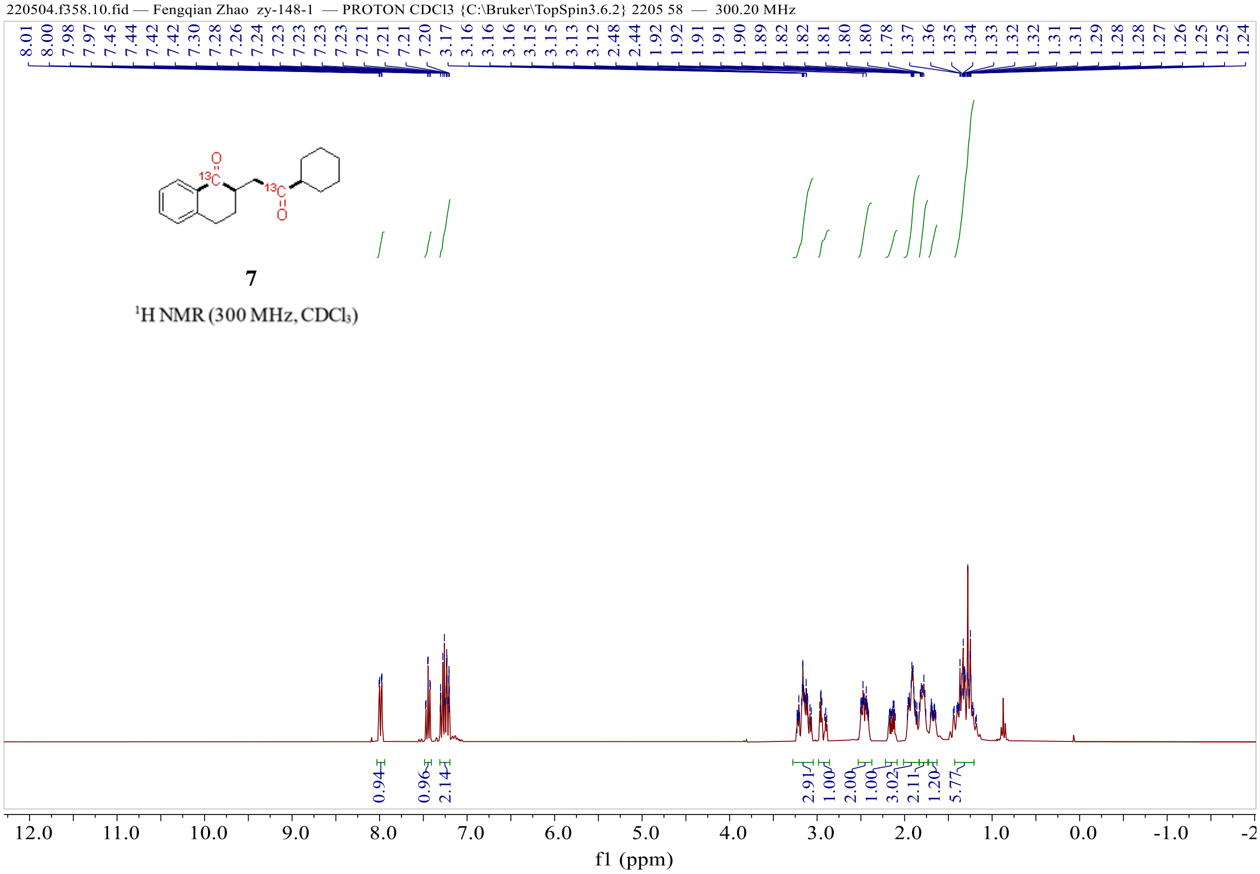
220504.f358.10.fid — Fengqian Zhao zy-148-1 — PROTON CDCl3 {C:\Bruker\TopSpin3.6.2} 2205 58 — 300.20 MHz
7
^{1}H NMR (300 MHz, CDCl$_3$)
f1 (ppm)

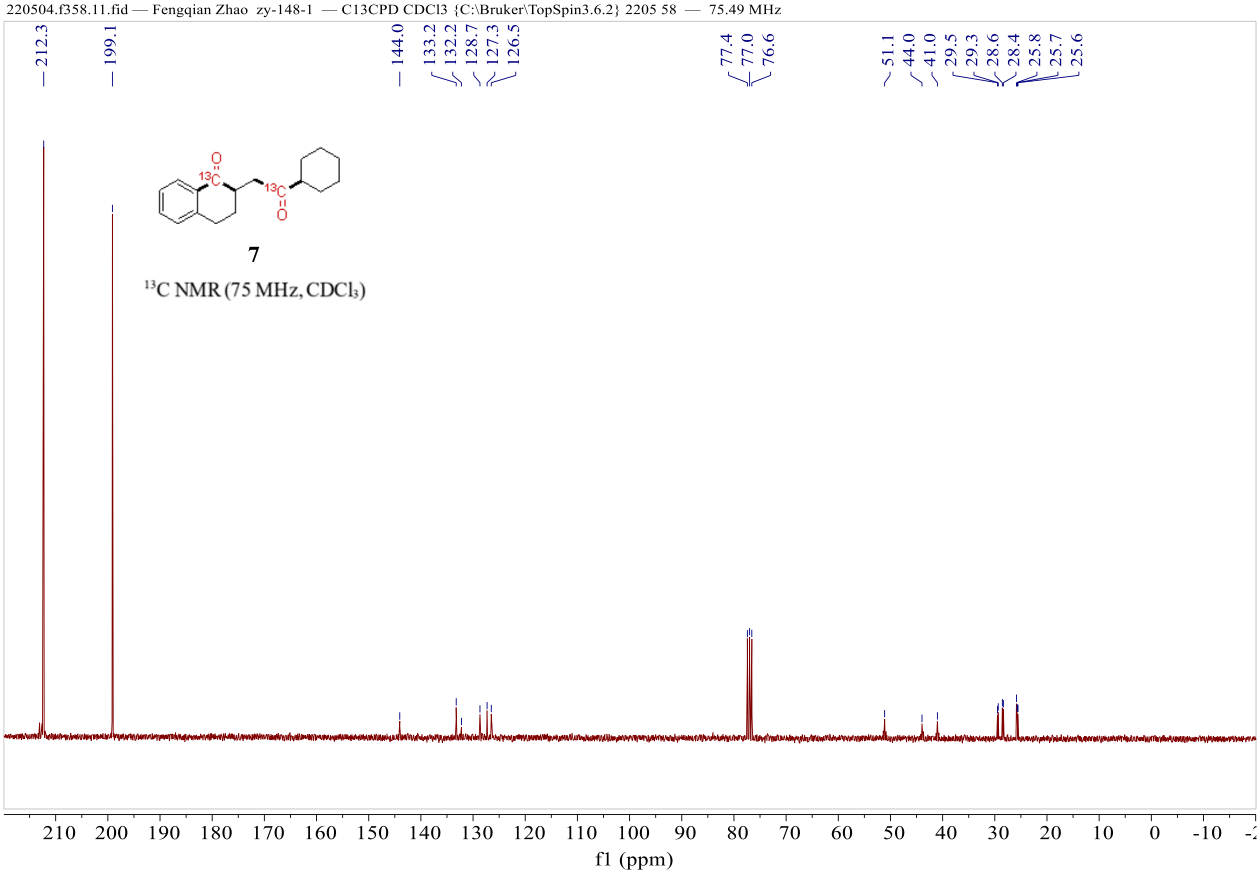
220504.f358.11.fid — Fengqian Zhao zy-148-1 — C13CPD CDCl3 {C:\Bruker\TopSpin3.6.2} 2205 58 — 75.49 MHz
212.3
199.1
144.0
133.2
132.2
128.7
127.3
126.5
77.4
77.0
76.6
51.1
44.0
41.0
29.5
29.3
28.6
28.4
25.8
25.7
25.6
7
^{13}C NMR (75 MHz, CDCl$_3$)
f1 (ppm)
